# Adaptive responses of yeast strains tolerant to acidic pH, acetate, and supraoptimal temperature

**DOI:** 10.1007/s00253-023-12556-7

**Published:** 2023-05-13

**Authors:** Prisciluis Caheri Salas-Navarrete, Paul Rosas-Santiago, Ramón Suárez-Rodríguez, Alfredo Martínez, Luis Caspeta

**Affiliations:** 1 Centro de Investigación en Biotecnología, Universidad Autónoma del Estado de Morelos, Av. Universidad 1001, Col. Chamilpa, Cuernavaca, 62209 Morelos México; 2 Departamento de Biología Molecular de Plantas, Instituto de Biotecnología, Universidad Nacional Autónoma de México, Av. Universidad 2001, Col. Chamilpa, Cuernavaca, 62210 Morelos México; 3 Departamento de Ingeniería Celular y Biocatálisis, Instituto de Biotecnología, Universidad Nacional Autónoma de México, Av. Universidad 2001, Col. Chamilpa, Cuernavaca, 62210 Morelos México

**Keywords:** *Saccharomyces cerevisiae*, Adaptive laboratory evolution, Thermo-acidic tolerance, Adaptive cellular responses, Genome-scale analysis

## Abstract

**Abstract:**

Ethanol fermentations can be prematurely halted as *Saccharomyces cerevisiae* faces adverse conditions, such as acidic pH, presence of acetic acid, and supraoptimal temperatures. The knowledge on yeast responses to these conditions is essential to endowing a tolerant phenotype to another strain by targeted genetic manipulation. In this study, physiological and whole-genome analyses were conducted to obtain insights on molecular responses which potentially render yeast tolerant towards thermoacidic conditions. To this end, we used thermotolerant TTY23, acid tolerant AT22, and thermo-acid tolerant TAT12 strains previously generated by adaptive laboratory evolution (ALE) experiments. The results showed an increase in thermoacidic profiles in the tolerant strains. The whole-genome sequence revealed the importance of genes related to: H^+^, iron, and glycerol transport (i.e., *PMA1*, *FRE1/2*, *JEN1*, *VMA2*, *VCX1*, *KHA1*, *AQY3*, and *ATO2*); transcriptional regulation of stress responses to drugs, reactive oxygen species and heat-shock (i.e., *HSF1*, *SKN7*, *BAS1*, *HFI1*, and *WAR1*); and adjustments of fermentative growth and stress responses by glucose signaling pathways (i.e., *ACS1*, *GPA1/2*, *RAS2*, *IRA2*, and *REG1*). At 30 °C and pH 5.5, more than a thousand differentially expressed genes (DEGs) were identified in each strain. The integration of results revealed that evolved strains adjust their intracellular pH by H^+^ and acetic acid transport, modify their metabolism and stress responses via glucose signaling pathways, control of cellular ATP pools by regulating translation and de novo synthesis of nucleotides, and direct the synthesis, folding and rescue of proteins throughout the heat-shock stress response. Moreover, the motifs analysis in mutated transcription factors suggested a significant association of SFP1, YRR1, BAS1, HFI1, HSF1, and SKN7 TFs with DEGs found in thermoacidic tolerant yeast strains.

**Key points:**

• *All the evolved strains overexpressed the plasma membrane H*^*+*^ *-ATPase PMA1 at optimal conditions*

• *Tolerant strain TAT12 mutated genes encoding weak acid and heat response TFs HSF1, SKN7, and WAR1*

• *TFs HSF1 and SKN7 likely controlled the transcription of metabolic genes associated to heat and acid tolerance*

**Supplementary Information:**

The online version contains supplementary material available at 10.1007/s00253-023-12556-7.

## Introduction

The yeast *Saccharomyces cerevisiae* has been widely used in vinification and industrial production of ethanol. In both processes, the yeast could be simultaneously exposed to supraoptimal temperatures, suboptimal pH, and acetic acid (Della-Bianca and Gombert 2013; Ough and Amerine 1966; Palmqvist and Hahn-Hägerdal 2000). These conditions hinder yeast growth and lifespan, which consequently inhibits or prematurely stops ethanol fermentation (Pampulha and Loureiro-Dias 1989; Pinto et al. 1989; Taherzadeh et al. 1997; Van Uden 1985). Therefore, engineered yeast strains with improved tolerance to thermoacidic conditions are more likely to successfully perform in ethanol fermentations. Yet, the engineering of these strains is challenging, as multiple genetic modifications may be required to obtain tolerant strains. In this context, knowledge on the basic adaptive responses is imperative in directing engineering efforts.

Currently, it is known that supraoptimal temperatures become lethal alongside the presence of acetic acid and suboptimal pH (Pinto et al. 1989). It is important to note that the internal pH of yeast is tightly regulated at 7.2 (Orij et al. 2011); however, a transient acidification can result from a mild increase in temperature as well as with the addition of sublethal pulses of acetic acid in media with low pH (Coote et al. 1991; Pampulha and Loureiro-Dias 1989; Triandafillou et al. 2020). Furthermore, as the concentration of acetic acid increased, a permanent decrease in pH is also observed (Ullah et al. 2012). Moreover, growth was gradually inhibited and eventually halted when the yeast’s cytosolic pH reached values below 5.5 (Fernández-Niño et al. 2015; Giannattasio et al. 2013; Ullah et al. 2012). Additionally, at elevated temperatures, the rate of growth decay caused by acetic acid is also exacerbated (Pinto et al. 1989). For instance, at 30 °C *S. cerevisiae* grew at a pH of 2.5–2.8 (Orij et al. 2009; Van Uden 1985), while at 39 °C, an increase of the pH value to at least 3.3 was necessary to allow its growth (Pinto et al. 1989). Finally, at pH 3.3 alongside concentrations of 1 or 4 g/L of acetic acid, the maximum temperatures that allowed for yeast growth were 36 °C and 26 °C, respectively (Pinto et al. 1989; Taherzadeh et al. 1997).

*S. cerevisiae* counteracts cytosolic acidification by pumping H^+^ out of the cell using the plasma membrane H^+^-ATPase PMA1 (Carmelo et al. 1996; Ferreira et al. 2001; Serrano et al. 1986). The activity of PMA1 increased during an up-shift in temperature or during the addition of acetic acid (Coote et al. 1994; Ullah et al. 2012), while ATP consumption also increased substantially (Lahtvee et al. 2016; Verduyn et al. 1992; Watson 1970). To avoid excessive energy consumption, PMA1 overactivity is constrained by the heat-shock protein (HSP) HSP30, which expression increases during heat shock and the addition of ethanol or a weak acid (Meena et al. 2011; Piper et al. 1994). Furthermore, excessive ATP consumption can also be followed by an increase in glucose and oxygen uptake rates, as well as an enlargement of the mitochondrial volume (Lahtvee et al. 2016; Verduyn et al. 1992). Hence, the accumulation of reactive oxygen species (ROS) also increased alongside the activities of the superoxide dismutase and catalase (Giannattasio et al. 2005). Additionally, efficient ROS scavenging reduced mitochondrial damage and avoided a retrograde response. This was followed by lethal pulses of acetic acid to activate the programmed cell death (Giannattasio et al. 2013).

The internal pH of *S. cerevisiae* is tightly controlled at close to neutral (Orij et al. 2011), because the activity of nearly all glycolytic enzymes decreases at a pH below 6.8 (Kumar et al. 2004; Pampulha and Loureiro-Dias 1990). Interestingly, the transcription of most glycolytic genes, including hexokinase (*HXK2)* and phosphofructokinase (*PFK1*), which regulate glucose signaling pathways, increases at high temperatures, low pH and while experiencing acetic acid stresses (Almeida et al. 2009; Gasch et al. 2000; Mira et al. 2010; Schüller et al. 2004). Both of these genes had a role in glucose-induced repression of several other genes involved in stress responses (Broach 2012; Gancedo 1998). For example, deletion of *HXK2*, *GPR1* (G-protein coupled receptor)/*GPA2* (G-protein alpha subunit), and *RGT2* (restores glucose transport)/*SNF3* (sucrose non fermenting), which are the sensors for glucose signaling pathways, restored intracellular pH in response to glucose limitation (Isom et al. 2018; Orij et al. 2011), and, in turn, the limitation of glucose reduced PMA1 activity. Furthermore, mutations in *SNF1*, *TPK2* (cAMP-dependent protein kinase), and *RAS2* (GTP-binding protein), which are downstream elements of glucose-signaling pathways, increased cytosolic acidification (Isom et al. 2018). Additionally, the downregulation of *RAS2*, *TPK1*, and *TPK3*, which are key elements of the Ras-cAMP-PKA pathway, reduced reactive oxygen species (ROS) accumulation and improved yeast growth at low pH (Lastauskienė and Čitavičius 2008; Leadsham and Gourlay 2010; Salas-Navarrete et al. 2022). Taking these facts into consideration, it was suggested that diminishing glucose repression is an adaptive response of yeasts towards higher acetic acid tolerance (Guaragnella and Bettiga 2021). Remarkably, mutations in *RAS2*, *IRA1* (encoding inhibitory regulator of the RAS-cAMP), *IRA2*, *CYR1* (for cyclic AMP requirement), and *BCY1* (for bypass of cyclic-AMP requirement), were found in strains tolerant to elevated temperature or acidic pH (Lastauskienė and Čitavičius 2008; Parts et al. 2011; Salas-Navarrete et al. 2022).

Glucose and nitrogen sensing pathways regulate vacuolar acidification, which helps cytosolic neutralization and prevents mitochondrial dysfunction (Bishop and Guarente 2007; Broach 2012; Hughes and Gottschling 2012). Vacuolar acidification is conducted by the vacuolar membrane ATPase (V-ATPase) which pumps cytosolic H^+^ into the vacuole lumen, making it an ally of PMA1 towards regulating cytosolic pH. Meanwhile, *S. cerevisiae* mutants in genes for the V-ATPase subunits *VMA1*, *VMA2*, and *VMA3*, as well as the assembler *VPH2* failed to acidify the vacuole and achieved cytosolic pH homeostasis (Martínez-Muñoz and Kane 2008; Preston et al. 1992). Vacuolar acidification is involved in vacuole-mitochondria signaling, protein metabolism and amino acids turnover (Hughes and Gottschling 2012). Signaling between mitochondria and vacuole also affected Fe/Cu metabolism and amino acids transport, which were determinant for acetic acid tolerance (Hu et al. 2019; Li and Kaplan 2004). Therefore, genes involved with iron uptake and reduction, like for the ferrous transporter (*FET3*), ferric reductases (*FRE1* and *FRE3*), and facilitators of iron transport (*FIT2* and *FIT3)*, as well as with the efflux of amino acids from vacuole (*ATG22*), and vacuolar protein sorting (*VPS1*, *VPS8*, and *VPS29*), were detected as key elements for acetic acid tolerance (Fletcher et al. 2016; Hu et al. 2019; Mira et al. 2010).

Diffusion of acetic acid into the cytosol may require its interaction with the cellular plasma membrane. Hence, it was suggested that the composition of the plasma membrane is determinant for acetic acid tolerance (Guaragnella and Bettiga 2021). In this regard, some genes involved in the biosynthesis of ergosterol (*ERG3*, *ERG4*, *ERG13*, and *ERG24*), dihydroceramides (*YDC1*), phytoceramides (*YPC1*), and phytosphingosine (*SUR2)* were found to be of importance in the integrity of the cellular membrane and, therefore, towards weak-acid tolerance (Fletcher et al. 2016; Mira et al. 2010). Additionally, since no significant negative effects were observed in the integrity of cell membrane in the presence of acetic acid, as was with the presence of other weak acids (Ullah et al. 2012), it was suggested that the over expression acetate transporters increase the tolerance to high concentrations of acetic acid (Mira et al. 2010; Ullah et al. 2012). In this context, multidrug transports were suggested to play an important role in yeasts tolerant to acetic acid, such as: *AQR1* (encoding acids quinidine resistance), *TPO2* (encoding transporter of polyamines), *TPO3*, *JEN1* (encoding monocarboxylate/proton symporter), and *PDR12* (for pleiotropic drug resistance) (Casal et al. 1999; Mira et al. 2010).

It is known that changes in transcriptional profiles of several genes associated with protein synthesis, such as folding, degradation, and transport, are also involved in yeasts’ response towards elevated temperature and high acetic acid concentrations (Caspeta et al. 2016; Gasch et al. 2000; Lee et al. 2015; Mira et al. 2010). For example, genome expression patterns in *S. cerevisiae* subjected to heat-shock changed in a set of around 772–900 genes (Caspeta et al. 2016; Gasch et al. 2000). Additionally, when using YPD media, the presence of elevated concentrations of acetic acid (0.3 M) and pH 5.8, and mild concentrations of acetic acid (0.15 M) and pH 3.0, it was observed that the number of genes which experienced expression changes were respectively between 227 and 722, (Dong et al. 2017; Lee et al. 2015; Li and Yuan 2010). Furthermore, promoter analysis and sequence characterization suggested a direct implication of transcription factors (TFs) MSN2 (multicopy suppressor of SNF1 mutation, a TF for general stress responses), MSN4 and YAP1 (yeast activator-protein 1 required for oxidative stress tolerance) as the major transcription factors modulating the heat-shock stress response (Gasch et al. 2000). MSN2, MSN4, and SKN7 (suppressor of kre null, a complementary TF for heat shock response) appeared to regulate 25% of approximately 600 genes required to exert acetic acid tolerance (Mira et al. 2010; Schüller et al. 2004). Interestingly, weak acid tolerance was unaffected in *MSN2* and *MSN4* mutants but, conversely, this trait was negatively affected in the case of *WAR1* mutants (Schüller et al. 2004).

In previous studies, we used the adaptive laboratory evolution (ALE) approach to select yeast strains tolerant to elevated temperature (TTY23) (Caspeta et al. 2019), low pH and acetic acid (AT22) , and acetic acid and elevated temperature (TAT12) (Salas-Navarrete et al. 2022). Additionally, targeted transcriptomic analysis of TTY23 genes associated with metabolism allowed fermentation engineering to improve ethanol production (Caspeta et al. 2019). Meanwhile, to improve thermoacidic tolerance in the parental strain S288C by reverse engineering, the whole genome sequencing and transcriptomic analysis of TAT12 had to be carried out (Salas-Navarrete et al. 2022). Also, the growth in nonpermissive pH and temperature profiles for the S288C strain suggested that this displaced its thermoacidic environment to lower pH values and higher temperatures. In this study, we evaluated the extension of thermoacidic profiles of growth in the three evolved strains. Therefore, these were cultivated under both optimal (ancestral) and thermoacidic cultivation conditions. The aim of these evaluations was to determine whether the extension of their thermoacidic profiles was associated to a growth trade-off in optimal conditions. Furthermore, the genome sequence of TTY23, AT22, and S288C strains was completed and their transcriptional responses at optimal growth conditions assessed. Also, we evaluated vacuolar fragmentation under optimal conditions and analyzed whether transcriptional changes in evolved strains were associated with such a typical characteristic of a thermoacidic yeast stress response. Therefore, in this report, it is shown that evolved yeast strains displaced their thermoacidic niches, which correlates to changes in genome structure and transcriptional profiles.

## Materials and methods

### *S. cerevisiae strains* , culture media, and cultivations

In this study, the wild-type (WT) strain of *S. cerevisiae* S288C was used as the reference strain in transcriptomic and genomic analyses (*MATα SUC2 gal2 mal2 mel flo1 flo8-1 hap1 ho bio1 bio6*) (Mortimer and Johnston 1986). This was also the ancestral strain for the selection of the strain TTY23, which was chosen from ALE experiments at 39.5 °C (Caspeta et al. 2019). Additionally, S288C was also used in ALE experiments at low pH (3.0 to 4.0) and elevated acetic acid concentrations (3.0–12.0 g/L) to select the AT22 strain (Salas-Navarrete et al. 2022). Strain TTY23 was the progenitor of strain TAT12, which was obtained by ALE experiments in the same conditions used to generate AT22. Therefore, when comparing AT22 to AT12 and TTY23, this strain was more tolerant to low pH, acetic acid, and elevated temperature (Salas-Navarrete et al. 2022).

All yeast strains were cultivated in a defined mineral media based on DELFT medium composition (Verduyn et al. 1992). Major components of this medium were per liter: 20 g glucose, 5 g (NH_4_)_2_SO_4_, 3 g K_2_PO_4_, and 0.5 g MgSO_4_. This was supplemented with 1 mL of a trace element solution and 1 mL of a vitamin solution. The composition of a 1000× trace element solution was per liter: ethylenediaminetetraacetic acid (sodium salt), 15.0 g; ZnSO_4_·7H_2_O, 4.5 g; MnCl_2_·2H_2_O, 0.84 g; CoCl_2_·6H_2_O, 0.3 g; CuSO_4_·5H_2_O, 0.3 g; Na_2_MoO_4_·2H_2_O, 0.4 g; CaCl_2_·2H_2_O, 4.5 g; FeSO_4_·7H_2_O, 3.0 g; H_3_BO_3_, 1.0 g; and KI, 0.10 g. The composition of a 1000× vitamin solution was per liter: biotin, 0.05 g; *p*-aminobenzoic acid, 0.2 g; nicotinic acid, 1 g; calcium pantothenate, 1 g; pyridoxine hydrochloride, 1 g; thiamine hydrochloride, 1 g; and myoinositol, 25 g. Concentrated solutions of glucose, major salts, and acetic acid were prepared separately and mixed into the medium to achieve the required concentrations of acetic acid. The medium pH was adjusted as needed with a 5 M solution of HCl. Undefined rich medium was used for cultivations in dish plates. Its composition per liter was 1% yeast extract, 2% peptone, and 2% glucose (YPD media).

Cultivations were performed in triplicate under a batch regime using 125-mL flasks, which were filled with 50 mL of defined media, inoculated at 0.1–0.2 units of absorbance at 600 nm (A600), and shaken at 200 revolutions per minute (rpm) in a shaker (G76, New Brunswick Scientific, Edison, NJ, USA). The temperature was controlled at the required value +/− 0.05. All the strains were grown for 15–20 generations before performing the main cultivation experiments to evaluate the specific rates of growth (m), glucose consumption, and ethanol production. The ability to survive and reach a steady state of growth was reported as final A600 (A600_f_) during 7–8 days of serial passages (dilutions), each initiated daily at 0.2 or 0.4 A600 (A600_i_). Namely, it was evaluated if stressed cells either declined growth daily to null (A600_f_ decreased daily until no difference was observed between this and A600_i_) or decreased it transitorily before reaching a balanced growth (A600_f_ decreased transitorily before going to a stable value higher than A600_i_). A stable growth (A600_f_) occurred if A600_f_ did not change more than 5% of an $\overline{A{600}_f}$ from at least 3 days $\left(\left|A{600}_f-\overline{A{600}_f}\right|<0.05\ast \overline{A{600}_f}\right)$.

### Analytic methods

Samples from cultivations were taken every hour. The biomass was measured indirectly as A600. This was converted to grams of cell dry weight (g_CDW_) by a factor of 0.34 g_CDW_/A600. Samples were centrifuged, and the supernatant was stored at −20 °C for further analysis of glucose, ethanol, and glycerol. Metabolites in the supernatant were quantified by high-performance liquid chromatography, HPLC (Waters, Millipore, Milford, MA). The HPLC was equipped with an ion exclusion column HPX-87H (BioRad, Hercules, CA, USA). The mobile phase consisted of a 5 mM H_2_SO_4_ solution flowed at 0.5 mL/min. The column was operated at 50 °C and the compounds were detected with a differential refractive-index and ultraviolet detectors (Waters, Millipore, Milford, MA) and then quantified with a calibration curve made with pure HPLC-grade standards.

### Analysis of gene expression

As we chose to evaluate adaptive responses caused by changes in the genomic structure of evolved strains, it was decided to perform the transcriptomic analyses of yeast strains at optimal ancestral conditions (Caspeta et al. 2016, 2019; Salas-Navarrete et al. 2022). Therefore, the total RNA was extracted from cell populations of S288C, TTY23, AT22, and TAT22 strains cultivated in minimal media at 30 °C, pH 5.2, and without the presence of acetic acid (namely, the optimal ancestral conditions). Samples were taken at the mid-log phase of growth (Supplemental Fig. S1). The total RNA from all strains was extracted and purified with the YeaStar RNA kit (Zymo Research, Irvine, USA). The quality and amount of mRNA in samples were assessed before sending them to the DNA Microarrays Unit of the Institute of Cellular Physiology, at the Universidad Nacional Autónoma de Mexico. cDNA was synthesized using mRNA as template, followed by cDNA staining and hybridization in DNA microarrays. A partial analysis of gene expression changes was also performed at the DNA Microarrays Unit. The genArise package was used to analyze genome-wide gene expression as a swap experimental approach (Gomez-Mayen et al. 2019). The raw data from microarrays was deposited in the GEO DataSets with accession numbers GSE226362 and GPL33201 in the NCBI. Transcriptional profiles of evolved strains and the parental S288C were compared. Differentially expressed genes were selected by calculating an intensity­dependent Z­score as *z*_*i*_ = (*R*_*i*_ − mean(*R*))/(*sd*(*R*)), where z_i_ is the z­score for each element gene, *R*_i_ is the log­ratio for each element gene, and sd(*R*) is the standard deviation of the log­ratio. Genes with |z­score| > 2 standard deviations were considered significantly expressed genes (Gomez-Mayen et al. 2019).

### Whole-genome sequencing and analysis

Whole-genome sequencing of the strains S288C, TTY23, AT22, and TAT12 was conducted. A 2 × 75 bp paired-end sequencing was performed using the next generation sequencing platform MiSeq Illumina® technology (Illumina, San Diego, CA, USA), as described by Salas-Navarrete et al. (2022). In brief, this task was performed at the Unit of Massive Sequencing and Bioinformatics of the Institute of Biotechnology of the Universidad Nacional Autónoma de México. The initial quality check of the sequenced reads was achieved with the FastQC package, version 0.11.9 (bioinformatics.babraham.ac.uk/projects/fastqc/). In addition, the TrimGalore package version 0.6.5 (github.com/FelixKrueger/TrimGalore) was used for trimming the adaptor sequences with default parameters for the pair-end sequencing data of all samples. Over 90% of the reads were guaranteed with a high-quality Q34 (*Q* = 34) score (inferred base call accuracy > 99.96%). This means that one could expect 4 errors in 10,000 base-calls according to $Q=-10\ast {\mathit{\log}}_{10}\left(\frac{\textrm{errors}}{\textrm{base}-\textrm{calls}}\right)$ (Ewing and Green 1998). When sequencing, a quality score of Q30 virtually ensures that all the reads will be perfect, with no errors. This is why Q30 is considered a benchmark for quality in next-generation sequencing platforms, as the one used in this study (Ewing and Green 1998; Ravi et al. 2018). More than three million mappable reads were obtained, providing an average mapped sequence coverage of over 80×. The reads were aligned with the reference genome of the S288C strain using the MosaikAligner package (github com/wanpinglee/MOSAIK) (Lee et al. 2014). Variant calling was conducted with freebayes (github.com/freebayes/ freebayes), and the obtained data was filtered by a quality score > 54 by using the Pandas module in python (pandas. pydata.org/). The data obtained from these analyses was fed into the YeastMine of the Yeastgenome database to search for features from genomic regions. The raw data from sequencing can be accessed at the BioProject with number PRJNA939183 in the NCBI.

### Vacuole and mitochondria visualization

The shape of vacuole and mitochondria was analyzed by staining. To do so, yeast cells were resuspended in 10 mM HEPES-5% glucose buffer at pH 7.4 and stained with CellTracker™ Blue CMAC dye at 100 mM (ThermoFisher Scientific, Waltham, Massachusetts, USA). Cells were incubated for 30 min at room temperature. To observe the mitochondria, cells were resuspended in 50 mM sodium citrate −2% glucose at pH 5 while being incubated with the MitoTracker (ThermoFisher Scientific, Waltham, Massachusetts, USA) at 500 nM. This was performed 30 min before their visualization through confocal fluorescence microscopy. To observe the vacuole, a violet diode laser at 405 nm was employed alongside an inverted multiphotonic confocal Olympus FV1000 microscope equipped with a 60× oil immersion objective (Olympus, Tokyo, Japan). The wavelengths employed for mitochondria were 543 nm (excitation) and 630/60 nm (emission).

## Results

The thermotolerant TTY23 strain was selected from one of the populations found in a previous work, in which three clonal populations of the *S. cerevisiae* strain S288C were evolved at 39.5 ± 0.2 °C for 1 year to generate over 1200 generations (Caspeta et al. 2019). Next, three clonal populations of the strains TTY23 and S288C were separately evolved at concentrations of acetic acid between 3 and 12 g/L, alongside acidic pH between 3 and 4, and at a temperature of 30 °C. From these experiments, the acetic acid and low pH tolerant strain AT22 was derived from S288C, and the strain TAT12, which is also tolerant to these conditions and elevated temperatures, was derived from the TTY23 which in turn was isolated after ~900 generations (Salas-Navarrete et al. 2022). Hence, the TAT12 strain accumulated around two thousand generations over two rounds of mutations. In this work, the physiological responses of strains S288C, TTY23, AT22, and TAT12 to cultivation under optimal conditions (30 °C and pH 5.5 without acetic acid), as well as in challenging thermoacidic conditions, were evaluated. In addition, the whole genome sequencing and transcriptional profiles of these strains were also conducted to assess the implication of gene modifications in transcriptional changes and adaptive responses to harmful thermoacidic environments.

### Physiological responses of evolved strains to heat, acetic acid, and acidic pH

**Fig. 1:**
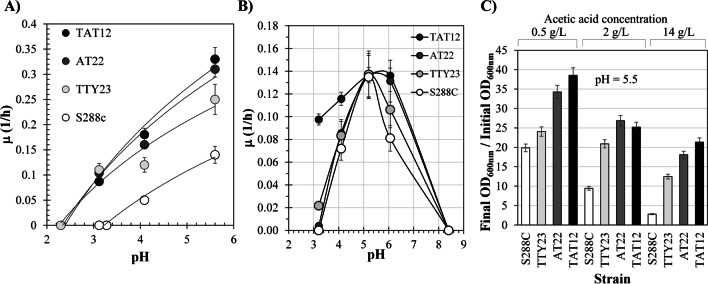
Effect of acidic pH, acetic acid, and supraoptimal temperature of 39 °C on the growth rate and biomass accumulation of parental S288C yeast strain and evolved strains TTY23, AT22, and TAT12. **A** Specific growth rate (m) of cells cultivated on acidic pH and 39 °C. **B** Effect of 2 g/L of acetic acid on the m of yeast cells growing at different pH values. **C** Biomass accumulation after 24 h of yeast cultivations with minimal media containing 0.5 g/L, 2 g/L, and 14 g/L of acetic acid, at a pH of 5.5, and temperature of 39 °C. The error bars represent the standard deviation calculated with three replicas from each elemental experiment

All evolved strains displaced their thermoacidic profile. The strains TTY23, AT22, and TAT12 were cultivated in acidic pH values between 2 and 5.5, at 39 °C to study their tolerance to acidic pH and elevated temperature (Fig. 1). The evolved strains grew faster than the parental strain S288C when these were cultivated at 39 °C and pH 5.5 (Fig. 1A). Under these conditions, the AT22 strain showed higher specific growth rates than TTY23. This result suggests that evolution under high acetic acid concentrations and acidic pH allowed the selection of thermotolerant derivates. Interestingly, the TTY23 strain ceased to grow at pH 2.3, which was also the case for the AT22 and TAT12 strains, but this was not the case for the parental S288C, which ceased to grow at pH 3.3. These results suggest a correlation between cellular responses to acid and elevated temperature evolutions.

The thermoacidic profile of strains was also evaluated with 2 g/L of acetic acid at 39 °C. As can be seen in the Fig. 1B, the TAT12 strain thrived at pH 3, whereas the AT22 reduced its m drastically, and the TTY23 and S288C strains did not grow. The TAT12 m decreased two-thirds fold when compared to the m observed when there was no acetic acid in the media (Fig. 1A). Furthermore, the m also decreased rapidly with acetic acid, as the pH changed from 4 to 3. The evolved strains accumulated more biomass after 24 h when cultivated at 30 °C, 14 g/L of acetic acid and pH 5.5 (Fig. 1C). In these conditions, the TAT12 strain accumulated 7.4-, 1.76-, and 1.22-times more biomass than the S288C, TTY23, and AT22 strains, respectively.

In optimal (ancestral) culture conditions, there was not a significant reduction of the specific growth rate of evolved strains AT22 and TAT12 compared with S288C. Namely, these strains did not show a growth disadvantage under optimal conditions in order to improve their performance in harmful thermoacidic conditions (trade-off). The evolved strains and their ancestor S288C were cultivated in optimal ancestral conditions, namely at 30 °C, pH 5.5, and without acetic acid. Growth kinetic and stoichiometric parameters are shown in Table 1 and Supplemental Fig. S2. These results show that there were not significant differences in growth parameters among the S288C, AT22, and TAT12 strains. The TTY23 strain showed a slightly lower growth rate, along with reduced specific rates of glucose consumption and ethanol production. However, glycerol production rate in this strain was slightly higher than in the S288C, AT22, and TAT12 strains. These results suggest that only the thermotolerant TTY23 strain showed a trade-off in growth parameters under ancestral optimal conditions. This decrease in growth was also observed in thermotolerant strains evolved from the strain CEN- PK113-7D (Caspeta and Nielsen 2015).

**Table 1:** Specific rates (*μ* and *q*) and yields (*Y*) calculated for the growth of S288C and evolved strains TTY23, AT22, and TAT12 cultivated in minimal medium, pH 5.5 and 30 °C, and without acetic acid

Strain	*μ* (1/h)	Specific rates (mmol/g_DCW_/h)			Yields (g/g)		
		*q*_Glc_	*q*_EtOH_	*q*_Gly_	*Y*_X/Glc_	*Y*_EtOH/Glc_	*Y*_Gly/Glc_
S288c	0.33 ± 0.001	14.73 ± 0.41	36.5 ± 1.2	0.85 ± 0.09	0.12 ± 0.02	0.37 ± 0.05	0.08 ± 0.02
TTY23	0.29 ± 0.001	14.18 ± 1.14	28.7 ± 1.53	1.15 ± 0.11	0.12 ± 0.03	0.33 ± 0.03	0.07 ± 0.01
AT22	0.30 ± 0.03	15.34 ± 0.63	32.4 ± 1.89	0.88 ± 0.23	0.11 ± 0.01	0.36 ± 0.02	0.07 ± 0.01
TAT12	0.32 ± 0.01	14.75 ± 1.75	35.5 ± 1.04	0.92 ± 0.12	0.14 ± 0.03	0.37 ± 0.03	0.07 ± 0.02

Abbreviations: *X* biomass, *DCW* dry cell weight, *Glc* glucose, *EtOH* ethanol, *Gly* glycerol

### Analysis of the genome sequences of evolved yeast strains

Whole genome sequence and analysis were conducted for all strains, including the S288C. The complete list of potential mutations without filtering can be found in the Supplemental Table S1. Compared to the reference genome sequence reported in yeastgenome.org, only two mutations were found in the strain S288C (Goffeau et al. 1996). A total of 381 mutations were found in strain AT22, which was evolved for 825 generations in acidic conditions at 30 °C (Table 2). The highest number of mutations was observed in the strain TAT12 (1097 mutations), which was subjected to one round of evolution at elevated temperature and another under acidic conditions, hence accumulating around 2000 generations. Strain TTY23, which was evolved by around 1200 generations at elevated temperature, showed 601 potential mutations. After filtering single nucleotide variations (SNVs) with a quality score of Q34 (*P* = 4 × 10^−4^), a total of 244, 194, and 488 potential mutations were respectively found in the open reading frames over all the chromosomes in the TTY23, AT22, and TAT12 strains. Since TAT12 was evolved from TTY23, it was expected that these strains would share several mutated genes; however, only 50 mutated genes coincided in these strains.

**Table 2:** Description of mutations found in the parental and evolved strains evaluated in this study

Strain	Total	SNV	DEL	INS	Complex	ORF
S288C	2	1	-	-	-	2
TTY23	601	487	83	27	-	367
AT22	381	343	27	3	2	271
TAT12	1097	957	90	30	7	861

Abbreviations are as follows: *SNV* single nucleotide variation, *DEL* deletion, *INS* insertion, *ORF* open reading frame

Following the analysis of mutated genes based on gene orthologs (GO), it was found that the response to chemical (GO:0042221), transcription by RNA polymerase II (GO:0006366), transmembrane transport (GO:0055085), and ion transport (GO:0006811) were the most numerous biological functions, as they represented nearly 40% of the potentially mutated genes (Supplemental Table S2). Out of the 261 possible mutated genes in all the evolved strains found in these four GO-terms, 68 were selected for further analysis (Table 3), as these included two key cellular processes: 22 genes that regulate nutrient availability and stress responses (carbon, nitrogen, and ions), and 44 genes that regulate transmembrane transport (chemicals, ions, protons, and unfolded-proteins). Out of these 68 genes, 38 were exclusive of the TAT12 strain. Remarkably, 8 genes which encode elements of the RAS-cAMP-PKA and SNF1-GAL83 signaling pathways were targeted in the evolution of this strain (*RAS2*, *GPA2*, *ASC1*, *IRA2*, *REG1*, *CAT8*, *HSF1*, and *MIG2*). The potentially mutated genes involved in transcription by RNA polymerase II included: *HFI1*, *HSF1*, *MSN4*, *RIM15*, *RTG3*, *SKN7*, *SUM1*, and *WAR1* (Supplemental Table S2).

**Table 3:** Identification of single nucleotide variations in the evolved strains. The description corresponds to the standard name from the yeastgenome.org. The presence of a specific single nucleotide variation (SNV) in evolved strains is indicated with an “X”

Standard name	Description	TTY23	AT22	TAT12
**Regulation of nutrient availability**				
*ASC1*	Absence of growth suppressor of CYP1			X
*BCK1*	Bypass of C kinase	X		
*CAT8*	Catabolite repression	X	X	
*CUP2*	Copper-binding transcription factor			X
*CYC8*	Cytochrome C	X		
*GAL3*	Galactose metabolism	X		
*GPA1*	G-protein alpha subunit		X	
*GPA2*	G-protein alpha subunit			X
*INO80*	Inositol requiring			X
*IRA2*	Inhibitory regulator of the RAS-cAMP pathway			X
*MIG2*	Multicopy inhibitor of GAL gene expression			X
*OAF3*	Oleate activated transcription factor		X	
*PHO4*	Phosphate metabolism			X
*PSR2*	Plasma membrane sodium response			X
*PUT3*	Proline utilization		X	
*RAS2*	Homologous to RAS proto-oncogene			X
*REG1*	Resistance to glucose repression	X		
*SNF2*	Sucrose nonfermenting			X
*SNF5*	Sucrose nonfermenting	X		
*STE3*	Sterile (receptor for a factor pheromone)			X
*SWI3*	Switching deficient	X		
*TOR1*	Target of rapamycin	X		
**Transporters**				
*AGP2*	High-affinity glutamine permease			X
*ANT1*	Adenine nucleotide transporter			X
*AQY3*	Aquaporin from yeast			X
*ASK10*	Activator of SKN7 (reg. of glycerol channel)			X
*ATO2*	Ammonia (ammonium) transport outward		X	
*BAP3*	Branched-chain amino acid permease			X
*CCH1*	Calcium channel homolog			X
*ERS1*	ERD suppressor			X
*FPS1*	FPD1 suppressor (efflux of glycerol)	X		
*FRE1*	Ferric reductase			X
*FRE2*	Ferric reductase			X
*GLK1*	Glucokinase			X
*HXT14*	Hexose transporter	X		X
*HXT3*	Hexose transporter			X
*HXT8*	Hexose transporter		X	
*JEN1*	Monocarboxylate/proton symporter			X
*KHA1*	K/H ion antiporter			X
*MEP1*	Ammonium permease			X
*MTM1*	Mn trafficking factor for mitochondrial SOD2	X		
*PCA1*	P-type cation-transporting ATPase			X
*PDR10*	Pleiotropic drug resistance			X
*PEX1*	Peroxin			X
*PEX10*	Peroxin		X	
*PEX15*	Peroxin	X		
*PEX2*	Peroxin			X
*PHO84*	Phosphate metabolism		X	
*PHO90*	Phosphate metabolism			X
*PHO91*	Phosphate metabolism		X	
*PMR1*	Plasma membrane ATPase related			X
*PXA1*	Peroxisomal ABC-transporter			
*SEC61*	Secretory	X		
*SEC62*	Secretory			X
*SEC63*	Secretory			X
*SIA1*	Suppressor of eIF5A (activates PMA1)			X
*SIT1*	Siderophore iron transport	X		
*SSA3*	Stress-seventy subfamily A			X
*TAT1*	Tyrosine and tryptophan amino acid transporter	X		
*VCX1*	Vacuolar H^+^/Ca^2+^ exchanger		X	
*VMA2*	Vacuolar membrane ATPase			X
*VMR1*	Vacuolar multidrug resistance	X		
*VPS27*	Vacuolar protein sorting	X		
*VSB1*	Vacuolar storage of basic amino acids			
*YME1*	Yeast mitochondrial escape			X
*YVC1*	Yeast vacuolar conductance			X
**Transcription factors**				
*HFI1*	Histone H2A functional interactor			X
*HSF1*	Heat shock factor 1			X
*MSN4*	Multicopy suppressor of SNF1 mutation			X
*RIM15*	Regulator of IME2			X
*RTG3*	Retrograde regulation			X
*SKN7*	Suppressor of Kre Null			X
*SUM1*	Suppressor of Mar1-1			X
*WAR1*	Weak acid resistance			X

Cellular transport related to intracellular regulation of pH, nutrient availability, and unfolded protein response was also targeted by gene mutations during evolution (Table 3). Genes related to the transport of carbohydrates (*GLK1*, *HXT3*, *HXT8*, and *HXT14*), ammonium and amino acids (*AGP2*, *ATO2*, *BAP3*, *TAT1*, and *VSB1*), metal ions (*CCH1*, *FRE1*, *FRE2*, *MTM1*, *PCA1*, *PHO84*, *PHO90*, *PHO91*, and *SIT1*), protons (*KHA1*, *VCX1*, *VMA2*, and *YVC1*), glycerol (*AQY3*, *ASK10*, *ERS1*, and *FPS1*), chemicals (*PDR10*, *PXA1*, and *VMR1*), and unfolded-proteins (*PEX1*, *PEX2*, *PEX10*, *PEX15*, *SEC61*, *SEC62*, *SEC63*, *SSA3*, *VPS27*, and *YME1*) were detected among the evolved strains.

### Transcriptional profile of evolved yeast strains

**Fig. 2:**
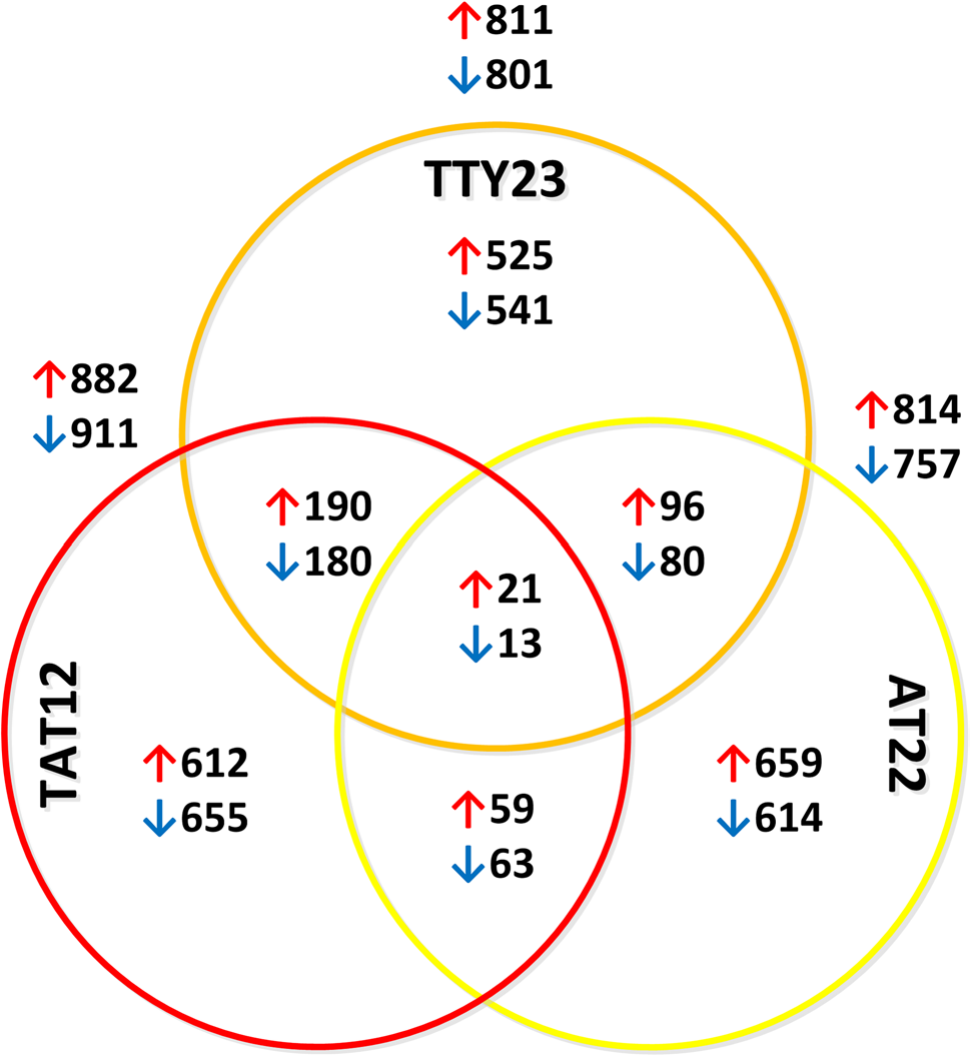
Venn diagram of differentially expressed genes (DEGs) identified in the evolved strains TTY23, AT22, and TAT12. Red and blue arrows indicate over expression and down expression relative to S288C, respectively. The analysis was carried on total RNA samples extracted from cells sampled from cultivations in minimal defined medium at a temperature of 30 °C and pH 5.5

Samples for transcriptomics analyses of S288C and evolved strains TT23, AT22, and TAT12 were taken in the middle exponential phase of cultivations carried out in minimal Delft media with 20 g/L glucose at 30 °C and pH 5.3 (Supplemental Fig. S1). The transcriptional profiles of evolved strains were compared with transcriptional profiles of parental strain S288C. The Venn diagram shown in Fig. 2 and transcriptomic analysis found in Supplemental Tables S3 and S4 show the associations of differentially expressed genes (DEGs) between the evolved strains compared with the parental strain S288C. Over 1500 genes changed their expression in each of the tolerant strains, with the TAT12 strain having the biggest change with almost 1700 genes. The strains evolved under elevated temperature (TTY23 and TAT12) shared 370 DEGs, while those evolved under acidic condition (AT22 and TAT12) shared 122 DEGs. Strains TTY23 and AT22, which only shared the common ancestral strain S288C, shared 176 DEGs. Interestingly, DEGs individually observed in each evolved strain accounted for bigger numbers, 1066, 1273, and 1267 in TTY23, AT22, and TAT12, respectively. Surprisingly, despite these three evolved strains showed higher tolerance to acid and elevated temperature, they only shared 44 DEGs.

The analysis of DEGs found in all evolved strains revealed several potential functions relevant to thermo-acidic tolerance: the plasma membrane P2-type H^+^-ATPase, *PMA1*, and the enolase II (*ENO2*), which catalyzes the conversion of 2-phosphoglycerate to phosphoenolpyruvate during glycolysis and the reverse reaction during gluconeogenesis; the P-loop ATPase, *OLA1*, and the elongation factor two, *EFT2*, which regulate translational elongation (Chen et al. 2015; Justice et al. 1998); the altered inheritance rate of mitochondria gene *AIM2* involved in mitochondrial biogenesis, function, and organization; the DNA helicase *YRF1-5* involved in telomere maintenance (Yamada et al. 1998); the serine hydroxymethyl transferase *SHM2* which converts serine to glycine plus methylenetetrahydrofolate involved in generating precursors for purine, pyrimidine, amino acids, and lipid biosynthesis (McNeil et al. 1994); the methionine adenosyl transferase *SAM1* involved in the synthesis of *S*-adenosylmethionine, *S*-(5′-adenosyl)-L-methionine used in the methylation of proteins, RNAs and lipids, as well as in the biosynthesis of biotin and polyamides (Thomas and Surdin-Kerjan 1991); and the general transcriptional co-repressor *CYC8*, the ubiquinol-cytochrome C oxidoreductase *QCR9*, and the transcriptional activator of genes involved in glycolysis *GCR1* (Willis et al. 2003), were all down expressed.

### The TAT12 and TT23 strains overexpressed key genes of protein synthesis, folding, rescue, and refolding

**Fig. 3:**
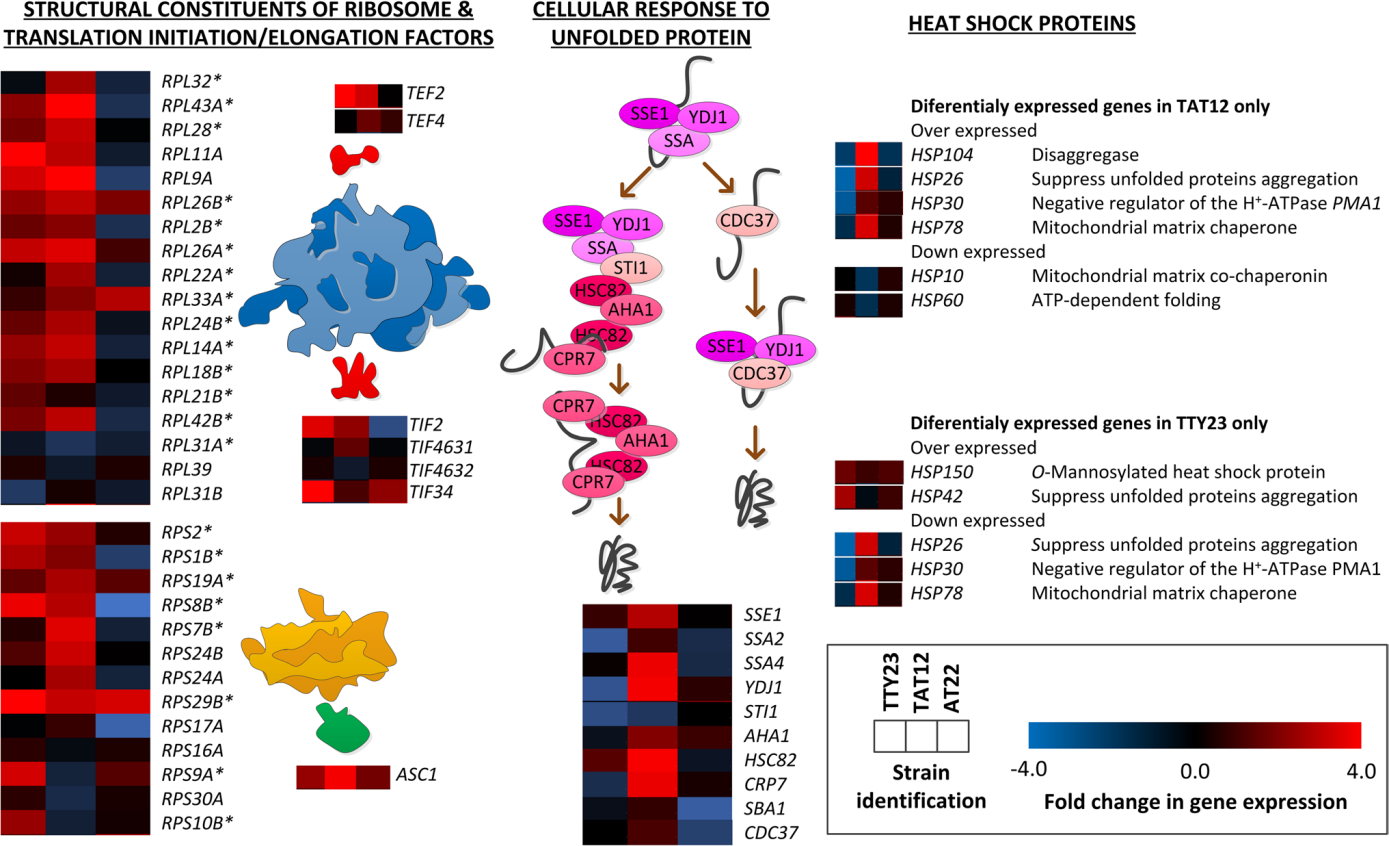
Changes in the expression of genes encoding key proteins of the translation and unfolded protein response in the yeast strains TTY23 (left), TAT12 (middle), and AT22 (right) relative to the S288C strain. Various genes encoding large and small subunits of the ribosome increased their RNA accumulation in strains evolved under high temperature (TTY23 and TAT12). Most of the chaperones involved in the response to unfolded proteins also increased their RNA accumulation in the TAT12 strain

Analysis of DEGs detected in individual strains revealed that TTY23 and TAT12, which evolved at elevated temperature, overregulated 42% of genes involved in cytoplasmic translation (Supplemental Table S2). A summary of key genes associated with the large (60s) and small (40s) subunits of ribosomes is shown in Fig. 3. Remarkably, most of the key genes encoding for protein folding and refolding elements were overexpressed in TAT12. The majority of them are under the control of the heat-sock transcription factor I (*HSF1*), which has a mutation in the 3’UTR region (Salas-Navarrete et al. 2022). This group of genes included the ATPase and chaperon components of the stress-seventy subfamily 70–HSP70 (*SSA1*, *SSA2*, *SSA3*, and *SSA4*) and components of the heat-shock protein HSP90 chaperone complex (*SSE1*, *STI1*, *HSC82*, and *HSP82*), which are all involved in protein folding and rescue from denaturation (Verghese et al. 2012). Also, the heat-shock protein HSP40 (*YDJ1*) which regulates the activity of HSP70 and HSP90, *AHA1* which activates the ATPase HSP90, the *HSP104* that cooperates with *YDJ1* and *SSA1* to refold and reactivate aggregated proteins, and *HSP26* that suppresses unfolded protein aggregation were overexpressed (Verghese et al. 2012). Interestingly, HSP30, which is a negative regulator of H^+^-ATPase PMA1 and that is induced by elevated temperature and weak organic acid (Piper et al. 1997), was downregulated in the strain TTY23, and upregulated in TAT12. Finally, the CPR7 that binds HSP82 and contributes to its activity was also upregulated in TAT12.

**Fig. 4:**
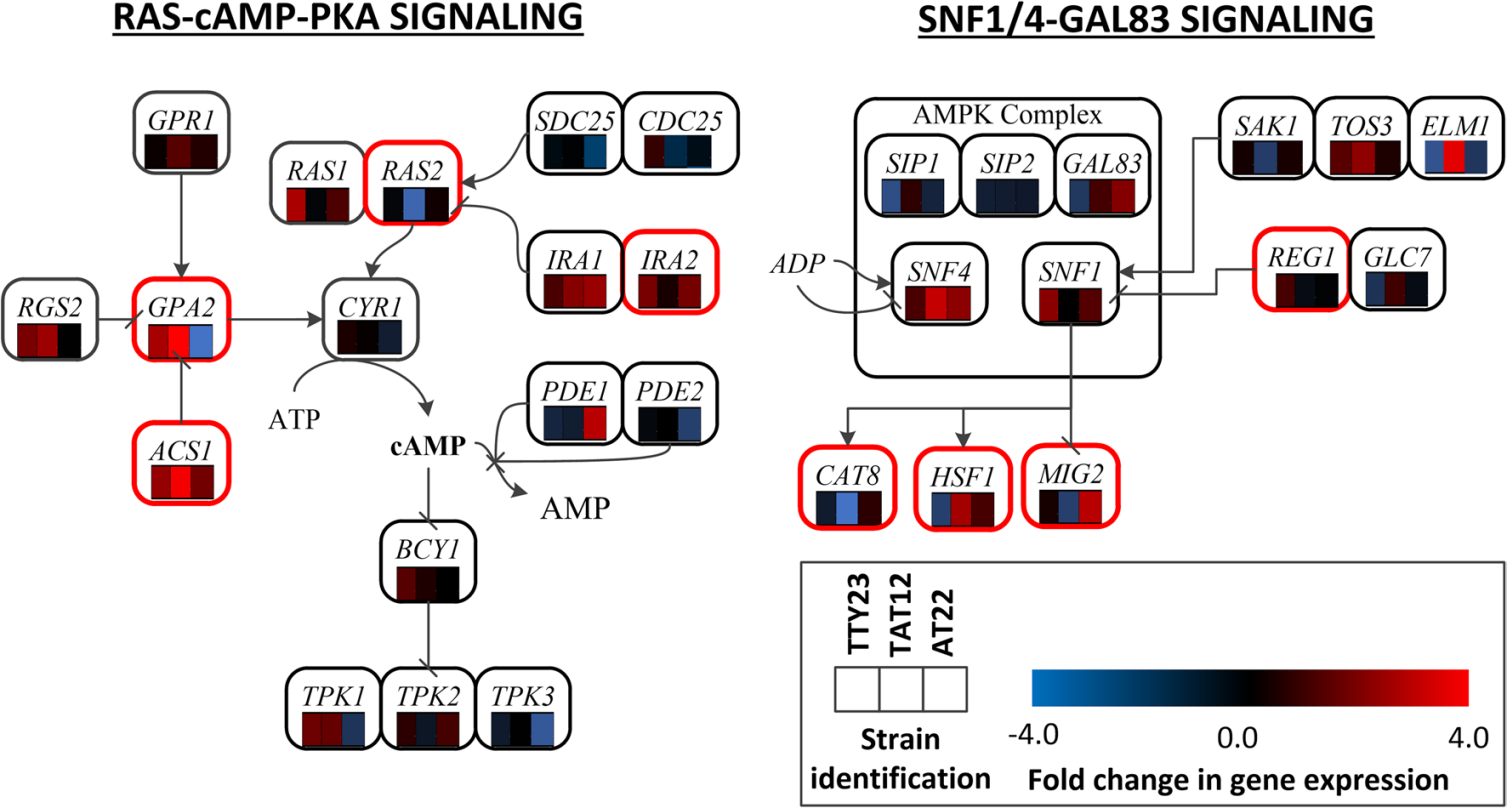
Differential expression of genes encoding for the elements of the glucose signaling pathways RAS-cAMP-PKA signaling and SNF1/4-GAL83 signaling. The red boxes identify the genes with single nucleotide variations in the strain TAT12

Parallel to the increased accumulation of transcripts of genes involved in translation and folding, the genes associated with the signaling pathways related to nutrient availability were also differentially expressed (Fig. 4). Whereas the gene encoding the adenylate cyclase *CYR1* did not change its transcription, the negative regulators ACS1, IRA1, and IRA2 were upregulated in TAT12. The GTP-binding protein RAS2 was only downregulated in the TAT12 strain. Conversely, key genes involved in the activation of the SNF1/4-GAL83 signaling pathway were overexpressed, for example: the AMP-activated S/T protein kinase (*SNF1*), the activating gamma subunit (*SNF4*), and the protein kinases TOS3 and ELM1, which phosphorylate and activate SNF1 (García-Salcedo et al. 2014).

**Fig. 5:**
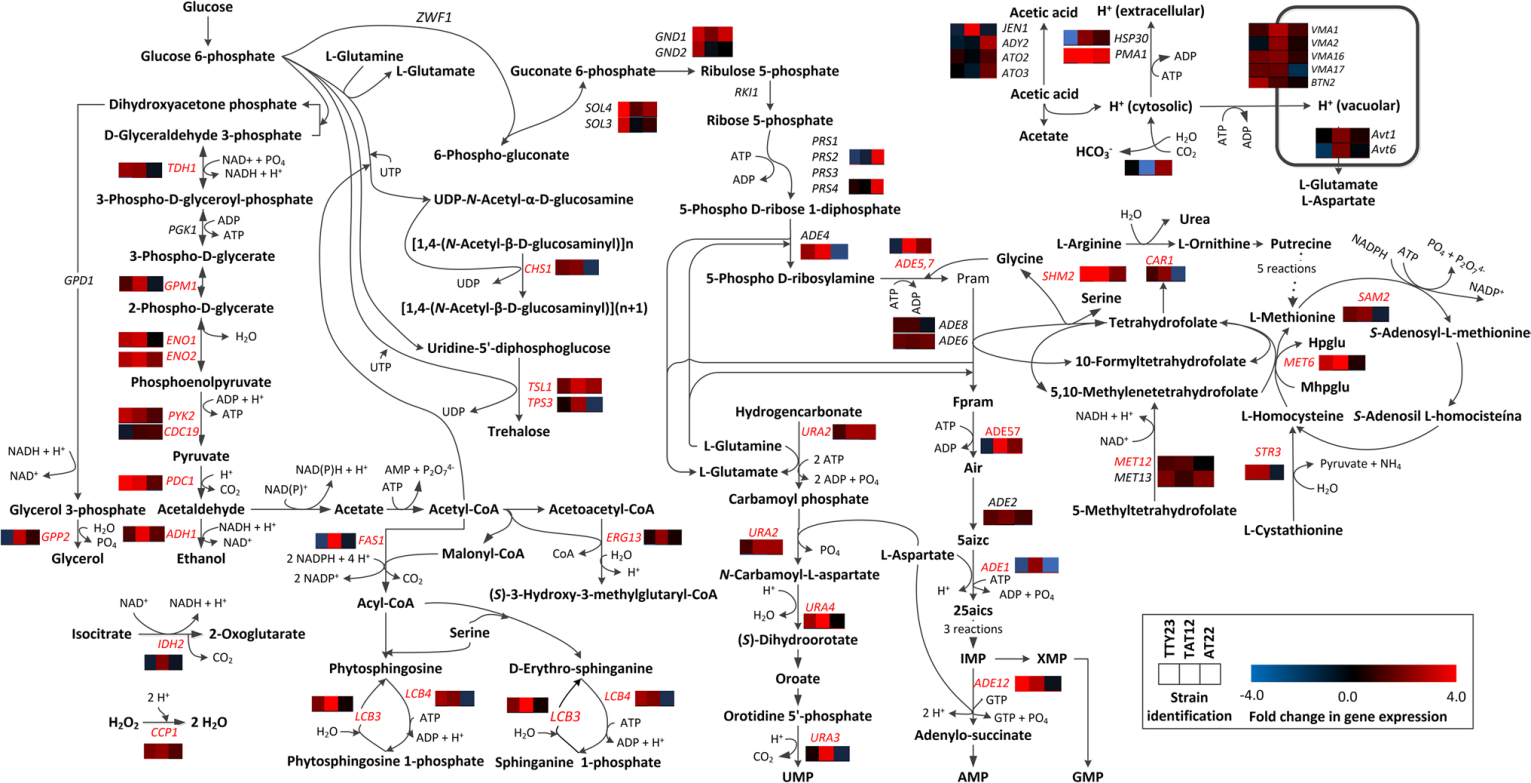
An abstract of changes in expression of important genes associated with metabolic functions related with glycolysis, fermentation, trehalose synthesis, de novo synthesis of nucleotides monophosphate, one carbon cycle, and transport of acetic acid and H^+^. Genes transcribed by the TFs, SKN7, and HSF1 are indicated in red. Abbreviations: 5-phospho-b-D-ribosylamine (Pram), 2-(formamido)-*N*1-(5-phospho-D-ribosyl)acetamidine (Fram), 5-amino-1-(5-phospho-D-ribosyl)imidazole (Air), 5-amino-1-(5-phospho-D-ribosyl)imidazole-4-carboxylate (5aizc), (*S*)-2-[5-amino-1-(5-phospho-D-ribosyl)imidazole-4-carboxamido]succinate (25aics), tetrahydropteroyltri-L-glutamate (Hpglu), and 5-methyltetrahydropteroyltri-L-glutamate (Mhpglu)

Most transcriptional changes in metabolic genes occurred in the thermoacidic tolerant strain TAT12. About 44%, 28%, and 62% of genes encoding enzymes of the glycolytic pathway were overexpressed in the strains TTY23, AT22, and TAT12, respectively (Fig. 5 and Supplemental Table S3). The overexpression of the hexokinase genes *HXK1* and *GLK1* was only found in TAT12, whereas *TDH1/2/3*, *GPM1*, *ENO1/2*, *PYK2*, and *CDC19*, which are associated with the energy-releasing phase in glycolysis were overexpressed in TAT12 and TTY23, and with lesser increments than in TAT12. Expression of most of these genes is under the control of the TFs SKN7 and HSF1 (Supplemental Table S4), which was mutated in TAT12. Gene encoding the major pyruvate carboxylase *PDC1* increased its expression in TTY23 and TAT12, while the acetaldehyde dehydrogenase *ADH1* and the DL-glycerol-3-phosphate phosphatase *GPP2*, which are involved in the reactions for ethanol and glycerol synthesis, were only overexpressed in the TAT12 strain. Genes encoding the enzymes for the oxidative branch of the pentose-phosphate pathway, *SOL3/4*, and *GND1/2* increased their expression in TTY23 and AT22, while two 5-phospho-D-ribose-1-diphosphate synthetases (*PRS2/4*) were the only one overexpressed in AT22.

Genes encoding enzymes of key reactions for the biosynthesis of trehalose (*TSL1* and *TPS3*), chitin (*CHS1*), Acyl-CoA (*FAS1*), mevalonate precursor HMG-CoA (*ERG13*), long-chain sphingolipids (*LCB3* and *LCB4*) were upregulated in TAT12 and in TTY23. Likewise, the expression of essential genes involved in the novo biosynthesis of purines and pyrimidines only increased in TAT12 (*ADE4*, *ADE57*, *URA2*, *ADE1*, *URA4*, *URA3*, and *ADE12*). The biosynthesis of purines and pyrimidines is associated with the synthesis of serine and glycine, as well as with folate and one-carbon metabolism. Some genes of these metabolic processes were overexpressed in TTY23 and TAT12 (i.e., *SHM2*, *MET12*, *MET13*, *MET6*, *SAM2*, and *STR3*).

The regulation of intracellular pH in *S. cerevisiae* in the presence of acetic acid broadly depends on transporters of the monocarboxylic acid and H^+^ (de Kok et al. 2012). Expression of the monocarboxylate/H^+^ symporter JEN1 increased in strain TAT12, meanwhile the acetate transporter ADY2 and its paralog ATO2, which also transports ammonium, were only overexpressed in AT22. *JEN1* was also mutated in TAT12 (Table 3). The plasma membrane H^+^-ATPase PMA1 was upregulated in all evolved strains, whereas the transcription of its negative regulator, *HSP30* increased in TAT12 and AT22, but decreased in TTY23. Additionally, mediated transcription of this H^+^ pump occurs via RAP1 and GCR1 in the presence of glucose (Rao et al. 1993). *GCR1* was downregulated in all evolved strains and *RAP1* expression augmented in TTY23. The carbonic anhydrase NCE103 that converts CO_2_ and H_2_O to HCO_3_^−^ and H^+^ decreased its mRNA levels in TAT12, while it increased in AT22. All these signals of internal acidification of TAT12 also corresponded with an increase in the transcripts of key genes involved in the formation of the vacuolar H^+^-ATPase (*VMA1*, *VMA2*, *CMA15*, and *VMA17*). The L-glutamate and L-aspartate vacuolar transporters AVT1 and AVT6 were overexpressed in TAT12. Proper functionality of V-ATPase is required for vacuolar acidification, which is the key for cellular protein turnover (Kane 2006).

Minor vacuolar fragmentation under optimal ancestral growth conditions occurred in all evolved strains. The V-ATPase is a major regulator of cellular homeostasis (Kane 2006). In conjunction with PMA1, the V-ATPase controls vacuolar acidification and cytosolic pH, which are essential for vacuolar and mitochondrial functions, such as degradation of improperly folded proteins accumulated during thermal and acidic stresses (Carmelo et al. 1996; Kane 2006). Overexpression of key genes associated with vacuole, like *VMA1/2* and *AVT1*, can compensate for this organelle functions when unproper acidification occurred (Hughes and Gottschling 2012). A decrement in V-ATPase activity triggers a downregulation of *PMA1* alongside cytosolic acidification, vacuolar alkalinization, reduction of mitochondrial transmembrane potential and vacuolar and mitochondrial fragmentation (Aufschnaiter and Büttner 2019). Whether transcriptional changes and mutations connected vacuolar fragmentation in evolved strains was another aim of this study.

**Fig. 6:**
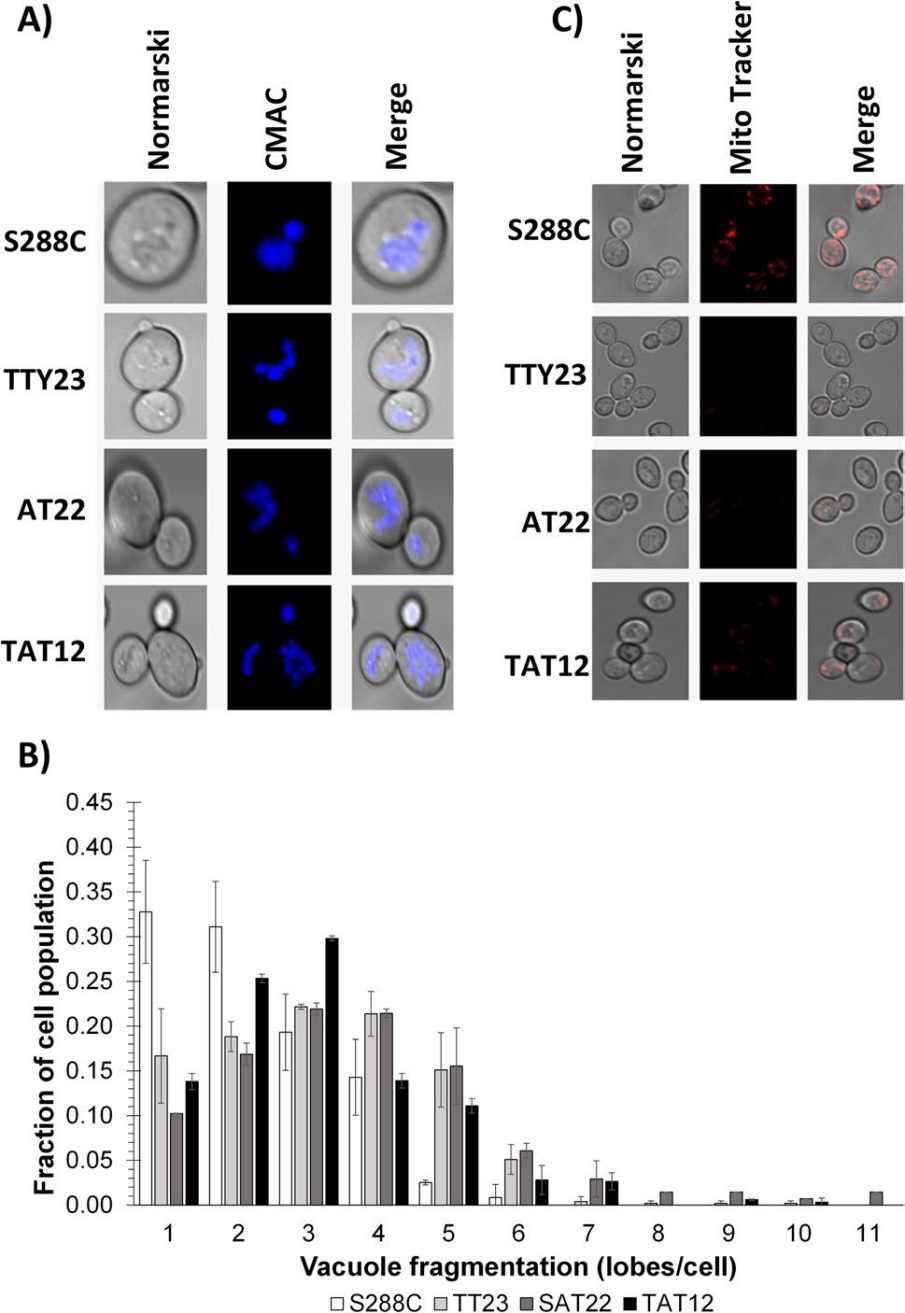
Vacuolar fragmentation in yeast cells sampled during the middle exponential phase of cultivations in minimal media at 30 °C, pH 5.5, and 30 °C

In the Figs. 6 A and B, it can be seen that the distribution of vacuolar lobes in the S288C strain population of 257 cells is skewed right with a pondered mean of 2.13 ± 0.17 lobes per cell and a maximum of 4 lobes (*P* value < 0.24). The strains TTY23, AT22, and TAT12 show a symmetrical distribution histogram with means of 3.4 ± 0.19, 3.54 ± 0.25, and 3.44 ± 0.13, and respective maximum lobes per cells of 6 (*P* value < 0.004), 7 (*P* value < 0.015), and 7 (*P* value < 0.006). The number of individuals analyzed in these populations totaled 311, 373, and 332 cells for TTY23, AT22, and TAT12, respectively. These results show a slight vacuolar fragmentation in evolved strains compared to their parental S288C. Additionally, a larger mitochondrial network across cells of S288C compared to the evolved strains was also observed (Fig. 6D).

## Discussion

### Evolved yeast strains expanded their thermoacidic profile

The strains evolved in acetic acid and low pH (AT22), supraoptimal temperature of 39 °C (TTY23), and both conditions (TAT12) thrived at a pH ~2.8 and at 39 °C. At this temperature, the parental S288C strain did not grow at pH below 3.3 (Fig. 1A). Similarly, the thermal death of a wine yeast strain IGC 4072 was observed at a pH ~3.3 and at ~38–39 °C (Pinto et al. 1989). Under 30 °C and anaerobic conditions, the strain CBS 8066 stopped growing at a pH below ~2.5 (Taherzadeh et al. 1997). In the presence of 1 g/L acetic acid, the thermal death of strain IGC 4072 was observed at ~37 °C and pH 3.3 (Pinto et al. 1989), 2 °C lower compared to the case without acetic acid. Increasing the concentration of acetic acid to 4 g/L and keeping the pH at 3.3 required to lower the temperature below 30 °C to allow yeast growth (Pinto et al. 1989; Taherzadeh et al. 1997). Likewise, in this work we observed that strain S288C, as well as the thermotolerant TTY23 did not thrive in 2 g/L acetic acid (32.7 mM undissociated acid), pH 3.3 and 39 °C, which were conditions that the AT22 and TAT12 strains were able to tolerate. Yet, the specific growth rate of TAT12 was five times higher than that observed in AT22 (Fig. 1B). Interestingly, when a similar concentration of undissociated acid was introduced (36.6 mM–14 g/L acetic acid at pH 5.5) all strains, including S288C, grew at 30°C. However, TAT12 accumulated 8.4, 1.76, and 1.22 times more biomass than the S288C, TTY23, and AT22 strains, respectively (Fig. 1C). TAT12 was also the only strain which grew with 3 g/L of acetic acid (48 mM undissociated acid), pH 3.3, and 37 °C (Salas-Navarrete et al. 2022). Under similar conditions, strain IGC 4072 required temperatures below 36 °C to grow (Pinto et al. 1989). Overall, the results from this and other studies suggest that the evolved strains AT22, TTY23, and TAT12 expanded their thermo-acidic profiles.

The whole genome sequence of evolved strains revealed mutations in key genes associated with TFs and nutrient availability

With a quality score Q34, we found that strains AT12, TTY23, and TAT12 respectively evolved over ~925, ~1097, and ~1912 generations and accumulated 194, 244, and 488 potential mutations. Overall, these mutations targeted 844 different genes. On average, these evolved strains accumulated ~2 SNVs per 100 generations. In a long-term laboratory evolution approach achieved in rich medium and optimal temperature (30 °C), Fisher et al. (2018) observed 2320 mutated genes among 64 sequenced strains evolved from W303. On average, these strains accumulated 91 mutations (maximum of 138 and minimum of 41) over ~4000 generations, which is equivalent to ~0.035–0.23 SNVs per 100 generations (Fisher et al. 2018). Comparatively, this result is one magnitude lower than the mutational ratio calculated in this work. However, we decided to keep Q34 (*P* = 0.0004) in our analysis since the sequence analysis of the S288C strain used here was comparable to that reported in yeastgenome.org, as only two mutations were detected. Our results suggest that in harmful conditions yeast may accumulate mutations faster than over optimal or mild conditions. This has been also noted in other studies with short-term evolution, although lower rates than the observed in this work have also been reported (Dettman et al. 2012).

To gain further understanding on molecular responses of evolved strains, we analyzed mutated TFs on their association with DEGs using YeTFaSCo (De Boer and Hughes 2012) (Supplemental Table S4). In the TTY23 strain, 55.95% and 32.34% of upregulated genes were potentially associated to the TFs SFP1 (split finger protein) and YRR1 (yeast reveromycin-A resistant) –*P* = 1.6 × 10^−6^ and *P* = 2.0 × 10^−3^. These regulate transcription of genes related to ribosomal and nutrients availability, as well as genes involved in multidrug resistance and oxidative stress response (Marion et al. 2004; Le Crom et al. 2002). In AT22, the TF BAS1 potentially regulates the expression of 47.16% of upregulated genes (*P* = 1 × 10^−3^), which participate in the histidine, purine, and pyrimidine biosynthetic pathways, and in the control of cellular ATP levels (Daignan-Fornier and Fink 1992; Takaine et al. 2022). In TAT12, the TFs HFI1 (histone H2A functional interactor), HSF1, and SKN7 potentially regulate the overexpression of 25.61%, 18.1%, and 21.9% genes (*P* = 2 × 10^−5^, 1 × 10^−5^, and 3 × 10^−5^), respectively. HFI1 is an adaptor protein for the histone acetyltransferase-coactivator complex that participates in global regulation of gene expression, including the heat-shock genes (Horiuchi et al. 1997). SKN7 recognizes 21 DNA motifs that regulate transcription from RNA polymerase II promoter in response to oxidative, heat-shock, and osmotic stress (Raitt et al. 2000). These motifs are different to those recognized by HSF1 (Raitt et al. 2000). Therefore, SKN7 is required for a proper cellular stress response, since mutants in this gene do not display a regular activation of heat-shock proteins HSP12, HSP26, and HSP104 through the motif GAAnnTTC (Raitt et al. 2000). Moreover, these HSPs were overexpressed in TAT12 (Fig. 3). Remarkably, the overexpression of genes associated to key metabolic functions to keep energy pools in TAT12 was associated to SKN7 (Fig. 5).

### Transcriptomics showed that evolved strains progressed to regulate internal pH and nutrient and energy availability

Regarding DEGs observed in all evolved strains compared with the parental S288C, all cultivated under optimal conditions, it is not surprising that *PMA1* was upregulated, as its ectopic expression enhanced yeasts tolerant to weak-acids, ROS and ethanol (Lee et al. 2017). *ENO2* overexpression is interesting, as it is most abundant in yeasts, and it encoded protein catalyzes the first common step of glycolysis and gluconeogenesis (Ho et al. 2018). OLA1 (obg-like ATPase) and EF2 (initiation factor 2) regulate translation rate upon acute heat-shock stress (Dannenmaier et al. 2021). EF2 forms a ternary complex with GTP and the initiator methionyl-tRNA, mediating ribosomal recruitment (Justice et al. 1998). Moreover, limiting the formation of this complex is a key mechanism for triggering the integrated stress response (ISR) as, in human cells, it decreases global translation in response to stress (Chen et al. 2015). OLA1 is a member of an ancient family of GTPases that regulates EF2 by hydrolyzing its attached GTP. This mechanism saves cellular energy and reprograms translation to maintain ATP pools which help to deal with stress responses (Chen et al. 2015). Regulation of ATP levels may also target genes involved in one-carbon metabolism and de-novo synthesis of nucleotides, like SHM2 and SAM1. Interestingly, these genes are under the control of the TF BAS1 (overexpressed/mutated in AT22), which regulates cellular ATP levels to prevent protein aggregation (Takaine et al. 2022)*.* These levels can be controlled via de novo synthesis of adenine nucleotides. Remarkably, various genes from this pathway were overexpressed in TAT12 and potentially regulated by SKN7 and HSF1 (Fig. 5).

Internal pH, which affects all cellular functions, is mainly regulated through cell metabolism and proton export and translocation into cellular compartments (Orij et al. 2011). Taking this into consideration, the role of glucose signaling genes in intracellular pH regulation upon a glucose pulse was analyzed by Isom et al. (2018). They observed that mutants in genes *GRP1*, *GPA2*, *RGT2*, *SNF3*, *RGT1*, *ELM1*, *REG1*, *MIG1*, and *HXK2*, involved in SNF1/4 and RAS/cAMP/PKA sugar-signaling pathways, helped yeast to recover neutral pH after a pulse of glucose, whereas mutations in *RAS2*, *SNF1*, *TPK2*, and *PDE2* increased cytosolic acidification (Isom et al. 2018). The overactivation of these pathways also stimulated acetic acid resistance (Fernandes et al. 2005). However, experimental evidence showed that RAS2 had a negative influence on cell viability in the presence of acetic acid (Lastauskienė and Čitavičius 2008). Also, the overactivation of the RAS/cAMP/PKA pathway can cause retrograde response and cell death (Leadsham and Gourlay 2010). Furthermore, a decreased expression of *RAS2*, *TPK1*, and *TPK3* has been shown to reduce ROS accumulation and cellular inactivation (Dimster-Denk et al. 1995; Leadsham and Gourlay 2010). Interestingly, mutations in key gene-coding regulators of the Ras-cAMP-PKA pathway, including *IRA1*, *IRA2*, *CYR1*, and *BCY1*, were found in a strain tolerant to supraoptimal temperatures (Parts et al. 2011). In this study we found that TAT12 was the only strain with mutations in genes associated with glucose-signaling pathways, including *RAS2*, *GPA2*, *ACS1*, *REG1*, and *IRA2* (Fig. 4). This strain also increased the expression of negative regulators (*GPA2*, *ACS1*, and *IRA2*) and decreased the expression of *RAS2*. To identify whether these genes were mutated in individuals from populations evolved at 39 °C (thermotolerant population) or acetic acid and low pH (acid-tolerant populations) will require a broader scope sequencing project than the one used here.

Weak-acid stress is well known as a potent fungistatic agent; therefore, the role of potential transporters in resistance has been evaluated (Verduyn et al. 1992). In search of potential targets that counteract weak-acid toxicity, Schüller et al. (2004) found that lacking *MSN2*/*MSN4* did not affect tolerance, as did the absence of *WAR1*. Remarkably, the regulon of this TF is composed by very few elements (De Boer and Hughes 2012; Schüller et al. 2004), including the plasma membrane ATP-binding cassette PDR12, which is a multidrug transporter required for weak-acid resistance (Schüller et al. 2004). The *WAR1* gene was potentially mutated in TAT12 whereas *PDR12* transcription only increased in TTY23. However, TAT12 overexpressed *JEN1* (also mutated), and AT22 overexpressed *ADY2* and its paralog *ATO2*, these three genes encode transporters of acetic acid (de Kok et al. 2012).

This work has shown that the yeast strains tolerant to high temperature (TTY23), acetic acid (AT22), and both of these conditions (TAT12) increased their thermoacidic profiles compared to the parental strain S288C. Thus, a compromise between the tolerance improvement and optimal growth under ideal ancestral conditions can be achieved. However, only the TTY23 strain slightly reduced its growth rate at 30 °C and pH 5.5. Transcriptomics and whole genome analysis showed that some adaptive responses of tolerant yeast strains potentially aimed at H+ and acetic acid transport, the regulation of metabolism and stress responses via RAS/cAMP/PKA signaling pathway and de novo synthesis of nucleotides; and on protein synthesis, folding, and rescue from unfolding. This conclusion is supported by mutations in: *HSF1* and *SKN7*, which are involved in protein folding, energy generation, and carbohydrate metabolism; *BAS1*, which participates in the control of ATP levels; *WAR1*, which is involved in the weak acid response; and *ASC1*, *GPA2*, *RAS2*, and *IRA2* which regulate the RAS/cAMP/PKA signaling pathway.

## Supplementary information

ESM 1 (PDF 5967 kb)

## Data Availability

The datasets generated during and/or analyzed during the current study are available from the corresponding author on reasonable request.
