# Supplementary material for: Adaptive responses of yeast strains tolerant to acidic pH, acetate, and supraoptimal temperature
Source: Appl Microbiol Biotechnol. 2023 May 13;107(12):4051–68. doi: 10.1007/s00253-023-12556-7 (PMC10238323; doi:10.1007/s00253-023-12556-7)
Supplement: Supplementary file 1 — (PDF 5967 kb) [file 253_2023_12556_MOESM1_ESM.pdf]

Supplementary materials

Applied Microbiology and Biotechnology

Adaptive responses of yeast strains tolerant to acidic pH, acetate and supraoptimal temperature

Prisciluis Caheri Salas-Navarrete[1], Paul Rosas-Santiago[2], Ramón Suárez-Rodriguez[1], Alfredo Martínez[3], Luis Caspeta[3*]

[1]Centro de Investigación en Biotecnología, Universidad Autónoma del Estado de Morelos, Av. Universidad 1001, Col. Chamilpa, Cuernavaca, 62209 Morelos, México.

[2]Departamento de Biología Molecular de Plantas, Instituto de Biotecnología, Universidad Nacional Autónoma de México, Av. Universidad 2001, Col. Chamilpa, Cuernavaca, 62210 Morelos, México.

[3]Departamento de Ingeniería Celular y Biocatálisis, Instituto de Biotecnología, Universidad Nacional Autónoma de México, Av. Universidad 2001, Col. Chamilpa, Cuernavaca, 62210 Morelos, México.

*Corresponding author. luis.caspeta@ibt.unam.mx. Tel. +52 777 3291 648.

Introduction. In this study, the adaptive responses of the wild-type (WT) strain of *S. cerevisiae* S288C and its thermotolerant TTY23, acid-tolerant AT22, and thermo-acidic-tolerant TAT12 variants, obtained by adaptive laboratory evolution (ALE) experiments, were evaluated in cultivations at optimal (ancestral) conditions (30 °C and pH 5.5, without acetic acid) and thermo-acidic conditions (acidic pH between 2.5 and 5.5, supraoptimal temperature of 39 °C, and concentrations of acetic acid between 0.5 and 14 g/L). Evaluations included judging of changes in gene expression and metabolic responses (consumption of glucose and accumulation of biomass, ethanol, and glycerol) of evolved variants compared with S288C, both assessments in cultivations under optimal conditions. Additionally, it was evaluated the genomic structure, in term of mutations occurred in evolved strains through the ALE experiments. In this supplementary materials section, the reader of the main text of this publication can find additional information which complement data offered in the main text. In the Fig. S1 it can be found the kinetics of biomass accumulation in cultivations of all strains at optimal conditions and the exact point, indicated by an arow, where the samples for transcriptomic analyses were taken. In the Fig. S2, it can be seen the kinetics of glucose consumption and production of biomass, ethanol, and glycerol observed in all strains cultivated in optimal conditions. The results from the whole genome sequence analysis of all strains, showing changes in one single nucleotide or fragments of DNA are presented in the Table S1. In Table S2 there is an analysis of GO-terms of the mayor cellular functions found in the whole genome sequence analysis. The analysis of changes in gene expression of yeast evolved strains compared to the parental strain S288C can be consulted in the Table S3. Finally, results from the analysis of mutated transcription factors and their associated differential expressed genes using YeTFaSCo is shown in the Table S4. For details on the methodologies to assess these evaluations please refer to the main text.

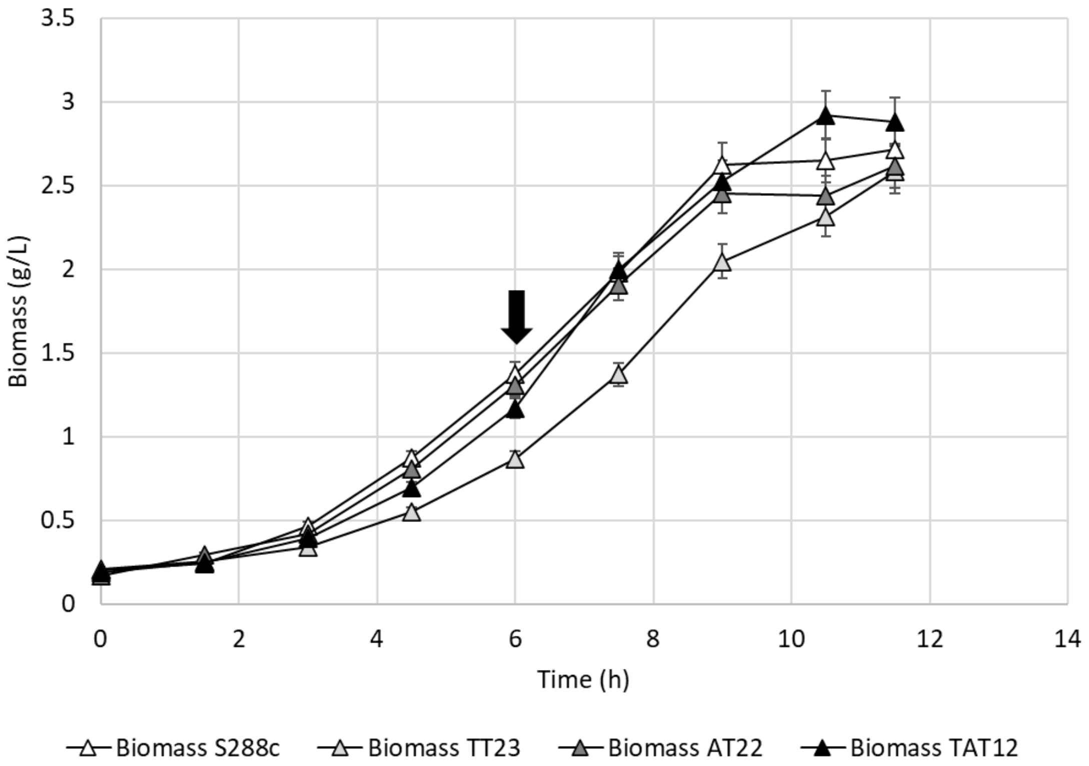

Fig. S1 Kinetics of biomass accumulation in cultivations of strains S288c, TT23, AT22, and TAT12 in minimal Delt media with 20 g/L glucose at 30°C, pH 5.2 and 250 RPMs. The arrow indicates the time point where samples for transcriptomics analyses were taken.

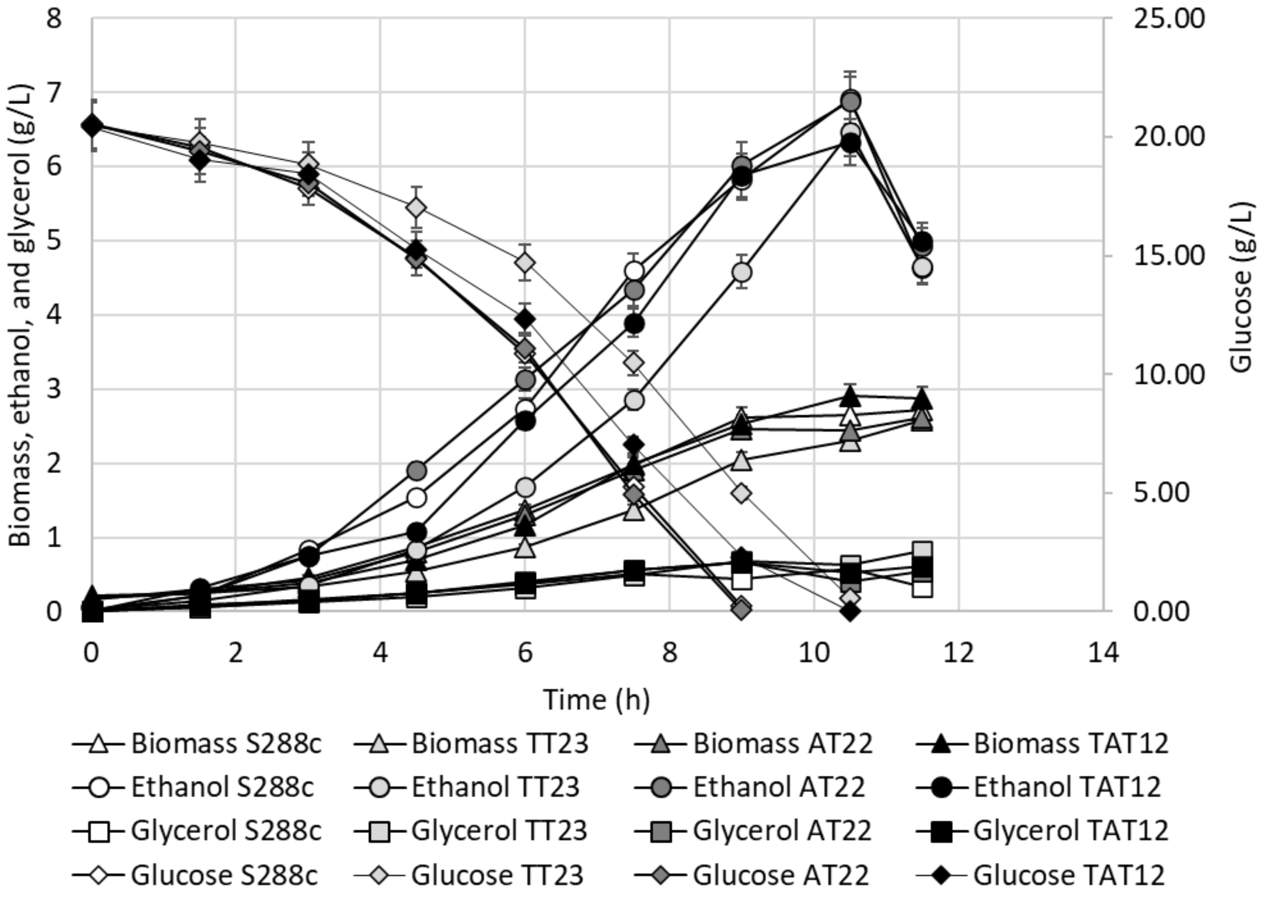

Fig. S2 Kinetics of biomass, glucose, ethanol, and glycerol concentrations during cultivations of strains S288c, TT23, AT22, and TAT12 in minimal Delt media with 20 g/L glucose at 30°C, pH 5.2 and 250 RPMs.

Table S1 Results from the whole genome sequence analysis. POS, position where the mutation is found; REF, reference sequence; ALT, alternate sequence; QUAL, quality score.

TTY23											
**POS**	**REF**	**ALT**	**QUAL**	**TYPE**	**Chromosome**	**DB identifier**	**GeneSysName**	**GeneStdName**	**Start**	**End**	**Protein name**
31250	GAAAAAAT	GAAAAAT	59.3806	TYPE=del	chrI	intergenic					
57423	T	C	59.3806	TYPE=snp	chrI	intergenic					
77302	C	A	59.5363	TYPE=snp	chrI	S000000033	YAL035W	FUN12	76427	79435	Function Unknown Now
185114	A	G	59.5992	TYPE=snp	chrI	S000000076	YAR028W		184892	185596	
15510	G	T	59.9101	TYPE=snp	chrII	S000000201	YBL105C	PKC1	14241	17696	Protein Kinase C
15510	G	T	60.0372	TYPE=snp	chrII	S000000201	YBL105C	PKC1	14241	17696	Protein Kinase C
15518	A	G	59.9101	TYPE=snp	chrII	S000000201	YBL105C	PKC1	14241	17696	Protein Kinase C
37981	A	G	59.7635	TYPE=snp	chrII	S000000195	YBL099W	ATP1	37053	38690	ATP synthase
92035	C	G	60.0372	TYPE=snp	chrII	intergenic					
126275	C	G	59.5363	TYPE=snp	chrII	intergenic					
171345	A	T	59.3806	TYPE=snp	chrII	intergenic					
221008	A	G	59.7861	TYPE=snp	chrII	intergenic					
248502	A	G	59.5363	TYPE=snp	chrII	S000000210	YBR006W	UGA2	247010	248503	Utilization of GAba
291821	ACC	AC	59.3806	TYPE=del	chrII	S000000229	YBR025C	OLA1	290681	291865	Obg-Like ATPase
304636	T	C	59.5363	TYPE=snp	chrII	S000000237	YBR033W	EDS1	301944	304703	Expression Dependent on Slt2
339027	GCC	GC	59.6621	TYPE=del	chrII	S000000256	YBR052C	RFS1	338720	339352	Rad55 (Fifty-five) Suppressor
378077	C	G	59.9101	TYPE=snp	chrII	S000000273	YBR069C	TAT1	376574	378433	Tyrosine and tryptophan Amino acid Transporter
464666	C	T	59.5363	TYPE=snp	chrII	S000000316	YBR112C	CYC8	462870	465770	CYtochrome C
476614	G	T	59.5363	TYPE=snp	chrII	intergenic					
483477	A	T	59.6499	TYPE=snp	chrII	intergenic					
582623	T	A	59.5363	TYPE=snp	chrII	S000000378	YBR174C		582338	582652	
582623	T	A	59.5363	TYPE=snp	chrII	S000000379	YBR175W	SWD3	582408	583355	Set1c, WD40 repeat protein
583130	C	T	59.9101	TYPE=snp	chrII	S000000379	YBR175W	SWD3	582408	583355	Set1c, WD40 repeat protein
618512	C	T	59.7128	TYPE=snp	chrII	S000000402	YBR198C	TAF5	616127	618523	TATA binding protein-Associated Factor
642752	G	T	59.3806	TYPE=snp	chrII	S000000413	YBR209W		642583	642900	

651823	A	T	59.3806	TYPE=snp	chrII	S000000418	YBR214W	SDS24	651415	652998	homolog of S. pombe SDS23
697649	G	T	59.3806	TYPE=snp	chrII	intergenic					
753247	G	T	59.7635	TYPE=snp	chrII	S000000479	YBR275C	RIF1	751356	757106	RAP1-Interacting Factor
755928	T	A	59.3806	TYPE=snp	chrII	S000000479	YBR275C	RIF1	751356	757106	RAP1-Interacting Factor
766989	G	A	59.7635	TYPE=snp	chrII	S000000485	YBR281C	DUG2	764970	767606	Deficient in Utilization of Glutathione
769087	A	G	59.7635	TYPE=snp	chrII	S000000487	YBR283C	SSH1	768943	770415	Sec Sixty-one Homolog
781773	A	G	59.3806	TYPE=snp	chrII	S000000493	YBR289W	SNF5	779667	782384	Sucrose NonFermenting
796924	C	A	59.9101	TYPE=snp	chrII	S000000500	YBR296C	PHO89	796798	798522	PHOsphate metabolism
48589	TAAAAAAAC	TAAAAAAAAC	59.5363	TYPE=ins	chrIII	intergenic					
50124	G	T	59.7635	TYPE=snp	chrIII	S000000548	YCL043C	PDI1	48653	50221	Protein Disulfide Isomerase
67249	A	G	59.3806	TYPE=snp	chrIII	S000000535	YCL030C	HIS4	65934	68333	HIStidine requiring
103256	C	T	59.5363	TYPE=snp	chrIII	S000000517	YCL011C	GBP2	102075	103358	G-strand Binding Protein
124224	C	A	60.0848	TYPE=snp	chrIII	intergenic					
133157	T	A	59.9101	TYPE=snp	chrIII	intergenic					
143131	T	C	60.0372	TYPE=snp	chrIII	intergenic					
143131	T	C	59.9101	TYPE=snp	chrIII	intergenic					
143131	T	C	59.5963	TYPE=snp	chrIII	intergenic					
146394	C	T	59.7635	TYPE=snp	chrIII	S000000610	YCR017C	CWH43	144773	147634	Calcofluor White Hypersensitive
163059	T	C	59.9101	TYPE=snp	chrIII	S000000619	YCR024C-A	PMP1	162945	163067	Plasma Membrane Proteolipid
168487	G	C	60.0372	TYPE=snp	chrIII	intergenic					
191364	A	G	59.9101	TYPE=snp	chrIII	S000000630	YCR034W	ELO2	190592	191635	fatty acid ELOngation
233027	T	A	59.5363	TYPE=snp	chrIII	intergenic					
264185	C	G	59.7635	TYPE=snp	chrIII	S000000683	YCR087W		263976	264491	
264185	C	G	59.7635	TYPE=snp	chrIII	S000007223	YCR087C-A		264006	264467	
282722	C	G	59.2846	TYPE=snp	chrIII	S000000689	YCR093W	CDC39	280117	286443	Cell Division Cycle
283705	G	A	59.9101	TYPE=snp	chrIII	S000000689	YCR093W	CDC39	280117	286443	Cell Division Cycle
114909	G	A	60.0372	TYPE=snp	chrIV	S000002352	YDL193W	NUS1	114672	115799	Nuclear Undecaprenyl pyrophosphate Synthase
123362	C	T	60.0372	TYPE=snp	chrIV	S000002348	YDL189W	RBS1	122216	123589	RNA-Binding Suppressor of PAS kinase
355124	A	G	59.3806	TYPE=snp	chrIV	S000002214	YDL056W	MBP1	352877	355378	MluI-box Binding Protein
384776	T	A	59.4577	TYPE=snp	chrIV	S000002195	YDL037C	BSC1	384601	385587	Bypass of Stop Codon
464312	C	T	59.7635	TYPE=snp	chrIV	S000002416	YDR009W	GAL3	463434	464996	GALactose metabolism
485279	G	A	59.572	TYPE=snp	chrIV	S000002426	YDR019C	GCV1	484163	485365	GlyCine cleaVage

499477	A	C	59.9101	TYPE=snp	chrIV	S000002435	YDR028C	REG1	497835	500879	REsistance to Glucose repression
513064	A	G	59.5363	TYPE=snp	chrIV	intergenic					
520434	G	A	59.7635	TYPE=snp	chrIV	intergenic					
561504	G	A	59.5363	TYPE=snp	chrIV	S000002461	YDR054C	CDC34	561440	562327	Cell Division Cycle
604398	T	A	59.7635	TYPE=snp	chrIV	S000002487	YDR080W	VPS41	604008	606986	Vacuolar Protein Sorting
625241	G	T	59.3806	TYPE=snp	chrIV	S000002497	YDR090C	ILT1	625066	625998	Ionic Liquid Tolerance
673657	T	C	59.3806	TYPE=snp	chrIV	S000002516	YDR109C		673520	675667	
705182	C	T	60.0372	TYPE=snp	chrIV	S000002534	YDR127W	ARO1	704484	709250	AROmatic amino acid requiring
722566	T	A	59.9101	TYPE=snp	chrIV	intergenic					
760993	G	A	60.0372	TYPE=snp	chrIV	S000002557	YDR150W	NUM1	755628	763874	NUclear Migration
817531	TAAAAAAA AAAC	TAAAAAAA AAAAC	60.0637	TYPE=ins	chrIV	intergenic					
862854	TAA	TA	60.0637	TYPE=del	chrIV	S000002614	YDR206W	EBS1	862054	864708	Est1-like Bcy1 Suppressor
885519	T	A	59.7635	TYPE=snp	chrIV	S000002619	YDR211W	GCD6	884727	886865	General Control Derexpressed
909550	GAA	GA	59.6621	TYPE=del	chrIV	intergenic					
914604	T	A	59.3806	TYPE=snp	chrIV	S000002632	YDR224C	HTB1	914317	914712	Histone h Two B
926475	C	T	59.7635	TYPE=snp	chrIV	S000002638	YDR230W		926223	926570	
926475	C	T	59.7635	TYPE=snp	chrIV	S000002639	YDR231C	COX20	926293	926910	Cytochrome c OXidase
987244	G	A	59.7635	TYPE=snp	chrIV	intergenic					
1037210	A	G	59.8449	TYPE=snp	chrIV	S000002696	YDR288W	NSE3	1E+06	1E+06	Non SMC Element
1058296	T	C	59.7635	TYPE=snp	chrIV	S000002706	YDR298C	ATP5	1E+06	1E+06	ATP synthase
1086575	T	A	59.9101	TYPE=snp	chrIV	S000002719	YDR311W	TFB1	1E+06	1E+06	Transcription Factor B
1111569	G	T	59.3806	TYPE=snp	chrIV	S000002730	YDR322W	MRPL35	1E+06	1E+06	Mitochondrial Ribosomal Protein, Large subunit
1126554	G	A	59.9101	TYPE=snp	chrIV	S000002737	YDR329C	PEX3	1E+06	1E+06	PEroXin
1131981	G	T	59.3806	TYPE=snp	chrIV	S000002740	YDR332W	IRC3	1E+06	1E+06	Increased Recombination Centers
1144932	GAAAAAAA AAAT	GAAAAAAA AAAAT	59.9101	TYPE=ins	chrIV	intergenic					
1151431	A	T	59.9101	TYPE=snp	chrIV	intergenic					
1162007	T	A	59.3806	TYPE=snp	chrIV	S000002752	YDR344C		1E+06	1E+06	
1262755	A	G	59.9101	TYPE=snp	chrIV	S000002802	YDR394W	RPT3	1E+06	1E+06	Regulatory Particle Triple-A protein, or Regulatory Particle Triphosphatase
1303748	A	T	59.5363	TYPE=snp	chrIV	S000002827	YDR419W	RAD30	1E+06	1E+06	RADiation sensitive
1307471	T	C	59.9101	TYPE=snp	chrIV	S000002828	YDR420W	HKR1	1E+06	1E+06	Hansenula mrakii Killer toxin Resistant

1314773	C	A	59.3806	TYPE=snp	chrIV	S000002829	YDR421W	ARO80	1E+06	1E+06	AROmatic amino acid requiring
1330816	G	A	59.9101	TYPE=snp	chrIV	intergenic					
1338715	A	T	59.7635	TYPE=snp	chrIV	S000002846	YDR438W	THI74	1E+06	1E+06	THIamine regulon
1339560	A	G	59.3806	TYPE=snp	chrIV	intergenic					
1378512	G	A	59.5363	TYPE=snp	chrIV	S000002865	YDR457W	TOM1	1E+06	1E+06	Temperature dependent Organization in Mitotic nucleus or Trigger Of Mitosis
1433710	A	T	59.7635	TYPE=snp	chrIV	S000002898	YDR490C	PKH1	1E+06	1E+06	Pkb-activating Kinase Homolog
1440202	C	T	59.5363	TYPE=snp	chrIV	S000002903	YDR495C	VPS3	1E+06	1E+06	Vacuolar Protein Sorting
1446246	T	A	59.3806	TYPE=snp	chrIV	S000002906	YDR498C	SEC20	1E+06	1E+06	SECretory
1449979	T	C	59.3806	TYPE=snp	chrIV	S000002907	YDR499W	LCD1	1E+06	1E+06	Lethal, Checkpoint-defective, DNA damage sensitive
1469980	A	G	59.3806	TYPE=snp	chrIV	intergenic					
35102	G	C	59.9101	TYPE=snp	chrIX	intergenic					
46385	C	G	59.3806	TYPE=snp	chrIX	S000001420	YIL158W	AIM20	46201	46815	Altered Inheritance rate of Mitochondria
78383	C	T	59.9101	TYPE=snp	chrIX	S000001406	YIL144W	NDC80	78074	80149	Nuclear Division Cycle
117470	A	T	59.2846	TYPE=snp	chrIX	S000001389	YIL127C	RRT14	117024	117644	Regulator of rDNA Transcription
122082	A	G	59.5363	TYPE=snp	chrIX	intergenic					
122089	GTTTTTTTT TTTTA	GTTTTTTTT TA	59.5363	TYPE=del	chrIX	intergenic					
131627	T	C	59.7635	TYPE=snp	chrIX	S000001384	YIL122W	POG1	130610	131665	Promoter Of Growth
143091	C	T	59.7635	TYPE=snp	chrIX	S000001378	YIL116W	HIS5	142928	144085	HIStidine requiring
152512	T	C	59.3806	TYPE=snp	chrIX	S000001374	YIL112W	HOS4	151595	154846	Hda One Similar
203372	C	G	60.0372	TYPE=snp	chrIX	intergenic					
270356	C	G	59.3806	TYPE=snp	chrIX	S000001308	YIL046W	MET30	268651	270573	METhionine requiring
282467	T	C	59.3806	TYPE=snp	chrIX	S000001300	YIL038C	NOT3	280142	282652	Negative On TATA
306870	C	T	60.0372	TYPE=snp	chrIX	S000001288	YIL026C	IRR1	304477	307929	IRRegular cell behavior
353955	A	T	59.7635	TYPE=snp	chrIX	S000001263	YIL001W		353940	355481	
357927	TGA	TA	59.9101	TYPE=del	chrIX	S000001441	YIR002C	MPH1	357415	360396	Mutator PHenotype
375304	G	A	59.5363	TYPE=snp	chrIX	intergenic					
253	AATT	AT	59.8629	TYPE=del	chrmt	intergenic					
547	TTCTTAATT AAATTAT	TTCTTAATT AAATTATCT	59.9831	TYPE=ins	chrmt	intergenic					

		TAATTAAAT TAT									
678	ATT	ATTT	59.7128	TYPE=ins	chrmt	intergenic					
686	T	A	59.8449	TYPE=snp	chrmt	intergenic					
3491	A	T	59.7635	TYPE=snp	chrmt	intergenic					
5706	TTAT	TT	59.8368	TYPE=del	chrmt	intergenic					
6079	A	T	60.0372	TYPE=snp	chrmt	intergenic					
6499	AATATATAT ATATATATA TT	AATATATAT ATATATATT	59.7635	TYPE=del	chrmt	intergenic					
8206	C	A	59.5363	TYPE=snp	chrmt	intergenic					
8680	T	C	60.0039	TYPE=snp	chrmt	intergenic					
9210	ATTTTTTTT TA	ATTTTTTTT A	59.3806	TYPE=del	chrmt	intergenic					
10090	G	A	59.3806	TYPE=snp	chrmt	intergenic					
14325	G	A	59.3806	TYPE=snp	chrmt	S000007260	Q0045	COX1	13818	26701	Cytochrome c OXidase
14325	G	A	59.3806	TYPE=snp	chrmt	S000007261	Q0050	AI1	13818	16322	
14325	G	A	59.3806	TYPE=snp	chrmt	S000007262	Q0055	AI2	13818	18830	
14325	G	A	59.3806	TYPE=snp	chrmt	S000007263	Q0060	AI3	13818	19996	
14325	G	A	59.3806	TYPE=snp	chrmt	S000007264	Q0065	AI4	13818	21935	
14325	G	A	59.3806	TYPE=snp	chrmt	S000007265	Q0070	AI5_ALPHA	13818	23167	
16927	G	T	59.8368	TYPE=snp	chrmt	S000007260	Q0045	COX1	13818	26701	Cytochrome c OXidase
16927	G	T	59.8368	TYPE=snp	chrmt	S000007262	Q0055	AI2	13818	18830	
16927	G	T	59.8368	TYPE=snp	chrmt	S000007263	Q0060	AI3	13818	19996	
16927	G	T	59.8368	TYPE=snp	chrmt	S000007264	Q0065	AI4	13818	21935	
16927	G	T	59.8368	TYPE=snp	chrmt	S000007265	Q0070	AI5_ALPHA	13818	23167	
19032	T	C	59.3806	TYPE=snp	chrmt	S000007260	Q0045	COX1	13818	26701	Cytochrome c OXidase
19032	T	C	59.3806	TYPE=snp	chrmt	S000007263	Q0060	AI3	13818	19996	
19032	T	C	59.3806	TYPE=snp	chrmt	S000007264	Q0065	AI4	13818	21935	
19032	T	C	59.3806	TYPE=snp	chrmt	S000007265	Q0070	AI5_ALPHA	13818	23167	
19129	T	A	59.6621	TYPE=snp	chrmt	S000007260	Q0045	COX1	13818	26701	Cytochrome c OXidase
19129	T	A	59.6621	TYPE=snp	chrmt	S000007263	Q0060	AI3	13818	19996	
19129	T	A	59.6621	TYPE=snp	chrmt	S000007264	Q0065	AI4	13818	21935	
19129	T	A	59.6621	TYPE=snp	chrmt	S000007265	Q0070	AI5_ALPHA	13818	23167	
20345	GAA	GA	60.0637	TYPE=del	chrmt	S000007260	Q0045	COX1	13818	26701	Cytochrome c OXidase
20345	GAA	GA	60.0637	TYPE=del	chrmt	S000007264	Q0065	AI4	13818	21935	
20345	GAA	GA	60.0637	TYPE=del	chrmt	S000007265	Q0070	AI5_ALPHA	13818	23167	
20345	GAA	GA	59.7635	TYPE=del	chrmt	S000007260	Q0045	COX1	13818	26701	Cytochrome c OXidase
20345	GAA	GA	59.7635	TYPE=del	chrmt	S000007264	Q0065	AI4	13818	21935	

20345	GAA	GA	59.7635	TYPE=del	chrmt	S000007265	Q0070	AI5_ALPHA	13818	23167	
20345	GAA	GA	62.2346	TYPE=del	chrmt	S000007260	Q0045	COX1	13818	26701	Cytochrome c OXidase
20345	GAA	GA	62.2346	TYPE=del	chrmt	S000007264	Q0065	AI4	13818	21935	
20345	GAA	GA	62.2346	TYPE=del	chrmt	S000007265	Q0070	AI5_ALPHA	13818	23167	
20345	GAA	GA	59.3806	TYPE=del	chrmt	S000007260	Q0045	COX1	13818	26701	Cytochrome c OXidase
20345	GAA	GA	59.3806	TYPE=del	chrmt	S000007264	Q0065	AI4	13818	21935	
20345	GAA	GA	59.3806	TYPE=del	chrmt	S000007265	Q0070	AI5_ALPHA	13818	23167	
20345	GAA	GA	59.9101	TYPE=del	chrmt	S000007260	Q0045	COX1	13818	26701	Cytochrome c OXidase
20345	GAA	GA	59.9101	TYPE=del	chrmt	S000007264	Q0065	AI4	13818	21935	
20345	GAA	GA	59.9101	TYPE=del	chrmt	S000007265	Q0070	AI5_ALPHA	13818	23167	
20345	GAA	GA	72.2002	TYPE=del	chrmt	S000007260	Q0045	COX1	13818	26701	Cytochrome c OXidase
20345	GAA	GA	72.2002	TYPE=del	chrmt	S000007264	Q0065	AI4	13818	21935	
20345	GAA	GA	72.2002	TYPE=del	chrmt	S000007265	Q0070	AI5_ALPHA	13818	23167	
20934	T	G	59.5363	TYPE=snp	chrmt	S000007260	Q0045	COX1	13818	26701	Cytochrome c OXidase
20934	T	G	59.5363	TYPE=snp	chrmt	S000007264	Q0065	AI4	13818	21935	
20934	T	G	59.5363	TYPE=snp	chrmt	S000007265	Q0070	AI5_ALPHA	13818	23167	
20934	T	G	59.8449	TYPE=snp	chrmt	S000007260	Q0045	COX1	13818	26701	Cytochrome c OXidase
20934	T	G	59.8449	TYPE=snp	chrmt	S000007264	Q0065	AI4	13818	21935	
20934	T	G	59.8449	TYPE=snp	chrmt	S000007265	Q0070	AI5_ALPHA	13818	23167	
20934	T	G	59.5363	TYPE=snp	chrmt	S000007260	Q0045	COX1	13818	26701	Cytochrome c OXidase
20934	T	G	59.5363	TYPE=snp	chrmt	S000007264	Q0065	AI4	13818	21935	
20934	T	G	59.5363	TYPE=snp	chrmt	S000007265	Q0070	AI5_ALPHA	13818	23167	
22297	C	A	59.8449	TYPE=snp	chrmt	S000007260	Q0045	COX1	13818	26701	Cytochrome c OXidase
22297	C	A	59.8449	TYPE=snp	chrmt	S000007265	Q0070	AI5_ALPHA	13818	23167	
23343	AATATATAT ATATATATT	AATATATAT ATATATATA TT	59.6621	TYPE=ins	chrmt	S000007260	Q0045	COX1	13818	26701	Cytochrome c OXidase
23827	A	G	59.5363	TYPE=snp	chrmt	S000007260	Q0045	COX1	13818	26701	Cytochrome c OXidase
23924	AATATATAT ATATATATA	AATATATAT ATATATA	59.6621	TYPE=del	chrmt	S000007260	Q0045	COX1	13818	26701	Cytochrome c OXidase
26878	TTATATATA TATATATAT AA	TTATATATA TATATATAT ATAA	59.3806	TYPE=ins	chrmt	intergenic					
28130	TAAAAAAA AAT	TAAAAAAA AT	59.9623	TYPE=del	chrmt	intergenic					
30435	TAA	TA	59.6621	TYPE=del	chrmt	intergenic					
30807	TTATATATA TAT	TTATATATA T	59.2447	TYPE=del	chrmt	intergenic					
31906	T	C	60.0039	TYPE=snp	chrmt	intergenic					

32345	TATAATAAT AATAA	TATAATAAT AA	59.5363	TYPE=del	chrmt	intergenic					
33169	A	T	59.6621	TYPE=snp	chrmt	intergenic					
33467	T	C	59.6621	TYPE=snp	chrmt	intergenic					
33467	T	C	59.8449	TYPE=snp	chrmt	intergenic					
33467	T	C	59.5363	TYPE=snp	chrmt	intergenic					
33618	TA	TTCA	59.3806	TYPE=ins	chrmt	intergenic					
33618	TA	TTCA	59.7635	TYPE=ins	chrmt	intergenic					
33629	A	T	59.7635	TYPE=snp	chrmt	intergenic					
37013	GAA	GA	59.8449	TYPE=del	chrmt	S000007270	Q0105	COB	36540	43647	CytochrOme B
37013	GAA	GA	59.8449	TYPE=del	chrmt	S000007271	Q0110	BI2	36540	38579	
37013	GAA	GA	59.8449	TYPE=del	chrmt	S000007272	Q0115	BI3	36540	40265	
37013	GAA	GA	59.8449	TYPE=del	chrmt	S000007273	Q0120	BI4	36540	42251	
38140	T	C	59.8449	TYPE=snp	chrmt	S000007270	Q0105	COB	36540	43647	CytochrOme B
38140	T	C	59.8449	TYPE=snp	chrmt	S000007271	Q0110	BI2	36540	38579	
38140	T	C	59.8449	TYPE=snp	chrmt	S000007272	Q0115	BI3	36540	40265	
38140	T	C	59.8449	TYPE=snp	chrmt	S000007273	Q0120	BI4	36540	42251	
38252	A	G	59.6621	TYPE=snp	chrmt	S000007270	Q0105	COB	36540	43647	CytochrOme B
38252	A	G	59.6621	TYPE=snp	chrmt	S000007271	Q0110	BI2	36540	38579	
38252	A	G	59.6621	TYPE=snp	chrmt	S000007272	Q0115	BI3	36540	40265	
38252	A	G	59.6621	TYPE=snp	chrmt	S000007273	Q0120	BI4	36540	42251	
39517	G	T	59.8449	TYPE=snp	chrmt	S000007270	Q0105	COB	36540	43647	CytochrOme B
39517	G	T	59.8449	TYPE=snp	chrmt	S000007272	Q0115	BI3	36540	40265	
39517	G	T	59.8449	TYPE=snp	chrmt	S000007273	Q0120	BI4	36540	42251	
39517	G	T	59.8449	TYPE=snp	chrmt	S000007270	Q0105	COB	36540	43647	CytochrOme B
39517	G	T	59.8449	TYPE=snp	chrmt	S000007272	Q0115	BI3	36540	40265	
39517	G	T	59.8449	TYPE=snp	chrmt	S000007273	Q0120	BI4	36540	42251	
39517	G	T	60.0637	TYPE=snp	chrmt	S000007270	Q0105	COB	36540	43647	CytochrOme B
39517	G	T	60.0637	TYPE=snp	chrmt	S000007272	Q0115	BI3	36540	40265	
39517	G	T	60.0637	TYPE=snp	chrmt	S000007273	Q0120	BI4	36540	42251	
39517	G	T	59.8449	TYPE=snp	chrmt	S000007270	Q0105	COB	36540	43647	CytochrOme B
39517	G	T	59.8449	TYPE=snp	chrmt	S000007272	Q0115	BI3	36540	40265	
39517	G	T	59.8449	TYPE=snp	chrmt	S000007273	Q0120	BI4	36540	42251	
39517	G	T	59.6621	TYPE=snp	chrmt	S000007270	Q0105	COB	36540	43647	CytochrOme B
39517	G	T	59.6621	TYPE=snp	chrmt	S000007272	Q0115	BI3	36540	40265	
39517	G	T	59.6621	TYPE=snp	chrmt	S000007273	Q0120	BI4	36540	42251	
39517	G	T	59.8449	TYPE=snp	chrmt	S000007270	Q0105	COB	36540	43647	CytochrOme B
39517	G	T	59.8449	TYPE=snp	chrmt	S000007272	Q0115	BI3	36540	40265	
39517	G	T	59.8449	TYPE=snp	chrmt	S000007273	Q0120	BI4	36540	42251	

39517	G	T	59.5363	TYPE=snp	chrmt	S000007270	Q0105	COB	36540	43647	CytochrOme B
39517	G	T	59.5363	TYPE=snp	chrmt	S000007272	Q0115	BI3	36540	40265	
39517	G	T	59.5363	TYPE=snp	chrmt	S000007273	Q0120	BI4	36540	42251	
40901	C	T	59.8449	TYPE=snp	chrmt	S000007270	Q0105	COB	36540	43647	CytochrOme B
40901	C	T	59.8449	TYPE=snp	chrmt	S000007273	Q0120	BI4	36540	42251	
41238	T	C	59.5363	TYPE=snp	chrmt	S000007270	Q0105	COB	36540	43647	CytochrOme B
41238	T	C	59.5363	TYPE=snp	chrmt	S000007273	Q0120	BI4	36540	42251	
42021	T	A	60.0372	TYPE=snp	chrmt	S000007270	Q0105	COB	36540	43647	CytochrOme B
42021	T	A	60.0372	TYPE=snp	chrmt	S000007273	Q0120	BI4	36540	42251	
42947	T	A	59.6621	TYPE=snp	chrmt	S000007270	Q0105	COB	36540	43647	CytochrOme B
43018	T	A	59.9036	TYPE=snp	chrmt	S000007270	Q0105	COB	36540	43647	CytochrOme B
43084	ATTTTTTTTA	ATTTTTTTTTA	59.9036	TYPE=ins	chrmt	S000007270	Q0105	COB	36540	43647	CytochrOme B
43084	ATTTTTTTTA	ATTTTTTTTTA	59.3806	TYPE=ins	chrmt	S000007270	Q0105	COB	36540	43647	CytochrOme B
43084	ATTTTTTTTA	ATTTTTTTTTA	60.0637	TYPE=ins	chrmt	S000007270	Q0105	COB	36540	43647	CytochrOme B
43093	A	T	59.6621	TYPE=snp	chrmt	S000007270	Q0105	COB	36540	43647	CytochrOme B
43425	A	G	59.5363	TYPE=snp	chrmt	S000007270	Q0105	COB	36540	43647	CytochrOme B
44014	CAA	CA	59.5992	TYPE=del	chrmt	intergenic					
45714	T	A	59.6621	TYPE=snp	chrmt	intergenic					
45971	ATTTTTTTTTTTTA	ATTTTTTTTTTTA	60.0637	TYPE=del	chrmt	intergenic					
46201	A	G	59.8449	TYPE=snp	chrmt	intergenic					
48547	TTATATATATATATATATAT	TTATATATATATATATAT	59.9869	TYPE=del	chrmt	intergenic					
48956	A	G	59.3806	TYPE=snp	chrmt	S000007275	Q0140	VAR1	48901	50097	
48963	T	A	59.5363	TYPE=snp	chrmt	S000007275	Q0140	VAR1	48901	50097	
49135	A	T	60.0039	TYPE=snp	chrmt	S000007275	Q0140	VAR1	48901	50097	
49329	A	T	59.7635	TYPE=snp	chrmt	S000007275	Q0140	VAR1	48901	50097	
49500	A	T	59.7635	TYPE=snp	chrmt	S000007275	Q0140	VAR1	48901	50097	
49500	A	T	59.6621	TYPE=snp	chrmt	S000007275	Q0140	VAR1	48901	50097	
49500	A	T	59.5363	TYPE=snp	chrmt	S000007275	Q0140	VAR1	48901	50097	
49500	A	T	59.5363	TYPE=snp	chrmt	S000007275	Q0140	VAR1	48901	50097	
49500	A	T	59.6621	TYPE=snp	chrmt	S000007275	Q0140	VAR1	48901	50097	
49500	A	T	59.6621	TYPE=snp	chrmt	S000007275	Q0140	VAR1	48901	50097	
49500	A	T	59.7635	TYPE=snp	chrmt	S000007275	Q0140	VAR1	48901	50097	
49882	T	A	59.8449	TYPE=snp	chrmt	S000007275	Q0140	VAR1	48901	50097	
50029	T	C	59.8449	TYPE=snp	chrmt	S000007275	Q0140	VAR1	48901	50097	

50395	A	G	59.6621	TYPE=snp	chrmt	intergenic						
52177	A	G	59.7535	TYPE=snp	chrmt	intergenic						
52845	C	T	59.6621	TYPE=snp	chrmt	intergenic						
53061	TAA	TA	59.3806	TYPE=del	chrmt	intergenic						
53362	T	C	59.5363	TYPE=snp	chrmt	intergenic						
53419	A	C	59.8042	TYPE=snp	chrmt	intergenic						
53728	G	A	59.3806	TYPE=snp	chrmt	intergenic						
55598	T	C	60.0372	TYPE=snp	chrmt	intergenic						
55726	T	C	59.3806	TYPE=snp	chrmt	intergenic						
56468	T	C	60.0039	TYPE=snp	chrmt	intergenic						
57518	G	A	59.7635	TYPE=snp	chrmt	intergenic						
58550	A	G	59.6621	TYPE=snp	chrmt	intergenic						
59053	TAAAAAAAAAT	TAAAAAAAAT	59.3806	TYPE=del	chrmt	intergenic						
59096	A	T	59.5363	TYPE=snp	chrmt	intergenic						
59907	CTT	CT	59.5363	TYPE=del	chrmt	intergenic						
60801	T	A	59.5363	TYPE=snp	chrmt	intergenic						
62029	C	A	59.3806	TYPE=snp	chrmt	intergenic						
62274	A	T	59.7635	TYPE=snp	chrmt	intergenic						
62585	C	A	59.5363	TYPE=snp	chrmt	intergenic						
63032	ATTATTTATTTATTTATTTATTTATT	ATTATTTATTTATTTATTTATTTATTTATT	59.5363	TYPE=ins	chrmt	intergenic						
63481	ATTTTTTTTTA	ATTTTTTTTA	59.2846	TYPE=del	chrmt	intergenic						
63656	CGGGGGGGGGGT	CGGGGGGGGGT	59.6621	TYPE=del	chrmt	intergenic						
63656	CGGGGGGGGGGT	CGGGGGGGGGGGT	59.6621	TYPE=ins	chrmt	intergenic						
66611	T	C	59.4761	TYPE=snp	chrmt	intergenic						
66625	AATATATATATATATT	AATATATATATATT	59.9101	TYPE=del	chrmt	intergenic						
67176	T	C	59.3806	TYPE=snp	chrmt	intergenic						
67213	TATAATAATAATAATAATAATAATAATAA	TATAATAATAATAATAATAATAATAA	59.3806	TYPE=del	chrmt	intergenic						
67400	TCCCCCCCCCA	TCCCCCCCCCCA	59.9623	TYPE=ins	chrmt	intergenic						

67847	A	T	59.9101	TYPE=snp	chrmt	intergenic						
68180	T	A	59.8449	TYPE=snp	chrmt	intergenic						
68199	AATATATAT ATATT	AATATATAT ATT	59.3806	TYPE=del	chrmt	intergenic						
69449	C	T	59.8449	TYPE=snp	chrmt	intergenic						
69833	A	T	59.5363	TYPE=snp	chrmt	intergenic						
70625	A	G	59.3806	TYPE=snp	chrmt	intergenic						
70799	AATATATAT ATATATT	AATATATAT ATATATATT	59.9623	TYPE=ins	chrmt	intergenic						
70891	C	G	59.3806	TYPE=snp	chrmt	intergenic						
71177	TAAAAAAA AT	TAAAAAAA T	59.9623	TYPE=del	chrmt	intergenic						
71537	CATATATAT ATATATATT	CATATATAT ATATATT	59.2846	TYPE=del	chrmt	intergenic						
72338	TTATATATA TATATATAT ATATAT	TTATATATA TATATATAT ATAT	59.9101	TYPE=del	chrmt	intergenic						
72338	TTATATATA TATATATAT ATATAT	TTATATATA TATATATAT ATAT	59.7635	TYPE=del	chrmt	intergenic						
73114	AGGGGGGC	AGGGGGC	59.6621	TYPE=del	chrmt	intergenic						
73114	AGGGGGGC	AGGGGGC	59.3806	TYPE=del	chrmt	intergenic						
73136	TCC	TC	59.9623	TYPE=del	chrmt	intergenic						
73136	TCC	TC	59.9101	TYPE=del	chrmt	intergenic						
73136	TCC	TC	59.3806	TYPE=del	chrmt	intergenic						
74219	A	T	59.9101	TYPE=snp	chrmt	S000007281	Q0250	COX2	73758	74513	Cytochrome c OXidase	
74703	A	G	59.6621	TYPE=snp	chrmt	S000007282	Q0255		74495	75984		
75080	G	T	59.6621	TYPE=snp	chrmt	S000007282	Q0255		74495	75984		
75439	T	C	59.8449	TYPE=snp	chrmt	S000007282	Q0255		74495	75984		
75577	T	A	59.8449	TYPE=snp	chrmt	S000007282	Q0255		74495	75984		
75898	G	A	59.8449	TYPE=snp	chrmt	S000007282	Q0255		74495	75984		
75996	ACT	ACCT	59.9623	TYPE=ins	chrmt	intergenic						
76836	G	A	59.4254	TYPE=snp	chrmt	intergenic						
77650	A	G	59.3806	TYPE=snp	chrmt	intergenic						
77699	G	C	59.8449	TYPE=snp	chrmt	intergenic						
78159	T	C	59.3806	TYPE=snp	chrmt	intergenic						
78703	A	T	59.9101	TYPE=snp	chrmt	intergenic						
78703	A	T	59.7635	TYPE=snp	chrmt	intergenic						

78703	A	T	59.5363	TYPE=snp	chrmt	intergenic					
78703	A	T	59.6621	TYPE=snp	chrmt	intergenic					
78703	A	T	60.0637	TYPE=snp	chrmt	intergenic					
78703	A	T	59.9101	TYPE=snp	chrmt	intergenic					
78703	A	T	59.5363	TYPE=snp	chrmt	intergenic					
79712	C	T	59.5363	TYPE=snp	chrmt	S000007283	Q0275	COX3	79213	80022	Cytochrome c OXidase
81808	A	G	59.3806	TYPE=snp	chrmt	intergenic					
82507	T	A	59.6621	TYPE=snp	chrmt	intergenic					
82507	T	A	59.5992	TYPE=snp	chrmt	intergenic					
82507	T	A	59.5363	TYPE=snp	chrmt	intergenic					
82507	T	A	59.4585	TYPE=snp	chrmt	intergenic					
82507	T	A	59.5363	TYPE=snp	chrmt	intergenic					
82507	T	A	59.7635	TYPE=snp	chrmt	intergenic					
82507	T	A	59.6621	TYPE=snp	chrmt	intergenic					
82507	T	A	59.3806	TYPE=snp	chrmt	intergenic					
82507	T	A	59.4316	TYPE=snp	chrmt	intergenic					
82507	T	A	60.0338	TYPE=snp	chrmt	intergenic					
82507	T	A	60.0039	TYPE=snp	chrmt	intergenic					
82507	T	A	60.0637	TYPE=snp	chrmt	intergenic					
82507	T	A	59.3806	TYPE=snp	chrmt	intergenic					
82507	T	A	59.5992	TYPE=snp	chrmt	intergenic					
82507	T	A	59.9101	TYPE=snp	chrmt	intergenic					
82507	T	A	59.9362	TYPE=snp	chrmt	intergenic					
82507	T	A	59.5363	TYPE=snp	chrmt	intergenic					
82507	T	A	60.0039	TYPE=snp	chrmt	intergenic					
82507	T	A	60.0039	TYPE=snp	chrmt	intergenic					
82507	T	A	59.8449	TYPE=snp	chrmt	intergenic					
82507	T	A	59.4585	TYPE=snp	chrmt	intergenic					
82507	T	A	59.7635	TYPE=snp	chrmt	intergenic					
82507	T	A	59.9101	TYPE=snp	chrmt	intergenic					
82507	T	A	59.7635	TYPE=snp	chrmt	intergenic					
82507	T	A	59.5992	TYPE=snp	chrmt	intergenic					
82507	T	A	59.9623	TYPE=snp	chrmt	intergenic					
82507	T	A	59.3806	TYPE=snp	chrmt	intergenic					
82507	T	A	59.5363	TYPE=snp	chrmt	intergenic					
82507	T	A	60.0039	TYPE=snp	chrmt	intergenic					
82593	C	T	59.6621	TYPE=snp	chrmt	intergenic					
83121	TAAAAAAA AAT	TAAAAAAA AT	59.3806	TYPE=del	chrmt	intergenic					

83555	T	A	59.5363	TYPE=snp	chrmt	intergenic					
83766	C	A	59.5363	TYPE=snp	chrmt	intergenic					
84247	GCCCCCCG	GCCCCCCC G	59.5363	TYPE=ins	chrmt	intergenic					
84247	GCCCCCCG	GCCCCCCC G	59.5363	TYPE=ins	chrmt	intergenic					
85596	GCCCCCCC G	GCCCCCCCC G	59.6621	TYPE=ins	chrmt	S000007284	Q0297		85554	85709	
85640	CC	CCTATACTA TAC	59.6621	TYPE=ins	chrmt	S000007284	Q0297		85554	85709	
10135	TCC	TC	59.9101	TYPE=del	chrV	intergenic					
28523	A	G	59.7635	TYPE=snp	chrV	S000000791	YEL065W	SIT1	27657	29543	Siderophore Iron Transport
45034	G	T	59.7635	TYPE=snp	chrV	S000000783	YEL057C	SDD1	45020	45721	Suppressor of Degenerative Death
70019	C	A	59.7635	TYPE=snp	chrV	S000000770	YEL044W	IES6	69757	70257	Ino Eighty Subunit
104597	G	C	59.7635	TYPE=snp	chrV	S000000751	YEL025C		102581	106147	
128887	C	T	59.5363	TYPE=snp	chrV	S000000739	YEL013W	VAC8	128825	130561	VACuole related
136718	A	G	59.3806	TYPE=snp	chrV	S000028741	YEL009C-A		136371	136778	
188761	C	A	59.3806	TYPE=snp	chrV	S000000818	YER016W	BIM1	188277	189311	BInding to Microtubules
225419	A	G	59.5363	TYPE=snp	chrV	intergenic					
320698	CAAAAAAA AT	CAAAAAAA T	59.9101	TYPE=del	chrV	S000000882	YER080W	AIM9	319963	321846	Altered Inheritance rate of Mitochondria
346405	T	A	59.3806	TYPE=snp	chrV	S000000895	YER093C	TSC11	343320	347612	Temperature-sensitive Suppressors of Csg2 mutants
352394	A	G	59.7635	TYPE=snp	chrV	S000000898	YER096W	SHC1	351698	353236	Sporulation-specific Homolog of CSD4
396428	ATTTTTTTC	ATTTTTTC	59.3806	TYPE=del	chrV	intergenic					
398692	T	C	59.7635	TYPE=snp	chrV	S000000920	YER118C	SHO1	397952	399055	Synthetic, High Osmolarity-sensitive
430751	GAAAAAAA AG	GAAAAAAA AAG	59.6621	TYPE=ins	chrV	intergenic					
431898	G	C	60.0637	TYPE=snp	chrV	intergenic					
546185	A	G	59.9101	TYPE=snp	chrV	S000000979	YER177W	BMH1	545611	546414	Brain Modulosignalin Homolog
23603	C	A	59.7635	TYPE=snp	chrVI	S000001841	YFL053W	DAK2	23423	25198	DihydroxyAcetone Kinase
24478	A	G	60.0372	TYPE=snp	chrVI	S000001841	YFL053W	DAK2	23423	25198	DihydroxyAcetone Kinase
39881	C	T	59.7635	TYPE=snp	chrVI	S000001846	YFL048C	EMP47	38843	40180	
40326	G	A	59.9101	TYPE=snp	chrVI	intergenic					

76169	ATTTTTTTT TTC	ATTTTTTTT TTTC	59.9623	TYPE=ins	chrVI	intergenic					
89460	T	C	59.2447	TYPE=snp	chrVI	S000001870	YFL024C	EPL1	87847	90345	Enhancer of Polycomb Like
110773	A	T	59.7128	TYPE=snp	chrVI	S000001882	YFL012W		110647	111093	
110849	G	C	59.7128	TYPE=snp	chrVI	S000001882	YFL012W		110647	111093	
159869	G	T	59.7635	TYPE=snp	chrVI	S000001903	YFR007W	YFH7	159299	160360	
196411	T	C	59.7635	TYPE=snp	chrVI	intergenic					
229128	T	C	60.0372	TYPE=snp	chrVI	S000001933	YFR037C	RSC8	227513	229186	Remodel the Structure of Chromatin
29709	T	C	59.5363	TYPE=snp	chrVII	S000003220	YGL251C	HFM1	27921	31636	Helicase Family Member
82733	A	T	59.3806	TYPE=snp	chrVII	S000003188	YGL220W	BOL2	82374	82736	BolA-like protein
99954	C	A	59.7635	TYPE=snp	chrVII	S000003175	YGL207W	SPT16	98969	102076	SuPpressor of Ty
142123	A	G	59.9101	TYPE=snp	chrVII	S000003161	YGL193C		141916	142227	
199203	C	T	59.5363	TYPE=snp	chrVII	intergenic					
228600	GAAAAAAA T	GAAAAAAT	60.0039	TYPE=del	chrVII	intergenic					
306513	T	C	59.9101	TYPE=snp	chrVII	intergenic					
348376	G	A	59.6621	TYPE=snp	chrVII	S000003054	YGL086W	MAD1	347119	349368	Mitotic Arrest-Deficient
364976	TAAAAAG	TAAAAG	59.8449	TYPE=del	chrVII	S000003044	YGL076C	RPL7A	364335	365996	Ribosomal Protein of the Large subunit
401674	C	A	59.7128	TYPE=snp	chrVII	intergenic					
535388	G	C	60.0372	TYPE=snp	chrVII	intergenic					
560306	A	G	60.0372	TYPE=snp	chrVII	intergenic					
567719	TAA	TA	60.0372	TYPE=del	chrVII	intergenic					
579366	C	A	59.3806	TYPE=snp	chrVII	intergenic					
610596	A	G	59.3806	TYPE=snp	chrVII	S000003292	YGR060W	ERG25	610564	611493	ERGosterol biosynthesis
628493	C	T	59.9101	TYPE=snp	chrVII	S000003302	YGR070W	ROM1	627806	631273	RhO1 Multicopy suppressor
636412	A	G	59.9101	TYPE=snp	chrVII	S000003307	YGR075C	PRP38	636147	636875	Pre-mRNA Processing
654766	C	T	59.5363	TYPE=snp	chrVII	S000003320	YGR088W	CTT1	654634	656322	CaTalase T
722138	A	T	59.5363	TYPE=snp	chrVII	S000003348	YGR116W	SPT6	720409	724764	SuPpressor of Ty's
745425	T	A	59.7635	TYPE=snp	chrVII	S000003357	YGR125W		742325	745435	
752995	A	G	59.7635	TYPE=snp	chrVII	S000003362	YGR130C		751394	753844	
800526	A	G	59.3806	TYPE=snp	chrVII	intergenic					
847067	A	T	59.9101	TYPE=snp	chrVII	S000003407	YGR175C	ERG1	846933	848423	ERGosterol biosynthesis
869886	T	C	59.7635	TYPE=snp	chrVII	S000003418	YGR186W	TFG1	867774	869981	Transcription Factor G
944916	T	C	59.9101	TYPE=snp	chrVII	intergenic					
959405	C	A	59.9101	TYPE=snp	chrVII	intergenic					
1006278	CGT	CT	59.9101	TYPE=del	chrVII	S000003489	YGR257C	MTM1	1E+06	1E+06	Manganese Trafficking factor for Mitochondrial SOD2

1014112	CTTTTTTTG	CTTTTTTTT G	59.3806	TYPE=ins	chrVII	intergenic					
1019746	A	T	59.3806	TYPE=snp	chrVII	S000003496	YGR264C	MES1	1E+06	1E+06	MEthionyl-tRNA Synthetase
28284	G	A	59.9101	TYPE=snp	chrVIII	S000001027	YHL035C	VMR1	27978	32756	Vacuolar Multidrug Resistance
29070	G	T	59.3806	TYPE=snp	chrVIII	S000001027	YHL035C	VMR1	27978	32756	Vacuolar Multidrug Resistance
76105	T	C	59.3806	TYPE=snp	chrVIII	S000001006	YHL014C	YLF2	76097	77314	
85508	TTATATATA T	TTATATAT	59.7635	TYPE=del	chrVIII	intergenic					
85508	TTATATATA T	TTATATAT	59.8449	TYPE=del	chrVIII	intergenic					
85508	TTATATATA T	TTATATAT	59.8449	TYPE=del	chrVIII	intergenic					
85508	TTATATATA T	TTATATAT	59.8449	TYPE=del	chrVIII	intergenic					
92085	ATTTTTA	ATTTTTTA	60.0637	TYPE=ins	chrVIII	intergenic					
129290	G	T	59.5363	TYPE=snp	chrVIII	intergenic					
132087	C	A	59.5363	TYPE=snp	chrVIII	S000001056	YHR014W	SPO13	132047	132922	SPOrulation
140787	G	A	59.9101	TYPE=snp	chrVIII	S000001060	YHR018C	ARG4	140011	141402	ARGinine requiring
169069	T	A	59.5363	TYPE=snp	chrVIII	S000001072	YHR030C	SLT2	168890	170344	Suppressor of the LyTic phenotype
231761	G	T	59.5363	TYPE=snp	chrVIII	S000001109	YHR067W	HTD2	230970	231812	Hydroxyacyl-Thioester Dehydratase
251531	G	T	59.8368	TYPE=snp	chrVIII	S000001118	YHR076W	PTC7	251101	252225	Phosphatase type Two C
279792	T	C	59.5363	TYPE=snp	chrVIII	intergenic					
304953	A	G	59.3806	TYPE=snp	chrVIII	S000001141	YHR099W	TRA1	302761	313995	similar to human TRRAP
341348	A	G	59.9101	TYPE=snp	chrVIII	S000001157	YHR115C	DMA1	340109	341359	Defective in Mitotic Arrest
94382	T	C	59.9101	TYPE=snp	chrX	S000003711	YJL175W		94049	94561	
94382	T	C	59.9101	TYPE=snp	chrX	S000003712	YJL176C	SWI3	92053	94530	SWItching deficient
96820	T	A	59.7635	TYPE=snp	chrX	intergenic					
110801	T	C	59.9101	TYPE=snp	chrX	S000003700	YJL164C	TPK1	109966	111159	Takashi's Protein Kinase
131441	G	A	59.9101	TYPE=snp	chrX	S000003690	YJL154C	VPS35	131101	133935	Vacuolar Protein Sorting
131605	G	T	59.7635	TYPE=snp	chrX	S000003690	YJL154C	VPS35	131101	133935	Vacuolar Protein Sorting
135940	T	C	59.7635	TYPE=snp	chrX	intergenic					
153033	T	C	59.5363	TYPE=snp	chrX	intergenic					
178042	CTTTTTTTTT G	CTTTTTTTT G	59.6621	TYPE=del	chrX	intergenic					
223605	CAAAAAAA G	CAAAAAAG	60.0637	TYPE=del	chrX	intergenic					

251426	A	G	59.7635	TYPE=snp	chrX	S000003631	YJL095W	BCK1	247255	251691	Bypass of C Kinase
269004	G	A	60.0372	TYPE=snp	chrX	S000003624	YJL088W	ARG3	268799	269815	ARGinine requiring
375550	C	A	59.5363	TYPE=snp	chrX	S000003575	YJL038C	LOH1	375115	375774	Loss Of Heterozygosity
376200	G	T	59.9101	TYPE=snp	chrX	intergenic					
456066	GATATATATATATATATATATATATATATATATATATATG	GATATATATATATATATATATATATATATATATATATG	59.5363	TYPE=ins	chrX	intergenic					
566760	C	T	59.5363	TYPE=snp	chrX	S000003827	YJR066W	TOR1	559416	566828	Target Of Rapamycin
573102	T	C	59.3806	TYPE=snp	chrX	S000003835	YJR074W	MOG1	573095	573751	Multicopy suppressor Of ts Gsp1
601566	AGG	AG	59.5363	TYPE=del	chrX	S000003852	YJR092W	BUD4	598735	603078	BUD site selection
646136	CTTTTTTTTTTA	CTTTTTTTTTTTA	59.5363	TYPE=ins	chrX	S000003880	YJR119C	JHD2	644304	646490	JmjC domain-containing Histone Demethylase
661417	G	T	60.0372	TYPE=snp	chrX	S000003888	YJR127C	RSF2	658917	663059	ReSpiration Factor
695407	T	C	60.0372	TYPE=snp	chrX	S000003901	YJR140C	HIR3	690750	695696	HIstone Regulation
724796	T	A	59.7635	TYPE=snp	chrX	intergenic					
13851	G	A	59.5363	TYPE=snp	chrXI	intergenic					
51223	C	T	59.7635	TYPE=snp	chrXI	S000001688	YKL205W	LOS1	50051	53353	Loss Of Suppression
90831	G	C	59.5363	TYPE=snp	chrXI	S000001670	YKL187C	FAT3	89284	91536	FATty acid transporter 3
103812	G	A	59.5363	TYPE=snp	chrXI	S000001665	YKL182W	FAS1	100671	106826	Fatty Acid Synthetase
201034	T	A	59.9101	TYPE=snp	chrXI	S000001611	YKL128C	PMU1	200884	201771	PhosphoMUtase
238477	A	G	59.5363	TYPE=snp	chrXI	S000001589	YKL106W	AAT1	237536	238891	Aspartate AminoTransferase
256772	G	T	59.5363	TYPE=snp	chrXI	S000001581	YKL098W	MTC2	256770	257843	Maintenance of Telomere Capping
315546	A	T	59.9101	TYPE=snp	chrXI	S000001549	YKL066W		315281	315724	
342593	CTTTTTTTTTTG	CTTTTTTTTTG	59.9101	TYPE=del	chrXI	intergenic					
356157	T	C	59.3806	TYPE=snp	chrXI	intergenic					
404467	C	T	60.0372	TYPE=snp	chrXI	S000001501	YKL018W	SWD2	404102	405091	Set1c, WD40 repeat protein
421623	C	A	59.9101	TYPE=snp	chrXI	S000001493	YKL010C	UFD4	421424	425875	Ubiquitin Fusion Degradation protein
522453	A	T	59.5363	TYPE=snp	chrXI	S000001752	YKR044W	UIP5	522015	523346	Ulp1 Interacting Protein
533507	T	C	59.5363	TYPE=snp	chrXI	intergenic					

560413	GGTAACTTGAAATGTATTGACGAGGAG	GG	60.0637	TYPE=del	chrXI	S000001770	YKR062W	TFA2	559666	560652	Transcription Factor a, subunit 2
570296	A	T	59.4585	TYPE=snp	chrXI	intergenic					
573772	T	A	59.3806	TYPE=snp	chrXI	S000001778	YKR070W		573574	574632	
608524	T	A	59.7635	TYPE=snp	chrXI	intergenic					
51406	C	A	59.3806	TYPE=snp	chrXII	S000003966	YLL043W	FPS1	49938	51947	fdp1 Suppressor
77236	G	A	59.7635	TYPE=snp	chrXII	S000003954	YLL031C	GPI13	77152	80205	GlycosylPhosphatidylInositol anchor biosynthesis
77959	T	A	59.5363	TYPE=snp	chrXII	S000003954	YLL031C	GPI13	77152	80205	GlycosylPhosphatidylInositol anchor biosynthesis
85200	G	T	59.3806	TYPE=snp	chrXII	S000003951	YLL028W	TPO1	84804	86564	Transporter of POlyamines
134608	T	C	59.5363	TYPE=snp	chrXII	S000003930	YLL007C	LMO1	134302	136299	eLMO homolog
151298	A	G	59.3806	TYPE=snp	chrXII	intergenic					
157447	C	T	59.3806	TYPE=snp	chrXII	S000003993	YLR003C	CMS1	156855	157730	Complementation of Mcm-10 Suppressor
222659	T	A	59.5363	TYPE=snp	chrXII	intergenic					
223607	G	C	59.8368	TYPE=snp	chrXII	intergenic					
284201	G	T	59.5363	TYPE=snp	chrXII	S000004067	YLR077W	FMP25	283872	285623	Found in Mitochondrial Proteome
303160	G	T	60.0372	TYPE=snp	chrXII	S000004076	YLR086W	SMC4	302243	306499	Structural Maintenance of Chromosomes
333973	A	T	59.9101	TYPE=snp	chrXII	S000004086	YLR096W	KIN2	332590	336033	KINase
362804	A	G	59.9101	TYPE=snp	chrXII	S000004096	YLR106C	REA1	349006	363738	Ribosome Export/Assembly
395625	C	G	59.3806	TYPE=snp	chrXII	intergenic					
544264	C	G	59.5363	TYPE=snp	chrXII	S000004186	YLR196W	PWP1	543968	545698	Periodic tryptophan (W) Protein
572034	A	G	59.5363	TYPE=snp	chrXII	intergenic					
616752	A	C	59.7635	TYPE=snp	chrXII	S000004229	YLR239C	LIP2	616332	617318	LIPoyl ligase
619549	A	G	59.7635	TYPE=snp	chrXII	S000004230	YLR240W	VPS34	617533	620160	Vacuolar Protein Sorting
623561	A	G	59.5363	TYPE=snp	chrXII	S000004232	YLR242C	ARV1	622918	623883	ARE2 Required for Viability
727821	T	C	59.3806	TYPE=snp	chrXII	S000004290	YLR299W	ECM38	726069	728051	ExtraCellular Mutant
739547	G	T	59.5363	TYPE=snp	chrXII	S000004296	YLR305C	STT4	738161	743863	STaurosporine and Temperature sensitive
742790	A	G	59.3806	TYPE=snp	chrXII	S000004296	YLR305C	STT4	738161	743863	STaurosporine and Temperature sensitive
750917	T	C	59.8449	TYPE=snp	chrXII	S000004300	YLR309C	IMH1	749034	751769	shares with Integrins and Myosins significant Homology

758754	G	A	59.5363	TYPE=snp	chrXII	S000004303	YLR312C	ATG39	757637	758833	AuTophaGy related
840834	C	A	59.5363	TYPE=snp	chrXII	S000004348	YLR356W	ATG33	840321	840914	AuTophaGy related
854922	T	A	59.3806	TYPE=snp	chrXII	intergenic					
876558	A	G	59.7635	TYPE=snp	chrXII	S000004370	YLR378C	SEC61	875736	877178	SECretory
925883	A	G	59.7635	TYPE=snp	chrXII	S000004395	YLR403W	SFP1	925568	927619	Split Finger Protein
981776	A	T	60.0505	TYPE=snp	chrXII	intergenic					
999875	C	A	60.0372	TYPE=snp	chrXII	S000004422	YLR430W	SEN1	993434	1E+06	Splicing ENdonuclease
1036344	C	T	60.0372	TYPE=snp	chrXII	S000004443	YLR451W	LEU3	1E+06	1E+06	LEUcine biosynthesis
1040366	A	G	59.7635	TYPE=snp	chrXII	S000004444	YLR452C	SST2	1E+06	1E+06	SuperSensiTive
143842	T	C	59.5363	TYPE=snp	chrXIII	S000004530	YML065W	ORC1	142210	144954	Origin Recognition Complex
169888	TAAAAAAA AT	TAAAAAAA T	59.8837	TYPE=del	chrXIII	intergenic					
170769	C	T	59.3806	TYPE=snp	chrXIII	S000004516	YML052W	SUR7	170402	171310	SUppressor of Rvs167 mutation
241917	A	T	59.7128	TYPE=snp	chrXIII	intergenic					
304620	A	G	59.7635	TYPE=snp	chrXIII	S000004618	YMR016C	SOK2	303236	305593	Suppressor Of Kinase
414637	C	G	59.7635	TYPE=snp	chrXIII	S000004680	YMR075W	RCO1	413982	416036	
426887	CAA	CA	59.5363	TYPE=del	chrXIII	S000004685	YMR080C	NAM7	426712	429627	Nuclear Accommodation of Mitochondria
431357	A	G	60.0372	TYPE=snp	chrXIII	intergenic					
452573	C	A	59.7635	TYPE=snp	chrXIII	S000004698	YMR092C	AIP1	451632	453479	Actin Interacting Protein
468456	C	A	59.3806	TYPE=snp	chrXIII	S000004707	YMR101C	SRT1	468445	469476	Suppressor of Rer-Two
522996	A	G	59.3806	TYPE=snp	chrXIII	S000004734	YMR127C	SAS2	522329	523345	Something About Silencing
561877	AATATATAT ATATATATA TATATATAT ATATATG	AATATATAT ATATATATA TATATATAT ATATG	59.3806	TYPE=del	chrXIII	intergenic					
581768	T	A	59.5363	TYPE=snp	chrXIII	S000004772	YMR162C	DNF3	578951	583921	Drs2 Neo1 Family
750124	G	T	59.9101	TYPE=snp	chrXIII	S000004853	YMR240C	CUS1	749930	751240	Cold sensitive U2 snRNA Suppressor
786005	C	A	59.7635	TYPE=snp	chrXIII	S000004872	YMR259C	TRM732	784621	788883	Transfer RNA Methyltransferase
789224	T	C	59.6621	TYPE=snp	chrXIII	intergenic					
810490	C	T	59.3806	TYPE=snp	chrXIII	S000004885	YMR272C	SCS7	809623	810777	Suppressor of Ca2+ Sensitivity
810490	C	T	59.3806	TYPE=snp	chrXIII	S000028695	YMR272W-A		810466	810579	
831156	A	T	59.9101	TYPE=snp	chrXIII	S000004893	YMR280C	CAT8	827028	831329	CATabolite repression
39299	A	G	59.7635	TYPE=snp	chrXIV	S000005262	YNL318C	HXT14	38707	40329	HeXose Transporter
41597	G	C	59.7635	TYPE=snp	chrXIV	S000005261	YNL317W	PFS2	40619	42016	Polyadenylation Factor Subunit

53324	T	C	59.9101	TYPE=snp	chrXIV	S000005253	YNL309W	STB1	52662	53924	Sin Three Binding protein
75805	C	A	59.9101	TYPE=snp	chrXIV	S000005241	YNL297C	MON2	71673	76583	MONensin sensitivity
150382	A	T	59.5363	TYPE=snp	chrXIV	S000005206	YNL262W	POL2	148212	154880	POLymerase
204604	C	G	59.3806	TYPE=snp	chrXIV	S000005182	YNL238W	KEX2	202428	204872	Killer EXpression defective
250506	G	C	60.0372	TYPE=snp	chrXIV	intergenic					
311585	G	A	59.3806	TYPE=snp	chrXIV	S000005116	YNL172W	APC1	310636	315882	Anaphase Promoting Complex subunit
330950	A	T	59.9101	TYPE=snp	chrXIV	intergenic					
348518	T	A	59.3806	TYPE=snp	chrXIV	S000005095	YNL151C	RPC31	347766	348521	RNA Polymerase C
377887	G	T	59.5363	TYPE=snp	chrXIV	S000005076	YNL132W	KRE33	375321	378491	Killer toxin REsistant
442305	C	T	59.9101	TYPE=snp	chrXIV	S000005041	YNL097C	PHO23	441366	442358	PHOsphate metabolism
467679	T	A	59.7635	TYPE=snp	chrXIV	S000005029	YNL085W	MKT1	467131	469623	Maintenance of K2 Killer Toxin
472369	A	G	59.7635	TYPE=snp	chrXIV	S000005027	YNL083W	SAL1	471377	472861	Suppressor of Aac2 Lethality
638119	A	G	59.7635	TYPE=snp	chrXIV	S000005289	YNR006W	VPS27	636986	638854	Vacuolar Protein Sorting
695311	C	A	59.3806	TYPE=snp	chrXIV	S000005320	YNR037C	RSM19	695052	695327	Ribosomal Small subunit of Mitochondria
726896	T	C	60.0637	TYPE=snp	chrXIV	intergenic					
757539	T	A	59.8368	TYPE=snp	chrXIV	S000005350	YNR067C	DSE4	755746	759099	Daughter Specific Expression
780393	A	T	59.9623	TYPE=snp	chrXIV	S000005358	YNR075W	COS10	779916	781040	COnserved Sequence
2982	C	T	60.0505	TYPE=snp	chrXV	intergenic					
38588	G	A	59.5363	TYPE=snp	chrXV	intergenic					
92007	T	C	59.5363	TYPE=snp	chrXV	intergenic					
241296	A	G	59.7635	TYPE=snp	chrXV	S000005408	YOL048C	RRT8	240204	241310	Regulator of rDNA Transcription
244687	C	T	59.7635	TYPE=snp	chrXV	S000005405	YOL045W	PSK2	243497	246802	Pas domain-containing Serine/threonine protein Kinase
247599	A	T	59.7635	TYPE=snp	chrXV	S000005404	YOL044W	PEX15	247150	248301	PEroXisome related
295723	A	G	59.7635	TYPE=snp	chrXV	S000005376	YOL016C	CMK2	294777	296120	CalModulin dependent protein Kinase
312841	GTT	GT	60.0039	TYPE=del	chrXV	intergenic					
318789	C	T	59.3806	TYPE=snp	chrXV	S000005364	YOL004W	SIN3	316938	321548	Switch INdependent
328993	G	T	60.0372	TYPE=snp	chrXV	S000005527	YOR001W	RRP6	326832	329033	Ribosomal RNA Processing
371162	TAAAAAG	TAAAAG	60.0039	TYPE=del	chrXV	intergenic					
374195	C	A	59.5363	TYPE=snp	chrXV	S000005548	YOR022C	DDL1	373710	375857	DDHD Domain-containing Lipase

396217	T	C	59.9101	TYPE=snp	chrXV	S000005560	YOR034C	AKR2	394837	397086	AnKyrin Repeat-containing protein
498503	T	C	60.0372	TYPE=snp	chrXV	S000005619	YOR093C	CMR2	497506	502452	Changed Mutation Rate
511358	A	T	59.7635	TYPE=snp	chrXV	intergenic					
557425	T	A	59.9101	TYPE=snp	chrXV	S000005650	YOR124C	UBP2	554824	558642	UBiquitin-specific Protease
565353	T	C	59.7635	TYPE=snp	chrXV	S000005654	YOR128C	ADE2	564476	566191	ADEnine requiring
568703	A	T	59.3806	TYPE=snp	chrXV	S000005655	YOR129C	AFI1	566877	569558	ArF3-Interacting protein
613019	G	A	59.6621	TYPE=snp	chrXV	S000005677	YOR151C	RPB2	612997	616671	RNA Polymerase B
613024	TAA	TA	59.6621	TYPE=del	chrXV	S000005677	YOR151C	RPB2	612997	616671	RNA Polymerase B
639617	T	C	59.3806	TYPE=snp	chrXV	S000005688	YOR162C	YRR1	639560	641992	Yeast Reveromycin-A Resistant
659262	G	A	59.3806	TYPE=snp	chrXV	S000005700	YOR174W	MED4	658747	659601	MEDiator complex
678783	T	A	59.5363	TYPE=snp	chrXV	S000005708	YOR182C	RPS30B	678191	678793	Ribosomal Protein of the Small subunit
710173	T	C	59.572	TYPE=snp	chrXV	intergenic					
716514	C	T	59.5363	TYPE=snp	chrXV	S000005722	YOR196C	LIP5	715593	716837	LIPoic acid
718146	C	A	59.5363	TYPE=snp	chrXV	S000005723	YOR197W	MCA1	717086	718384	MetaCAspase
770916	G	A	59.5363	TYPE=snp	chrXV	S000005756	YOR230W	WTM1	770800	772113	WD repeat containing Transcriptional Modulator
771295	G	A	59.3806	TYPE=snp	chrXV	S000005756	YOR230W	WTM1	770800	772113	WD repeat containing Transcriptional Modulator
987454	T	C	59.7635	TYPE=snp	chrXV	S000005875	YOR348C	PUT4	986899	988782	Proline UTilization
994749	G	C	59.7635	TYPE=snp	chrXV	S000005877	YOR350C	MNE1	992864	994855	
1010124	T	C	59.7635	TYPE=snp	chrXV	intergenic					
49136	A	G	59.5363	TYPE=snp	chrXVI	S000006182	YPL261C		48996	49304	
66069	C	T	59.5363	TYPE=snp	chrXVI	S000006177	YPL256C	CLN2	64977	66614	CycLiN
71681	ATTTTTTTTTTG	ATTTTTTTTTG	59.5363	TYPE=del	chrXVI	S000006174	YPL253C	VIK1	71063	73006	Vegetative Interaction with Kar3p
73282	A	G	59.9101	TYPE=snp	chrXVI	intergenic					
91323	T	C	59.7635	TYPE=snp	chrXVI	S000006163	YPL242C	IQG1	90622	95109	IQGAP-related protein
114944	T	C	59.7635	TYPE=snp	chrXVI	intergenic					
127937	G	T	59.9101	TYPE=snp	chrXVI	S000006145	YPL224C	MMT2	126634	128088	Mitochondrial Metal Transporter
142458	C	G	59.9101	TYPE=snp	chrXVI	S000006138	YPL217C	BMS1	139620	143171	BMh Sensitive
240697	T	C	59.5363	TYPE=snp	chrXVI	S000006085	YPL164C	MLH3	239350	241497	MutL Homolog
273483	G	T	59.3806	TYPE=snp	chrXVI	S000006068	YPL147W	PXA1	273255	275867	PeroXisomal ABC-transporter
287202	C	G	59.7635	TYPE=snp	chrXVI	intergenic					
297876	A	G	59.3806	TYPE=snp	chrXVI	S000006056	YPL135W	ISU1	297553	298050	IScU homolog

298502	AATATATAT ATATATATA	AATATATAT ATATATATA TA	59.3806	TYPE=ins	chrXVI	intergenic					
340595	G	A	60.0372	TYPE=snp	chrXVI	S000006032	YPL111W	CAR1	339944	340945	Catabolism of ARginine
388599	G	T	59.7635	TYPE=snp	chrXVI	S000006006	YPL085W	SEC16	387067	393654	SECretory
397890	T	A	59.3806	TYPE=snp	chrXVI	S000006004	YPL083C	SEN54	396702	398105	Splicing ENdonuclease
423628	TGG	TG	59.5363	TYPE=del	chrXVI	S000005990	YPL069C	BTS1	422885	423892	Bet Two Suppressor
568975	T	A	59.3806	TYPE=snp	chrXVI	S000006210	YPR006C	ICL2	567269	568996	IsoCitrate Lyase
569138	T	A	59.7635	TYPE=snp	chrXVI	intergenic					
587691	A	G	60.0372	TYPE=snp	chrXVI	intergenic					
781511	T	A	59.7635	TYPE=snp	chrXVI	intergenic					
801718	C	T	59.5363	TYPE=snp	chrXVI	S000006339	YPR135W	CTF4	799234	802017	ChrTransmission Fidelity
850566	G	A	60.0637	TYPE=snp	chrXVI	intergenic					
856452	GACAGCTT CTCA	GA	60.0372	TYPE=del	chrXVI	intergenic					
856465	A	G	60.0372	TYPE=snp	chrXVI	intergenic					
857024	T	C	59.6621	TYPE=snp	chrXVI	intergenic					
899302	G	T	59.5363	TYPE=snp	chrXVI	S000006385	YPR181C	SEC23	897361	899667	SECretory
906085	G	T	59.7635	TYPE=snp	chrXVI	S000006388	YPR184W	GDB1	902044	906654	Glycogen DeBranching
918854	G	A	59.7635	TYPE=snp	chrXVI	S000006394	YPR190C	RPC82	917077	919041	RNA Polymerase C
919175	T	C	59.7635	TYPE=snp	chrXVI	intergenic					

AT22

POS	REF	ALT	QUAL	TYPE	Chromosome	DB_identifier	GeneSysName	GeneStdName	Start	End	Protein name
34484	C	T	56.199	snp	ChrI	S000000057	YAL061W	BDH2	33448	34701	
61437	T	C	56.199	snp	ChrI	S000000040	YAL042W	ERV46	61316	62563	ER Vesicle
61437	T	C	56.199	snp	ChrI	S000002138	YAL042C-A		61231	61608	
92429	TAAAAAAA AAG	TAAAAAAA AG	56.264	del	ChrI						
97867	T	C	56.04	snp	ChrI	S000000024	YAL026C	DRS2	95630	99697	Deficiency of Ribosomal Subunits
145610	GT	GCT	56.157	ins	ChrI	S000000002	YAL002W	VPS8	143707	147531	Vacuolar Protein Sorting
151368	C	T	56.157	snp	ChrI						
193585	G	A	56.105	snp	ChrI	S000000081	YAR042W	SWH1	192619	196185	
201096	TGG	TG	56.04	del	ChrI						
48100	G	T	56.152	snp	ChrII	S000000187	YBL091C	MAP2	47363	48628	Methionine AminoPeptidase
116308	T	A	56.105	snp	ChrII	S000000151	YBL055C		115573	116829	

188247	C	A	56.279	snp	ChrII	S000000113	YBL017C	PEP1	186844	191583	carboxyPEPtidase Y-deficient
216368	C	A	56.279	snp	ChrII						
533485	A	G	56.105	snp	ChrII	S000000348	YBR144C		533235	533549	
623426	G	T	56.04	snp	ChrII						
653152	C	T	98.465	snp	ChrII						
758767	G	T	56.199	snp	ChrII	S000000480	YBR276C	PPS1	757620	760043	Protein Phosphatase S phase
758781	T	G	56.199	snp	ChrII	S000000480	YBR276C	PPS1	757620	760043	Protein Phosphatase S phase
785595	T	C	56.199	snp	ChrII						
17536	A	G	56.04	snp	ChrIII	S000000568	YCL063W	VAC17	17290	18561	VACuole related
22070	C	A	56.279	snp	ChrIII	S000000566	YCL061C	MRC1	18816	22106	Mediator of the Replication Checkpoint
29646	T	A	56.04	snp	ChrIII						
272910	CAAAA	GAAAG	56.04	complex	ChrIII						
312959	A	T	56.098	snp	ChrIII	S000000703	YCR106W	RDS1	310958	313456	Regulator of Drug Sensitivity
31489	C	T	56.199	snp	ChrIV	S000002396	YDL237W	AIM6	30657	31829	Altered Inheritance rate of Mitochondria
75318	A	G	56.157	snp	ChrIV	S000002373	YDL214C	PRR2	74446	76545	Pheromone Response Regulator
78257	T	C	56.04	snp	ChrIV						
133359	A	T	56.157	snp	ChrIV						
167716	G	A	56.199	snp	ChrIV	S000002320	YDL161W	ENT1	167714	169078	Epsin N-Terminal homology
168755	A	T	56.157	snp	ChrIV	S000002320	YDL161W	ENT1	167714	169078	Epsin N-Terminal homology
233419	A	G	56.105	snp	ChrIV	S000002286	YDL128W	VCX1	232652	233887	VaCuolar H+/Ca2+ eXchanger
297954	G	A	56.199	snp	ChrIV	S000002247	YDL089W	NUR1	296820	298274	NUclear Rim1
313254	G	A	56.178	snp	ChrIV	S000002237	YDL079C	MRK1	312951	314748	Mds1p Related Kinase
333803	C	A	56.279	snp	ChrIV	S000002226	YDL068W		333500	333829	
333803	C	A	56.279	snp	ChrIV	S000002227	YDL069C	CBS1	333121	333810	Cytochrome B Synthesis
366332	G	A	56.178	snp	ChrIV						
414062	C	A	56.279	snp	ChrIV	S000002179	YDL021W	GPM2	413953	414888	Glycerate PhosphoMutase
524936	GTT	GT	56.279	del	ChrIV						
525814	A	G	56.105	snp	ChrIV	S000002444	YDR037W	KRS1	525440	527215	Lysyl (K) tRNA Synthetase
592097	G	C	56.105	snp	ChrIV						

608680	A	G	56.04	snp	ChrIV	S000002488	YDR081C	PDC2	607304	610081	Pyruvate DeCarboxylase
713304	T	C	56.04	snp	ChrIV						
788752	T	A	56.04	snp	ChrIV	S000002573	YDR166C	SEC5	786306	789221	SECretory
824703	G	A	56.157	snp	ChrIV	S000002588	YDR180W	SCC2	821295	825776	Sister Chromatid Cohesion
833018	A	G	56.157	snp	ChrIV	S000002594	YDR186C	SND1	832859	835492	Srp-iNDependent targeting
944412	T	C	56.028	snp	ChrIV	S000002648	YDR240C	SNU56	943674	945152	Small NUclear ribonucleoprotein associated
976431	TA	TGA	56.199	ins	ChrIV						
993574	C	A	56.105	snp	ChrIV	S000002670	YDR262W		993134	993952	
998894	GCAC	GC	56.279	del	ChrIV	S000002673	YDR265W	PEX10	998864	999877	PEroXin
1038200	C	T	56.04	snp	ChrIV						
1048036	C	A	56.199	snp	ChrIV	S000002701	YDR293C	SSD1	1045640	1049392	Suppressor of SIT4 Deletion
1068434	A	T	56.04	snp	ChrIV						
1129836	G	T	56.199	snp	ChrIV	S000002739	YDR331W	GPI8	1129588	1130823	GlycosylPhosphatidylInositol anchor biosynthesis
1147142	G	T	56.157	snp	ChrIV	S000002745	YDR337W	MRPS28	1146319	1147179	Mitochondrial Ribosomal Protein, Small subunit
1274439	C	A	56.279	snp	ChrIV						
1290378	C	A	56.04	snp	ChrIV	S000002817	YDR409W	SIZ1	1289406	1292120	SAP and mIZ-finger domain
1293230	T	A	56.199	snp	ChrIV						
1395066	G	A	56.178	snp	ChrIV						
1404189	T	A	56.157	snp	ChrIV						
1427522	A	G	56.199	snp	ChrIV	S000002894	YDR486C	VPS60	1427431	1428120	Vacuolar Protein Sorting
1486923	A	T	56.199	snp	ChrIV	S000002931	YDR523C	SPS1	1485566	1487038	SPorulation Specific
47287	A	T	56.199	snp	ChrIX	S000001419	YIL157C	COA1	46949	47542	Cytochrome Oxidase Assembly
47504	C	A	56.105	snp	ChrIX	S000001419	YIL157C	COA1	46949	47542	Cytochrome Oxidase Assembly
47504	C	A	56.105	snp	ChrIX	S000028797	YIL156W-A		47292	47693	
55412	G	T	56.199	snp	ChrIX	S000001415	YIL153W	RRD1	55198	56379	Resistant to Rapamycin Deletion
65223	C	T	56.199	snp	ChrIX	S000001411	YIL149C	MLP2	63028	68067	Myosin-Like Protein
78776	T	A	56.04	snp	ChrIX	S000001406	YIL144W	NDC80	78074	80149	Nuclear Division Cycle
83293	A	G	56.157	snp	ChrIX	S000028796	YIL142C-A		83208	83540	

142636	C	A	56.04	snp	ChrIX						
145011	C	A	56.072	snp	ChrIX	S000001377	YIL115C	NUP159	144327	148709	NUclear Pore
181460	A	G	56.04	snp	ChrIX	S000001359	YIL097W	FYV10	180427	181977	Function required for Yeast Viability
255048	T	A	56.157	snp	ChrIX						
278005	A	G	56.199	snp	ChrIX	S000001302	YIL040W	APQ12	277723	278139	APical growth revealed by Quantitative morphological analysis
285310	A	G	56.157	snp	ChrIX						
294730	G	C	56.279	snp	ChrIX	S000001293	YIL031W	ULP2	292633	295737	UbL-specific Protease
310056	A	T	56.105	snp	ChrIX	S000001285	YIL023C	YKE4	309386	310426	Yeast ortholog of mouse KE4
404285	T	C	56.04	snp	ChrIX	S000001464	YIR025W	MND2	403659	404765	Meiotic Nuclear Divisions
410457	T	A	56.04	snp	ChrIX						
80965	C	G	56.105	snp	ChrV	S000000764	YEL038W	UTR4	80462	81145	Unidentified TRanscript
83912	A	T	56.199	snp	ChrV	S000000762	YEL036C	ANP1	83050	84552	ANP and osmotic sensitive
121907	C	A	56.199	snp	ChrV	S000000744	YEL018W	EAF5	121471	122310	Esa1p-Associated Factor
121907	C	A	56.199	snp	ChrV	S000028742	YEL018C-A		121455	121988	
121949	C	T	56.199	snp	ChrV	S000000744	YEL018W	EAF5	121471	122310	Esa1p-Associated Factor
121949	C	T	56.199	snp	ChrV	S000028742	YEL018C-A		121455	121988	
176929	C	A	56.31	snp	ChrV						
203014	T	A	56.04	snp	ChrV	S000000826	YER024W	YAT2	202192	204963	
206574	G	C	56.279	snp	ChrV	S000000827	YER025W	GCD11	205251	206834	General Control Derexpressed
235331	C	A	56.279	snp	ChrV	S000000844	YER042W	MXR1	234937	235491	peptide Methionine sulfoXide Reductase
237771	C	T	56.279	snp	ChrV	S000000846	YER044C	ERG28	237570	238016	ERGosterol biosynthesis
245380	A	T	56.04	snp	ChrV	S000000849	YER047C	SAP1	243810	246503	Sin1 Associated Protein
257457	C	A	56.04	snp	ChrV	S000000854	YER052C	HOM3	256375	257958	HOMoserine requiring
333628	C	A	56.04	snp	ChrV	S000000890	YER088C	DOT6	333176	335188	Disruptor Of Telomeric silencing
348418	A	T	56.04	snp	ChrV						
543577	C	T	56.04	snp	ChrV	S000000978	YER176W	ECM32	541690	545055	ExtraCellular Mutant
103496	C	T	56.199	snp	ChrVI						
229293	C	A	56.04	snp	ChrVI						
256815	A	T	56.199	snp	ChrVI						
60547	GA	TG	56.157	mnp	ChrVII	S000003202	YGL233W	SEC15	59122	61854	SECretory
92677	A	G	56.105	snp	ChrVII	S000003179	YGL211W	NCS6	92512	93591	Needs Cla4 to Survive
137281	A	G	56.279	snp	ChrVII	S000003163	YGL195W	GCN1	131525	139543	General Control Nonderepressible

256342	G	T	56.279	snp	ChrVII	S000003102	YGL134W	PCL10	255663	256964	Pho85 CycLin
279507	C	G	56.199	snp	ChrVII	S000003090	YGL122C	NAB2	278946	280523	Nuclear polyAdenylated RNA-Binding
347292	ACA	AA	56.04	del	ChrVII	S000003054	YGL086W	MAD1	347119	349368	Mitotic Arrest-Deficient
366615	G	T	56.04	snp	ChrVII						
446241	C	A	56.279	snp	ChrVII						
504333	C	A	56.105	snp	ChrVII						
507560	G	T	56.279	snp	ChrVII	S000003239	YGR007W	ECT1	506969	507940	Ethanolamine-phosphate CytidylylTransferase
550845	T	A	56.04	snp	ChrVII	S000003264	YGR032W	GSC2	548264	553951	Glucan Synthase of Cerevisiae
602427	A	G	56.279	snp	ChrVII	S000003288	YGR056W	RSC1	601661	604447	Remodel the Structure of Chromatin
654647	G	C	56.04	snp	ChrVII	S000003320	YGR088W	CTT1	654634	656322	CaTalase T
657377	A	G	56.04	snp	ChrVII	S000003321	YGR089W	NNF2	656960	659770	
671743	A	T	56.028	snp	ChrVII	S000003325	YGR093W	DRN1	670388	671911	Debranching enzyme-associated RiboNuclease
829227	C	A	56.199	snp	ChrVII	S000003397	YGR165W	MRPS35	829116	830153	Mitochondrial Ribosomal Protein, Small subunit
847714	A	T	56.199	snp	ChrVII	S000003407	YGR175C	ERG1	846933	848423	ERGosterol biosynthesis
855552	G	A	56.199	snp	ChrVII	S000003412	YGR180C	RNR4	855264	856301	RiboNucleotide Reductase
889271	GCA	GA	56.199	del	ChrVII	S000003427	YGR195W	SKI6	888882	889622	SuperKIller
1037222	A	G	56.157	snp	ChrVII	S000003503	YGR271W	SLH1	1031791	1037694	SKI2-Like Helicase
1074776	T	C	56.157	snp	ChrVII	S000003521	YGR289C	MAL11	1073963	1075813	MALtose fermentation
65794	T	C	56.105	snp	ChrVIII	S000001013	YHL021C	AIM17	64462	65859	Altered Inheritance rate of Mitochondria
66166	G	T	56.157	snp	ChrVIII						
70173	A	G	56.04	snp	ChrVIII						
114061	T	A	56.199	snp	ChrVIII	S000001047	YHR005C	GPA1	113499	114917	G Protein Alpha subunit
126035	G	T	56.279	snp	ChrVIII						
126066	CAC	CG	56.105	complex	ChrVIII						
129120	A	T	56.157	snp	ChrVIII	S000001053	YHR011W	DIA4	127780	129120	Digs Into Agar
148648	T	C	56.258	snp	ChrVIII	S000001063	YHR021C	RPS27B	147871	148669	Ribosomal Protein of the Small subunit
263998	TAA	TA	56.232	del	ChrVIII	S000001122	YHR080C	LAM4	262801	266838	Lipid transfer protein Anchored at Membrane contact sites

316235	C	T	56.04	snp	ChrVIII						
318313	C	T	56.199	snp	ChrVIII	S000001144	YHR102W	KIC1	316572	319814	Kinase that Interacts with Cdc31p
352410	T	C	56.199	snp	ChrVIII	S000001162	YHR120W	MSH1	349574	352453	MutS Homolog
384444	GTT	GT	56.157	del	ChrVIII	S000001184	YHR142W	CHS7	383538	384488	CHitin Synthase-related
392885	T	C	56.04	snp	ChrVIII	S000001190	YHR147C	MRPL6	392639	393283	Mitochondrial Ribosomal Protein, Large subunit
421525	G	T	56.072	snp	ChrVIII	S000001204	YHR161C	YAP1801	420373	422286	Yeast Assembly Polypeptide
443788	ATT	AT	56.04	del	ChrVIII						
27792	G	T	56.04	snp	ChrX	S000003750	YJL214W	HXT8	26887	28596	HeXose Transporter
39033	T	A	56.157	snp	ChrX	S000003745	YJL209W	CBP1	38005	39969	Cytochrome B mRNA Processing
46474	G	T	56.04	snp	ChrX	S000003743	YJL207C	LAA1	41389	47433	Large AP-1 Accessory
54596	T	A	56.105	snp	ChrX	S000003737	YJL201W	ECM25	54379	56178	ExtraCellular Mutant
147594	T	A	56.157	snp	ChrX						
460619	T	C	56.199	snp	ChrX	S000003774	YJR013W	GPI14	460382	461593	GlycosylPhosphatidylInositol anchor biosynthesis
469691	GAAAAAAAAAAAC	GAAAAAAAAAAC	56.04	del	ChrX						
498981	ACA	AA	56.279	del	ChrX	S000003796	YJR035W	RAD26	497355	500612	RADiation sensitive
515098	G	A	56.157	snp	ChrX	S000003803	YJR042W	NUP85	514055	516289	NUclear Pore
537999	A	G	56.232	snp	ChrX						
572142	T	C	56.157	snp	ChrX						
581903	G	A	56.105	snp	ChrX						
652028	T	C	56.279	snp	ChrX	S000003884	YJR123W	RPS5	651901	652578	Ribosomal Protein of the Small subunit
687471	G	T	56.04	snp	ChrX	S000003899	YJR138W	IML1	684567	689321	Increased MinichrLoss
725149	G	T	56.072	snp	ChrX						
5045	T	A	56.04	snp	ChrXI	S000001705	YKL222C		3503	5620	
55227	TAAAAAT	TAAAAT	56.04	del	ChrXI	S000001687	YKL204W	EAP1	53704	55602	EIF4E-Associated Protein
55921	T	A	56.04	snp	ChrXI						
61539	C	A	56.178	snp	ChrXI	S000001686	YKL203C	TOR2	55935	63359	Target Of Rapamycin
79230	ATTTTTTTTA	ATTTTTTTA	56.04	del	ChrXI	S000001676	YKL193C	SDS22	78866	79882	homolog of S. pombe SDS22
118678	T	A	56.04	snp	ChrXI						
158260	C	G	56.157	snp	ChrXI						
185452	G	T	56.04	snp	ChrXI	S000001621	YKL138C	MRPL31	185286	185681	Mitochondrial Ribosomal Protein, Large subunit

188006	C	A	56.04	snp	ChrXI	S000001618	YKL135C	APL2	186460	188640	clathrin Adaptor Protein complex Large chain
240837	T	A	56.199	snp	ChrXI	S000001588	YKL105C	SEG2	239185	242583	Stability of Eisosomes Guaranteed
243766	T	A	56.04	snp	ChrXI	S000001587	YKL104C	GFA1	243220	245373	Glutamine:Fructose-6-phosphate Amidotransferase
249206	G	A	56.279	snp	ChrXI	S000001584	YKL101W	HSL1	248920	253476	Histone Synthetic Lethal
306678	A	G	56.157	snp	ChrXI	S000001553	YKL070W		306211	306720	
310839	C	A	56.119	snp	ChrXI	S000001551	YKL068W	NUP100	310199	313078	NUclear Pore
321201	C	A	56.279	snp	ChrXI	S000001546	YKL063C		321015	321518	
329634	C	T	56.04	snp	ChrXI						
404060	GC	AA	56.258	mnp	ChrXI						
411408	G	A	56.199	snp	ChrXI	S000001498	YKL015W	PUT3	408544	411483	Proline UTilization
465529	C	A	56.04	snp	ChrXI	S000001722	YKR014C	YPT52	465367	466071	Yeast Protein Two
537932	T	C	56.199	snp	ChrXI	S000001762	YKR054C	DYN1	535647	547925	DYNein
563328	G	A	56.04	snp	ChrXI	S000001772	YKR064W	OAF3	562547	565138	Oleate Activated transcription Factor
593433	G	T	56.279	snp	ChrXI	S000001790	YKR082W	NUP133	592825	596298	NUclear Pore
616140	T	A	56.04	snp	ChrXI	S000001801	YKR093W	PTR2	615730	617535	Peptide TRansport
637495	T	C	56.04	snp	ChrXI	S000001807	YKR099W	BAS1	635851	638286	BASal
36423	A	G	56.199	snp	ChrXII						
49852	C	T	56.04	snp	ChrXII	RegiÃ³n intergÃ©nica		FPS1			
52672	C	T	56.04	snp	ChrXII	RegiÃ³n intergÃ©nica		En realidad no afecta a FPS1. Parece que afecta el promotor de ATG10			
110889	G	A	56.199	snp	ChrXII	S000003941	YLL018C	DPS1	109902	111575	
286355	G	C	56.199	snp	ChrXII	S000004068	YLR078C	BOS1	285736	286559	Bet One Suppressor
322476	A	G	56.279	snp	ChrXII	S000004081	YLR091W	GEP5	322297	323178	GEnetic interactors of Prohibitins
434291	TGTGT	TTT	56.04	complex	ChrXII	S000004137	YLR147C	SMD3	434158	434463	
460002	CAAAAAAAAAAAAAAAAC	CAAAAAAAAC	56.296	del	ChrXII						
460103	T	C	56.157	snp	ChrXII						
460103	T	C	56.157	snp	ChrXII						

460103	T	C	56.258	snp	ChrXII						
460107	T	A	56.098	snp	ChrXII						
460152	T	G	56.157	snp	ChrXII						
460152	T	G	56.04	snp	ChrXII						
460262	G	T	109.38	snp	ChrXII						
460450	T	C	56.232	snp	ChrXII						
460477	ATTTTTTTT C	ATTTTTTTC	56.157	del	ChrXII						
460569	T	C	56.258	snp	ChrXII						
552655	C	G	56.04	snp	ChrXII						
611250	C	A	56.105	snp	ChrXII	S000004224	YLR234W	TOP3	609783	611753	TOPoisomerase
638577	T	C	56.269	snp	ChrXII	S000004239	YLR249W	YEF3	636780	639914	Yeast Elongation Factor
698529	T	A	56.098	snp	ChrXII	S000004267	YLR277C	YSH1	697156	699495	Yeast Seventy-three Homolog
745217	C	T	56.279	snp	ChrXII						
871065	T	C	56.04	snp	ChrXII	S000004365	YLR373C	VID22	868662	871367	Vacuolar Import and Degradation
971293	A	T	56.279	snp	ChrXII	S000004414	YLR422W	DCK1	965897	971695	DoCK1 homolog
1020309	A	T	56.098	snp	ChrXII	S000004434	YLR442C	SIR3	101931 5	102225 1	Silent Information Regulator
1039435	T	A	56.199	snp	ChrXII	S000004444	YLR452C	SST2	103927 0	104136 6	SuperSensiTive
11956	A	T	56.279	snp	ChrXIII	S000004599	YML130C	ERO1	11483	13174	ER Oxidation or Endoplasmic Reticulum Oxidoreductin
14763	C	T	56.04	snp	ChrXIII						
16515	G	T	56.199	snp	ChrXIII	S000004597	YML128C	MSC1	15135	16676	Meiotic Sister-Chromatid recombination
24641	A	T	56.199	snp	ChrXIII	S000004592	YML123C	PHO84	24037	25800	PHOsphate metabolism
110410	C	T	56.199	snp	ChrXIII	S000004544	YML079W		110247	110852	
123492	G	T	56.199	snp	ChrXIII	S000004538	YML073C	RPL6A	123227	124172	Ribosomal Protein of the Large subunit
123492	G	T	56.199	snp	ChrXIII	S000004538	YML073C	RPL6A	123227	124172	Ribosomal Protein of the Large subunit
166646	C	A	56.04	snp	ChrXIII	S000004518	YML054C	CYB2	165533	167308	CYtochrome B
179071	T	A	56.105	snp	ChrXIII	S000004511	YML048W	GSF2	178426	179637	Glucose Signaling Factor
179179	A	G	56.199	snp	ChrXIII	S000004511	YML048W	GSF2	178426	179637	Glucose Signaling Factor
214916	A	T	56.199	snp	ChrXIII	S000004493	YML031W	NDC1	214189	216156	Nuclear Division Cycle
236333	A	G	56.04	snp	ChrXIII						
252842	C	T	56.04	snp	ChrXIII	S000004467	YML008C	ERG6	251839	252990	ERGosterol biosynthesis

295178	G	A	56.04	snp	ChrXIII						
386251	C	A	56.031	snp	ChrXIII	S000004659	YMR055C	BUB2	386101	387021	Budding Uninhibited by Benzimidazole
418162	G	T	56.04	snp	ChrXIII	S000004681	YMR076C	PDS5	416196	420029	Precocious Dissociation of Sisters
484455	C	T	56.199	snp	ChrXIII	S000004714	YMR108W	ILV2	484084	486147	IsoLeucine-plus-Valine requiring
544359	G	T	56.279	snp	ChrXIII	S000004745	YMR137C	PSO2	542978	544963	PSOralen derivative sensitive
549500	C	A	56.199	snp	ChrXIII						
549500	C	A	56.152	snp	ChrXIII						
730503	G	T	56.199	snp	ChrXIII	S000004842	YMR229C	RRP5	725934	731123	Ribosomal RNA Processing
744079	A	G	56.279	snp	ChrXIII	S000004850	YMR237W	BCH1	743749	745923	Bud7 and Chs6 Homolog
774017	ATTTTTTTTTTTTC	ATTTTTTTTTTC	56.232	del	ChrXIII						
774560	G	T	56.279	snp	ChrXIII						
809198	A	G	56.279	snp	ChrXIII						
815689	T	A	56.04	snp	ChrXIII	S000004888	YMR275C	BUL1	815651	818581	Binds Ubiquitin Ligase
828781	T	C	56.152	snp	ChrXIII	S000004893	YMR280C	CAT8	827028	831329	CATabolite repression
837033	G	T	56.157	snp	ChrXIII						
907672	A	T	56.279	snp	ChrXIII	S000004936	YMR317W		907364	910786	
912699	A	T	56.279	snp	ChrXIII						
915529	G	C	56.152	snp	ChrXIII						
73523	C	T	56.279	snp	ChrXIV	S000005241	YNL297C	MON2	71673	76583	MONensin sensitivity
118252	C	G	56.105	snp	ChrXIV	S000005221	YNL277W	MET2	117349	118809	METhionine requiring
179374	G	T	56.279	snp	ChrXIV						
198347	C	T	56.199	snp	ChrXIV						
307695	G	A	56.04	snp	ChrXIV	S000005119	YNL175C	NOP13	307401	308612	NucleOlar Protein
317559	A	T	56.199	snp	ChrXIV	S000005113	YNL169C	PSD1	316169	317671	PhosphatidylSerine Decarboxylase
542913	A	T	56.258	snp	ChrXIV						
626100	T	C	56.04	snp	ChrXIV						
633082	T	A	56.279	snp	ChrXIV	S000005285	YNR002C	ATO2	633008	633856	Ammonia (Ammonium) Transport Outward
649833	A	G	56.157	snp	ChrXIV	S000005296	YNR013C	PHO91	649028	651712	PHOsphate metabolism
649833	A	G	56.178	snp	ChrXIV	S000005296	YNR013C	PHO91	649028	651712	PHOsphate metabolism
668267	A	T	56.157	snp	ChrXIV						
674780	ATTTTTTTTTTTTC	ATTTTTTTTTTC	56.232	del	ChrXIV						
38065	ATA	AA	56.199	del	ChrXV						

66022	A	T	56.098	snp	ChrXV	S000005497	YOL137W	BSC6	65621	67114	Bypass of Stop Codon
117730	A	G	56.157	snp	ChrXV						
132631	A	G	56.157	snp	ChrXV						
259985	G	A	56.279	snp	ChrXV	S000005394	YOL034W	SMC5	259923	263204	Structural Maintenance of Chromosomes
280586	C	A	56.199	snp	ChrXV	S000005382	YOL022C	TSR4	280272	281498	Twenty S rRNA accumulation
339211	T	A	56.04	snp	ChrXV	S000005533	YOR007C	SGT2	338938	339978	Small Glutamine-rich Tetratricopeptide repeat-containing protein
345434	G	C	56.199	snp	ChrXV	S000005535	YOR009W	TIR4	344335	345798	TIp1-Related
357715	C	A	56.178	snp	ChrXV	S000005540	YOR014W	RTS1	357674	359947	Rox Three Suppressor
444785	A	T	56.199	snp	ChrXV	S000005589	YOR063W	RPL3	444686	445849	Ribosomal Protein of the Large subunit
532710	ATT	AT	56.232	del	ChrXV	S000005638	YOR112W	CEX1	531508	533793	Cytoplasmic EXport protein
545623	T	A	56.279	snp	ChrXV	S000005643	YOR117W	RPT5	545029	546333	Regulatory Particle Triple-A protein, or Regulatory Particle Triphosphatase
609059	G	A	56.199	snp	ChrXV	S000005674	YOR148C	SPP2	608640	609197	Suppressor of PrP
613254	C	T	56.105	snp	ChrXV	S000005677	YOR151C	RPB2	612997	616671	RNA Polymerase B
634879	T	A	56.157	snp	ChrXV	S000005686	YOR160W	MTR10	633839	636757	Mrna TRansport defective
669887	C	A	56.199	snp	ChrXV	S000005704	YOR178C	GAC1	667860	670241	Glycogen ACcumulation
733640	G	A	56.199	snp	ChrXV						
754666	G	T	56.105	snp	ChrXV	S000005745	YOR219C	STE13	752214	755009	STErile
775808	ATT	AT	56.105	del	ChrXV						
781758	G	T	56.105	snp	ChrXV						
838537	A	T	56.157	snp	ChrXV	S000005800	YOR274W	MOD5	837674	838960	tRNA MODification
851650	C	A	56.157	snp	ChrXV	S000005813	YOR287C	RRP36	850937	851839	Ribosomal RNA Processing
907148	A	G	56.105	snp	ChrXV	S000005843	YOR316C	COT1	906236	907555	CObalt Toxicity
911791	A	T	56.199	snp	ChrXV	S000005845	YOR318C		911784	912436	
914454	G	C	56.157	snp	ChrXV	S000005847	YOR320C	GNT1	913619	915094	GlcNAc Transferase
962946	G	T	56.157	snp	ChrXV	S000005868	YOR341W	RPA190	960987	965981	RNA Polymerase A
1005364	C	A	56.105	snp	ChrXV	S000005882	YOR355W	GDS1	1005137	1006705	
41159	G	T	56.279	snp	ChrXVI	S000006186	YPL265W	DIP5	41043	42869	DIcarboxylic amino acid Permease
145489	A	T	56.04	snp	ChrXVI	S000006137	YPL216W		143821	147129	
166363	G	A	56.199	snp	ChrXVI	S000006124	YPL203W	TPK2	166256	167398	Takashi's Protein Kinase

185326	T	C	56.279	snp	ChrXVI	S000006111	YPL190C	NAB3	185317	187725	Nuclear polyAdenylated RNA-Binding
204241	G	T	56.04	snp	ChrXVI	S000006102	YPL181W	CTI6	203421	204941	Cyc8-Tup1 Interacting protein
288555	G	T	56.199	snp	ChrXVI	S000006061	YPL140C	MKK2	287514	289034	Mitogen-activated Kinase Kinase
291665	T	C	56.157	snp	ChrXVI	S000006059	YPL138C	SPP1	291366	292427	Set1c, Phd finger Protein
323046	A	G	56.105	snp	ChrXVI	S000006041	YPL120W	VPS30	322071	323744	Vacuolar Protein Sorting
410368	T	A	56.279	snp	ChrXVI						
584999	A	T	56.199	snp	ChrXVI	S000006217	YPR013C	CMR3	584632	585585	Changed Mutation Rate
599928	C	T	56.04	snp	ChrXVI	S000006224	YPR020W	ATP20	599870	600217	ATP synthase
604923	G	T	56.157	snp	ChrXVI	S000006226	YPR022C	SDD4	603911	607312	Suppressor of Degenerative Death
639208	T	C	56.098	snp	ChrXVI						
679448	GTTTTTTTTTTTTC	GTTTTTTTTTTTTTC	56.296	ins	ChrXVI						
694348	A	G	56.178	snp	ChrXVI	S000006278	YPR074C	TKL1	692796	694838	TransKetoLase
711293	CTTTTTTTTTTTA	CTTTTTTTTTTA	56.105	del	ChrXVI						
776439	A	C	56.04	snp	ChrXVI						
804602	G	T	56.279	snp	ChrXVI						
804613	C	T	56.279	snp	ChrXVI						
818699	G	T	56.279	snp	ChrXVI	S000006347	YPR143W	RRP15	818323	819075	Ribosomal RNA Processing
839494	G	T	56.199	snp	ChrXVI	S000006360	YPR156C	TPO3	837909	839777	Transporter of POlyamines
844021	G	T	56.279	snp	ChrXVI						
866667	A	G	56.279	snp	ChrXVI						
7525	A	G	56.279	snp	mitochondrion MT						
14184	A	G	56.04	snp	mitochondrion MT	S000007260	Q0045	COX1	13818	26701	Cytochrome c OXidase
14184	A	G	56.04	snp	mitochondrion MT	S000007261	Q0050	AI1	13818	16322	
14184	A	G	56.04	snp	mitochondrion MT	S000007262	Q0055	AI2	13818	18830	
14184	A	G	56.04	snp	mitochondrion MT	S000007263	Q0060	AI3	13818	19996	
14184	A	G	56.04	snp	mitochondrion MT	S000007264	Q0065	AI4	13818	21935	
14184	A	G	56.04	snp	mitochondrion MT	S000007265	Q0070	AI5_ALPHA	13818	23167	

14841	A	G	56.157	snp	mitochondrion MT	S000007260	Q0045	COX1	13818	26701	Cytochrome c OXidase
14841	A	G	56.157	snp	mitochondrion MT	S000007261	Q0050	AI1	13818	16322	
14841	A	G	56.157	snp	mitochondrion MT	S000007262	Q0055	AI2	13818	18830	
14841	A	G	56.157	snp	mitochondrion MT	S000007263	Q0060	AI3	13818	19996	
14841	A	G	56.157	snp	mitochondrion MT	S000007264	Q0065	AI4	13818	21935	
14841	A	G	56.157	snp	mitochondrion MT	S000007265	Q0070	AI5_ALPHA	13818	23167	
15410	A	G	56.157	snp	mitochondrion MT	S000007260	Q0045	COX1	13818	26701	Cytochrome c OXidase
15410	A	G	56.157	snp	mitochondrion MT	S000007261	Q0050	AI1	13818	16322	
15410	A	G	56.157	snp	mitochondrion MT	S000007262	Q0055	AI2	13818	18830	
15410	A	G	56.157	snp	mitochondrion MT	S000007263	Q0060	AI3	13818	19996	
15410	A	G	56.157	snp	mitochondrion MT	S000007264	Q0065	AI4	13818	21935	
15410	A	G	56.157	snp	mitochondrion MT	S000007265	Q0070	AI5_ALPHA	13818	23167	
16621	C	T	56.199	snp	mitochondrion MT	S000007260	Q0045	COX1	13818	26701	Cytochrome c OXidase
16621	C	T	56.199	snp	mitochondrion MT	S000007262	Q0055	AI2	13818	18830	
16621	C	T	56.199	snp	mitochondrion MT	S000007263	Q0060	AI3	13818	19996	
16621	C	T	56.199	snp	mitochondrion MT	S000007264	Q0065	AI4	13818	21935	
16621	C	T	56.199	snp	mitochondrion MT	S000007265	Q0070	AI5_ALPHA	13818	23167	
18614	T	A	56.04	snp	mitochondrion MT	S000007260	Q0045	COX1	13818	26701	Cytochrome c OXidase
18614	T	A	56.04	snp	mitochondrion MT	S000007262	Q0055	AI2	13818	18830	
18614	T	A	56.04	snp	mitochondrion MT	S000007263	Q0060	AI3	13818	19996	
18614	T	A	56.04	snp	mitochondrion MT	S000007264	Q0065	AI4	13818	21935	
18614	T	A	56.04	snp	mitochondrion MT	S000007265	Q0070	AI5_ALPHA	13818	23167	
20139	G	A	56.157	snp	mitochondrion MT	S000007260	Q0045	COX1	13818	26701	Cytochrome c OXidase

20139	G	A	56.157	snp	mitochondrion MT	S000007264	Q0065	AI4	13818	21935	
20139	G	A	56.157	snp	mitochondrion MT	S000007265	Q0070	AI5_ALPHA	13818	23167	
20345	GAA	GA	56.232	del	mitochondrion MT	S000007260	Q0045	COX1	13818	26701	Cytochrome c OXidase
20345	GAA	GA	56.232	del	mitochondrion MT	S000007264	Q0065	AI4	13818	21935	
20345	GAA	GA	56.232	del	mitochondrion MT	S000007265	Q0070	AI5_ALPHA	13818	23167	
20588	G	A	56.269	snp	mitochondrion MT	S000007260	Q0045	COX1	13818	26701	Cytochrome c OXidase
20588	G	A	56.269	snp	mitochondrion MT	S000007264	Q0065	AI4	13818	21935	
20588	G	A	56.269	snp	mitochondrion MT	S000007265	Q0070	AI5_ALPHA	13818	23167	
20934	T	G	56.279	snp	mitochondrion MT	S000007260	Q0045	COX1	13818	26701	Cytochrome c OXidase
20934	T	G	56.279	snp	mitochondrion MT	S000007264	Q0065	AI4	13818	21935	
20934	T	G	56.279	snp	mitochondrion MT	S000007265	Q0070	AI5_ALPHA	13818	23167	
20934	T	G	56.04	snp	mitochondrion MT	S000007260	Q0045	COX1	13818	26701	Cytochrome c OXidase
20934	T	G	56.04	snp	mitochondrion MT	S000007264	Q0065	AI4	13818	21935	
20934	T	G	56.04	snp	mitochondrion MT	S000007265	Q0070	AI5_ALPHA	13818	23167	
20934	T	G	56.199	snp	mitochondrion MT	S000007260	Q0045	COX1	13818	26701	Cytochrome c OXidase
20934	T	G	56.199	snp	mitochondrion MT	S000007264	Q0065	AI4	13818	21935	
20934	T	G	56.199	snp	mitochondrion MT	S000007265	Q0070	AI5_ALPHA	13818	23167	
20934	T	G	56.157	snp	mitochondrion MT	S000007260	Q0045	COX1	13818	26701	Cytochrome c OXidase
20934	T	G	56.157	snp	mitochondrion MT	S000007264	Q0065	AI4	13818	21935	
20934	T	G	56.157	snp	mitochondrion MT	S000007265	Q0070	AI5_ALPHA	13818	23167	
23332	GTT	GT	56.178	del	mitochondrion MT	S000007260	Q0045	COX1	13818	26701	Cytochrome c OXidase
31122	AA	ACA	56.04	ins	mitochondrion MT						
31124	TAT	TT	56.04	del	mitochondrion MT						

37684	T	A	56.199	snp	mitochondrion MT	S000007270	Q0105	COB	36540	43647	CytochrOme B
37684	T	A	56.199	snp	mitochondrion MT	S000007271	Q0110	BI2	36540	38579	
37684	T	A	56.199	snp	mitochondrion MT	S000007272	Q0115	BI3	36540	40265	
37684	T	A	56.199	snp	mitochondrion MT	S000007273	Q0120	BI4	36540	42251	
39517	G	T	56.04	snp	mitochondrion MT	S000007270	Q0105	COB	36540	43647	CytochrOme B
39517	G	T	56.04	snp	mitochondrion MT	S000007272	Q0115	BI3	36540	40265	
39517	G	T	56.04	snp	mitochondrion MT	S000007273	Q0120	BI4	36540	42251	
39517	G	T	56.157	snp	mitochondrion MT	S000007270	Q0105	COB	36540	43647	CytochrOme B
39517	G	T	56.157	snp	mitochondrion MT	S000007272	Q0115	BI3	36540	40265	
39517	G	T	56.157	snp	mitochondrion MT	S000007273	Q0120	BI4	36540	42251	
39517	G	T	56.157	snp	mitochondrion MT	S000007270	Q0105	COB	36540	43647	CytochrOme B
39517	G	T	56.157	snp	mitochondrion MT	S000007272	Q0115	BI3	36540	40265	
39517	G	T	56.157	snp	mitochondrion MT	S000007273	Q0120	BI4	36540	42251	
39517	G	T	56.157	snp	mitochondrion MT	S000007270	Q0105	COB	36540	43647	CytochrOme B
39517	G	T	56.157	snp	mitochondrion MT	S000007272	Q0115	BI3	36540	40265	
39517	G	T	56.157	snp	mitochondrion MT	S000007273	Q0120	BI4	36540	42251	
39517	G	T	56.157	snp	mitochondrion MT	S000007270	Q0105	COB	36540	43647	CytochrOme B
39517	G	T	56.157	snp	mitochondrion MT	S000007272	Q0115	BI3	36540	40265	
39517	G	T	56.157	snp	mitochondrion MT	S000007273	Q0120	BI4	36540	42251	
41771	T	A	56.157	snp	mitochondrion MT	S000007270	Q0105	COB	36540	43647	CytochrOme B
41771	T	A	56.157	snp	mitochondrion MT	S000007273	Q0120	BI4	36540	42251	
42031	C	G	56.031	snp	mitochondrion MT	S000007270	Q0105	COB	36540	43647	CytochrOme B
42031	C	G	56.031	snp	mitochondrion MT	S000007273	Q0120	BI4	36540	42251	

59999	G	T	56.157	snp	mitochondrion MT						
61255	A	T	56.04	snp	mitochondrion MT	S000007279	Q0160	SCEI	61022	61729	
61988	T	A	56.199	snp	mitochondrion MT						
62039	G	T	56.04	snp	mitochondrion MT						
67492	T	C	56.04	snp	mitochondrion MT						
72687	TCA	TA	56.04	del	mitochondrion MT						
74016	ATTTTTC	ATTTTC	56.105	del	mitochondrion MT	S000007281	Q0250	COX2	73758	74513	Cytochrome c OXidase
74107	T	C	56.04	snp	mitochondrion MT	S000007281	Q0250	COX2	73758	74513	Cytochrome c OXidase
78559	T	C	56.199	snp	mitochondrion MT						
79313	C	A	56.157	snp	mitochondrion MT	S000007283	Q0275	COX3	79213	80022	Cytochrome c OXidase
80764	T	C	56.105	snp	mitochondrion MT						

TAT12

POS	REF	ALT	QUAL	TYPE	Cromosome	DB identifier	GeneSysName	GeneStdName	Start	End	Protein name
27126	TTCC	CTCG	55.7364	complex	Chromosome I	S000000059	YAL063C	FLO9	24000	27968	FLOcculation
56545	T	A	54.1002	snp	Chromosome I	S000000045	YAL047C	SPC72	54989	56857	Spindle Pole Component
75181	A	G	55.9635	snp	Chromosome I	S000000034	YAL036C	RBG1	75043	76152	RiBosome interacting Gtpase
76300	T	C	56.1101	snp	Chromosome I	intergenic					
78459	TACTAACA	TA	55.1533	del	Chromosome I	S000000033	YAL035W	FUN12	76427	79435	Function Unknown Now
118532	T	A	54.1002	snp	Chromosome I	intergenic					
137680	T	A	55.5807	snp	Chromosome I	intergenic					
141807	A	T	54.8661	snp	Chromosome I	intergenic					
145818	C	A	55.1533	snp	Chromosome I	S000000002	YAL002W	VPS8	143707	147531	Vacuolar Protein Sorting
15101	T	G	55.8622	snp	Chromosome II	S000000201	YBL105C	PKC1	14241	17696	Protein Kinase C

16392	A	G	54.1002	snp	Chromosome II	S000000201	YBL105C	PKC1	14241	17696	Protein Kinase C
22538	A	G	56.2849	snp	Chromosome II	S000000199	YBL103C	RTG3	22076	23536	ReTroGrade regulation
38968	GTTTTTTTTTTTC	GTTTTTTTTTTC	55.3887	del	Chromosome II	intergenic					
48832	A	G	55.8622	snp	Chromosome II	S000000186	YBL090W	MRP21	48825	49358	Mitochondrial Ribosomal Protein
52925	G	T	55.1533	snp	Chromosome II	S000000184	YBL088C	TEL1	51019	59382	TELomere maintenance
85200	T	C	54.1002	snp	Chromosome II	S000000171	YBL075C	SSA3	84499	86448	Stress-Seventy subfamily A
102344	C	A	54.5182	snp	Chromosome II	S000000159	YBL063W	KIP1	101886	105221	KInesin related Protein
111656	T	C	54.1002	snp	Chromosome II	S000000154	YBL058W	SHP1	111437	112708	Suppressor of High-copy PP1
123749	T	C	54.8661	snp	Chromosome II	S000000147	YBL051C	PIN4	122753	124759	Psi+ INducibility
147282	T	A	56.1101	snp	Chromosome II	S000000133	YBL037W	APL3	147209	150286	clathrin Adaptor Protein complex Large chain
164069	A	G	54.5182	snp	Chromosome II	intergenic					
175855	T	A	54.8661	snp	Chromosome II	S000000119	YBL023C	MCM2	174920	177526	MiniChromosome Maintenance
206773	T	C	55.9635	snp	Chromosome II	S000000106	YBL010C	LAA2	206107	206949	Large Adaptin Accessory
263246	A	G	55.9635	snp	Chromosome II	intergenic					
275419	CT	AG	54.1002	mnp	Chromosome II	S000000222	YBR018C	GAL7	274427	275527	GALactose metabolism
294580	C	G	54.6922	snp	Chromosome II	S000000232	YBR028C	YPK3	294425	296002	
303604	T	A	55.1533	snp	Chromosome II	S000000237	YBR033W	EDS1	301944	304703	Expression Dependent on Slt2
323212	G	T	54.5182	snp	Chromosome II	S000000247	YBR043C	QDR3	321876	323945	QuiniDine Resistance
323402	A	T	55.8622	snp	Chromosome II	S000000247	YBR043C	QDR3	321876	323945	QuiniDine Resistance
326322	A	G	54.8661	snp	Chromosome II	intergenic					
336005	T	C	54.1002	snp	Chromosome II	S000000253	YBR049C	REB1	334386	336818	RNA polymerase I Enhancer Binding protein
343321	TCA	TA	55.8906	del	Chromosome II	S000000258	YBR054W	YRO2	343101	344135	

366103	A	T	55.3887	snp	Chromosome II	S000000266	YBR062C		365976	366600	
380563	C	T	56.2849	snp	Chromosome II	S000000275	YBR071W		380411	381046	
381227	T	A	55.9635	snp	Chromosome II	intergenic					
389425	TCTTCGT	TTGCT	55.8906	complex	Chromosome II	intergenic					
389425	TCTTCGT	TTGCT	55.8906	complex	Chromosome II	intergenic					
415495	G	A	54.5182	snp	Chromosome II	intergenic					
434053	C	G	55.8622	snp	Chromosome II	S000000298	YBR094W	PBY1	432036	434297	P-BodY associated protein
453362	C	T	55.0097	snp	Chromosome II	intergenic					
474080	G	A	55.3887	snp	Chromosome II	intergenic					
492184	A	G	54.1002	snp	Chromosome II	S000000331	YBR127C	VMA2	491269	492822	Vacuolar Membrane Atpase
501131	C	A	55.5807	snp	Chromosome II	S000000336	YBR132C	AGP2	499652	501442	high-Affinity Glutamine Permease
537255	T	C	56.2849	snp	Chromosome II	S000000351	YBR147W	RTC2	536575	537465	Restriction of Telomere Capping
542285	C	A	55.1533	snp	Chromosome II	S000000354	YBR150C	TBS1	541209	544493	ThiaBendazole Sensitive
542347	G	A	54.5182	snp	Chromosome II	S000000354	YBR150C	TBS1	541209	544493	ThiaBendazole Sensitive
542859	C	T	54.5182	snp	Chromosome II	S000000354	YBR150C	TBS1	541209	544493	ThiaBendazole Sensitive
563303	A	G	56.2849	snp	Chromosome II	S000000366	YBR162C	TOS1	563203	564570	Target Of Sbf
582198	C	T	56.1101	snp	Chromosome II	intergenic					
586331	TAAAAAAAAAAAAG	TAAAAAAAAAAAG	54.3092	del	Chromosome II	S000000382	YBR178W		586071	586445	
597525	GAA	GA	55.3887	del	Chromosome II	S000000388	YBR184W		597363	598934	
638038	CTTT	CT	54.1002	del	Chromosome II	S000000412	YBR208C	DUR1,2	636703	642210	Degradation of URea
644325	GACTGTTAGCT	AACAGCTAGT	56.2849	complex	Chromosome II	intergenic					
644336	ACA	AA	56.2849	del	Chromosome II	intergenic					

667378	T	A	55.5807	snp	Chromosome II	S000000426	YBR222C	PCS60	666720	668351	Peroxisomal CoA-dependent Synthetase
676181	C	A	54.8661	snp	Chromosome II	S000000432	YBR228W	SLX1	675313	676227	Synthetic Lethal of unknown (X) function
692538	G	A	54.6922	snp	Chromosome II	S000000441	YBR237W	PRP5	691969	694518	Pre-mRNA Processing
701950	AG	AGCGGG	56.1623	ins	Chromosome II	intergenic					
737300	C	A	55.5807	snp	Chromosome II	S000000467	YBR263W	SHM1	736264	737736	Serine HydroxyMethyltransferase
745927	T	C	54.1002	snp	Chromosome II	S000000475	YBR271W	EFM2	744852	746111	Elongation Factor Methyltransferase
765699	G	T	56.1623	snp	Chromosome II	S000000485	YBR281C	DUG2	764970	767606	Deficient in Utilization of Glutathione
766163	G	T	56.2849	snp	Chromosome II	S000000485	YBR281C	DUG2	764970	767606	Deficient in Utilization of Glutathione
793310	G	T	55.3887	snp	Chromosome II	S000000499	YBR295W	PCA1	792849	796499	P-type Cation-transporting ATPase
800737	C	A	54.6922	snp	Chromosome II	S000000501	YBR297W	MAL33	800523	801929	MALtose
334	C	T	56.1101	snp	Chromosome III	intergenic					
3884	A	G	55.9635	snp	Chromosome III	intergenic					
14937	T	C	54.3092	snp	Chromosome III	intergenic					
38397	T	G	54.8661	snp	Chromosome III	S000000555	YCL050C	APA1	37836	38801	AP4A phosphorylase
43884	A	T	54.5182	snp	Chromosome III	S000000552	YCL047C	POF1	43661	44437	Promoter Of Filamentation
50923	C	T	55.7364	snp	Chromosome III	S000000545	YCL040W	GLK1	50838	52340	GLucoKinase
50923	C	T	55.7364	snp	Chromosome III	S000000547	YCL042W		50584	50943	
93991	CTGT	CT	55.3887	del	Chromosome III	S000000522	YCL017C	NFS1	92777	94270	NiFS-like
118936	C	A	56.204	snp	Chromosome III	S000000596	YCR003W	MRPL32	118620	119171	Mitochondrial Ribosomal Protein, Large subunit
121516	G	A	55.7364	snp	Chromosome III	S000000598	YCR005C	CIT2	120946	122328	CITrate synthase
126620	G	T	55.5807	snp	Chromosome III	S000000600	YCR007C		126011	126730	
159335	G	C	55.7364	snp	Chromosome III	S000000617	YCR023C		158538	160373	

184294	C	A	55.3887	snp	Chromosome III	S000000628	YCR032W	BPH1	179520	186023	Beige Protein Homolog
189014	A	G	55.8622	snp	Chromosome III	S000000629	YCR033W	SNT1	186489	190169	SaNT domains
189077	G	A	54.1002	snp	Chromosome III	S000000629	YCR033W	SNT1	186489	190169	SaNT domains
191697	A	T	55.9635	snp	Chromosome III	intergenic					
192744	G	A	55.7364	snp	Chromosome III	S000000631	YCR035C	RRP43	191834	193018	Ribosomal RNA Processing
197928	A	T	54.8661	snp	Chromosome III	S000000634	YCR038C	BUD5	197621	199549	BUD site selection
223857	C	A	54.8661	snp	Chromosome III	S000000655	YCR059C	YIH1	223454	224230	Yeast Impact Homolog
236420	C	T	56.2849	snp	Chromosome III	intergenic					
248061	T	A	55.8622	snp	Chromosome III	S000000671	YCR075C	ERS1	248033	248815	ERd Suppressor
263446	G	C	55.7364	snp	Chromosome III	S000000682	YCR086W	CSM1	263392	263964	Chromosome Segregation in Meiosis
276315	C	T	55.1533	snp	Chromosome III	S000000687	YCR091W	KIN82	274404	276566	protein KINase
280828	A	T	54.5182	snp	Chromosome III	S000000689	YCR093W	CDC39	280117	286443	Cell Division Cycle
282138	G	T	54.5182	snp	Chromosome III	S000000689	YCR093W	CDC39	280117	286443	Cell Division Cycle
282138	G	T	54.5182	snp	Chromosome III	S000000725	YCRX20C		282116	282427	
296006	C	A	55.1533	snp	Chromosome III	intergenic					
298398	TGT	TT	55.5807	del	Chromosome III	S000000695	YCR098C	GIT1	297049	298605	GlycerophosphoInosiTol
72402	A	T	55.7364	snp	Chromosome IV	S000002374	YDL215C	GDH2	70640	73918	Glutamate DeHydrogenase
95161	A	T	54.1002	snp	Chromosome IV	S000002363	YDL204W	RTN2	94605	95786	ReTiculoN-like
136057	C	T	55.5807	snp	Chromosome IV	S000002339	YDL180W		135896	137539	
137827	C	A	54.1002	snp	Chromosome IV	intergenic					
143221	T	A	54.8661	snp	Chromosome IV	S000002335	YDL176W	IPF1	142097	144223	Involved in actin Patch Formation
157139	C	T	54.6922	snp	Chromosome IV	S000002329	YDL170W	UGA3	156318	157904	Utilization of GAba
157848	G	T	56.1036	snp	Chromosome IV	S000002329	YDL170W	UGA3	156318	157904	Utilization of GAba

199799	A	T	54.5182	snp	Chromosome IV	intergenic						
208687	G	A	55.1533	snp	Chromosome IV	S000002299	YDL140C	RPO21	205360	210561	RNA POlymerase
225574	C	A	55.1533	snp	Chromosome IV	S000002290	YDL132W	CDC53	224304	226751	Cell Division Cycle
245201	T	C	55.8622	snp	Chromosome IV	S000002279	YDL121C	EXP1	245133	245582	ER eXport of Pma1
312027	A	G	55.5807	snp	Chromosome IV	S000002238	YDL080C	THI3	310642	312471	THIamine metabolism
313358	C	A	55.8622	snp	Chromosome IV	S000002237	YDL079C	MRK1	312951	314748	Mds1p Related Kinase
345012	C	A	55.3887	snp	Chromosome IV	intergenic					
348806	T	C	54.5182	snp	Chromosome IV	S000002216	YDL058W	USO1	345665	351037	yUSOu - transport in Japanese
355165	A	G	54.1002	snp	Chromosome IV	S000002214	YDL056W	MBP1	352877	355378	MluI-box Binding Protein
425957	TAA	TA	54.8661	del	Chromosome IV	intergenic					
450550	A	G	54.3092	snp	Chromosome IV	S000002408	YDR001C	NTH1	450220	452475	Neutral TreHalase
455132	G	C	54.8661	snp	Chromosome IV	intergenic					
460893	C	T	55.7364	snp	Chromosome IV	S000002413	YDR006C	SOK1	458542	461247	Suppressor Of Kinase
550013	C	A	55.9635	snp	Chromosome IV	S000002453	YDR046C	BAP3	548762	550576	Branched-chain Amino acid Permease
553655	C	G	55.8622	snp	Chromosome IV	S000002456	YDR049W	VMS1	553254	555152	VCP/Cdc48-associated Mitochondrial Stress-responsive
553953	G	T	55.9635	snp	Chromosome IV	S000002456	YDR049W	VMS1	553254	555152	VCP/Cdc48-associated Mitochondrial Stress-responsive
562733	A	G	55.3887	snp	Chromosome IV	intergenic					
596728	C	T	56.2849	snp	Chromosome IV	intergenic		TPS2			Trehalose-6-Phosphate Synthase/phosphatase
616148	C	A	54.1002	snp	Chromosome IV	S000002492	YDR085C	AFR1	614288	616150	Alpha-Factor Receptor regulator
665653	G	T	55.0097	snp	Chromosome IV	S000002512	YDR105C	TMS1	665349	666770	
723744	C	A	55.0097	snp	Chromosome IV	S000002542	YDR135C	YCF1	723004	727551	Yeast Cadmium Factor

739753	G	A	54.1002	snp	Chromosome IV	S000002548	YDR141C	DOP1	734901	739997	homolog of A. nidulans DOPey
757295	G	T	55.8622	snp	Chromosome IV	S000002557	YDR150W	NUM1	755628	763874	NUclear Migration
784892	C	T	56.1623	snp	Chromosome IV	S000002572	YDR165W	TRM82	784871	786205	Transfer RNA Methyltransferase
794404	G	A	54.5182	snp	Chromosome IV	intergenic					
813229	T	C	55.5807	snp	Chromosome IV	S000002582	YDR175C	RSM24	813193	814152	Ribosomal Small subunit of Mitochondria
819881	A	G	55.5807	snp	Chromosome IV	S000002587	YDR179W-A	NVJ3	819433	820824	Nucleus-Vacuole Junction
825927	T	C	55.1533	snp	Chromosome IV	S000002589	YDR181C	SAS4	825910	827355	Something About Silencing
830884	CTT	CTTT	55.3887	ins	Chromosome IV	S000002592	YDR184C	ATC1	830629	831513	Aip Three Complex
833920	C	G	55.7364	snp	Chromosome IV	S000002594	YDR186C	SND1	832859	835492	Srp-iNDependent targeting
838432	T	C	55.5807	snp	Chromosome IV	S000002597	YDR189W	SLY1	838392	840392	Suppressor of Loss of Ypt1
842452	A	G	54.5182	snp	Chromosome IV	S000002599	YDR191W	HST4	842337	843449	Homolog of SIR Two (SIR2)
844727	A	G	55.7364	snp	Chromosome IV	S000002600	YDR192C	NUP42	843569	844861	NUclear Pore
844727	A	G	55.7364	snp	Chromosome IV	S000002601	YDR193W		844554	844952	
847289	C	T	54.5182	snp	Chromosome IV	S000002602	YDR194C	MSS116	845952	847946	Mitochondrial Splicing System
956095	G	T	55.5807	snp	Chromosome IV	S000002655	YDR247W	VHS1	956013	957398	Viable in a Hal3 Sit4 background
956763	C	T	55.8622	snp	Chromosome IV	S000002655	YDR247W	VHS1	956013	957398	Viable in a Hal3 Sit4 background
1026487	A	T	54.1002	snp	Chromosome IV	S000002691	YDR283C	GCN2	1025070	1030049	General Control Nonderepressible
1041378	A	T	54.5182	snp	Chromosome IV	S000002699	YDR291W	HRQ1	1039728	1042961	Homologous to RecQ protein
1054751	TAAAAAAAAAAAT	TAAAAAAAAAAT	56.0449	del	Chromosome IV	intergenic					
1064139	C	A	54.5182	snp	Chromosome IV	S000002709	YDR301W	CFT1	1063352	1067425	Cleavage Factor Two
1084201	T	A	54.8661	snp	Chromosome IV	S000002718	YDR310C	SUM1	1081128	1084316	SUppresor of Mar1-1
1103722	A	G	54.5182	snp	Chromosome IV	intergenic					

1128389	A	T	55.1533	snp	Chromosome IV	S000002738	YDR330W	UBX5	1127872	1129374	UBiquitin regulatory X
1138401	G	A	55.5807	snp	Chromosome IV	S000002742	YDR334W	SWR1	1135932	1140476	SWi2/snf2-Related
1138951	C	A	55.7364	snp	Chromosome IV	S000002742	YDR334W	SWR1	1135932	1140476	SWi2/snf2-Related
1163600	C	A	55.8622	snp	Chromosome IV	S000002753	YDR345C	HXT3	1162957	1164660	HeXose Transporter
1180116	G	A	54.5182	snp	Chromosome IV	S000002759	YDR351W	SBE2	1178666	1181260	Suppressor of BEm4
1198214	C	A	55.1533	snp	Chromosome IV	S000002770	YDR362C	TFC6	1196679	1198697	Transcription Factor C
1198982	A	T	55.9635	snp	Chromosome IV	intergenic					
1230729	A	G	56.0449	snp	Chromosome IV	S000002787	YDR379W	RGA2	1230167	1233196	Rho GTPase Activating Protein
1255104	C	A	54.8661	snp	Chromosome IV	S000002798	YDR390C	UBA2	1254937	1256847	UBiquitin Activating
1257846	C	A	56.1623	snp	Chromosome IV	S000002799	YDR391C		1257358	1258056	
1269223	G	T	54.5182	snp	Chromosome IV	S000002806	YDR398W	UTP5	1267471	1269402	U Three Protein
1283190	G	A	54.5182	snp	Chromosome IV	S000002814	YDR406W	PDR15	1279210	1283799	Pleiotropic Drug Resistance
1284481	A	G	54.1002	snp	Chromosome IV	S000002815	YDR407C	TRS120	1284069	1287938	TRapp Subunit
1291103	T	A	54.1002	snp	Chromosome IV	S000002817	YDR409W	SIZ1	1289406	1292120	SAP and mIZ-finger domain
1316223	A	T	54.8661	snp	Chromosome IV	S000002830	YDR422C	SIP1	1315326	1317773	SNF1-Interacting Protein
1321982	CATATATATATATATATATA	CATATATATATATATATA	56.1623	del	Chromosome IV	S000002834	YDR426C		1321629	1322006	
1323451	C	A	55.5807	snp	Chromosome IV	intergenic					
1341295	C	T	55.8906	snp	Chromosome IV	intergenic					
1342421	A	G	55.5807	snp	Chromosome IV	intergenic					
1375696	A	T	55.1533	snp	Chromosome IV	S000002865	YDR457W	TOM1	1369790	1379596	Temperature dependent Organization in Mitotic nucleus or Trigger Of Mitosis
1393040	T	A	55.7364	snp	Chromosome IV	S000002872	YDR464W	SPP41	1388872	1393179	Suppressor of PrP4

1438942	T	C	54.1002	snp	Chromosome IV	S000002903	YDR495C	VPS3	1438115	1441150	Vacuolar Protein Sorting
1448472	A	T	56.1101	snp	Chromosome IV	S000002907	YDR499W	LCD1	1447830	1450073	Lethal, Checkpoint-defective, DNA damage sensitive
1448480	C	A	54.8661	snp	Chromosome IV	S000002907	YDR499W	LCD1	1447830	1450073	Lethal, Checkpoint-defective, DNA damage sensitive
1458699	C	A	55.3887	snp	Chromosome IV	S000002913	YDR505C	PSP1	1456695	1459220	Polymerase SuPpressor
1461060	A	G	55.7364	snp	Chromosome IV	S000002914	YDR506C	GMC1	1459728	1461554	Grand Meiotic recombination Cluster
1474983	C	T	54.8661	snp	Chromosome IV	S000002924	YDR516C	EMI2	1474974	1476476	Early Meiotic Induction
1512948	G	T	56.1623	snp	Chromosome IV	S000002947	YDR539W	FDC1	1512094	1513605	Ferulic acid DeCarboxylase
44916	A	G	55.5807	snp	Chromosome IX	S000001421	YIL159W	BNR1	41825	45952	BNi1 Related
66424	G	A	54.1002	snp	Chromosome IX	S000001411	YIL149C	MLP2	63028	68067	Myosin-Like Protein
75147	T	A	54.6922	snp	Chromosome IX	S000001408	YIL146C	ATG32	74184	75773	AuTophaGy related
82353	T	A	54.5182	snp	Chromosome IX	S000001405	YIL143C	SSL2	80510	83041	Suppressor of Stem-Loop mutation
100323	G	T	55.9635	snp	Chromosome IX	S000001394	YIL132C	CSM2	99860	100501	Chromosome Segregation in Meiosis
102891	G	A	56.204	snp	Chromosome IX	S000001392	YIL130W	ASG1	102782	105676	Activator of Stress Genes
110882	A	T	54.8661	snp	Chromosome IX	S000001391	YIL129C	TAO3	106107	113237	Transcriptional Activator of OCH1
119485	T	C	55.1533	snp	Chromosome IX	S000001388	YIL126W	STH1	117992	122071	SNF Two Homolog
121164	A	G	55.3887	snp	Chromosome IX	S000001388	YIL126W	STH1	117992	122071	SNF Two Homolog
139973	A	G	55.8622	snp	Chromosome IX	S000001380	YIL118W	RHO3	139752	140447	Ras HOmolog
143735	C	G	55.0097	snp	Chromosome IX	S000001378	YIL116W	HIS5	142928	144085	HIStidine requiring
150021	C	G	54.6922	snp	Chromosome IX	intergenic					
150262	G	T	55.7364	snp	Chromosome IX	intergenic					
153465	A	C	55.7364	snp	Chromosome IX	S000001374	YIL112W	HOS4	151595	154846	Hda One Similar

169903	ATT	AT	54.5182	del	Chromosome IX	intergenic					
181275	A	T	55.8622	snp	Chromosome IX	S000001359	YIL097W	FYV10	180427	181977	Function required for Yeast Viability
186046	G	T	55.1533	snp	Chromosome IX	S000001357	YIL095W	PRK1	183937	186369	p53 Regulatory Kinase
189871	T	C	54.8661	snp	Chromosome IX	S000001354	YIL092W		189066	190967	
257207	C	A	55.7993	snp	Chromosome IX	intergenic					
258277	A	G	54.5182	snp	Chromosome IX	S000001313	YIL051C	MMF1	257843	258280	Mitochondrial Matrix Factor
268558	TCG	TG	54.5182	del	Chromosome IX	intergenic					
298801	A	T	55.4847	snp	Chromosome IX	S000001292	YIL030C	SSM4	296050	300009	Suppressor of mrna Stability Mutant
312201	T	A	54.5182	snp	Chromosome IX	S000001284	YIL022W	TIM44	311165	312460	Translocase of the Inner Mitochondrial membrane
318694	T	A	55.7364	snp	Chromosome IX	S000001279	YIL017C	VID28	318200	320965	Vacuolar Import and Degradation
325394	C	T	56.0449	snp	Chromosome IX	S000003536	YIL014C-A		325212	325526	
325920	A	G	56.204	snp	Chromosome IX	intergenic					
348136	C	A	55.8906	snp	Chromosome IX	S000001266	YIL004C	BET1	347946	348505	Blocked Early in Transport
350585	ATT	AT	54.5182	del	Chromosome IX	intergenic					
367231	G	T	54.8661	snp	Chromosome IX	S000001445	YIR006C	PAN1	365466	369908	Poly(A)-binding protein-dependent poly(A) riboNuclease
379572	G	T	55.7364	snp	Chromosome IX	S000001451	YIR012W	SQT1	378486	379781	Suppressor of QSR1 Truncations
399973	T	C	55.0097	snp	Chromosome IX	S000001462	YIR023W	DAL81	399777	402689	Degradation of Allantoin
404096	C	T	54.8661	snp	Chromosome IX	S000001464	YIR025W	MND2	403659	404765	Meiotic Nuclear Divisions
6315	A	G	54.1002	snp	Chromosome V	S000000800	YEL074W		6126	6464	
13315	T	C	55.9635	snp	Chromosome V	intergenic					
15781	C	A	55.1533	snp	Chromosome V	intergenic					

43087	C	T	55.1533	snp	Chromosome V	intergenic					
43087	C	T	55.1533	snp	Chromosome V	intergenic					
63448	A	T	55.8622	snp	Chromosome V	intergenic					
102579	TGT	TT	56.204	del	Chromosome V	intergenic					
111582	C	A	54.8661	snp	Chromosome V	S000000748	YEL022W	GEA2	111421	115800	Guanine nucleotide Exchange on ARF
138534	C	A	56.1623	snp	Chromosome V	intergenic					
173256	C	T	54.5182	snp	Chromosome V	S000000812	YER010C		172634	173338	
177771	A	G	55.5807	snp	Chromosome V	intergenic					
178329	AC	GA	54.5182	mnp	Chromosome V	S000000814	YER012W	PRE1	177835	178431	PRoteinase yscE
188778	G	A	55.9635	snp	Chromosome V	S000000818	YER016W	BIM1	188277	189311	BInding to Microtubules
195995	G	T	54.5182	snp	Chromosome V	S000000822	YER020W	GPA2	195168	196517	G Protein Alpha subunit
198806	G	A	55.1533	snp	Chromosome V	intergenic					
207884	A	T	54.5182	snp	Chromosome V	S000000828	YER026C	CHO1	207644	208474	CHOline requiring
234420	A	T	54.1002	snp	Chromosome V	S000000843	YER041W	YEN1	232461	234740	
235403	T	A	54.5915	snp	Chromosome V	S000000844	YER042W	MXR1	234937	235491	peptide Methionine sulfoXide Reductase
244683	A	G	55.3887	snp	Chromosome V	S000000849	YER047C	SAP1	243810	246503	Sin1 Associated Protein
388215	C	T	54.9825	snp	Chromosome V	S000000915	YER113C	TMN3	387932	390052	TransMembrane Nine
388875	G	C	56.1623	snp	Chromosome V	S000000915	YER113C	TMN3	387932	390052	TransMembrane Nine
422475	T	A	55.4847	snp	Chromosome V	intergenic					
475697	A	G	55.9635	snp	Chromosome V	S000000956	YER154W	OXA1	475020	476228	cytochrome OXidase Activity
484719	A	T	54.1002	snp	Chromosome V	intergenic					
487532	A	T	55.8622	snp	Chromosome V	intergenic					
524861	C	T	55.3887	snp	Chromosome V	S000000971	YER169W	RPH1	523369	525759	Regulator of PHR1

532626	G	T	56.1623	snp	Chromosome V	S000000974	YER172C	BRR2	529530	536021	Bad Response to Refrigeration
533207	A	T	55.5807	snp	Chromosome V	S000000974	YER172C	BRR2	529530	536021	Bad Response to Refrigeration
551643	G	T	54.8661	snp	Chromosome V	S000000983	YER181C		551473	551796	
556564	TGT	TT	54.1002	del	Chromosome V	S000000986	YER184C	TOG1	556296	558680	Transcriptional regulator of Oleate utilization Genes
561615	C	A	54.8661	snp	Chromosome V	intergenic					
567820	C	A	54.8661	snp	Chromosome V	intergenic					
567841	G	T	56.1036	snp	Chromosome V	intergenic					
22473	TAA	TA	56.2849	del	Chromosome VI	S000001840	YFL054C	AQY3	20847	22787	AQuaporin from Yeast
64495	A	T	54.2021	snp	Chromosome VI	S000006436	YFL034C-A	RPL22B	64243	64932	Ribosomal Protein of the Large subunit
66264	A	C	55.9635	snp	Chromosome VI	S000001860	YFL034W	MIL1	65477	68698	Medium adaptin-Interacting Ligand
71419	T	C	55.1533	snp	Chromosome VI	S000001861	YFL033C	RIM15	69115	74427	Regulator of IME2
76824	A	T	56.0449	snp	Chromosome VI	intergenic		HAC1			
77956	TTAATAAA	TTAAA	55.5807	del	Chromosome VI	S000001864	YFL030W	AGX1	76831	77988	Alanine:Glyoxylate aminotrans(X)ferase
89057	T	A	56.1623	snp	Chromosome VI	S000001870	YFL024C	EPL1	87847	90345	Enhancer of Polycomb Like
106883	G	A	56.0449	snp	Chromosome VI	S000001879	YFL015C		106469	106963	
107775	A	G	54.3092	snp	Chromosome VI	intergenic					
121823	A	T	55.271	snp	Chromosome VI	S000001886	YFL008W	SMC1	119429	123106	Stability of MiniChromosomes
134551	GTT	GT	55.3887	del	Chromosome VI	S000001891	YFL003C	MSH4	134521	137157	MutS Homolog
182067	T	C	56.2849	snp	Chromosome VI	intergenic		IGD1			Inhibitor of Glycogen Debranching
208352	A	T	56.0449	snp	Chromosome VI	intergenic					
213628	T	A	54.8661	snp	Chromosome VI	S000001926	YFR030W	MET10	213312	216419	METhionine requiring
225632	C	T	54.5182	snp	Chromosome VI	S000001930	YFR034C	PHO4	225020	225958	PHOsphate metabolism

248244	A	G	55.1533	snp	Chromosome VI	intergenic					
27789	G	T	54.6922	snp	Chromosome VII	intergenic					
55699	G	T	55.3012	snp	Chromosome VII	S000003204	YGL235W		55279	55815	
55699	G	T	55.271	snp	Chromosome VII	S000003205	YGL236C	MTO1	53787	55796	Mitochondrial Translation Optimization
61837	T	C	56.1623	snp	Chromosome VII	S000003202	YGL233W	SEC15	59122	61854	SECretory
69671	A	G	55.4847	snp	Chromosome VII	S000003196	YGL227W	VID30	69671	72547	Vacuolar Import and Degradation
88329	C	A	55.9635	snp	Chromosome VII	S000003183	YGL215W	CLG1	87981	89339	Cyclin-Like Gene
90682	G	T	56.0449	snp	Chromosome VII	S000003181	YGL213C	SKI8	90054	91247	SuperKIller
94663	A	T	55.7364	snp	Chromosome VII	intergenic					
96241	C	T	55.3887	snp	Chromosome VII	S000003177	YGL209W	MIG2	95858	97006	Multicopy Inhibitor of GAL gene expression
100662	C	A	55.367	snp	Chromosome VII	S000003175	YGL207W	SPT16	98969	102076	SuPpressor of Ty
104879	T	C	55.5807	snp	Chromosome VII	S000003174	YGL206C	CHC1	102543	107504	Clathrin Heavy Chain
120562	C	A	54.5182	snp	Chromosome VII	S000003169	YGL201C	MCM6	117854	120907	MiniChromosome Maintenance
138035	A	T	55.9635	snp	Chromosome VII	S000003163	YGL195W	GCN1	131525	139543	General Control Nonderepressible
155408	G	A	54.5182	snp	Chromosome VII	S000003152	YGL184C	STR3	154615	156012	Sulfur TRansfer
166480	G	A	54.8661	snp	Chromosome VII	intergenic					
187684	C	T	54.5182	snp	Chromosome VII	S000003135	YGL167C	PMR1	187616	190468	Plasma Membrane ATPase Related
187684	C	T	54.5182	snp	Chromosome VII	S000003136	YGL168W	HUR1	187464	187796	HydroxyUrea Resistance
191743	C	G	55.3887	snp	Chromosome VII	S000003133	YGL165C		191398	191976	
191743	C	G	55.3887	snp	Chromosome VII	S000003134	YGL166W	CUP2	191129	191806	
204550	G	T	54.6922	snp	Chromosome VII	intergenic					
221940	G	A	55.3012	snp	Chromosome VII	S000003118	YGL150C	INO80	221104	225573	INOsitol requiring

252188	C	T	54.5182	snp	Chromosome VII	S000003105	YGL137W	SEC27	249869	252738	SECretory
294953	C	A	54.5182	snp	Chromosome VII	S000003082	YGL114W		293460	295637	
307312	G	T	54.8661	snp	Chromosome VII	intergenic					
308163	A	G	54.1002	snp	Chromosome VII	S000003073	YGL105W	ARC1	307437	308567	Aminoacyl-tRNA synthetase Cofactor
313847	C	G	54.8661	snp	Chromosome VII	S000003068	YGL100W	SEH1	313234	314283	SEc13 Homolog
338407	G	A	54.3092	snp	Chromosome VII	S000003060	YGL092W	NUP145	337906	341859	NUclear Pore
347945	C	A	54.1002	snp	Chromosome VII	S000003054	YGL086W	MAD1	347119	349368	Mitotic Arrest-Deficient
371429	T	A	54.5182	snp	Chromosome VII	intergenic					
376820	A	T	55.1533	snp	Chromosome VII	S000003035	YGL067W	NPY1	376101	377255	NADH PYrophosphatase
405995	A	T	55.7364	snp	Chromosome VII	S000003018	YGL050W	TYW3	405776	406597	Trna-YW synthesizing protein
444629	G	A	54.1002	snp	Chromosome VII	S000002995	YGL027C	CWH41	443642	446143	Calcofluor White Hypersensitive
456538	T	A	55.8622	snp	Chromosome VII	S000002989	YGL021W	ALK1	454785	457067	
503282	T	C	55.3887	snp	Chromosome VII	S000003236	YGR004W	PEX31	502938	504326	PEroXisome related
517782	A	T	56.204	snp	Chromosome VII	S000003246	YGR014W	MSB2	516943	520863	Multicopy Suppression of a Budding defect
528485	C	A	54.8661	snp	Chromosome VII	S000003253	YGR021W	DPC29	527632	528504	Delta-Psi dependent mitochondrial import and Cleavage protein of ~29 kDa
587996	G	T	54.8661	snp	Chromosome VII	S000003279	YGR047C	TFC4	586392	589469	Transcription Factor class C
609639	G	T	55.3887	snp	Chromosome VII	intergenic					
644608	G	T	55.9635	snp	Chromosome VII	intergenic					
651162	G	A	56.0449	snp	Chromosome VII	intergenic					
681095	G	A	54.5182	snp	Chromosome VII	S000003329	YGR097W	ASK10	678695	682135	Activator of SKn7
732413	A	T	54.8661	snp	Chromosome VII	S000003353	YGR121C	MEP1	731449	732927	
737815	T	A	55.7993	snp	Chromosome VII	S000003355	YGR123C	PPT1	736662	738203	Protein Phosphatase T

742795	T	C	54.3092	snp	Chromosome VII	S000003357	YGR125W		742325	745435	
745955	G	A	56.1623	snp	Chromosome VII	S000003358	YGR126W		745835	746527	
771631	A	G	56.1623	snp	Chromosome VII	S000003373	YGR141W	VPS62	770569	771972	Vacuolar Protein Sorting
776275	A	T	55.7364	snp	Chromosome VII	S000003375	YGR143W	SKN1	775193	777508	Suppressor of Kre Null
783924	C	T	54.1002	snp	Chromosome VII	intergenic					
835056	CTT	CT	54.8661	del	Chromosome VII	S000003401	YGR169C	PUS6	834689	835903	PseudoUridine Synthase
872453	T	C	56.1623	snp	Chromosome VII	S000003420	YGR188C	BUB1	872044	875109	Budding Uninhibited by Benzimidazole
877485	A	G	54.5182	snp	Chromosome VII	S000003421	YGR189C	CRH1	876669	878192	Congo Red Hypersensitive
885444	C	A	54.8661	snp	Chromosome VII	S000003425	YGR193C	PDX1	884509	885741	Pyruvate Dehydrogenase complex protein X
930431	A	G	55.3887	snp	Chromosome VII	S000003449	YGR217W	CCH1	924696	930815	Calcium Channel Homolog
934407	C	A	56.1623	snp	Chromosome VII	S000003450	YGR218W	CRM1	932541	935795	Chromosome Region Maintenance
935780	C	T	55.3887	snp	Chromosome VII	S000003450	YGR218W	CRM1	932541	935795	Chromosome Region Maintenance
955451	C	A	54.8661	snp	Chromosome VII	S000003465	YGR233C	PHO81	954674	958210	PHOsphate metabolism
959516	T	A	54.5182	snp	Chromosome VII	intergenic					
965426	T	C	55.5807	snp	Chromosome VII	S000003469	YGR237C		963298	965655	
968891	CTTTTTTTTTTTTC	CTTTTTTTTTTTC	55.271	del	Chromosome VII	intergenic					
991783	T	A	55.3887	snp	Chromosome VII	S000003482	YGR250C	RIE1	991176	993521	Restoration of Impaired growths of ERMES-lacking cells
1010825	T	A	55.8622	snp	Chromosome VII	intergenic					
1012220	ATTTTTTTTTTG	ATTTTTTTTTTTG	55.5807	ins	Chromosome VII	intergenic					
1047305	A	G	55.8906	snp	Chromosome VII	S000003510	YGR278W	CWC22	1046731	1048464	Complexed With Cef1p
1059663	GTTTTTTTTTTTG	GTTTTTTTTTTG	54.8661	del	Chromosome VII	S000003515	YGR283C		1059015	1060040	

1065364	T	C	55.7993	snp	Chromosome VII	intergenic					
16050	A	G	55.1533	snp	Chromosome VIII	S000001034	YHL042W		15667	16119	
16383	T	A	56.0449	snp	Chromosome VIII	intergenic					
36684	G	A	54.1002	snp	Chromosome VIII	S000001024	YHL032C	GUT1	36379	38508	Glycerol UTilization
50759	C	T	55.5807	snp	Chromosome VIII	intergenic					
53029	G	A	55.3887	snp	Chromosome VIII	intergenic					
54477	G	A	55.1533	snp	Chromosome VIII	intergenic					
61310	G	T	54.1002	snp	Chromosome VIII	S000001015	YHL023C	NPR3	59123	62563	Nitrogen Permease Regulator
74813	T	A	54.1002	snp	Chromosome VIII	intergenic					
84018	G	A	56.157	snp	Chromosome VIII	intergenic					
93806	C	A	56.0449	snp	Chromosome VIII	S000001000	YHL008C		92627	94510	
93814	ATT	AT	56.0449	del	Chromosome VIII	S000001000	YHL008C		92627	94510	
117113	C	T	56.1101	snp	Chromosome VIII	intergenic					
185563	T	A	54.5182	snp	Chromosome VIII	S000001081	YHR039C	MSC7	184875	186809	Meiotic Sister-Chromatid recombination
200731	A	G	54.8661	snp	Chromosome VIII	S000001089	YHR047C	AAP1	198740	201310	Arginine/alanine AminoPeptidase
233230	T	A	54.1002	snp	Chromosome VIII	S000001110	YHR068W	DYS1	232133	233296	DeoxyhYpusine Synthase
240860	G	T	54.5182	snp	Chromosome VIII	S000001114	YHR072W	ERG7	239098	241293	ERGosterol biosynthesis
289228	T	C	54.1002	snp	Chromosome VIII	S000001135	YHR093W	AHT1	289142	289690	
301908	A	T	56.204	snp	Chromosome VIII	S000001140	YHR098C	SFB3	299145	301934	Sed Five Binding
307376	T	A	55.7364	snp	Chromosome VIII	S000001141	YHR099W	TRA1	302761	313995	similar to human TRRAP

312715	A	G	55.271	snp	Chromosome VIII	S000001141	YHR099W	TRA1	302761	313995	similar to human TRRAP
314834	G	T	55.3887	snp	Chromosome VIII	intergenic					
340321	G	T	55.4847	snp	Chromosome VIII	S000001157	YHR115C	DMA1	340109	341359	Defective in Mitotic Arrest
346798	C	T	54.8661	snp	Chromosome VIII	S000001161	YHR119W	SET1	346043	349285	SET domain-containing
352842	G	T	56.2849	snp	Chromosome VIII	S000001163	YHR121W	LSM12	352756	353319	Like SM
365287	A	G	54.1002	snp	Chromosome VIII	S000001172	YHR130C		364965	365300	
376511	T	C	54.8661	snp	Chromosome VIII	S000001179	YHR137W	ARO9	375709	377250	AROmatic amino acid requiring
404477	C	A	54.5182	snp	Chromosome VIII	S000001197	YHR154W	RTT107	402966	406178	Regulator of Ty1 Transposition
412873	G	C	54.8661	snp	Chromosome VIII	intergenic					
413152	A	G	55.271	snp	Chromosome VIII	S000001200	YHR157W	REC104	412907	413455	RECombination
417040	G	A	54.1002	snp	Chromosome VIII	S000001201	YHR158C	KEL1	413685	417179	KELch repeat
417054	G	A	54.1002	snp	Chromosome VIII	S000001201	YHR158C	KEL1	413685	417179	KELch repeat
439349	A	G	54.8661	snp	Chromosome VIII	S000001210	YHR167W	THP2	439342	440127	THO2 - HPR1 Phenotype
494656	TCA	TA	54.8661	del	Chromosome VIII	S000001240	YHR197W	RIX1	493896	496187	RIbosome eXport
514254	C	A	54.8661	snp	Chromosome VIII	S000001249	YHR206W	SKN7	512732	514600	Suppressor of Kre Null
23491	A	G	54.5182	snp	Chromosome X	S000003753	YJL217W	REE1	23133	23729	REgulation of Enolase
33731	G	A	55.7364	snp	Chromosome X	intergenic					
37123	G	T	54.1002	snp	Chromosome X	S000003746	YJL210W	PEX2	36919	37734	PEroXin
37123	G	T	54.1002	snp	Chromosome X	S000003747	YJL211C		36757	37200	
41124	G	T	55.8622	snp	Chromosome X	S000003744	YJL208C	NUC1	40194	41183	NUClease
51220	T	C	56.2849	snp	Chromosome X	S000003740	YJL204C	RCY1	50629	53151	ReCYcling

61069	A	G	54.1002	snp	Chromosome X	S000003734	YJL198W	PHO90	60844	63489	PHOsphate metabolism
69162	T	C	54.8661	snp	Chromosome X	intergenic					
73566	G	T	55.3887	snp	Chromosome X	intergenic					
76337	T	C	54.3092	snp	Chromosome X	S000003724	YJL188C	BUD19	76202	76510	BUD site selection
76337	T	C	54.3092	snp	Chromosome X	S000003725	YJL189W	RPL39	75933	76474	Ribosomal Protein of the Large subunit
90353	C	A	55.7364	snp	Chromosome X	intergenic		ATG27			
105224	G	A	54.5182	snp	Chromosome X	S000003703	YJL167W	ERG20	105014	106072	ERGosterol biosynthesis
107614	G	A	54.8661	snp	Chromosome X	S000003701	YJL165C	HAL5	106894	109461	HALotolerance
113064	A	G	54.5182	snp	Chromosome X	S000003699	YJL163C		111666	113333	
126719	A	T	55.8622	snp	Chromosome X	S000007613	YJL156W-A		126604	126825	
156811	C	T	54.6922	snp	Chromosome X	S000003672	YJL136C	RPS21B	156550	157273	Ribosomal Protein of the Small subunit
164216	A	G	55.8906	snp	Chromosome X	intergenic					
171390	A	G	54.2342	snp	Chromosome X	S000003666	YJL130C	URA2	165723	172367	URAcil requiring
191221	A	G	56.3017	snp	Chromosome X	S000003656	YJL120W		191022	191345	Dubios Protein
210541	G	T	55.7364	snp	Chromosome X	S000003646	YJL110C	GZF3	209922	211577	Gata Zinc Finger protein
238097	C	T	55.3887	snp	Chromosome X	S000003636	YJL100W	LSB6	237263	239086	Las Seventeen Binding protein
253897	G	T	56.2849	snp	Chromosome X	S000003630	YJL094C	KHA1	251821	254442	K/H ion Antiporter
262185	T	C	55.5807	snp	Chromosome X	S000003627	YJL091C	GWT1	261080	262552	GPI-anchored Wall protein Transfer
262215	ATTTTTTTTTTA	ATTTTTTTTTA	54.1002	del	Chromosome X	S000003627	YJL091C	GWT1	261080	262552	GPI-anchored Wall protein Transfer
280434	T	C	54.5182	snp	Chromosome X	S000003619	YJL083W	TAX4	278841	280655	
282265	GC	CT	54.8661	mnp	Chromosome X	S000003618	YJL082W	IML2	281185	283380	Increased Minichromosome Loss
286745	G	C	56.1623	snp	Chromosome X	S000003616	YJL080C	SCP160	285558	289226	S. cerevisiae protein involved in the Control of Ploidy

287402	T	A	56.1623	snp	Chromosome X	S000003616	YJL080C	SCP160	285558	289226	S. cerevisiae protein involved in the Control of Ploidy
304525	C	A	54.1002	snp	Chromosome X	S000003609	YJL073W	JEM1	303181	305118	DnaJ-like protein of the ER Membrane
305377	A	G	54.8661	snp	Chromosome X	S000003608	YJL072C	PSF2	305221	305862	Partner of Sld Five
317497	A	T	54.5182	snp	Chromosome X	S000003598	YJL062W	LAS21	317284	319776	Local Anestheticum Sensitive
321893	T	G	56.1623	snp	Chromosome X	S000003597	YJL061W	NUP82	320016	322157	NUclear Pore
325557	T	G	54.8661	snp	Chromosome X	S000003595	YJL059W	YHC3	324964	326190	Yeast Homolog of human Cln3
328578	G	T	56.1623	snp	Chromosome X	S000003593	YJL057C	IKS1	328118	330121	
371113	G	T	55.3887	snp	Chromosome X	S000003576	YJL039C	NUP192	368748	373799	NUclear Pore
376270	T	C	56.204	snp	Chromosome X	intergenic					
389521	C	T	54.5182	snp	Chromosome X	S000003566	YJL029C	VPS53	388385	390853	Vacuolar Protein Sorting
395231	A	G	54.3092	snp	Chromosome X	S000003562	YJL025W	RRN7	393967	395511	Regulation of RNA polymerase I
408264	G	A	56.1623	snp	Chromosome X	S000003551	YJL014W	CCT3	407558	409162	Chaperonin Containing TCP-1
431319	C	A	54.6922	snp	Chromosome X	intergenic					
449733	A	G	55.7364	snp	Chromosome X	S000003766	YJR006W	POL31	449202	450665	POLymerase
516257	C	A	54.5182	snp	Chromosome X	S000003803	YJR042W	NUP85	514055	516289	NUclear Pore
617850	T	A	54.1002	snp	Chromosome X	intergenic					
630760	T	A	55.7364	snp	Chromosome X	S000003870	YJR109C	CPA2	629585	632941	Carbamyl Phosphate synthetase A
635036	A	G	54.5182	snp	Chromosome X	S000003871	YJR110W	YMR1	633615	635681	Yeast Myotubularin Related
684682	T	A	56.2849	snp	Chromosome X	S000003899	YJR138W	IML1	684567	689321	Increased Minichromosome Loss
11029	C	A	54.8661	snp	Chromosome XI	S000001703	YKL220C	FRE2	9091	11226	Ferric REductase

23616	C	A	56.2849	snp	Chromosome XI	S000001700	YKL217W	JEN1	22234	24084	Monocarboxylate/proton symporter of the plasma membrane; transport activity is dependent on the pH gradient across the membrane
29768	TAA	TA	54.5182	del	Chromosome XI	S000001698	YKL215C	OXP1	26827	30687	OXoProlinase
36108	G	A	55.1533	snp	Chromosome XI	S000001695	YKL212W	SAC1	34543	36414	Suppressor of ACtin
53051	G	T	56.1831	snp	Chromosome XI	S000001688	YKL205W	LOS1	50051	53353	Loss Of Suppression
73014	C	G	55.271	snp	Chromosome XI	S000001680	YKL197C	PEX1	70734	73865	PEroXin
75595	G	T	55.5807	snp	Chromosome XI	intergenic					
84164	G	C	55.1533	snp	Chromosome XI	intergenic					
106578	G	C	54.8661	snp	Chromosome XI	S000001665	YKL182W	FAS1	100671	106826	Fatty Acid Synthetase
108579	G	T	55.5807	snp	Chromosome XI	S000001664	YKL181W	PRS1	107316	108599	PhosphoRibosylpyrophosphate Synthetase
113724	G	T	54.5182	snp	Chromosome XI	S000001661	YKL178C	STE3	113215	114627	STErile
124720	T	A	56.0449	snp	Chromosome XI	S000001656	YKL173W	SNU114	122517	125543	Small NUclear ribonucleoprotein associated
132145	G	T	56.2849	snp	Chromosome XI	S000001651	YKL168C	KKQ8	131288	133462	Putative serine/threonine protein kinase with unknown cellular role
150977	TC	TGCC	55.5807	ins	Chromosome XI	intergenic					
151757	A	G	55.9635	snp	Chromosome XI	intergenic					
156817	T	C	55.7364	snp	Chromosome XI	S000001640	YKL157W	APE2	154991	158232	AminoPEptidase
159328	C	A	55.7364	snp	Chromosome XI	intergenic					
167426	TCC	TC	54.6922	del	Chromosome XI	S000001633	YKL150W	MCR1	166544	167452	Mitochondrial NADH-Cytochrome b5 Reductase
175948	A	G	54.1002	snp	Chromosome XI	S000001627	YKL144C	RPC25	175843	176481	RNA Polymerase C
191652	A	T	55.0097	snp	Chromosome XI	intergenic					

243881	C	A	56.0449	snp	Chromosome XI	S000001587	YKL104C	GFA1	243220	245373	Glutamine:Fructose-6-phosphate Amidotransferase
244249	G	T	54.3092	snp	Chromosome XI	S000001587	YKL104C	GFA1	243220	245373	Glutamine:Fructose-6-phosphate Amidotransferase
244308	TAA	TAAA	56.2849	ins	Chromosome XI	S000001587	YKL104C	GFA1	243220	245373	Glutamine:Fructose-6-phosphate Amidotransferase
294653	T	C	55.1533	snp	Chromosome XI	S000001557	YKL074C	MUD2	294610	296193	Mutant U1 Die
307080	G	C	55.0097	snp	Chromosome XI	intergenic					
313592	C	G	56.157	snp	Chromosome XI	intergenic					
317246	A	G	54.3092	snp	Chromosome XI	intergenic		mnr2			
324344	A	G	56.1623	snp	Chromosome XI	S000001545	YKL062W	MSN4	323228	325120	Multicopy suppressor of SNF1 mutation
327963	C	A	55.3887	snp	Chromosome XI	intergenic					
334853	C	A	55.9635	snp	Chromosome XI	S000001539	YKL056C	TMA19	334412	334915	Translation Machinery Associated
391234	A	T	54.5182	snp	Chromosome XI	S000001508	YKL025C	PAN3	390240	392279	Poly(A) Nuclease
402538	C	A	54.8661	snp	Chromosome XI	intergenic					
412908	TAA	TA	54.1002	del	Chromosome XI	S000001497	YKL014C	URB1	411619	416913	Unhealthy Ribosome Biogenesis
424630	C	A	55.3887	snp	Chromosome XI	S000001493	YKL010C	UFD4	421424	425875	Ubiquitin Fusion Degradation protein
428401	T	C	55.3887	snp	Chromosome XI	S000001491	YKL008C	LAC1	427295	428551	Longevity-Assurance gene Cognate (LAG1 Cognate)
429619	A	G	54.8661	snp	Chromosome XI	S000001490	YKL007W	CAP1	429302	430108	CAPping
430913	T	A	55.8906	snp	Chromosome XI	intergenic					
462915	G	T	55.3887	snp	Chromosome XI	intergenic					
466401	C	G	56.1623	snp	Chromosome XI	intergenic					
466677	A	T	55.0097	snp	Chromosome XI	intergenic					
466690	C	T	55.1533	snp	Chromosome XI	intergenic		YPT52			

480435	G	T	54.8661	snp	Chromosome XI	S000001729	YKR021W	ALY1	479234	481981	Arrestin-Like Yeast protein
480828	G	T	54.1002	snp	Chromosome XI	S000001729	YKR021W	ALY1	479234	481981	Arrestin-Like Yeast protein
492267	A	G	54.8661	snp	Chromosome XI	S000001735	YKR027W	BCH2	491364	493661	Bud7 and Chs6 Homolog
492267	A	G	54.8661	snp	Chromosome XI	S000001735	YKR027W	BCH2	491364	493661	Bud7 and Chs6 Homolog
548307	C	G	56.3017	snp	Chromosome XI	S000001763	YKR055W	RHO4	548216	549091	Ras HOmolog
575736	G	T	56.0449	snp	Chromosome XI	S000001779	YKR071C	DRE2	574934	575980	Derexpressed for Ribosomal protein S14 Expression
602277	A	G	54.3092	snp	Chromosome XI	S000001794	YKR086W	PRP16	599857	603072	Pre-mRNA Processing
608034	G	T	54.8661	snp	Chromosome XI	S000001797	YKR089C	TGL4	605633	608365	TriacylGlycerol Lipase
622662	A	T	54.1002	snp	Chromosome XI	S000001803	YKR095W	MLP1	619805	625432	Myosin-Like Protein
623609	G	T	56.1623	snp	Chromosome XI	S000001803	YKR095W	MLP1	619805	625432	Myosin-Like Protein
634339	T	C	54.1002	snp	Chromosome XI	S000001806	YKR098C	UBP11	633026	635179	UBiquitin-specific Protease
15118	GTT	GTTT	54.5182	ins	Chromosome XII	S000003986	YLL063C	AYT1	14648	16072	AcetYlTransferase
60275	C	T	55.3887	snp	Chromosome XII	S000003963	YLL040C	VPS13	54211	63645	Vacuolar Protein Sorting
73886	C	A	55.1533	snp	Chromosome XII	S000003956	YLL033W	IRC19	73409	74101	Increased Recombination Centers
79980	A	T	54.5182	snp	Chromosome XII	S000003954	YLL031C	GPI13	77152	80205	GlycosylPhosphatidylInositol anchor biosynthesis
80917	A	G	55.5807	snp	Chromosome XII	intergenic					
84090	C	G	55.1533	snp	Chromosome XII	intergenic					
99951	C	A	55.8622	snp	Chromosome XII	S000003945	YLL022C	HIF1	99044	100201	Hat1 Interacting Factor
103060	CAA	CA	55.5807	del	Chromosome XII	S000003944	YLL021W	SPA2	100947	105347	Spindle Pole Antigen
119453	A	G	54.5182	snp	Chromosome XII	S000003938	YLL015W	BPT1	116432	121111	Bile Pigment Transporter
155137	C	T	55.8622	snp	Chromosome XII	S000003992	YLR002C	NOC3	154343	156334	NucleOlar Complex associated
181332	T	C	55.7364	snp	Chromosome XII	S000004009	YLR019W	PSR2	180288	181481	Plasma membrane Sodium Response

187137	ATT	AT	54.5182	del	Chromosome XII	intergenic					
228881	T	C	55.3887	snp	Chromosome XII	intergenic					
261947	G	T	56.3017	snp	Chromosome XII	S000004050	YLR060W	FRS1	260979	262766	phenylalanyl (F)-tRNA Synthetase
281955	G	A	54.6693	snp	Chromosome XII	intergenic					
298148	A	T	54.8661	snp	Chromosome XII	S000004074	YLR084C	RAX2	296589	300251	
301650	G	A	56.1101	snp	Chromosome XII	S000004075	YLR085C	ARP6	300673	301989	Actin-Related Protein
307186	A	T	55.1533	snp	Chromosome XII	S000004077	YLR087C	CSF1	306855	315731	Cold Sensitive for Fermentation
311161	T	C	55.7364	snp	Chromosome XII	S000004077	YLR087C	CSF1	306855	315731	Cold Sensitive for Fermentation
328880	A	G	54.8661	snp	Chromosome XII	S000004084	YLR094C	GIS3	327730	329238	Glg1-2 Suppressor
459885	C	T	54.8661	snp	Chromosome XII	intergenic					
459928	T	C	54.8661	snp	Chromosome XII	intergenic					
459964	T	A	56.0775	snp	Chromosome XII	intergenic					
460156	A	T	56.0449	snp	Chromosome XII	intergenic					
460477	ATTTTTTTTC	ATTTTTTTC	55.1533	del	Chromosome XII	intergenic					
460490	T	A	55.8622	snp	Chromosome XII	intergenic					
504524	T	C	54.1002	snp	Chromosome XII	intergenic					
514561	C	A	54.1002	snp	Chromosome XII	S000004169	YLR179C		514108	514713	
521523	T	C	55.9635	snp	Chromosome XII	S000004173	YLR183C	TOS4	520543	522012	Target Of Sbf
534667	A	T	55.7364	snp	Chromosome XII	intergenic					
546574	A	G	55.3887	snp	Chromosome XII	S000004187	YLR197W	NOP56	546097	547611	NucleOlar Protein of 56.8 kDa
549560	GTAT	GT	56.1623	del	Chromosome XII	S000004191	YLR201C	COQ9	549511	550293	COenzyme Q
558938	G	A	55.8622	snp	Chromosome XII	S000004197	YLR207W	HRD3	556788	559289	HMG-coA Reductase Degradation

569949	A	G	54.3092	snp	Chromosome XII	S000004204	YLR214W	FRE1	568567	570627	Ferric REductase
579092	T	C	54.1002	snp	Chromosome XII	intergenic					
583254	TGA	TA	55.1533	del	Chromosome XII	S000004213	YLR223C	IFH1	582233	585490	Interacts with Fork Head
630304	T	C	54.5182	snp	Chromosome XII	S000004237	YLR247C	IRC20	628684	633354	Increased Recombination Centers
658886	G	T	55.1533	snp	Chromosome XII	S000004247	YLR257W		658826	659791	
670017	A	G	56.2849	snp	Chromosome XII	intergenic		TMA7			Protein of unknown that associates with ribosomes
687262	CTT	CT	54.5182	del	Chromosome XII	intergenic					
698051	C	A	55.3887	snp	Chromosome XII	S000004267	YLR277C	YSH1	697156	699495	Yeast Seventy-three Homolog
700135	A	G	55.0097	snp	Chromosome XII	S000004268	YLR278C		699999	704024	
713451	C	T	54.1002	snp	Chromosome XII	intergenic					
729675	G	T	54.3092	snp	Chromosome XII	S000004291	YLR300W	EXG1	728955	730301	EXo-1,3-beta-Glucanase
771056	T	A	55.8622	snp	Chromosome XII	S000004311	YLR319C	BUD6	769318	771684	BUD site selection
820436	C	A	54.8661	snp	Chromosome XII	intergenic					
872563	CAAAAAC	CAAAAC	55.1533	del	Chromosome XII	S000004367	YLR375W	STP3	871697	872728	protein with similarity to Stp1p
880583	G	T	56.204	snp	Chromosome XII	S000004373	YLR381W	CTF3	879723	881924	Chromosome Transmission Fidelity
892946	C	G	55.5807	snp	Chromosome XII	intergenic		HMO1			
949243	C	G	55.7364	snp	Chromosome XII	intergenic					
950305	CTTTTTTTTTTTG	CTTTTTTTTTTTTG	55.5807	ins	Chromosome XII	S000028572	YLR412C-A		950267	950473	
994777	G	T	56.157	snp	Chromosome XII	S000004422	YLR430W	SEN1	993434	1000129	Splicing ENdonuclease
1000906	T	C	54.1002	snp	Chromosome XII	S000004423	YLR431C	ATG23	1000342	1001703	AuTophaGy related
1006709	T	C	55.8622	snp	Chromosome XII	S000004427	YLR435W	TSR2	1006378	1006995	Twenty S rRNA accumulation
1020829	ATTTTTTTTTC	ATTTTTTTTC	55.1533	del	Chromosome XII	S000004434	YLR442C	SIR3	1019315	1022251	Silent Information Regulator

1034469	A	G	54.3092	snp	Chromosome XII	S000004442	YLR450W	HMG2	1032627	1035764	3-Hydroxy-3-MethylGlutaryl-coenzyme a reductase
8719	T	A	55.7364	snp	Chromosome XIII	intergenic					
11843	C	A	55.7364	snp	Chromosome XIII	S000004599	YML130C	ERO1	11483	13174	ER Oxidation or Endoplasmic Reticulum Oxidoreductin
14697	T	A	54.8661	snp	Chromosome XIII	S000004598	YML129C	COX14	14541	14753	Cytochrome c OXidase
23102	T	C	55.3887	snp	Chromosome XIII	S000004593	YML124C	TUB3	22048	23683	TUBulin
28261	T	C	55.7364	snp	Chromosome XIII	intergenic					
48164	T	A	55.9635	snp	Chromosome XIII	S000004579	YML111W	BUL2	46942	49704	Binds Ubiquitin Ligase
112707	C	A	55.3887	snp	Chromosome XIII	S000004541	YML076C	WAR1	112513	115347	Weak Acid Resistance
132877	C	T	55.7364	snp	Chromosome XIII	intergenic					
144575	A	T	54.1002	snp	Chromosome XIII	S000004530	YML065W	ORC1	142210	144954	Origin Recognition Complex
166295	A	G	55.3887	snp	Chromosome XIII	S000004518	YML054C	CYB2	165533	167308	CYtochrome B
170352	C	T	55.3887	snp	Chromosome XIII	intergenic					
220011	C	A	54.5182	snp	Chromosome XIII	intergenic					
245578	G	T	54.3092	snp	Chromosome XIII	S000004474	YML012C-A		245537	245914	
245578	G	T	54.3092	snp	Chromosome XIII	S000004475	YML013W	UBX2	244149	245903	UBiquitin regulatory X
261584	A	G	54.8661	snp	Chromosome XIII	S000004464	YML005W	TRM12	260221	261609	TRna Methyltransferase
292608	TACCT	TT	55.3887	del	Chromosome XIII	S000004614	YMR012W	CLU1	291134	294967	CLUstered mitochondria
301448	ACA	AA	54.8661	del	Chromosome XIII	S000004617	YMR015C	ERG5	300869	302485	ERGosterol biosynthesis
325905	T	A	56.204	snp	Chromosome XIII	S000004629	YMR027W		325877	327289	

325905	T	A	56.204	snp	Chromosome XIII	S000004629	YMR027W		325877	327289	
325909	TAA	TA	56.204	del	Chromosome XIII	S000004629	YMR027W		325877	327289	
335457	G	A	56.1101	snp	Chromosome XIII	S000004635	YMR032W	HOF1	335298	337307	Homolog Of cdc Fifteen
338713	A	G	55.5807	snp	Chromosome XIII	S000004636	YMR033W	ARP9	337788	339277	Actin-Related Protein
364341	G	T	54.8661	snp	Chromosome XIII	S000004650	YMR047C	NUP116	363364	366705	NUclear Pore
369413	G	A	55.3887	snp	Chromosome XIII	S000004652	YMR049C	ERB1	368094	370517	Eukaryotic Ribosome Biogenesis
412910	T	C	55.9635	snp	Chromosome XIII	intergenic					
431603	C	G	54.8661	snp	Chromosome XIII	intergenic					
442579	G	T	54.1002	snp	Chromosome XIII	S000004693	YMR087W	PDL32	442527	443381	Protein of Dual Localization of 32 kDa
448333	GA	AG	55.1533	mnp	Chromosome XIII	intergenic					
473478	G	A	56.1101	snp	Chromosome XIII	S000004710	YMR104C	YPK2	473420	475453	Yeast Protein Kinase
500655	T	A	54.3092	snp	Chromosome XIII	S000004722	YMR116C	ASC1	499456	500688	Absence of growth Suppressor of Cyp1
500655	T	A	54.3092	snp	Chromosome XIII	S000004722	YMR116C	ASC1	499456	500688	Absence of growth Suppressor of Cyp1
547098	T	G	55.1533	snp	Chromosome XIII	S000004747	YMR139W	RIM11	546125	547237	Regulator of IME2
552370	T	G	54.1002	snp	Chromosome XIII	S000004751	YMR143W	RPS16A	551928	552903	Ribosomal Protein of the Small subunit
576363	A	G	56.1101	snp	Chromosome XIII	S000004770	YMR160W		575066	577516	
601036	A	T	56.1623	snp	Chromosome XIII	intergenic					
613224	A	G	55.9635	snp	Chromosome XIII	S000004788	YMR176W	ECM5	611740	615975	ExtraCellular Mutant
618860	A	G	54.5182	snp	Chromosome XIII	S000004790	YMR178W	FPY1	618479	619303	FAD PYrophosphatase
643340	A	T	55.7364	snp	Chromosome XIII	S000004802	YMR190C	SGS1	640915	645258	Slow Growth Suppressor

645183	T	C	54.8661	snp	Chromosome XIII	S000004802	YMR190C	SGS1	640915	645258	Slow Growth Suppressor
652148	T	C	55.7364	snp	Chromosome XIII	intergenic					
679442	T	C	54.5182	snp	Chromosome XIII	S000004820	YMR207C	HFA1	677193	683564	
690829	G	T	54.8661	snp	Chromosome XIII	S000004825	YMR212C	EFR3	690695	693043	PHO Eighty Five Requiring
693618	C	A	54.1002	snp	Chromosome XIII	S000004826	YMR213W	CEF1	693381	695153	CErevisiae homolog of cdc Five
700539	G	C	55.1533	snp	Chromosome XIII	S000004829	YMR216C	SKY1	698811	701039	SRPK1-like Kinase in Yeast
716819	ACC	AC	55.7364	del	Chromosome XIII	S000004836	YMR223W	UBP8	716715	718130	UBiquitin-specific processing Protease
718664	TGG	TG	54.5182	del	Chromosome XIII	S000004837	YMR224C	MRE11	718575	720653	Meiotic REcombination
736728	A	G	54.8661	snp	Chromosome XIII	intergenic					
773393	T	C	54.1002	snp	Chromosome XIII	S000004863	YMR251W	GTO3	772915	774015	Glutathione Transferase Omega-like
799204	T	C	55.8622	snp	Chromosome XIII	S000004879	YMR266W	RSN1	798518	801379	Rescue of Sro7 at high Nacl
807205	T	A	54.5182	snp	Chromosome XIII	S000004884	YMR271C	URA10	806865	807548	URAcil requiring
809198	A	G	54.1002	snp	Chromosome XIII	intergenic					
816788	G	A	54.3092	snp	Chromosome XIII	S000004888	YMR275C	BUL1	815651	818581	Binds Ubiquitin Ligase
831622	A	T	55.8622	snp	Chromosome XIII	intergenic		CAT8			
839527	C	G	56.1623	snp	Chromosome XIII	S000004897	YMR284W	YKU70	838187	839995	Yeast KU protein
848031	T	C	55.9635	snp	Chromosome XIII	S000004901	YMR288W	HSH155	845571	848486	Human Sap Homolog
856919	A	T	55.8906	snp	Chromosome XIII	intergenic					
859551	C	A	55.3887	snp	Chromosome XIII	S000004911	YMR296C	LCB1	859215	860891	Long-Chain Base
885300	GG	GTG	54.1002	ins	Chromosome XIII	S000004923	YMR306W	FKS3	881159	886516	FK506 Sensitivity

886865	G	T	54.1002	snp	Chromosome XIII	intergenic					
897577	T	A	54.5182	snp	Chromosome XIII	S000004928	YMR311C	GLC8	896914	897603	GLyCogen
902163	A	T	56.2849	snp	Chromosome XIII	S000004931	YMR314W	PRE5	901709	902413	PRoteinase yscE
33082	T	A	56.204	snp	Chromosome XIV	S000005267	YNL323W	LEM3	31944	33188	Ligand-Effect Modulator
40019	T	A	55.8906	snp	Chromosome XIV	S000005262	YNL318C	HXT14	38707	40329	HeXose Transporter
46696	A	T	55.8622	snp	Chromosome XIV	S000005257	YNL313C	EMW1	45308	48022	Essential for Maintenance of the cell Wall
53943	G	T	55.5807	snp	Chromosome XIV	intergenic					
80273	G	T	55.1533	snp	Chromosome XIV	intergenic					
120845	T	A	55.1533	snp	Chromosome XIV	S000005219	YNL275W	BOR1	119268	120998	BORon transporter
134206	G	T	55.3887	snp	Chromosome XIV	S000005215	YNL271C	BNI1	129522	135383	Bud Neck Involved
147246	A	T	55.5807	snp	Chromosome XIV	S000005207	YNL263C	YIF1	146896	147840	YIP1-Interacting Factor
151196	A	T	54.1002	snp	Chromosome XIV	S000005206	YNL262W	POL2	148212	154880	POLymerase
164651	C	A	55.3887	snp	Chromosome XIV	S000005200	YNL256W	FOL1	164623	167097	FOLic acid synthesis
186673	C	A	54.1002	snp	Chromosome XIV	S000005189	YNL245C	CWC25	186346	186885	Complexed With Cef1p
196887	T	A	55.9635	snp	Chromosome XIV	S000005185	YNL241C	ZWF1	196426	197943	ZWischenFerment
200096	A	T	55.7364	snp	Chromosome XIV	intergenic					
204839	G	T	54.5182	snp	Chromosome XIV	S000005182	YNL238W	KEX2	202428	204872	Killer EXpression defective
216696	C	A	55.9635	snp	Chromosome XIV	S000005175	YNL231C	PDR16	215987	217042	Pleiotropic Drug Resistance
247965	G	A	54.5182	snp	Chromosome XIV	S000005156	YNL212W	VID27	247461	249809	Vacuolar Import and Degradation
255202	T	C	54.1002	snp	Chromosome XIV	intergenic					

266622	A	G	56.1036	snp	Chromosome XIV	S000005142	YNL198C		266514	266816	
367728	T	C	54.8661	snp	Chromosome XIV	S000005082	YNL138W	SRV2	366741	368321	Suppressor of RasVal19
374067	A	G	55.1533	snp	Chromosome XIV	intergenic					
376281	C	T	55.1533	snp	Chromosome XIV	S000005076	YNL132W	KRE33	375321	378491	Killer toxin REsistant
377887	G	T	55.8622	snp	Chromosome XIV	S000005076	YNL132W	KRE33	375321	378491	Killer toxin REsistant
401213	A	G	54.8661	snp	Chromosome XIV	S000005063	YNL119W	NCS2	401040	402521	Needs Cla4 to Survive
401213	A	G	54.8661	snp	Chromosome XIV	S000005064	YNL120C		401033	401518	
401341	C	T	54.1002	snp	Chromosome XIV	S000005063	YNL119W	NCS2	401040	402521	Needs Cla4 to Survive
401341	C	T	54.1002	snp	Chromosome XIV	S000005064	YNL120C		401033	401518	
405235	T	C	55.9635	snp	Chromosome XIV	S000005062	YNL118C	DCP2	402652	405564	mRNA DeCaPping
419960	A	T	54.6922	snp	Chromosome XIV	intergenic					
427171	G	A	55.5807	snp	Chromosome XIV	intergenic					
432659	A	T	56.204	snp	Chromosome XIV	S000005046	YNL102W	POL1	430087	434493	POLymerase
439397	GTTTTTTC	GTTTTTTTC	56.2849	ins	Chromosome XIV	intergenic		RAS2			
439397	GTTTTTTC	GTTTTTTTC	56.2849	ins	Chromosome XIV	intergenic					
454073	G	T	55.7364	snp	Chromosome XIV	S000005035	YNL091W	NST1	452408	456130	Negatively affects Salt Tolerance
457793	T	A	55.9635	snp	Chromosome XIV	S000005032	YNL088W	TOP2	457704	461990	TOPoisomerase
464638	A	T	54.8661	snp	Chromosome XIV	S000005031	YNL087W	TCB2	462411	465947	Three Calcium and lipid Binding domains (TriCalBins)
477968	G	A	55.4847	snp	Chromosome XIV	S000005024	YNL080C	EOS1	476932	478032	ER-localized and Oxidants Sensitive
511125	A	G	56.204	snp	Chromosome XIV	S000005005	YNL061W	NOP2	510540	512396	NucleOlar Protein

516162	TGG	TG	54.5182	del	Chromosome XIV	S000005003	YNL058C		515763	516713	
534087	T	C	55.5807	snp	Chromosome XIV	S000004995	YNL050C		534079	534982	
577821	T	A	56.204	snp	Chromosome XIV	S000004974	YNL029C	KTR5	577205	578773	Kre Two Related
596591	T	C	54.8661	snp	Chromosome XIV	S000004965	YNL020C	ARK1	595623	597539	Actin Regulating Kinase
624496	G	C	54.5182	snp	Chromosome XIV	S000004949	YNL004W	HRB1	622915	624621	Hypothetical RNA-Binding protein
639256	A	T	54.7444	snp	Chromosome XIV	S000005290	YNR007C	ATG3	639182	640114	AuTophaGy related
639265	C	T	54.7086	snp	Chromosome XIV	S000005290	YNR007C	ATG3	639182	640114	AuTophaGy related
639868	A	T	54.3092	snp	Chromosome XIV	S000005290	YNR007C	ATG3	639182	640114	AuTophaGy related
653096	A	G	56.1101	snp	Chromosome XIV	S000005297	YNR014W		652465	653103	
662701	A	C	54.8661	snp	Chromosome XIV	intergenic					
706311	T	A	54.5182	snp	Chromosome XIV	S000005328	YNR045W	PET494	706139	707608	PETite colonies
720164	C	A	54.5182	snp	Chromosome XIV	S000005335	YNR052C	POP2	719346	720647	PGK promoter directed OverProduction
724374	T	C	55.1533	snp	Chromosome XIV	intergenic					
757746	C	A	55.7364	snp	Chromosome XIV	S000005350	YNR067C	DSE4	755746	759099	Daughter Specific Expression
47594	A	G	55.7364	snp	Chromosome XV	intergenic					
60240	C	T	55.0097	snp	Chromosome XV	intergenic					
68294	A	T	55.9635	snp	Chromosome XV	S000005496	YOL136C	PFK27	67561	68754	6-PhosphoFructo-2-Kinase
79297	C	G	56.204	snp	Chromosome XV	S000005488	YOL128C	YGK3	78352	79479	Yeast homolog of Glycogen synthase Kinase
99505	G	A	54.8661	snp	Chromosome XV	intergenic					
159104	CA	TG	55.7364	mnp	Chromosome XV	intergenic					
164537	G	T	56.1623	snp	Chromosome XV	S000005444	YOL084W	PHM7	162356	165331	PHosphate Metabolism

173153	C	G	55.1533	snp	Chromosome XV	S000005441	YOL081W	IRA2	171070	180309	Inhibitory Regulator of the RAS-cAMP pathway
181829	T	C	54.5182	snp	Chromosome XV	S000005438	YOL078W	AVO1	181682	185212	Adheres VOraciously (to TOR2)
196047	C	A	55.1533	snp	Chromosome XV	S000005433	YOL072W	THP1	194970	196337	Tho2/Hpr1 Phenotype
197261	T	A	55.5807	snp	Chromosome XV	S000005431	YOL070C	NBA1	197220	198725	Nap1p and Bud neck Associated
211969	T	C	54.1002	snp	Chromosome XV	S000005423	YOL062C	APM4	210520	211995	clathrin Adaptor Protein complex Medium chain
221800	A	T	54.1002	snp	Chromosome XV	S000005418	YOL057W		220767	222902	
226691	T	A	55.3887	snp	Chromosome XV	intergenic					
234936	T	C	55.9635	snp	Chromosome XV	intergenic					
238963	A	G	54.6922	snp	Chromosome XV	S000005409	YOL049W	GSH2	238619	240094	Glutathione
245273	G	T	55.1533	snp	Chromosome XV	S000005405	YOL045W	PSK2	243497	246802	Pas domain-containing Serine/threonine protein Kinase
252656	T	A	54.8661	snp	Chromosome XV	intergenic					
322411	T	C	55.1533	snp	Chromosome XV	S000005363	YOL003C	PFA4	321858	322994	Protein Fatty Acyltransferase
347324	G	A	56.3017	snp	Chromosome XV	intergenic		TIR2			Putative cell wall mannoprotein
365653	A	G	55.8622	snp	Chromosome XV	S000005544	YOR018W	ROD1	364369	366882	Resistance to O-Dinitrobenzene
407139	A	T	54.3092	snp	Chromosome XV	S000005566	YOR040W	GLO4	407064	407921	GLyOxalase
433195	G	A	54.5182	snp	Chromosome XV	S000005583	YOR057W	SGT1	432186	433373	Suppressor of G2 (Two) allele of skp1
434833	C	A	56.1101	snp	Chromosome XV	S000005584	YOR058C	ASE1	433688	436345	Anaphase Spindle Elongation
489475	C	A	54.8661	snp	Chromosome XV	S000005613	YOR087W	YVC1	487707	489734	Yeast Vacuolar Conductance
489475	C	A	54.8661	snp	Chromosome XV	S000005614	YOR088W		488286	489734	
495095	A	G	56.2849	snp	Chromosome XV	intergenic					
496062	C	A	55.1533	snp	Chromosome XV	S000005618	YOR092W	ECM3	495127	496968	ExtraCellular Mutant

517146	G	A	56.0449	snp	Chromosome XV	intergenic					
533330	G	T	55.1533	snp	Chromosome XV	S000005638	YOR112W	CEX1	531508	533793	Cytoplasmic EXport protein
565547	C	T	54.8661	snp	Chromosome XV	S000005654	YOR128C	ADE2	564476	566191	ADEnine requiring
582132	C	A	55.1533	snp	Chromosome XV	S000005663	YOR137C	SIA1	581813	583681	Suppressor of eIF5A
593934	G	T	54.8661	snp	Chromosome XV	S000005668	YOR142W	LSC1	593057	594046	Ligase of Succinyl-CoA
606634	A	T	56.2849	snp	Chromosome XV	S000005673	YOR147W	MDM32	606607	608475	Mitochondrial Distribution and Morphology
614649	C	A	55.3887	snp	Chromosome XV	S000005677	YOR151C	RPB2	612997	616671	RNA Polymerase B
656227	A	T	54.5182	snp	Chromosome XV	S000005698	YOR172W	YRM1	654210	656570	Yeast Reveromycin resistance Modulator
656729	A	T	56.0449	snp	Chromosome XV	intergenic					
682518	T	A	55.7364	snp	Chromosome XV	intergenic		GSP2			
685250	C	A	56.1101	snp	Chromosome XV	S000005713	YOR187W	TUF1	684030	685343	
697641	T	C	56.1623	snp	Chromosome XV	intergenic					
726808	C	T	54.8661	snp	Chromosome XV	S000005731	YOR205C	GEP3	725564	727234	GEnetic interactors of Prohibitins
787882	G	A	56.1623	snp	Chromosome XV	S000005767	YOR241W	MET7	786995	788641	METhionine requiring
797371	A	C	54.6922	snp	Chromosome XV	intergenic					
806210	A	T	54.8661	snp	Chromosome XV	S000005780	YOR254C	SEC63	805032	807023	SECretory
810081	G	T	55.5807	snp	Chromosome XV	S000005782	YOR256C	TRE2	808254	810683	Transferrrin REceptor like
828199	A	G	56.3017	snp	Chromosome XV	S000005796	YOR270C	VPH1	828052	830574	Vacuolar pH
834039	T	C	55.7364	snp	Chromosome XV	S000005798	YOR272W	YTM1	832813	834195	
859384	CTT	CT	55.5807	del	Chromosome XV	S000005816	YOR290C	SNF2	855147	860258	Sucrose NonFermenting
881875	G	T	55.1533	snp	Chromosome XV	S000005827	YOR301W	RAX1	880965	882272	Revert to Axial
893847	TAT	TT	54.5182	del	Chromosome XV	S000005834	YOR307C	SLY41	892731	894092	Suppressor of Loss of Ypt1

934020	T	C	55.1533	snp	Chromosome XV	S000005855	YOR328W	PDR10	931803	936497	Pleiotropic Drug Resistance
946570	G	A	55.3887	snp	Chromosome XV	S000005862	YOR335C	ALA1	946233	949109	ALAnyl-tRNA synthetase
946570	G	A	55.3887	snp	Chromosome XV	S000028717	YOR335W-A		946568	946648	
954011	C	T	55.9635	snp	Chromosome XV	intergenic					
1003695	G	A	54.8661	snp	Chromosome XV	intergenic					
49394	TGG	TG	56.204	del	Chromosome XVI	S000006181	YPL260W	CUB1	49303	50958	Cu2+ suppressing and Bleomycin sensitive
67949	TGG	TG	54.1002	del	Chromosome XVI	S000006176	YPL255W	BBP1	67725	68882	Bfr1 Binding Protein
70105	C	A	54.8661	snp	Chromosome XVI	S000006175	YPL254W	HFI1	69485	70951	Histone H2A Functional Interactor
117451	T	A	56.1101	snp	Chromosome XVI	S000006150	YPL229W		117067	117687	
140736	C	A	55.9635	snp	Chromosome XVI	S000006138	YPL217C	BMS1	139620	143171	BMh Sensitive
178153	G	A	54.1002	snp	Chromosome XVI	S000006116	YPL195W	APL5	176223	179021	clathrin Adaptor Protein complex Large chain
184039	T	C	55.1533	snp	Chromosome XVI	S000006112	YPL191C		183597	184679	
185176	G	T	54.1002	snp	Chromosome XVI	intergenic					
195074	G	A	55.1533	snp	Chromosome XVI	S000006107	YPL186C	UIP4	194512	195426	Ulp1 Interacting Protein
215080	A	G	54.5182	snp	Chromosome XVI	intergenic					
335401	T	A	55.8622	snp	Chromosome XVI	S000006036	YPL115C	BEM3	332100	335486	Bud EMergence
370388	T	A	55.7721	snp	Chromosome XVI	S000006015	YPL094C	SEC62	369839	370663	SECretory
378990	T	C	54.1002	snp	Chromosome XVI	intergenic					
385188	A	T	56.157	snp	Chromosome XVI	S000006007	YPL086C	ELP3	384773	386446	ELongator Protein
403221	A	G	56.2849	snp	Chromosome XVI	S000006003	YPL082C	MOT1	398480	404083	Modifier of Transcription

422512	C	A	54.3092	snp	Chromosome XVI	S000005991	YPL070W	MUK1	420948	422786	coMpUtationally-linked to Kap95
435979	T	A	54.1002	snp	Chromosome XVI	intergenic					
482299	G	T	55.7993	snp	Chromosome XVI	intergenic					
517285	T	A	55.1533	snp	Chromosome XVI	intergenic					
579720	A	T	56.2849	snp	Chromosome XVI	S000006214	YPR010C	RPA135	577585	581196	RNA Polymerase A
580699	C	A	55.3887	snp	Chromosome XVI	S000006214	YPR010C	RPA135	577585	581196	RNA Polymerase A
599333	C	A	55.5807	snp	Chromosome XVI	S000006223	YPR019W	MCM4	596750	599551	MiniChromosome Maintenance
612501	C	T	55.9635	snp	Chromosome XVI	S000006228	YPR024W	YME1	610481	612724	Yeast Mitochondrial Escape
623342	C	G	55.1533	snp	Chromosome XVI	intergenic					
636877	GCA	GA	55.1521	del	Chromosome XVI	S000006236	YPR032W	SRO7	634123	637224	Suppressor of rho3
638771	G	A	54.1002	snp	Chromosome XVI	S000006237	YPR033C	HTS1	637379	639019	Histidine-Trna Synthetase
646838	G	T	55.5807	snp	Chromosome XVI	S000006241	YPR037C	ERV2	646448	647038	Essential for Respiration and Viability
646838	G	T	55.5807	snp	Chromosome XVI	S000006242	YPR038W	IRC16	646836	647195	Increased Recombination Centers
737036	T	A	54.6922	snp	Chromosome XVI	S000006309	YPR105C	COG4	736984	739569	Conserved Oligomeric Golgi complex
738667	G	A	54.5182	snp	Chromosome XVI	S000006309	YPR105C	COG4	736984	739569	Conserved Oligomeric Golgi complex
759400	T	G	55.9635	snp	Chromosome XVI	S000006320	YPR116W	RRG8	758648	759481	Required for Respiratory Growth
759400	T	G	55.9635	snp	Chromosome XVI	S000006320	YPR116W	RRG8	758648	759481	Required for Respiratory Growth
785994	G	T	55.1533	snp	Chromosome XVI	intergenic					
792146	G	C	55.9635	snp	Chromosome XVI	S000006332	YPR128C	ANT1	791218	792204	Adenine Nucleotide Transporter
801316	C	T	54.1002	snp	Chromosome XVI	S000006339	YPR135W	CTF4	799234	802017	Chromosome Transmission Fidelity

804613	C	T	56.0775	snp	Chromosome XVI	intergenic					
823928	C	A	54.1002	snp	Chromosome XVI	S000006349	YPR145W	ASN1	822620	824338	ASparagiNe requiring
1687	C	T	55.1533	snp	mitochondrion MT	intergenic					
2820	T	C	54.8661	snp	mitochondrion MT	intergenic					
6541	G	C	54.5182	snp	mitochondrion MT	intergenic					
7352	A	T	54.8661	snp	mitochondrion MT	intergenic					
7395	G	A	55.3887	snp	mitochondrion MT	intergenic					
10784	G	T	56.1362	snp	mitochondrion MT	intergenic					
14072	C	A	55.8622	snp	mitochondrion MT	S000007260	Q0045	COX1	13818	26701	Cytochrome c OXidase
14072	C	A	55.8622	snp	mitochondrion MT	S000007261	Q0050	AI1	13818	16322	
14072	C	A	55.8622	snp	mitochondrion MT	S000007262	Q0055	AI2	13818	18830	
14072	C	A	55.8622	snp	mitochondrion MT	S000007263	Q0060	AI3	13818	19996	
14072	C	A	55.8622	snp	mitochondrion MT	S000007264	Q0065	AI4	13818	21935	
14072	C	A	55.8622	snp	mitochondrion MT	S000007265	Q0070	AI5_ALPHA	13818	23167	
14433	T	A	55.5807	snp	mitochondrion MT	S000007260	Q0045	COX1	13818	26701	Cytochrome c OXidase
14433	T	A	55.5807	snp	mitochondrion MT	S000007261	Q0050	AI1	13818	16322	
14433	T	A	55.5807	snp	mitochondrion MT	S000007262	Q0055	AI2	13818	18830	
14433	T	A	55.5807	snp	mitochondrion MT	S000007263	Q0060	AI3	13818	19996	
14433	T	A	55.5807	snp	mitochondrion MT	S000007264	Q0065	AI4	13818	21935	
14433	T	A	55.5807	snp	mitochondrion MT	S000007265	Q0070	AI5_ALPHA	13818	23167	

14764	C	T	54.1002	snp	mitochondrion MT	S000007260	Q0045	COX1	13818	26701	Cytochrome c OXidase
14764	C	T	54.1002	snp	mitochondrion MT	S000007261	Q0050	AI1	13818	16322	
14764	C	T	54.1002	snp	mitochondrion MT	S000007262	Q0055	AI2	13818	18830	
14764	C	T	54.1002	snp	mitochondrion MT	S000007263	Q0060	AI3	13818	19996	
14764	C	T	54.1002	snp	mitochondrion MT	S000007264	Q0065	AI4	13818	21935	
14764	C	T	54.1002	snp	mitochondrion MT	S000007265	Q0070	AI5_ALPHA	13818	23167	
15335	A	G	55.1533	snp	mitochondrion MT	S000007260	Q0045	COX1	13818	26701	Cytochrome c OXidase
15335	A	G	55.1533	snp	mitochondrion MT	S000007261	Q0050	AI1	13818	16322	
15335	A	G	55.1533	snp	mitochondrion MT	S000007262	Q0055	AI2	13818	18830	
15335	A	G	55.1533	snp	mitochondrion MT	S000007263	Q0060	AI3	13818	19996	
15335	A	G	55.1533	snp	mitochondrion MT	S000007264	Q0065	AI4	13818	21935	
15335	A	G	55.1533	snp	mitochondrion MT	S000007265	Q0070	AI5_ALPHA	13818	23167	
15415	CAAAAAAT	CAAAAAT	55.1533	del	mitochondrion MT	S000007260	Q0045	COX1	13818	26701	Cytochrome c OXidase
15415	CAAAAAAT	CAAAAAT	55.1533	del	mitochondrion MT	S000007261	Q0050	AI1	13818	16322	
15415	CAAAAAAT	CAAAAAT	55.1533	del	mitochondrion MT	S000007262	Q0055	AI2	13818	18830	
15415	CAAAAAAT	CAAAAAT	55.1533	del	mitochondrion MT	S000007263	Q0060	AI3	13818	19996	
15415	CAAAAAAT	CAAAAAT	55.1533	del	mitochondrion MT	S000007264	Q0065	AI4	13818	21935	
15415	CAAAAAAT	CAAAAAT	55.1533	del	mitochondrion MT	S000007265	Q0070	AI5_ALPHA	13818	23167	
16068	G	T	55.3887	snp	mitochondrion MT	S000007260	Q0045	COX1	13818	26701	Cytochrome c OXidase
16068	G	T	55.3887	snp	mitochondrion MT	S000007261	Q0050	AI1	13818	16322	

16068	G	T	55.3887	snp	mitochondrion MT	S000007262	Q0055	AI2	13818	18830	
16068	G	T	55.3887	snp	mitochondrion MT	S000007263	Q0060	AI3	13818	19996	
16068	G	T	55.3887	snp	mitochondrion MT	S000007264	Q0065	AI4	13818	21935	
16068	G	T	55.3887	snp	mitochondrion MT	S000007265	Q0070	AI5_ALPHA	13818	23167	
16524	A	G	55.1533	snp	mitochondrion MT	S000007260	Q0045	COX1	13818	26701	Cytochrome c OXidase
16524	A	G	55.1533	snp	mitochondrion MT	S000007262	Q0055	AI2	13818	18830	
16524	A	G	55.1533	snp	mitochondrion MT	S000007263	Q0060	AI3	13818	19996	
16524	A	G	55.1533	snp	mitochondrion MT	S000007264	Q0065	AI4	13818	21935	
16524	A	G	55.1533	snp	mitochondrion MT	S000007265	Q0070	AI5_ALPHA	13818	23167	
16590	T	A	55.1533	snp	mitochondrion MT	S000007260	Q0045	COX1	13818	26701	Cytochrome c OXidase
16590	T	A	55.1533	snp	mitochondrion MT	S000007262	Q0055	AI2	13818	18830	
16590	T	A	55.1533	snp	mitochondrion MT	S000007263	Q0060	AI3	13818	19996	
16590	T	A	55.1533	snp	mitochondrion MT	S000007264	Q0065	AI4	13818	21935	
16590	T	A	55.1533	snp	mitochondrion MT	S000007265	Q0070	AI5_ALPHA	13818	23167	
16922	C	A	55.271	snp	mitochondrion MT	S000007260	Q0045	COX1	13818	26701	Cytochrome c OXidase
16922	C	A	55.1533	snp	mitochondrion MT	S000007262	Q0055	AI2	13818	18830	
16922	C	A	55.1533	snp	mitochondrion MT	S000007263	Q0060	AI3	13818	19996	
16922	C	A	55.1533	snp	mitochondrion MT	S000007264	Q0065	AI4	13818	21935	
16922	C	A	55.1533	snp	mitochondrion MT	S000007265	Q0070	AI5_ALPHA	13818	23167	
17268	A	T	55.1533	snp	mitochondrion MT	S000007260	Q0045	COX1	13818	26701	Cytochrome c OXidase

17268	A	T	55.1533	snp	mitochondrion MT	S000007262	Q0055	AI2	13818	18830	
17268	A	T	55.1533	snp	mitochondrion MT	S000007263	Q0060	AI3	13818	19996	
17268	A	T	55.1533	snp	mitochondrion MT	S000007264	Q0065	AI4	13818	21935	
17268	A	T	55.1533	snp	mitochondrion MT	S000007265	Q0070	AI5_ALPHA	13818	23167	
17726	A	G	55.1533	snp	mitochondrion MT	S000007260	Q0045	COX1	13818	26701	Cytochrome c OXidase
17726	A	G	55.1533	snp	mitochondrion MT	S000007262	Q0055	AI2	13818	18830	
17726	A	G	55.1533	snp	mitochondrion MT	S000007263	Q0060	AI3	13818	19996	
17726	A	G	55.1533	snp	mitochondrion MT	S000007264	Q0065	AI4	13818	21935	
17726	A	G	55.1533	snp	mitochondrion MT	S000007265	Q0070	AI5_ALPHA	13818	23167	
17915	T	A	56.1101	snp	mitochondrion MT	S000007260	Q0045	COX1	13818	26701	Cytochrome c OXidase
17915	T	A	56.1101	snp	mitochondrion MT	S000007262	Q0055	AI2	13818	18830	
17915	T	A	56.1101	snp	mitochondrion MT	S000007263	Q0060	AI3	13818	19996	
17915	T	A	56.1101	snp	mitochondrion MT	S000007264	Q0065	AI4	13818	21935	
17915	T	A	56.1101	snp	mitochondrion MT	S000007265	Q0070	AI5_ALPHA	13818	23167	
18280	G	T	55.9635	snp	mitochondrion MT	S000007260	Q0045	COX1	13818	26701	Cytochrome c OXidase
18280	G	T	55.9129	snp	mitochondrion MT	S000007262	Q0055	AI2	13818	18830	
18280	G	T	55.9129	snp	mitochondrion MT	S000007263	Q0060	AI3	13818	19996	
18280	G	T	55.9129	snp	mitochondrion MT	S000007264	Q0065	AI4	13818	21935	
18280	G	T	55.8906	snp	mitochondrion MT	S000007265	Q0070	AI5_ALPHA	13818	23167	
18532	T	A	55.8622	snp	mitochondrion MT	S000007260	Q0045	COX1	13818	26701	Cytochrome c OXidase

18532	T	A	55.7993	snp	mitochondrion MT	S000007262	Q0055	AI2	13818	18830	
18532	T	A	55.7993	snp	mitochondrion MT	S000007263	Q0060	AI3	13818	19996	
18532	T	A	55.7993	snp	mitochondrion MT	S000007264	Q0065	AI4	13818	21935	
18532	T	A	55.7993	snp	mitochondrion MT	S000007265	Q0070	AI5_ALPHA	13818	23167	
18643	G	A	56.204	snp	mitochondrion MT	S000007260	Q0045	COX1	13818	26701	Cytochrome c OXidase
18643	G	A	56.204	snp	mitochondrion MT	S000007262	Q0055	AI2	13818	18830	
18643	G	A	56.204	snp	mitochondrion MT	S000007263	Q0060	AI3	13818	19996	
18643	G	A	56.204	snp	mitochondrion MT	S000007264	Q0065	AI4	13818	21935	
18643	G	A	56.204	snp	mitochondrion MT	S000007265	Q0070	AI5_ALPHA	13818	23167	
18960	T	A	54.5182	snp	mitochondrion MT	S000007260	Q0045	COX1	13818	26701	Cytochrome c OXidase
18960	T	A	54.4955	snp	mitochondrion MT	S000007263	Q0060	AI3	13818	19996	
18960	T	A	54.3206	snp	mitochondrion MT	S000007264	Q0065	AI4	13818	21935	
18960	T	A	54.3092	snp	mitochondrion MT	S000007265	Q0070	AI5_ALPHA	13818	23167	
19814	T	A	55.9635	snp	mitochondrion MT	S000007260	Q0045	COX1	13818	26701	Cytochrome c OXidase
19814	T	A	55.9635	snp	mitochondrion MT	S000007263	Q0060	AI3	13818	19996	
19814	T	A	55.9635	snp	mitochondrion MT	S000007264	Q0065	AI4	13818	21935	
19814	T	A	55.9635	snp	mitochondrion MT	S000007265	Q0070	AI5_ALPHA	13818	23167	
20044	G	A	55.1533	snp	mitochondrion MT	S000007260	Q0045	COX1	13818	26701	Cytochrome c OXidase
20044	G	A	55.1533	snp	mitochondrion MT	S000007264	Q0065	AI4	13818	21935	
20044	G	A	55.1533	snp	mitochondrion MT	S000007265	Q0070	AI5_ALPHA	13818	23167	

20172	C	T	55.1533	snp	mitochondrion MT	S000007260	Q0045	COX1	13818	26701	Cytochrome c OXidase
20172	C	T	55.1533	snp	mitochondrion MT	S000007264	Q0065	AI4	13818	21935	
20172	C	T	55.1533	snp	mitochondrion MT	S000007265	Q0070	AI5_ALPHA	13818	23167	
20345	GAA	GA	55.7364	del	mitochondrion MT	S000007260	Q0045	COX1	13818	26701	Cytochrome c OXidase
20345	GAA	GA	55.7364	del	mitochondrion MT	S000007264	Q0065	AI4	13818	21935	
20345	GAA	GA	55.7364	del	mitochondrion MT	S000007265	Q0070	AI5_ALPHA	13818	23167	
20345	GAA	GA	55.7364	del	mitochondrion MT	S000007260	Q0045	COX1	13818	26701	Cytochrome c OXidase
20345	GAA	GA	55.7364	del	mitochondrion MT	S000007264	Q0065	AI4	13818	21935	
20345	GAA	GA	55.7364	del	mitochondrion MT	S000007265	Q0070	AI5_ALPHA	13818	23167	
20345	GAA	GA	55.7364	del	mitochondrion MT	S000007260	Q0045	COX1	13818	26701	Cytochrome c OXidase
20345	GAA	GA	55.7214	del	mitochondrion MT	S000007264	Q0065	AI4	13818	21935	
20345	GAA	GA	55.7214	del	mitochondrion MT	S000007265	Q0070	AI5_ALPHA	13818	23167	
20345	GAA	GA	55.6761	del	mitochondrion MT	S000007260	Q0045	COX1	13818	26701	Cytochrome c OXidase
20345	GAA	GA	55.6585	del	mitochondrion MT	S000007264	Q0065	AI4	13818	21935	
20345	GAA	GA	55.6585	del	mitochondrion MT	S000007265	Q0070	AI5_ALPHA	13818	23167	
20345	GAA	GA	55.5807	del	mitochondrion MT	S000007260	Q0045	COX1	13818	26701	Cytochrome c OXidase
20345	GAA	GA	55.5807	del	mitochondrion MT	S000007264	Q0065	AI4	13818	21935	
20345	GAA	GA	55.5807	del	mitochondrion MT	S000007265	Q0070	AI5_ALPHA	13818	23167	
20431	TGT	TT	55.1533	del	mitochondrion MT	S000007260	Q0045	COX1	13818	26701	Cytochrome c OXidase
20431	TGT	TT	55.1533	del	mitochondrion MT	S000007264	Q0065	AI4	13818	21935	

20431	TGT	TT	55.1533	del	mitochondrion MT	S000007265	Q0070	AI5_ALPHA	13818	23167	
20555	A	T	55.8622	snp	mitochondrion MT	S000007260	Q0045	COX1	13818	26701	Cytochrome c OXidase
20555	A	T	55.8622	mnr2	mitochondrion MT	S000007264	Q0065	AI4	13818	21935	
20555	A	T	55.8622	snp	mitochondrion MT	S000007265	Q0070	AI5_ALPHA	13818	23167	
20565	A	G	55.9635	snp	mitochondrion MT	S000007260	Q0045	COX1	13818	26701	Cytochrome c OXidase
20565	A	G	55.9635	snp	mitochondrion MT	S000007264	Q0065	AI4	13818	21935	
20565	A	G	55.9635	snp	mitochondrion MT	S000007265	Q0070	AI5_ALPHA	13818	23167	
20799	A	G	55.8622	snp	mitochondrion MT	S000007260	Q0045	COX1	13818	26701	Cytochrome c OXidase
20799	A	G	55.8622	snp	mitochondrion MT	S000007264	Q0065	AI4	13818	21935	
20799	A	G	55.8622	snp	mitochondrion MT	S000007265	Q0070	AI5_ALPHA	13818	23167	
20846	CTTATTATTATTAT	CTTATTATTAT	56.1831	del	mitochondrion MT	S000007260	Q0045	COX1	13818	26701	Cytochrome c OXidase
20846	CTTATTATTATTAT	CTTATTATTAT	56.1623	del	mitochondrion MT	S000007264	Q0065	AI4	13818	21935	
20846	CTTATTATTATTAT	CTTATTATTAT	56.1623	del	mitochondrion MT	S000007265	Q0070	AI5_ALPHA	13818	23167	
20847	T	A	56.1623	snp	mitochondrion MT	S000007260	Q0045	COX1	13818	26701	Cytochrome c OXidase
20847	T	A	56.1623	snp	mitochondrion MT	S000007264	Q0065	AI4	13818	21935	
20847	T	A	56.1623	snp	mitochondrion MT	S000007265	Q0070	AI5_ALPHA	13818	23167	
20934	T	G	56.0449	snp	mitochondrion MT	S000007260	Q0045	COX1	13818	26701	Cytochrome c OXidase
20934	T	G	56.0449	snp	mitochondrion MT	S000007264	Q0065	AI4	13818	21935	
20934	T	G	56.0449	snp	mitochondrion MT	S000007265	Q0070	AI5_ALPHA	13818	23167	
20934	T	G	56.0449	snp	mitochondrion MT	S000007260	Q0045	COX1	13818	26701	Cytochrome c OXidase

20934	T	G	56.0449	snp	mitochondrion MT	S000007264	Q0065	AI4	13818	21935	
20934	T	G	56.0449	snp	mitochondrion MT	S000007265	Q0070	AI5_ALPHA	13818	23167	
20934	T	G	56.0449	snp	mitochondrion MT	S000007260	Q0045	COX1	13818	26701	Cytochrome c OXidase
20934	T	G	56.0449	snp	mitochondrion MT	S000007264	Q0065	AI4	13818	21935	
20934	T	G	56.0449	snp	mitochondrion MT	S000007265	Q0070	AI5_ALPHA	13818	23167	
20934	T	G	56.0449	snp	mitochondrion MT	S000007260	Q0045	COX1	13818	26701	Cytochrome c OXidase
20934	T	G	56.0449	snp	mitochondrion MT	S000007264	Q0065	AI4	13818	21935	
20934	T	G	56.0449	snp	mitochondrion MT	S000007265	Q0070	AI5_ALPHA	13818	23167	
20934	TCAGG	GCAGT	56.0449	complex	mitochondrion MT	S000007260	Q0045	COX1	13818	26701	Cytochrome c OXidase
20934	TCAGG	GCAGT	56.0449	complex	mitochondrion MT	S000007264	Q0065	AI4	13818	21935	
20934	TCAGG	GCAGT	56.0449	complex	mitochondrion MT	S000007265	Q0070	AI5_ALPHA	13818	23167	
20934	T	G	56.0449	snp	mitochondrion MT	S000007260	Q0045	COX1	13818	26701	Cytochrome c OXidase
20934	T	G	56.0449	snp	mitochondrion MT	S000007264	Q0065	AI4	13818	21935	
20934	T	G	56.0449	snp	mitochondrion MT	S000007265	Q0070	AI5_ALPHA	13818	23167	
20934	T	G	56.0449	snp	mitochondrion MT	S000007260	Q0045	COX1	13818	26701	Cytochrome c OXidase
20934	T	G	56.0449	snp	mitochondrion MT	S000007264	Q0065	AI4	13818	21935	
20934	T	G	56.0449	snp	mitochondrion MT	S000007265	Q0070	AI5_ALPHA	13818	23167	
20934	T	G	56.0449	snp	mitochondrion MT	S000007260	Q0045	COX1	13818	26701	Cytochrome c OXidase
20934	T	G	56.0449	snp	mitochondrion MT	S000007264	Q0065	AI4	13818	21935	
20934	T	G	56.0449	snp	mitochondrion MT	S000007265	Q0070	AI5_ALPHA	13818	23167	

20934	T	G	56.0449	snp	mitochondrion MT	S000007260	Q0045	COX1	13818	26701	Cytochrome c OXidase
20934	T	G	56.0449	snp	mitochondrion MT	S000007264	Q0065	AI4	13818	21935	
20934	T	G	56.0449	snp	mitochondrion MT	S000007265	Q0070	AI5_ALPHA	13818	23167	
20934	T	G	56.0449	snp	mitochondrion MT	S000007260	Q0045	COX1	13818	26701	Cytochrome c OXidase
20934	T	G	56.0449	snp	mitochondrion MT	S000007264	Q0065	AI4	13818	21935	
20934	T	G	56.0449	snp	mitochondrion MT	S000007265	Q0070	AI5_ALPHA	13818	23167	
20934	T	G	56.0449	snp	mitochondrion MT	S000007260	Q0045	COX1	13818	26701	Cytochrome c OXidase
20934	T	G	56.0449	snp	mitochondrion MT	S000007264	Q0065	AI4	13818	21935	
20934	T	G	56.0449	snp	mitochondrion MT	S000007265	Q0070	AI5_ALPHA	13818	23167	
20934	T	G	56.0449	snp	mitochondrion MT	S000007260	Q0045	COX1	13818	26701	Cytochrome c OXidase
20934	T	G	56.0449	snp	mitochondrion MT	S000007264	Q0065	AI4	13818	21935	
20934	T	G	56.0449	snp	mitochondrion MT	S000007265	Q0070	AI5_ALPHA	13818	23167	
20934	T	G	56.0449	snp	mitochondrion MT	S000007260	Q0045	COX1	13818	26701	Cytochrome c OXidase
20934	T	G	56.0449	snp	mitochondrion MT	S000007264	Q0065	AI4	13818	21935	
20934	T	G	56.0449	snp	mitochondrion MT	S000007265	Q0070	AI5_ALPHA	13818	23167	
20934	T	G	56.0449	snp	mitochondrion MT	S000007260	Q0045	COX1	13818	26701	Cytochrome c OXidase
20934	T	G	56.0449	snp	mitochondrion MT	S000007264	Q0065	AI4	13818	21935	
20934	T	G	56.0449	snp	mitochondrion MT	S000007265	Q0070	AI5_ALPHA	13818	23167	
20934	T	G	56.0449	snp	mitochondrion MT	S000007260	Q0045	COX1	13818	26701	Cytochrome c OXidase
20934	T	G	56.0449	snp	mitochondrion MT	S000007264	Q0065	AI4	13818	21935	

20934	T	G	56.0449	snp	mitochondrion MT	S000007265	Q0070	AI5_ALPHA	13818	23167	
20934	T	G	56.0449	snp	mitochondrion MT	S000007260	Q0045	COX1	13818	26701	Cytochrome c OXidase
20934	T	G	56.0449	snp	mitochondrion MT	S000007264	Q0065	AI4	13818	21935	
20934	T	G	56.0449	snp	mitochondrion MT	S000007265	Q0070	AI5_ALPHA	13818	23167	
20934	T	G	56.0449	snp	mitochondrion MT	S000007260	Q0045	COX1	13818	26701	Cytochrome c OXidase
20934	T	G	56.0368	snp	mitochondrion MT	S000007264	Q0065	AI4	13818	21935	
20934	T	G	56.0331	snp	mitochondrion MT	S000007265	Q0070	AI5_ALPHA	13818	23167	
21172	A	T	54.1002	snp	mitochondrion MT	S000007260	Q0045	COX1	13818	26701	Cytochrome c OXidase
21172	A	T	54.1002	snp	mitochondrion MT	S000007264	Q0065	AI4	13818	21935	
21172	A	T	54.1002	snp	mitochondrion MT	S000007265	Q0070	AI5_ALPHA	13818	23167	
21895	A	G	56.1623	snp	mitochondrion MT	S000007260	Q0045	COX1	13818	26701	Cytochrome c OXidase
21895	A	G	56.1623	snp	mitochondrion MT	S000007264	Q0065	AI4	13818	21935	
21895	A	G	56.1623	snp	mitochondrion MT	S000007265	Q0070	AI5_ALPHA	13818	23167	
22062	C	T	55.7364	snp	mitochondrion MT	S000007260	Q0045	COX1	13818	26701	Cytochrome c OXidase
22062	C	T	55.7364	snp	mitochondrion MT	S000007265	Q0070	AI5_ALPHA	13818	23167	
22200	T	A	56.204	snp	mitochondrion MT	S000007260	Q0045	COX1	13818	26701	Cytochrome c OXidase
22200	T	A	56.204	snp	mitochondrion MT	S000007265	Q0070	AI5_ALPHA	13818	23167	
22236	T	C	55.8622	snp	mitochondrion MT	S000007260	Q0045	COX1	13818	26701	Cytochrome c OXidase
22236	T	C	55.8622	snp	mitochondrion MT	S000007265	Q0070	AI5_ALPHA	13818	23167	
22593	T	A	54.1002	snp	mitochondrion MT	S000007260	Q0045	COX1	13818	26701	Cytochrome c OXidase

22593	T	A	54.1002	snp	mitochondrion MT	S000007265	Q0070	AI5_ALPHA	13818	23167	
22891	T	A	55.3887	snp	mitochondrion MT	S000007260	Q0045	COX1	13818	26701	Cytochrome c OXidase
22891	T	A	55.3887	snp	mitochondrion MT	S000007265	Q0070	AI5_ALPHA	13818	23167	
22907	G	T	54.8661	snp	mitochondrion MT	S000007260	Q0045	COX1	13818	26701	Cytochrome c OXidase
22907	G	T	54.8661	snp	mitochondrion MT	S000007265	Q0070	AI5_ALPHA	13818	23167	
23082	C	A	55.1533	snp	mitochondrion MT	S000007260	Q0045	COX1	13818	26701	Cytochrome c OXidase
23082	C	A	55.1533	snp	mitochondrion MT	S000007265	Q0070	AI5_ALPHA	13818	23167	
23261	A	T	54.1002	snp	mitochondrion MT	S000007260	Q0045	COX1	13818	26701	Cytochrome c OXidase
24373	ATA	ATTA	56.2849	ins	mitochondrion MT	S000007260	Q0045	COX1	13818	26701	Cytochrome c OXidase
24373	ATA	ATTA	56.2849	ins	mitochondrion MT	S000007266	Q0075	AI5_BETA	24156	25255	
24376	TT	TCT	56.2849	ins	mitochondrion MT	S000007260	Q0045	COX1	13818	26701	Cytochrome c OXidase
24376	TT	TCT	56.2849	ins	mitochondrion MT	S000007266	Q0075	AI5_BETA	24156	25255	
24379	ATA	AA	56.2849	del	mitochondrion MT	S000007260	Q0045	COX1	13818	26701	Cytochrome c OXidase
24379	ATA	AA	56.2849	del	mitochondrion MT	S000007266	Q0075	AI5_BETA	24156	25255	
24386	AA	TT	56.2849	mnp	mitochondrion MT	S000007260	Q0045	COX1	13818	26701	Cytochrome c OXidase
24386	AA	TT	56.2849	mnp	mitochondrion MT	S000007266	Q0075	AI5_BETA	24156	25255	
24392	TT	TAT	56.2849	ins	mitochondrion MT	S000007260	Q0045	COX1	13818	26701	Cytochrome c OXidase
24392	TT	TAT	56.2849	ins	mitochondrion MT	S000007266	Q0075	AI5_BETA	24156	25255	
24394	TAGA	TA	56.2849	del	mitochondrion MT	S000007260	Q0045	COX1	13818	26701	Cytochrome c OXidase
24394	TAGA	TA	56.2849	del	mitochondrion MT	S000007266	Q0075	AI5_BETA	24156	25255	

24465	A	G	55.1533	snp	mitochondrion MT	S000007260	Q0045	COX1	13818	26701	Cytochrome c OXidase
24465	A	G	55.1533	snp	mitochondrion MT	S000007266	Q0075	AI5_BETA	24156	25255	
24543	C	A	54.8661	snp	mitochondrion MT	S000007260	Q0045	COX1	13818	26701	Cytochrome c OXidase
24543	C	A	54.8661	snp	mitochondrion MT	S000007266	Q0075	AI5_BETA	24156	25255	
24549	A	G	54.8661	snp	mitochondrion MT	S000007260	Q0045	COX1	13818	26701	Cytochrome c OXidase
24549	A	G	54.8661	snp	mitochondrion MT	S000007266	Q0075	AI5_BETA	24156	25255	
24643	G	T	54.1002	snp	mitochondrion MT	S000007260	Q0045	COX1	13818	26701	Cytochrome c OXidase
24643	G	T	54.1002	snp	mitochondrion MT	S000007266	Q0075	AI5_BETA	24156	25255	
24881	C	A	54.5182	snp	mitochondrion MT	S000007260	Q0045	COX1	13818	26701	Cytochrome c OXidase
24881	C	A	54.5182	snp	mitochondrion MT	S000007266	Q0075	AI5_BETA	24156	25255	
25188	TAAAAAAT	TAAAAAT	55.1533	del	mitochondrion MT	S000007260	Q0045	COX1	13818	26701	Cytochrome c OXidase
25188	TAAAAAAT	TAAAAAT	55.1533	del	mitochondrion MT	S000007266	Q0075	AI5_BETA	24156	25255	
26185	G	T	55.8622	snp	mitochondrion MT	S000007260	Q0045	COX1	13818	26701	Cytochrome c OXidase
26693	C	T	54.5182	snp	mitochondrion MT	S000007260	Q0045	COX1	13818	26701	Cytochrome c OXidase
28157	GCCCCCCCCG	GCCCCCCCCCG	55.9635	ins	mitochondrion MT	intergenic					
28559	C	A	54.1002	snp	mitochondrion MT	S000007268	Q0085	ATP6	28487	29266	ATP synthase
31163	C	A	54.5182	snp	mitochondrion MT	intergenic					
32257	A	T	55.3887	snp	mitochondrion MT	intergenic					
33014	A	G	55.1533	snp	mitochondrion MT	intergenic					
33022	A	G	55.1533	snp	mitochondrion MT	intergenic					

33467	T	C	55.5807	snp	mitochondrion MT	intergenic					
33467	T	C	55.5807	snp	mitochondrion MT	intergenic					
33467	T	C	55.5807	snp	mitochondrion MT	intergenic					
33467	T	C	55.5807	snp	mitochondrion MT	intergenic					
33467	T	C	55.5807	snp	mitochondrion MT	intergenic					
36521	A	C	55.8906	snp	mitochondrion MT	intergenic					
36546	T	A	55.3887	snp	mitochondrion MT	S000007270	Q0105	COB	36540	43647	CytochrOme B
36546	T	A	55.3887	snp	mitochondrion MT	S000007271	Q0110	BI2	36540	38579	
36546	T	A	55.3887	snp	mitochondrion MT	S000007272	Q0115	BI3	36540	40265	
36546	T	A	55.3887	snp	mitochondrion MT	S000007273	Q0120	BI4	36540	42251	
36691	C	T	55.5807	snp	mitochondrion MT	S000007270	Q0105	COB	36540	43647	CytochrOme B
36691	C	T	55.5807	snp	mitochondrion MT	S000007271	Q0110	BI2	36540	38579	
36691	C	T	55.5807	snp	mitochondrion MT	S000007272	Q0115	BI3	36540	40265	
36691	C	T	55.5807	snp	mitochondrion MT	S000007273	Q0120	BI4	36540	42251	
36880	GAA	GA	55.3887	del	mitochondrion MT	S000007270	Q0105	COB	36540	43647	CytochrOme B
36880	GAA	GA	55.3887	del	mitochondrion MT	S000007271	Q0110	BI2	36540	38579	
36880	GAA	GA	55.3887	del	mitochondrion MT	S000007272	Q0115	BI3	36540	40265	
36880	GAA	GA	55.3887	del	mitochondrion MT	S000007273	Q0120	BI4	36540	42251	
36953	G	T	54.8661	snp	mitochondrion MT	S000007270	Q0105	COB	36540	43647	CytochrOme B
36953	G	T	54.8661	snp	mitochondrion MT	S000007271	Q0110	BI2	36540	38579	

36953	G	T	54.8661	snp	mitochondrion MT	S000007272	Q0115	BI3	36540	40265	
36953	G	T	54.8661	snp	mitochondrion MT	S000007273	Q0120	BI4	36540	42251	
38194	A	G	54.1002	snp	mitochondrion MT	S000007270	Q0105	COB	36540	43647	CytochrOme B
38194	A	G	54.1002	snp	mitochondrion MT	S000007271	Q0110	BI2	36540	38579	
38194	A	G	54.1002	snp	mitochondrion MT	S000007272	Q0115	BI3	36540	40265	
38194	A	G	54.1002	snp	mitochondrion MT	S000007273	Q0120	BI4	36540	42251	
38287	C	T	55.3887	snp	mitochondrion MT	S000007270	Q0105	COB	36540	43647	CytochrOme B
38287	C	T	55.3887	snp	mitochondrion MT	S000007271	Q0110	BI2	36540	38579	
38287	C	T	55.3887	snp	mitochondrion MT	S000007272	Q0115	BI3	36540	40265	
38287	C	T	55.3887	snp	mitochondrion MT	S000007273	Q0120	BI4	36540	42251	
38363	A	T	54.1002	snp	mitochondrion MT	S000007270	Q0105	COB	36540	43647	CytochrOme B
38363	A	T	54.1002	snp	mitochondrion MT	S000007271	Q0110	BI2	36540	38579	
38363	A	T	54.1002	snp	mitochondrion MT	S000007272	Q0115	BI3	36540	40265	
38363	A	T	54.1002	snp	mitochondrion MT	S000007273	Q0120	BI4	36540	42251	
39517	G	T	56.2849	snp	mitochondrion MT	S000007270	Q0105	COB	36540	43647	CytochrOme B
39517	G	T	56.2849	snp	mitochondrion MT	S000007272	Q0115	BI3	36540	40265	
39517	G	T	56.2827	snp	mitochondrion MT	S000007273	Q0120	BI4	36540	42251	
39517	G	T	56.2637	snp	mitochondrion MT	S000007270	Q0105	COB	36540	43647	CytochrOme B
39517	G	T	56.2637	snp	mitochondrion MT	S000007272	Q0115	BI3	36540	40265	
39517	G	T	56.2637	snp	mitochondrion MT	S000007273	Q0120	BI4	36540	42251	

39517	G	T	56.2637	snp	mitochondrion MT	S000007270	Q0105	COB	36540	43647	CytochrOme B
39517	G	T	56.2637	snp	mitochondrion MT	S000007272	Q0115	BI3	36540	40265	
39517	G	T	56.2637	snp	mitochondrion MT	S000007273	Q0120	BI4	36540	42251	
39517	G	T	56.2637	snp	mitochondrion MT	S000007270	Q0105	COB	36540	43647	CytochrOme B
39517	G	T	56.2372	snp	mitochondrion MT	S000007272	Q0115	BI3	36540	40265	
39517	G	T	56.2372	snp	mitochondrion MT	S000007273	Q0120	BI4	36540	42251	
39517	G	T	56.2339	snp	mitochondrion MT	S000007270	Q0105	COB	36540	43647	CytochrOme B
39517	G	T	56.204	snp	mitochondrion MT	S000007272	Q0115	BI3	36540	40265	
39517	G	T	56.204	snp	mitochondrion MT	S000007273	Q0120	BI4	36540	42251	
39517	G	T	56.204	snp	mitochondrion MT	S000007270	Q0105	COB	36540	43647	CytochrOme B
39517	G	T	56.204	snp	mitochondrion MT	S000007272	Q0115	BI3	36540	40265	
39517	G	T	56.204	snp	mitochondrion MT	S000007273	Q0120	BI4	36540	42251	
39517	G	T	56.204	snp	mitochondrion MT	S000007270	Q0105	COB	36540	43647	CytochrOme B
39517	G	T	56.204	snp	mitochondrion MT	S000007272	Q0115	BI3	36540	40265	
39517	G	T	56.204	snp	mitochondrion MT	S000007273	Q0120	BI4	36540	42251	
39517	G	T	56.204	snp	mitochondrion MT	S000007270	Q0105	COB	36540	43647	CytochrOme B
39517	G	T	56.204	snp	mitochondrion MT	S000007272	Q0115	BI3	36540	40265	
39517	G	T	56.204	snp	mitochondrion MT	S000007273	Q0120	BI4	36540	42251	
39517	G	T	56.204	snp	mitochondrion MT	S000007270	Q0105	COB	36540	43647	CytochrOme B
39517	G	T	56.204	snp	mitochondrion MT	S000007272	Q0115	BI3	36540	40265	

39517	G	T	56.204	snp	mitochondrion MT	S000007273	Q0120	BI4	36540	42251	
39517	G	T	56.204	snp	mitochondrion MT	S000007270	Q0105	COB	36540	43647	CytochrOme B
39517	G	T	56.204	snp	mitochondrion MT	S000007272	Q0115	BI3	36540	40265	
39517	G	T	56.204	snp	mitochondrion MT	S000007273	Q0120	BI4	36540	42251	
40943	T	A	54.8661	snp	mitochondrion MT	S000007270	Q0105	COB	36540	43647	CytochrOme B
40943	T	A	54.8661	snp	mitochondrion MT	S000007273	Q0120	BI4	36540	42251	
41288	A	G	56.0042	snp	mitochondrion MT	S000007270	Q0105	COB	36540	43647	CytochrOme B
41288	A	G	55.9862	snp	mitochondrion MT	S000007273	Q0120	BI4	36540	42251	
41375	T	C	55.8622	snp	mitochondrion MT	S000007270	Q0105	COB	36540	43647	CytochrOme B
41375	T	C	55.8622	snp	mitochondrion MT	S000007273	Q0120	BI4	36540	42251	
41898	G	T	54.2021	snp	mitochondrion MT	S000007270	Q0105	COB	36540	43647	CytochrOme B
41898	G	T	54.2021	snp	mitochondrion MT	S000007273	Q0120	BI4	36540	42251	
42039	G	T	54.8661	snp	mitochondrion MT	S000007270	Q0105	COB	36540	43647	CytochrOme B
42039	G	T	54.8661	snp	mitochondrion MT	S000007273	Q0120	BI4	36540	42251	
42496	T	A	56.0449	snp	mitochondrion MT	S000007270	Q0105	COB	36540	43647	CytochrOme B
43084	ATTTTTTTTA	ATTTTTTTTTA	56.1623	ins	mitochondrion MT	S000007270	Q0105	COB	36540	43647	CytochrOme B
43084	ATTTTTTTTA	ATTTTTTTTTA	56.1623	ins	mitochondrion MT	S000007270	Q0105	COB	36540	43647	CytochrOme B
43084	ATTTTTTTTA	ATTTTTTTTTA	56.1623	ins	mitochondrion MT	S000007270	Q0105	COB	36540	43647	CytochrOme B
43084	ATTTTTTTTA	ATTTTTTTTTA	56.1623	ins	mitochondrion MT	S000007270	Q0105	COB	36540	43647	CytochrOme B
43084	ATTTTTTTTA	ATTTTTTTTTA	56.1623	ins	mitochondrion MT	S000007270	Q0105	COB	36540	43647	CytochrOme B

43084	ATTTTTTTT A	ATTTTTTTT TA	56.1623	ins	mitochondrio n MT	S000007270	Q0105	COB	36540	43647	CytochrOme B
43084	ATTTTTTTT A	ATTTTTTTT TA	56.1623	ins	mitochondrio n MT	S000007270	Q0105	COB	36540	43647	CytochrOme B
43084	ATTTTTTTT A	ATTTTTTTT TA	56.1623	ins	mitochondrio n MT	S000007270	Q0105	COB	36540	43647	CytochrOme B
43084	ATTTTTTTT A	ATTTTTTTT TA	56.157	ins	mitochondrio n MT	S000007270	Q0105	COB	36540	43647	CytochrOme B
43354	T	A	54.8661	snp	mitochondrio n MT	S000007270	Q0105	COB	36540	43647	CytochrOme B
43652	T	G	56.1101	snp	mitochondrio n MT	intergenic					
44452	CTT	CT	54.1002	del	mitochondrio n MT	intergenic					
44452	C	A	54.1002	snp	mitochondrio n MT	intergenic					
45690	C	T	55.5807	snp	mitochondrio n MT	intergenic					
46779	A	G	55.9635	snp	mitochondrio n MT	S000007274	Q0130	OLI1	46723	46953	OLIgomycin resistance
51309	C	A	54.1002	snp	mitochondrio n MT	S000007277	Q0143		51277	51429	
53872	T	A	56.1036	snp	mitochondrio n MT	intergenic					
55293	C	A	56.2849	snp	mitochondrio n MT	intergenic		ORI3			Mitochondrial origin of replication
57545	T	C	56.2849	snp	mitochondrio n MT	intergenic		ORI4,21S_RRNA			Mitochondrial origin of replication, Mitochondrial 21S rRNA, intron encodes the I-SceI DNA endonuclease
58679	G	T	54.8661	snp	mitochondrio n MT	intergenic					
58937	G	T	54.8661	snp	mitochondrio n MT	intergenic					
59013	T	C	56.1623	snp	mitochondrio n MT	intergenic					
59028	T	C	56.204	snp	mitochondrio n MT	intergenic					
59311	C	A	56.1623	snp	mitochondrio n MT	intergenic					

59891	A	G	55.1533	snp	mitochondrion MT	intergenic					
59957	G	T	54.8661	snp	mitochondrion MT	intergenic					
60648	A	T	55.1533	snp	mitochondrion MT	intergenic					
60690	G	A	55.3887	snp	mitochondrion MT	intergenic					
61437	C	A	56.1101	snp	mitochondrion MT	S000007279	Q0160	SCEI	61022	61729	
61437	C	A	56.1101	snp	mitochondrion MT	S000007279	Q0160	SCEI	61022	61729	
61437	C	A	56.1101	snp	mitochondrion MT	S000007279	Q0160	SCEI	61022	61729	
61437	C	A	56.1101	snp	mitochondrion MT	S000007279	Q0160	SCEI	61022	61729	
61437	C	A	56.1101	snp	mitochondrion MT	S000007279	Q0160	SCEI	61022	61729	
61437	C	A	56.1101	snp	mitochondrion MT	S000007279	Q0160	SCEI	61022	61729	
61437	C	A	56.1101	snp	mitochondrion MT	S000007279	Q0160	SCEI	61022	61729	
61437	C	A	56.1101	snp	mitochondrion MT	S000007279	Q0160	SCEI	61022	61729	
61437	C	A	56.1101	snp	mitochondrion MT	S000007279	Q0160	SCEI	61022	61729	
62020	A	G	55.7364	snp	mitochondrion MT	intergenic					
62023	A	G	54.5182	snp	mitochondrion MT	intergenic					
63560	A	T	55.7364	snp	mitochondrion MT	intergenic					
64383	T	C	55.1533	snp	mitochondrion MT	intergenic					
65697	C	A	55.9635	snp	mitochondrion MT	intergenic					
65751	T	C	55.3887	snp	mitochondrion MT	intergenic					
65917	CGGGGGGGGGC	CGGGGGGGGC	55.9635	del	mitochondrion MT	S000007280	Q0182		65770	66174	

65917	CGGGGGGGGGC	CGGGGGGGGC	55.9635	del	mitochondrion MT	S000007280	Q0182		65770	66174	
67981	C	A	54.8661	snp	mitochondrion MT	intergenic					
69907	T	A	55.3887	snp	mitochondrion MT	intergenic					
71279	A	T	54.8661	snp	mitochondrion MT	intergenic					
73114	AGGGGGGC	AGGGGGC	55.7364	del	mitochondrion MT	intergenic					
73262	A	T	54.5182	snp	mitochondrion MT	intergenic					
73891	TT	TTCTGTCT	55.3887	ins	mitochondrion MT	S000007281	Q0250	COX2	73758	74513	Cytochrome c OXidase
74080	A	T	56.2849	snp	mitochondrion MT	S000007281	Q0250	COX2	73758	74513	Cytochrome c OXidase
74122	A	T	55.0097	snp	mitochondrion MT	S000007281	Q0250	COX2	73758	74513	Cytochrome c OXidase
74148	T	C	55.0097	snp	mitochondrion MT	S000007281	Q0250	COX2	73758	74513	Cytochrome c OXidase
74173	A	T	55.9635	snp	mitochondrion MT	S000007281	Q0250	COX2	73758	74513	Cytochrome c OXidase
74251	C	A	55.4448	snp	mitochondrion MT	S000007281	Q0250	COX2	73758	74513	Cytochrome c OXidase
74474	A	G	54.5182	snp	mitochondrion MT	S000007281	Q0250	COX2	73758	74513	Cytochrome c OXidase
74956	G	A	56.0042	snp	mitochondrion MT	S000007282	Q0255		74495	75984	
75316	T	C	55.5807	snp	mitochondrion MT	S000007282	Q0255		74495	75984	
75860	CTT	CT	54.8661	del	mitochondrion MT	S000007282	Q0255		74495	75984	
76744	AA	TT	55.7364	mnp	mitochondrion MT	intergenic					
78532	A	G	54.3092	snp	mitochondrion MT	intergenic					
79532	G	A	55.7364	snp	mitochondrion MT	S000007283	Q0275	COX3	79213	80022	Cytochrome c OXidase
79577	C	A	55.5807	snp	mitochondrion MT	S000007283	Q0275	COX3	79213	80022	Cytochrome c OXidase

79595	G	T	54.8661	snp	mitochondrion MT	S000007283	Q0275	COX3	79213	80022	Cytochrome c OXidase
79674	T	C	55.1533	snp	mitochondrion MT	S000007283	Q0275	COX3	79213	80022	Cytochrome c OXidase
79788	T	A	56.204	snp	mitochondrion MT	S000007283	Q0275	COX3	79213	80022	Cytochrome c OXidase
80688	T	C	55.7364	snp	mitochondrion MT	intergenic					
82969	TATA	TCC	54.1002	complex	mitochondrion MT	intergenic					
82987	G	T	54.8661	snp	mitochondrion MT	intergenic					
83380	CGGGGGGA	CGGGGGGGGA	54.5182	ins	mitochondrion MT	intergenic					
83951	GAG	GG	56.1101	del	mitochondrion MT	intergenic					
85596	GCCCCCCCG	GCCCCCCCCG	56.204	ins	mitochondrion MT	S000007284	Q0297		85554	85709	
85596	GCCCCCCCG	GCCCCCCCCG	56.204	ins	mitochondrion MT	S000007284	Q0297		85554	85709	
85596	GCCCCCCCG	GCCCCCCCCG	56.1831	ins	mitochondrion MT	S000007284	Q0297		85554	85709	

Table S2 GO-terms of the mayor cellular functions found in the whole genome sequence analysis.

**TTY23**								
**GO ID**	**TERM**	**NUM LIST ANNOTATIONS**	**LIST SIZE**	**CLUSTER FREQUENCY**	**TOTAL NUM ANNOTATIONS**	**POPULATION SIZE**	**GENOME FREQUENCY**	**ANNOTATED GENES**
GO:0006366	transcription by RNA polymerase II	36	244	14.75%	486	6486	7.49%	YBR112C, YBR198C, YBR289W, YCR093W, YDR009W, YDR224C, YDR311W, YDR394W, YDR421W, YER177W, YFL024C, YFR037C, YGL207W, YGL220W, YGR116W, YGR186W, YHR099W, YIL038C, YIL122W, YJL176C, YJR119C, YJR140C, YKR062W, YLR240W, YLR403W, YLR430W, YLR451W, YMR075W, YMR280C, YNL097C, YNL309W, YOL004W, YOR151C, YOR162C, YOR174W, YOR230W

GO:0042221	response to chemical	27	244	11.07%	440	6486	6.78%	YBR006W, YBR289W, YCL043C, YCR093W, YDR009W, YDR028C, YDR420W, YER118C, YFL053W, YGL220W, YGR088W, YGR116W, YHL035C, YHR030C, YIL046W, YIL122W, YJL095W, YJL176C, YJR066W, YLR096W, YLR299W, YLR378C, YLR452C, YMR162C, YMR280C, YOR162C, YPL256C
GO:0000278	mitotic cell cycle	26	244	10.66%	332	6486	5.12%	YBL105C, YBR275C, YDL056W, YDR054C, YDR419W, YDR420W, YDR457W, YER016W, YER177W, YGL086W, YGR116W, YHR115C, YIL026C, YIL046W, YJR092W, YJR140C, YLR086W, YNL172W, YNL262W, YNL309W, YOL004W, YOR129C, YPL242C, YPL253C, YPL256C, YPR135W
GO:0006325	chromatin organization	22	244	9.02%	251	6486	3.87%	YBR112C, YBR175W, YBR198C, YBR275C, YBR289W, YDR028C, YDR224C, YEL044W, YFR037C, YGL207W, YGR116W, YJL176C, YJR119C, YJR140C, YLR086W, YML065W, YMR075W, YMR080C, YMR127C, YNL097C, YOL004W, YOR230W
GO:0006629	lipid metabolic process	18	244	7.38%	297	6486	4.58%	YCR017C, YCR034W, YDL193W, YER093C, YGR060W, YGR175C, YHR067W, YJR066W, YKL182W, YLL031C, YLR240W, YLR242C, YLR305C, YMR101C, YMR272C, YOR022C, YPL069C, YPR006C
GO:0051726	regulation of cell cycle	17	244	6.97%	255	6486	3.93%	YBR175W, YBR275C, YCR093W, YDR499W, YER016W, YER177W, YGL086W, YGL207W, YHR014W, YHR030C, YHR115C, YJR066W, YMR092C, YNL172W, YNL262W, YPL242C, YPL256C
GO:0006281	DNA repair	16	244	6.56%	264	6486	4.07%	YBR275C, YBR289W, YDR224C, YDR288W, YDR311W, YDR419W, YFL024C, YFR037C, YHR099W, YIR002C, YJL164C, YLR430W, YNL262W, YOL004W, YPL164C, YPR135W
GO:0033043	regulation of organelle organization	15	244	6.15%	237	6486	3.65%	YBL105C, YCR093W, YDR490C, YER016W, YGL086W, YGL207W, YHR014W, YHR115C, YJL164C, YJR119C, YMR092C, YNL172W, YOR001W, YOR124C, YPR181C
GO:0048285	organelle fission	15	244	6.15%	243	6486	3.75%	YDR150W, YDR419W, YER016W, YER177W, YGL086W, YGL251C, YHR014W, YHR115C, YIL026C, YLR086W, YNL172W, YNL262W, YPL164C, YPL253C, YPR135W
GO:0051321	meiotic cell cycle	13	244	5.33%	291	6486	4.49%	YBR175W, YER096W, YER177W, YGL251C, YHR014W, YJL038C, YJR066W, YLR086W, YML052W, YOL004W, YOL048C, YOR230W, YPL164C

GO:0018193	peptidyl-amino acid modification	13	244	5.33%	148	6486	2.28%	YBR175W, YBR198C, YDR490C, YFL024C, YGR116W, YHR099W, YJL164C, YJR066W, YJR140C, YKL018W, YLR239C, YMR127C, YOR196C
GO:0007059	chromosome segregation	13	244	5.33%	194	6486	2.99%	YDR419W, YER016W, YGL086W, YGL207W, YGL251C, YHR014W, YIL026C, YIL144W, YLR086W, YNL172W, YNL262W, YPL253C, YPR135W
GO:0051603	proteolysis involved in cellular protein catabolic process	12	244	4.92%	224	6486	3.45%	YCL043C, YDR054C, YDR394W, YDR457W, YHR115C, YIL046W, YKL010C, YLR378C, YNL172W, YNR075W, YOR124C, YOR197W
GO:0006310	DNA recombination	12	244	4.92%	190	6486	2.93%	Q0050, Q0055, Q0065, Q0070, YBR289W, YDR206W, YGL251C, YIR002C, YMR080C, YNL262W, YPL164C, YPR135W
GO:0016570	histone modification	12	244	4.92%	110	6486	1.70%	YBR175W, YBR198C, YFL024C, YGR116W, YHR099W, YIL112W, YJR119C, YJR140C, YKL018W, YMR075W, YMR127C, YOL004W
GO:0006605	protein targeting	11	244	4.51%	242	6486	3.73%	YBR283C, YDR080W, YDR329C, YDR495C, YEL013W, YLR240W, YLR378C, YNL097C, YNL297C, YNR006W, YOL044W
GO:0071554	cell wall organization or biogenesis	11	244	4.51%	198	6486	3.05%	YBL105C, YCR017C, YDR420W, YDR490C, YER093C, YER177W, YHR030C, YJL038C, YJL095W, YJR066W, YOL048C
GO:0006417	regulation of translation	10	244	4.10%	207	6486	3.19%	YAL035W, YBL105C, YCR093W, YDR206W, YDR211W, YDR490C, YIL038C, YMR080C, YOL045W, YOR001W
GO:0070647	protein modification by small protein conjugation or removal	10	244	4.10%	164	6486	2.53%	YDR054C, YDR457W, YER177W, YHR115C, YIL038C, YIL046W, YKL010C, YMR080C, YNL172W, YOR124C
GO:0006520	cellular amino acid metabolic process	10	244	4.10%	155	6486	2.39%	YBR006W, YCL030C, YDR019C, YDR421W, YHR018C, YIL116W, YJL088W, YKL106W, YLR451W, YPL111W
GO:0006260	DNA replication	10	244	4.10%	139	6486	2.14%	YBR275C, YGL207W, YGL251C, YIR002C, YLR430W, YML065W, YMR075W, YNL262W, YOL004W, YPR135W
GO:0055085	transmembrane transport	10	244	4.10%	271	6486	4.18%	YBR069C, YBR296C, YEL065W, YGR125W, YHL035C, YLL028W, YLL043W, YLR378C, YNL318C, YOL044W

GO:0006811	ion transport	10	244	4.10%	210	6486	3.24%	YBR069C, YBR296C, YEL065W, YGR257C, YLL028W, YLL043W, YNL083W, YNR006W, YOR348C, YPL147W
GO:0006397	mRNA processing	10	244	4.10%	180	6486	2.78%	Q0050, Q0115, Q0120, YGR075C, YGR116W, YKL018W, YLR430W, YMR240C, YNL317W, YOR350C
GO:0006401	RNA catabolic process	9	244	3.69%	152	6486	2.34%	YBL105C, YCL011C, YCR093W, YDR206W, YDR490C, YIL038C, YJR119C, YMR080C, YOR001W
GO:0007005	mitochondrion organization	9	244	3.69%	282	6486	4.35%	YDR150W, YDR231C, YDR322W, YDR332W, YLL007C, YLR077W, YLR305C, YLR356W, YOR124C
GO:0007010	cytoskeleton organization	9	244	3.69%	250	6486	3.85%	YBL105C, YDR150W, YER016W, YER093C, YHR115C, YJR092W, YMR092C, YPL242C, YPL253C
GO:0006468	protein phosphorylation	8	244	3.28%	127	6486	1.96%	YDR311W, YDR490C, YHR030C, YJL164C, YJR066W, YOL016C, YOL045W, YPL256C
GO:0008380	RNA splicing	8	244	3.28%	140	6486	2.16%	Q0065, Q0110, Q0115, Q0120, YGR075C, YMR240C, YOR350C, YPL083C
GO:0006354	DNA-templated transcription, elongation	8	244	3.28%	105	6486	1.62%	YCR093W, YFR037C, YGR116W, YGR186W, YIL038C, YJR140C, YLR240W, YMR075W
GO:0000910	cytokinesis	8	244	3.28%	96	6486	1.48%	YBL105C, YDR420W, YHR030C, YHR115C, YJR092W, YMR092C, YOR129C, YPL242C
GO:0006364	rRNA processing	8	244	3.28%	327	6486	5.04%	YAL035W, YDR457W, YGL076C, YLR106C, YLR196W, YLR430W, YOR001W, YPL217C
GO:0005975	carbohydrate metabolic process	8	244	3.28%	148	6486	2.28%	YDL189W, YDR009W, YDR109C, YDR420W, YER177W, YFL053W, YOL045W, YPR184W
GO:0031399	regulation of protein modification process	7	244	2.87%	118	6486	1.82%	YDR490C, YER177W, YGR116W, YGR186W, YJR066W, YJR140C, YPL256C
GO:0032200	telomere organization	7	244	2.87%	134	6486	2.07%	YBR175W, YBR275C, YCL011C, YDR499W, YKL018W, YMR127C, YOR001W
GO:0006974	cellular response to DNA damage stimulus	7	244	2.87%	78	6486	1.20%	YBR112C, YDR499W, YER177W, YJR066W, YLR430W, YNL085W, YNL262W
GO:0015931	nucleobase-containing compound transport	6	244	2.46%	139	6486	2.14%	YCL011C, YGR116W, YKL205W, YNL083W, YNR006W, YPL147W

GO:0051049	regulation of transport	6	244	2.46%	96	6486	1.48%	YHR030C, YLR096W, YLR106C, YLR240W, YNL097C, YPR181C
GO:0032787	monocarboxylic acid metabolic process	6	244	2.46%	127	6486	1.96%	YBR006W, YCR034W, YHR067W, YKL182W, YMR272C, YPR006C
GO:0016050	vesicle organization	6	244	2.46%	88	6486	1.36%	YDR080W, YDR498C, YEL013W, YLR305C, YPL085W, YPR181C
GO:0006897	endocytosis	6	244	2.46%	102	6486	1.57%	YBR214W, YDR490C, YLR242C, YML052W, YNL297C, YNR075W
GO:0051052	regulation of DNA metabolic process	6	244	2.46%	119	6486	1.83%	YBR275C, YIR002C, YKL010C, YMR075W, YOL004W, YOR001W
GO:0006352	DNA-templated transcription, initiation	6	244	2.46%	75	6486	1.16%	YBR198C, YDR394W, YGL207W, YGR116W, YGR186W, YNL151C
GO:0070925	organelle assembly	6	244	2.46%	112	6486	1.73%	YBL105C, YCR093W, YDR490C, YEL013W, YHR115C, YJL164C
GO:0043543	protein acylation	6	244	2.46%	70	6486	1.08%	YBR198C, YFL024C, YHR099W, YJR140C, YMR127C, YOR034C
GO:0006997	nucleus organization	5	244	2.05%	96	6486	1.48%	YDR457W, YJR119C, YLR086W, YNL097C, YOL004W
GO:0043934	sporulation	5	244	2.05%	131	6486	2.02%	YER096W, YER177W, YJL038C, YML052W, YOL048C
GO:0006873	cellular ion homeostasis	5	244	2.05%	132	6486	2.04%	YDR495C, YEL065W, YGR257C, YPL135W, YPL224C
GO:0048193	Golgi vesicle transport	5	244	2.05%	192	6486	2.96%	YDR498C, YFL048C, YLR309C, YPL085W, YPR181C
GO:0016197	endosomal transport	5	244	2.05%	86	6486	1.33%	YCR034W, YDR490C, YJL154C, YNL297C, YNR075W
GO:0048284	organelle fusion	5	244	2.05%	99	6486	1.53%	YDR080W, YDR498C, YEL013W, YLR305C, YOR124C
GO:0051604	protein maturation	5	244	2.05%	86	6486	1.33%	YDR080W, YDR231C, YLR239C, YNL238W, YOR196C
GO:0006979	response to oxidative stress	5	244	2.05%	103	6486	1.59%	YBR006W, YDR231C, YGR088W, YJR066W, YOR162C
GO:0006869	lipid transport	4	244	1.64%	65	6486	1.00%	YKL187C, YLR242C, YMR162C, YPL147W
GO:0006360	transcription by RNA polymerase I	4	244	1.64%	70	6486	1.08%	YDR311W, YJR066W, YNL097C, YOL004W
GO:0008033	tRNA processing	4	244	1.64%	118	6486	1.82%	YLR430W, YMR259C, YPL083C, YPL135W
GO:0048308	organelle inheritance	4	244	1.64%	55	6486	0.85%	YDR150W, YDR329C, YDR495C, YEL013W

GO:0006497	protein lipidation	4	244	1.64%	45	6486	0.69%	YCR017C, YLL031C, YLR242C, YOR034C
GO:0007124	pseudohyphal growth	4	244	1.64%	76	6486	1.17%	YCR093W, YER177W, YMR016C, YMR162C
GO:0009451	RNA modification	4	244	1.64%	177	6486	2.73%	YJR066W, YMR259C, YNL132W, YPL135W
GO:0009408	response to heat	4	244	1.64%	53	6486	0.82%	YBR025C, YJR066W, YNL097C, YOL004W
GO:0002181	cytoplasmic translation	4	244	1.64%	197	6486	3.04%	YAL035W, YGL076C, YOL045W, YOR182C
GO:0042274	ribosomal small subunit biogenesis	4	244	1.64%	137	6486	2.11%	YAL035W, YDR457W, YNL132W, YPL217C
GO:0045333	cellular respiration	4	244	1.64%	89	6486	1.37%	Q0045, Q0105, Q0250, Q0275
GO:0008213	protein alkylation	3	244	1.23%	52	6486	0.80%	YBR175W, YGR116W, YKL018W
GO:0006865	amino acid transport	3	244	1.23%	46	6486	0.71%	YBR069C, YGR125W, YOR348C
GO:0055086	nucleobase-containing small molecule metabolic process	3	244	1.23%	163	6486	2.51%	YBL099W, YDR298C, YOR128C
GO:0007033	vacuole organization	3	244	1.23%	101	6486	1.56%	YDR080W, YDR495C, YEL013W
GO:0043144	sno(s)RNA processing	3	244	1.23%	45	6486	0.69%	YKL018W, YLR430W, YOR001W
GO:0042273	ribosomal large subunit biogenesis	3	244	1.23%	118	6486	1.82%	YDR457W, YGL076C, YLR106C
GO:0006091	generation of precursor metabolites and energy	3	244	1.23%	80	6486	1.23%	YER177W, YOL045W, YPR184W
GO:0006413	translational initiation	3	244	1.23%	61	6486	0.94%	YAL035W, YDR211W, YJR066W
GO:0061025	membrane fusion	3	244	1.23%	51	6486	0.79%	YDR080W, YDR498C, YLR305C
GO:0006470	protein dephosphorylatio n	2	244	0.82%	30	6486	0.46%	YGR186W, YHR076W

GO:0006353	DNA-templated transcription, termination	2	244	0.82%	41	6486	0.63%	YGR116W, YLR430W
GO:0032543	mitochondrial translation	2	244	0.82%	167	6486	2.57%	Q0140, YNR037C
GO:0006486	protein glycosylation	2	244	0.82%	64	6486	0.99%	YDL193W, YMR101C
GO:0008643	carbohydrate transport	2	244	0.82%	36	6486	0.56%	YLL043W, YNL318C
GO:0042255	ribosome assembly	2	244	0.82%	64	6486	0.99%	YAL035W, YLR106C
GO:0006970	response to osmotic stress	2	244	0.82%	68	6486	1.05%	YDR420W, YER118C
GO:0001403	invasive growth in response to glucose limitation	2	244	0.82%	54	6486	0.83%	YBR289W, YNL097C
GO:0006383	transcription by RNA polymerase III	2	244	0.82%	45	6486	0.69%	YNL151C, YPR190C
GO:0007031	peroxisome organization	2	244	0.82%	50	6486	0.77%	YDR329C, YOL044W
GO:0007114	cell budding	1	244	0.41%	56	6486	0.86%	YJR092W
GO:0006457	protein folding	1	244	0.41%	98	6486	1.51%	YCL043C
GO:0006418	tRNA aminoacylation for protein translation	1	244	0.41%	36	6486	0.56%	YGR264C
GO:0000054	ribosomal subunit export from nucleus	1	244	0.41%	52	6486	0.80%	YLR106C
GO:0042594	response to starvation	1	244	0.41%	62	6486	0.96%	YDR028C
GO:0006887	exocytosis	1	244	0.41%	47	6486	0.72%	YLR096W
**AT22**								
**GO ID**	**TERM**	**NUM LIST ANNOTATIONS**	**LIST SIZE**	**CLUSTER FREQUENCY**	**TOTAL NUM ANNOTATIONS**	**POPULATION SIZE**	**GENOME FREQUENCY**	**ANNOTATED GENES**

GO:0006366	transcription by RNA polymerase II	15	194	7.73%	486	6485	7.49%	YDL214C, YDR081C, YER088C, YGR056W, YIL153W, YJR035W, YKL015W, YKR064W, YKR082W, YKR099W, YLR277C, YMR280C, YOR117W, YOR151C, YPL190C
GO:0006366	chromosome segregation	14	194	7.22%	194	6485	2.99%	YCL061C, YDR180W, YDR409W, YGL086W, YIL031W, YIL144W, YIR025W, YKL193C, YKR054C, YLR234W, YMR076C, YOL034W, YOR014W, YOR178C
GO:0000278	mitotic cell cycle	14	194	7.22%	333	6485	5.13%	YCL061C, YDL089W, YDR180W, YGL086W, YIL031W, YIL153W, YIR025W, YKL101W, YKR054C, YLR234W, YMR055C, YMR076C, YOR014W, YOR178C
GO:0051726	regulation of cell cycle	13	194	6.70%	255	6485	3.93%	YCL061C, YDL089W, YDR293C, YDR523C, YGL086W, YGL134W, YIL031W, YIR025W, YKL101W, YMR055C, YOR014W, YOR178C, YPL138C
GO:0048285	organelle fission	13	194	6.70%	243	6485	3.75%	YCL061C, YDL089W, YDR180W, YGL086W, YIL031W, YIR025W, YKR054C, YLR234W, YML128C, YMR055C, YMR076C, YOR014W, YOR178C
GO:0006281	DNA repair	13	194	6.70%	265	6485	4.09%	YCL061C, YDR180W, YEL018W, YGR056W, YHR120W, YIL153W, YJR035W, YKR082W, YLR442C, YMR076C, YMR137C, YOL034W, YOR014W
GO:0042221	response to chemical	11	194	5.67%	454	6485	7.00%	YAL026C, YBL055C, YCR106W, YER042W, YGR088W, YHR005C, YJR138W, YKL068W, YKR082W, YLR452C, YMR280C
GO:0006325	chromatin organization	11	194	5.67%	251	6485	3.87%	YCL061C, YDR180W, YER088C, YGR056W, YKR082W, YLR373C, YLR442C, YMR076C, YOR274W, YPL138C, YPL181W
GO:0006310	DNA recombination	10	194	5.15%	190	6485	2.93%	Q0050, Q0055, Q0065, Q0070, Q0160, YDR180W, YIR025W, YLR234W, YML128C, YOL034W
GO:0006417	regulation of translation	10	194	5.15%	206	6485	3.18%	YDL069C, YDR293C, YER025W, YER176W, YGL122C, YGL195W, YGR195W, YGR271W, YKL204W, YPL203W
GO:0015931	nucleobase-containing compound transport	10	194	5.15%	139	6485	2.14%	YGL122C, YIL115C, YIL149C, YJR042W, YJR123W, YKL068W, YKR082W, YMR275C, YOR112W, YOR160W
GO:0007005	mitochondrion organization	10	194	5.15%	282	6485	4.35%	YBL017C, YDL069C, YHL021C, YHR120W, YIL157C, YLR091W, YLR422W, YMR275C, YNL169C, YPR020W
GO:0006397	mRNA processing	10	194	5.15%	180	6485	2.78%	Q0050, Q0115, Q0120, YDR240C, YGL122C, YGR093W, YLR147C, YLR277C, YOR148C, YPL190C

GO:0071554	cell wall organization or biogenesis	9	194	4.64%	197	6485	3.04%	YDR293C, YDR523C, YGR032W, YHR102W, YHR142W, YJR013W, YKL104C, YMR237W, YPL140C
GO:0008380	RNA splicing	9	194	4.64%	140	6485	2.16%	Q0065, Q0110, Q0115, Q0120, YDR240C, YGR093W, YLR147C, YLR277C, YOR148C
GO:0048193	Golgi vesicle transport	9	194	4.64%	192	6485	2.96%	YAL026C, YAL042W, YDR166C, YGL233W, YHR142W, YKL135C, YKR014C, YLR078C, YMR237W
GO:0006997	nucleus organization	9	194	4.64%	97	6485	1.50%	YDR180W, YER088C, YHR005C, YIL040W, YIL115C, YJR042W, YKR082W, YML031W, YPL181W
GO:0051321	meiotic cell cycle	9	194	4.64%	291	6485	4.49%	YDR523C, YGR032W, YIR025W, YLR234W, YML128C, YMR076C, YOR014W, YOR178C, YPL138C
GO:0006811	ion transport	8	194	4.12%	215	6485	3.32%	YDL128W, YIL023C, YML123C, YMR275C, YNR002C, YNR013C, YOR316C, YPR156C
GO:0006629	lipid metabolic process	8	194	4.12%	297	6485	4.58%	YDR331W, YER044C, YGR007W, YGR175C, YJR013W, YML008C, YNL169C, YPL120W
GO:0006897	endocytosis	8	194	4.12%	102	6485	1.57%	YAL026C, YAR042W, YDL161W, YHR161C, YKL203C, YKR014C, YMR275C, YNL297C
GO:0033043	regulation of organelle organization	8	194	4.12%	237	6485	3.65%	YGL086W, YIL031W, YIR025W, YJR138W, YNL169C, YOR014W, YOR178C, YPL203W
GO:0007010	cytoskeleton organization	8	194	4.12%	254	6485	3.92%	YDL161W, YDR523C, YIL149C, YIL153W, YKL203C, YKR054C, YML031W, YOR014W
GO:0006364	rRNA processing	8	194	4.12%	328	6485	5.06%	YGR195W, YHR021C, YJR123W, YMR229C, YOL022C, YOR063W, YOR287C, YPR143W
GO:0006605	protein targeting	7	194	3.61%	242	6485	3.73%	YAL002W, YBL017C, YDR265W, YKR014C, YNL297C, YOR007C, YPL120W
GO:0016197	endosomal transport	7	194	3.61%	86	6485	1.33%	YAL002W, YAL026C, YDR486C, YJL207C, YKR014C, YNL297C, YPL120W
GO:0055085	transmembrane transport	7	194	3.61%	271	6485	4.18%	YDL128W, YDR265W, YJL214W, YML123C, YNR002C, YNR013C, YOR316C
GO:0045333	cellular respiration	7	194	3.61%	89	6485	1.37%	Q0045, Q0105, Q0250, Q0275, YJL209W, YNL169C, YOR355W
GO:0032543	mitochondrial translation	6	194	3.09%	167	6485	2.58%	YDL069C, YDR337W, YGR165W, YHR011W, YHR147C, YKL138C
GO:0006401	RNA catabolic process	6	194	3.09%	152	6485	2.34%	YGL122C, YGR195W, YJL209W, YKL204W, YLR442C, YPL190C
GO:0032200	telomere organization	6	194	3.09%	134	6485	2.07%	YCL061C, YER088C, YKR082W, YLR234W, YLR442C, YPL138C

GO:0070647	protein modification by small protein conjugation or removal	6	194	3.09%	164	6485	2.53%	YDR265W, YDR409W, YGL211W, YIL031W, YIR025W, YMR275C
GO:0002181	cytoplasmic translation	5	194	2.58%	197	6485	3.04%	YER025W, YGR271W, YHR021C, YML073C, YPL203W
GO:0006520	cellular amino acid metabolic process	5	194	2.58%	155	6485	2.39%	YEL038W, YER052C, YKL015W, YMR108W, YNL277W
GO:0006468	protein phosphorylation	5	194	2.58%	126	6485	1.94%	YDL079C, YDR523C, YGL134W, YKL203C, YPL140C
GO:0005975	carbohydrate metabolic process	5	194	2.58%	148	6485	2.28%	YDR081C, YGL134W, YGR032W, YIL097W, YOR178C
GO:0042274	ribosomal small subunit biogenesis	5	194	2.58%	137	6485	2.11%	YHR021C, YJR123W, YMR229C, YOL022C, YOR287C
GO:0070925	organelle assembly	5	194	2.58%	112	6485	1.73%	YJR138W, YKR014C, YNL169C, YOR014W, YPL203W
GO:0042273	ribosomal large subunit biogenesis	4	194	2.06%	119	6485	1.84%	YML073C, YMR229C, YOR063W, YPR143W
GO:0008033	tRNA processing	4	194	2.06%	118	6485	1.82%	YGL211W, YLR091W, YOR274W, YPL190C
GO:0006091	generation of precursor metabolites and energy	4	194	2.06%	80	6485	1.23%	YDR081C, YGL134W, YOR178C, YPR074C
GO:0006979	response to oxidative stress	4	194	2.06%	106	6485	1.63%	YBL055C, YER042W, YGR088W, YJR138W
GO:0051052	regulation of DNA metabolic process	4	194	2.06%	119	6485	1.84%	YCL061C, YLR234W, YLR442C, YPL181W
GO:0042255	ribosome assembly	4	194	2.06%	64	6485	0.99%	YHR021C, YML073C, YMR229C, YOR063W
GO:0009408	response to heat	4	194	2.06%	53	6485	0.82%	YKL068W, YKR082W, YOR007C, YOR178C
GO:0018193	peptidyl-amino acid modification	4	194	2.06%	148	6485	2.28%	YDR409W, YIL031W, YOR355W, YPL138C
GO:0006260	DNA replication	4	194	2.06%	139	6485	2.14%	YBR276C, YCL061C, YLR442C, YPL181W
GO:0051049	regulation of transport	4	194	2.06%	96	6485	1.48%	YHR005C, YKL068W, YKL203C, YNR013C
GO:0007033	vacuole organization	3	194	1.55%	101	6485	1.56%	YCL063W, YJR138W, YOR014W

GO:0048308	organelle inheritance	3	194	1.55%	55	6485	0.85%	YCL063W, YDR166C, YMR275C
GO:0006869	lipid transport	3	194	1.55%	71	6485	1.09%	YAL026C, YAR042W, YHR080C
GO:0051603	proteolysis involved in protein catabolic process	3	194	1.55%	224	6485	3.45%	YIL097W, YIR025W, YOR117W
GO:0006457	protein folding	3	194	1.55%	98	6485	1.51%	YHR142W, YML048W, YML130C
GO:0006887	exocytosis	3	194	1.55%	47	6485	0.72%	YAR042W, YDR166C, YGL233W
GO:0051604	protein maturation	3	194	1.55%	86	6485	1.33%	YBL091C, YNL169C, YOR219C
GO:0048284	organelle fusion	3	194	1.55%	99	6485	1.53%	YHR005C, YLR078C, YNL169C
GO:0043144	sno(s)RNA processing	3	194	1.55%	45	6485	0.69%	YGR195W, YLR277C, YPL190C
GO:0006418	tRNA aminoacylation for protein translation	3	194	1.55%	36	6485	0.56%	YDR037W, YHR011W, YLL018C
GO:0031399	regulation of protein modification process	3	194	1.55%	118	6485	1.82%	YDR409W, YGL134W, YIR025W
GO:0009451	RNA modification	3	194	1.55%	177	6485	2.73%	YGL211W, YKL203C, YOR274W
GO:0016050	vesicle organization	2	194	1.03%	88	6485	1.36%	YKR014C, YLR078C
GO:0000054	ribosomal subunit export from nucleus	2	194	1.03%	52	6485	0.80%	YIL115C, YJR042W
GO:0016570	histone modification	2	194	1.03%	110	6485	1.70%	YOR355W, YPL138C
GO:0006497	protein lipidation	2	194	1.03%	45	6485	0.69%	YDR331W, YJR013W
GO:0055086	nucleobase-containing small molecule metabolic process	2	194	1.03%	163	6485	2.51%	YGR180C, YPR020W
GO:0006353	DNA-templated transcription, termination	2	194	1.03%	41	6485	0.63%	YLR277C, YPL190C
GO:0006486	protein glycosylation	2	194	1.03%	64	6485	0.99%	YEL036C, YOR320C

GO:0006414	translational elongation	2	194	1.03%	333	6485	5.13%	YGL195W, YLR249W
GO:0006873	cellular ion homeostasis	2	194	1.03%	131	6485	2.02%	YDL128W, YOR316C
GO:0043934	sporulation	2	194	1.03%	131	6485	2.02%	YDR523C, YGR032W
GO:0006974	cellular response to DNA damage stimulus	2	194	1.03%	78	6485	1.20%	YCL061C, YJR035W
GO:0008643	carbohydrate transport	2	194	1.03%	36	6485	0.56%	YGR289C, YJL214W
GO:0061025	membrane fusion	1	194	0.52%	51	6485	0.79%	YLR078C
GO:0009311	oligosaccharide metabolic process	1	194	0.52%	43	6485	0.66%	YGR289C
GO:0006865	amino acid transport	1	194	0.52%	46	6485	0.71%	YPL265W
GO:0007114	cell budding	1	194	0.52%	56	6485	0.86%	YHR102W
GO:0006354	DNA-templated transcription, elongation	1	194	0.52%	105	6485	1.62%	YGR056W
GO:0006352	DNA-templated transcription, initiation	1	194	0.52%	75	6485	1.16%	YOR117W
GO:0032787	monocarboxylic acid metabolic process	1	194	0.52%	127	6485	1.96%	YML054C
GO:0006383	transcription by RNA polymerase III	1	194	0.52%	45	6485	0.69%	YGL122C
GO:0008213	protein alkylation	1	194	0.52%	52	6485	0.80%	YPL138C
GO:0006413	translational initiation	1	194	0.52%	61	6485	0.94%	YER025W
GO:0000902	cell morphogenesis	1	194	0.52%	27	6485	0.42%	YJL201W
GO:0006766	vitamin metabolic process	1	194	0.52%	52	6485	0.80%	YDR081C
GO:0000910	cytokinesis	1	194	0.52%	96	6485	1.48%	YOR014W
GO:0043543	protein acylation	1	194	0.52%	70	6485	1.08%	YOR355W
GO:0006970	response to osmotic stress	1	194	0.52%	68	6485	1.05%	YIL153W
GO:0007031	peroxisome organization	1	194	0.52%	50	6485	0.77%	YDR265W

GO:0006360	transcription by RNA polymerase I	1	194	0.52%	70	6485	1.08%	YOR341W
GO:0001403	invasive growth in response to glucose limitation	1	194	0.52%	54	6485	0.83%	YPL203W

TAT12

**GO ID**	**TERM**	**NUM LIST ANNOTATIONS**	**LIST SIZE**	**CLUSTER FREQUENCY**	**TOTAL NUM ANNOTATIONS**	**POPULATION SIZE**	**GENOME FREQUENCY**	**ANNOTATED GENES**
GO:0042221	response to chemical	44	488	9.02%	454	6485	7.00%	YBL058W, YBL103C, YCL047C, YCR091W, YCR093W, YDL170W, YDR001C, YDR049W, YDR135C, YDR192C, YDR406W, YER020W, YER042W, YGL166W, YGL209W, YGR014W, YGR097W, YHR158C, YHR206W, YIL030C, YIR023W, YJL073W, YJL080C, YJL082W, YJL110C, YJR138W, YKL056C, YKL150W, YKL178C, YKR071C, YLL063C, YLR019W, YLR207W, YML013W, YMR116C, YMR176W, YMR216C, YNL080C, YNL231C, YNL241C, YOL081W, YOR018W, YOR270C, YOR328W
GO:0006366	transcription by RNA polymerase II	43	488	8.81%	486	6485	7.49%	YBL103C, YBR049C, YCR093W, YDL080C, YDL140C, YDL170W, YDR301W, YDR310C, YDR516C, YER169W, YER184C, YFL024C, YFL033C, YFR034C, YGL150C, YGL166W, YGL207W, YGL209W, YHR099W, YHR119W, YHR167W, YHR206W, YIL017C, YIL126W, YIL143C, YIR023W, YJL110C, YKL062W, YLR019W, YLR223C, YLR277C, YLR430W, YML076C, YMR033W, YNL118C, YNR052C, YOL072W, YOR151C, YOR172W, YOR290C, YPL082C, YPL086C, YPL254W
GO:0000278	mitotic cell cycle	37	488	7.58%	333	6485	5.13%	YAL047C, YBL023C, YBL051C, YBL063W, YBL105C, YCR038C, YDL056W, YDL132W, YDR184C, YDR247W, YDR457W, YER016W, YFL008W, YFL033C, YGL021W, YGL086W, YGR188C, YGR250C, YHR115C, YHR158C, YIL159W, YIR006C, YIR025W, YLL021W, YLR084C, YLR319C, YLR381W, YML124C, YMR032W, YMR190C, YNL262W, YNL271C, YOL070C, YOL072W, YOR058C, YOR301W, YPR135W

GO:0006281	DNA repair	34	488	6.97%	265	6485	4.09%	YBL023C, YBL088C, YDL140C, YDR191W, YDR291W, YDR334W, YER041W, YFL008W, YFL024C, YGL092W, YGL150C, YGL168W, YGL201C, YHR099W, YHR154W, YIL126W, YIL132C, YIL143C, YJL072C, YJR006W, YKL025C, YLR247C, YLR430W, YLR442C, YMR190C, YMR224C, YMR284W, YNL102W, YNL262W, YOL072W, YOR290C, YOR328W, YPR019W, YPR135W
GO:0006310	DNA recombination	33	488	6.76%	190	6485	2.93%	Q0050, Q0055, Q0065, Q0070, Q0160, YBL023C, YDR191W, YDR334W, YFL003C, YGL150C, YGL168W, YGL201C, YGL213C, YHR039C, YHR157W, YHR167W, YIL132C, YIL143C, YIR025W, YJL072C, YJL208C, YLL033W, YLR247C, YMR190C, YMR224C, YMR284W, YNL088W, YNL262W, YOR290C, YOR328W, YPR019W, YPR038W, YPR135W
GO:0007010	cytoskeleton organization	30	488	6.15%	254	6485	3.92%	YAL047C, YBL063W, YBL105C, YDL176W, YDR085C, YDR150W, YDR379W, YEL022W, YER016W, YGL206C, YHR115C, YHR158C, YIL095W, YIL118W, YIL126W, YIL149C, YIL159W, YIR006C, YKL007W, YKR055W, YLL021W, YLR319C, YMR032W, YNL020C, YNL138W, YNL271C, YOL078W, YOR058C, YPL115C, YPL255W
GO:0055085	transmembrane transport	29	488	5.94%	271	6485	4.18%	YBL075C, YBR043C, YBR127C, YBR132C, YBR147W, YBR295W, YCL040W, YCR098C, YDR046C, YDR135C, YDR345C, YFL054C, YGR097W, YGR121C, YGR125W, YIL022W, YJL059W, YJL094C, YJL198W, YJL210W, YKL197C, YKL217W, YNL318C, YOR087W, YOR137C, YOR254C, YOR328W, YPL094C, YPR024W
GO:0051321	meiotic cell cycle	28	488	5.74%	291	6485	4.49%	YBL058W, YBR043C, YCR086W, YDR506C, YDR516C, YER020W, YFL003C, YFL033C, YGL213C, YHL023C, YHR039C, YHR119W, YHR157W, YIL017C, YIL126W, YIL132C, YIR025W, YJL080C, YLL033W, YLL040C, YML124C, YMR139W, YMR190C, YMR224C, YMR306W, YNL088W, YNL102W, YNL231C
GO:0033043	regulation of organelle organization	28	488	5.74%	237	6485	3.65%	YBL023C, YBL105C, YCR093W, YDR189W, YDR291W, YER016W, YGL086W, YGL207W, YGR004W, YGR188C, YHL023C, YHR115C, YHR158C, YIL095W, YIL118W, YIL159W, YIR006C, YIR025W, YJR138W, YKL007W, YKR055W, YLL021W, YLR319C, YLR381W, YMR032W, YNL138W, YNL271C, YNL323W

GO:0048285	organelle fission	26	488	5.33%	243	6485	3.75%	YAL047C, YBL063W, YCR086W, YDR150W, YER016W, YFL003C, YFL008W, YGL086W, YGL213C, YGR188C, YHR039C, YHR115C, YHR157W, YHR158C, YIL132C, YIR025W, YJL080C, YLR381W, YML124C, YMR190C, YMR224C, YNL088W, YNL138W, YNL262W, YOR058C, YPR135W
GO:0006325	chromatin organization	23	488	4.71%	251	6485	3.87%	YCR086W, YDR181C, YDR191W, YDR310C, YDR334W, YFR034C, YGL092W, YGL150C, YGL207W, YHR119W, YIL126W, YJL080C, YKL062W, YLL022C, YLR085C, YLR223C, YLR442C, YML065W, YMR033W, YMR284W, YNL088W, YOR290C, YPL254W
GO:0051726	regulation of cell cycle	22	488	4.51%	255	6485	3.93%	YAL047C, YBL051C, YCR093W, YDR283C, YDR499W, YER016W, YFL033C, YGL021W, YGL086W, YGL207W, YGR188C, YGR250C, YHR115C, YHR119W, YHR154W, YHR158C, YIR025W, YLR381W, YMR032W, YMR190C, YNL231C, YNL262W
GO:0006629	lipid metabolic process	22	488	4.51%	297	6485	4.58%	YDR191W, YER026C, YGR143W, YHR072W, YJL062W, YJL091C, YJL100W, YJL167W, YJR110W, YKL008C, YKL150W, YKL182W, YKL212W, YKR089C, YLL031C, YLR450W, YMR015C, YMR207C, YMR296C, YNL087W, YNL231C, YPR128C
GO:0006811	ion transport	21	488	4.30%	215	6485	3.32%	YBR132C, YBR295W, YCR075C, YCR098C, YDR135C, YGL167C, YGR121C, YGR217W, YHL008C, YJL059W, YJL094C, YJL198W, YKL217W, YKL220C, YLL015W, YLR214W, YMR275C, YNL275W, YOR087W, YOR137C, YPR128C
GO:0006260	DNA replication	21	488	4.30%	139	6485	2.14%	YBL023C, YBR228W, YDR310C, YGL168W, YGL201C, YGL207W, YHR197W, YJL072C, YJR006W, YLR002C, YLR381W, YLR430W, YLR442C, YML065W, YMR190C, YNL088W, YNL102W, YNL262W, YOR290C, YPR019W, YPR135W
GO:0051603	proteolysis involved in protein catabolic process	21	488	4.30%	224	6485	3.45%	YBL058W, YBL075C, YCL047C, YDL132W, YDR049W, YDR330W, YDR457W, YER012W, YGL227W, YHR115C, YIL017C, YIL030C, YIL097W, YIR025W, YJL073W, YKL010C, YLR207W, YML013W, YMR314W, YOR256C, YPR024W

GO:0048193	Golgi vesicle transport	20	488	4.10%	192	6485	2.96%	YDL058W, YDR141C, YDR189W, YDR407C, YEL022W, YGL137W, YGL206C, YGL233W, YHR098C, YIL004C, YJL029C, YJL204C, YKR027W, YLL040C, YNL263C, YOR307C, YPL070W, YPL195W, YPR032W, YPR105C
GO:0007059	chromosome segregation	20	488	4.10%	194	6485	2.99%	YBL058W, YBL063W, YCR086W, YDR409W, YER016W, YFL008W, YGL086W, YGL207W, YGR188C, YIL126W, YIL132C, YIR025W, YJL080C, YLR381W, YML124C, YMR190C, YMR311C, YNL262W, YOR058C, YPR135W
GO:0006397	mRNA processing	18	488	3.69%	180	6485	2.78%	Q0050, Q0115, Q0120, YBR237W, YCR035C, YDR301W, YER172C, YGR278W, YKL074C, YKL173W, YKR086W, YLR277C, YLR430W, YMR213W, YMR216C, YMR288W, YNL245C, YOL072W
GO:0008380	RNA splicing	18	488	3.69%	140	6485	2.16%	Q0065, Q0110, Q0115, Q0120, YBR237W, YDR194C, YER172C, YGR278W, YKL074C, YKL173W, YKR086W, YLR277C, YMR087W, YMR213W, YMR216C, YMR223W, YMR288W, YNL245C
GO:0006605	protein targeting	17	488	3.48%	242	6485	3.73%	YAL002W, YBL075C, YDR495C, YER154W, YGR141W, YIL022W, YJL210W, YKL197C, YLL040C, YLR431C, YNL275W, YNR007C, YOR254C, YPL094C, YPL195W, YPR024W, YPR105C
GO:0006364	rRNA processing	17	488	3.48%	328	6485	5.06%	YAL035W, YCR035C, YDR398W, YDR457W, YHR197W, YJL136C, YKL014C, YLR002C, YLR197W, YLR430W, YLR435W, YMR049C, YMR143W, YNL061W, YOR205C, YPL082C, YPL217C
GO:0032200	telomere organization	17	488	3.48%	134	6485	2.07%	YBL088C, YDR181C, YDR191W, YDR291W, YDR499W, YGL092W, YGL150C, YHR119W, YHR167W, YJL080C, YLL022C, YLR223C, YLR442C, YMR190C, YMR224C, YMR284W, YNL088W
GO:0000910	cytokinesis	15	488	3.07%	96	6485	1.48%	YBL105C, YCR038C, YDR184C, YHR115C, YHR158C, YIL159W, YIR006C, YLL021W, YLR084C, YLR319C, YMR032W, YNL271C, YOL070C, YOL072W, YOR301W
GO:0005975	carbohydrate metabolic process	15	488	3.07%	148	6485	2.28%	YBL058W, YBR018C, YCL040W, YDR516C, YGL027C, YGL227W, YGR143W, YHL032C, YHR047C, YIL017C, YIL097W, YLR300W, YMR311C, YOL045W, YOL136C
GO:0006417	regulation of translation	15	488	3.07%	206	6485	3.18%	YAL035W, YBL105C, YBR271W, YCR035C, YCR093W, YDR283C, YGL195W, YKL025C, YML129C, YMR116C, YNL091W, YNL118C, YNR045W, YNR052C, YOL045W

GO:0051049	regulation of transport	15	488	3.07%	96	6485	1.48%	YBR132C, YDR189W, YGL092W, YGR097W, YHL008C, YHR158C, YIL095W, YIL118W, YJL039C, YJL165C, YJL198W, YKR021W, YMR216C, YNL020C, YOR018W
GO:0015931	nucleobase-containing compound transport	15	488	3.07%	139	6485	2.14%	YDR192C, YGL092W, YGR218W, YHR167W, YIL143C, YIL149C, YJL061W, YJR042W, YKL205W, YKR095W, YMR047C, YMR275C, YOL072W, YOR112W, YPR128C
GO:0006401	RNA catabolic process	15	488	3.07%	152	6485	2.34%	YBL105C, YCR035C, YCR093W, YGL213C, YJL208C, YJR006W, YKL025C, YKL220C, YKR095W, YLR442C, YMR116C, YNL004W, YNL118C, YNR052C, YOL072W
GO:0071554	cell wall organization or biogenesis	15	488	3.07%	197	6485	3.04%	YBL105C, YBR043C, YCR032W, YDR351W, YGL027C, YGR143W, YGR189C, YJL083W, YKL104C, YKL181W, YKR027W, YLR300W, YMR104C, YMR306W, YNL313C
GO:0070925	organelle assembly	14	488	2.87%	112	6485	1.73%	YBL058W, YBL063W, YBL105C, YBR127C, YCR093W, YHL023C, YHR115C, YHR167W, YJR138W, YLR431C, YNL118C, YNR007C, YOR057W, YOR058C
GO:0006468	protein phosphorylation	14	488	2.87%	126	6485	1.94%	YBL088C, YBR028C, YCR059C, YCR091W, YDL079C, YDR283C, YDR422C, YGL021W, YIL143C, YJL057C, YMR116C, YMR139W, YMR216C, YOL045W
GO:0032787	monocarboxylic acid metabolic process	13	488	2.66%	127	6485	1.96%	YCL040W, YDL170W, YDR191W, YDR539W, YER010C, YGR193C, YIR023W, YKL182W, YML054C, YMR207C, YOL136C, YOR040W, YPR128C
GO:0007005	mitochondrion organization	13	488	2.66%	282	6485	4.35%	YBL103C, YDR150W, YER154W, YIL022W, YIL146C, YLL040C, YML129C, YMR275C, YNL138W, YNR007C, YOR147W, YPR024W, YPR116W
GO:0006873	cellular ion homeostasis	13	488	2.66%	131	6485	2.02%	YBR127C, YBR295W, YCL017C, YDR184C, YDR495C, YER113C, YGL167C, YJL059W, YJL165C, YMR216C, YOR087W, YOR147W, YOR270C
GO:0070647	protein modification by small protein conjugation or removal	13	488	2.66%	164	6485	2.53%	YDR390C, YDR409W, YDR457W, YHR115C, YIR025W, YJL210W, YKL010C, YLR207W, YML111W, YMR223W, YMR275C, YNL119W, YPL191C
GO:0043934	sporulation	12	488	2.46%	131	6485	2.02%	YBL058W, YBR043C, YDR516C, YER020W, YHR119W, YIL017C, YLL033W, YLL040C, YMR139W, YMR224C, YMR306W, YNL231C

GO:0009451	RNA modification	12	488	2.46%	177	6485	2.73%	YCL017C, YDR165W, YGL050W, YGL236C, YGR169C, YKR086W, YLR197W, YML005W, YNL061W, YNL119W, YNL132W, YPL086C
GO:0006979	response to oxidative stress	12	488	2.46%	106	6485	1.63%	YER042W, YGR097W, YHR206W, YJR138W, YKL056C, YKL150W, YKR071C, YMR176W, YNL080C, YNL241C, YOL081W, YOR290C
GO:0018193	peptidyl-amino acid modification	12	488	2.46%	148	6485	2.28%	YBR028C, YDR390C, YDR409W, YFL024C, YGL150C, YHR068W, YHR099W, YHR119W, YLL022C, YMR216C, YMR223W, YPL254W
GO:0008033	tRNA processing	11	488	2.25%	118	6485	1.82%	YCL017C, YDR165W, YGL050W, YGL236C, YGR169C, YLR430W, YML005W, YMR087W, YNL119W, YPL086C, YPR116W
GO:0055086	nucleobase-containing small molecule metabolic process	11	488	2.25%	163	6485	2.51%	Q0085, Q0130, YCL040W, YCL047C, YCL050C, YGR193C, YJL130C, YMR271C, YOL136C, YOR128C, YOR142W
GO:0007114	cell budding	11	488	2.25%	56	6485	0.86%	YCR038C, YDR184C, YIL129C, YIR006C, YKR089C, YLL021W, YLR084C, YLR319C, YNL271C, YOL070C, YOR301W
GO:0006974	cellular response to DNA damage stimulus	11	488	2.25%	78	6485	1.20%	YBL023C, YBL051C, YBL088C, YDR283C, YDR499W, YER041W, YHR154W, YLR183C, YLR430W, YMR190C, YNL262W
GO:0042594	response to starvation	10	488	2.05%	64	6485	0.99%	YAL036C, YDR191W, YDR283C, YFL033C, YFR034C, YGL209W, YHL023C, YJL083W, YLL040C, YOR290C
GO:0006520	cellular amino acid metabolic process	10	488	2.05%	155	6485	2.39%	YDL170W, YFL030W, YGL184C, YHR137W, YIL051C, YIL116W, YIR023W, YJL130C, YJR109C, YPR145W
GO:0016570	histone modification	10	488	2.05%	110	6485	1.70%	YDR191W, YER169W, YFL024C, YGL150C, YHR099W, YHR119W, YIL112W, YLL022C, YMR223W, YPL254W
GO:0051052	regulation of DNA metabolic process	9	488	1.84%	119	6485	1.84%	YDR291W, YDR310C, YHR154W, YHR197W, YIL143C, YKL010C, YLR442C, YMR190C, YNL088W
GO:0007124	pseudohyphal growth	9	488	1.84%	76	6485	1.17%	YCL047C, YCR093W, YDR422C, YER020W, YER113C, YHL023C, YLL021W, YLR319C, YNL119W
GO:0007033	vacuole organization	9	488	1.84%	101	6485	1.56%	YBL058W, YDR135C, YDR495C, YHL023C, YJR138W, YLL015W, YLR431C, YNL323W, YNR007C
GO:0042274	ribosomal small subunit biogenesis	9	488	1.84%	137	6485	2.11%	YAL035W, YDR398W, YDR457W, YJL136C, YLR435W, YMR143W, YNL132W, YOR205C, YPL217C
GO:0042273	ribosomal large subunit biogenesis	9	488	1.84%	119	6485	1.84%	YDR457W, YHR197W, YIR012W, YKL014C, YLR002C, YLR435W, YMR049C, YNL061W, YOR272W

GO:0006354	DNA-templated transcription, elongation	9	488	1.84%	105	6485	1.62%	YCR093W, YDR194C, YER169W, YHR167W, YHR206W, YIL126W, YMR033W, YNR052C, YOL072W
GO:0006897	endocytosis	9	488	1.84%	102	6485	1.57%	YGL206C, YIL095W, YIR006C, YJL165C, YJL204C, YKR021W, YMR275C, YNL020C, YOR018W
GO:0006997	nucleus organization	8	488	1.64%	97	6485	1.50%	YCR086W, YDR457W, YGL092W, YJL039C, YJL073W, YJR042W, YMR047C, YPL186C
GO:0016197	endosomal transport	8	488	1.64%	86	6485	1.33%	YAL002W, YDR141C, YDR407C, YGL137W, YJL029C, YJL204C, YOR256C, YPL070W
GO:0006091	generation of precursor metabolites and energy	8	488	1.64%	80	6485	1.23%	YBL058W, YCL040W, YHR047C, YLR087C, YMR311C, YNL241C, YOL045W, YOL136C
GO:0002181	cytoplasmic translation	8	488	1.64%	197	6485	3.04%	YAL035W, YAL036C, YFL034C-A, YJL136C, YJL189W, YKL056C, YMR143W, YOL045W
GO:0032543	mitochondrial translation	8	488	1.64%	167	6485	2.58%	YBL090W, YCR003W, YDR175C, YIL051C, YML129C, YNR045W, YOR187W, YPR033C
GO:0048284	organelle fusion	7	488	1.43%	99	6485	1.53%	YDL058W, YDR135C, YDR189W, YIL004C, YJL073W, YKL212W, YLL015W
GO:0031399	regulation of protein modification process	7	488	1.43%	118	6485	1.82%	YCR059C, YDR409W, YGL150C, YHR119W, YIR025W, YLR207W, YMR116C
GO:0043543	protein acylation	7	488	1.43%	70	6485	1.08%	YFL024C, YGL150C, YHR099W, YHR119W, YLL022C, YOL003C, YPL254W
GO:0006352	DNA-templated transcription, initiation	7	488	1.43%	75	6485	1.16%	YBR049C, YGL207W, YIL143C, YIR023W, YKL144C, YNL118C, YPL082C
GO:0001403	invasive growth in response to glucose limitation	6	488	1.23%	54	6485	0.83%	YCL047C, YER020W, YER113C, YMR116C, YNL119W, YOR290C
GO:0016050	vesicle organization	6	488	1.23%	88	6485	1.36%	YDL058W, YDR189W, YEL022W, YHR098C, YIL004C, YKL212W
GO:0006457	protein folding	6	488	1.23%	98	6485	1.51%	YBL075C, YJL014W, YJL073W, YML130C, YPR024W, YPR037C
GO:0006865	amino acid transport	6	488	1.23%	46	6485	0.71%	YBR043C, YBR147W, YCR075C, YDR046C, YGR125W, YJL059W
GO:0006360	transcription by RNA polymerase I	5	488	1.02%	70	6485	1.08%	YBR049C, YDR398W, YJL025W, YPL082C, YPR010C
GO:0045333	cellular respiration	5	488	1.02%	89	6485	1.37%	Q0045, Q0105, Q0250, Q0275, YOR142W

GO:0007031	peroxisome organization	5	488	1.02%	50	6485	0.77%	YDL204W, YGR004W, YJL210W, YKL197C, YPR128C
GO:0006497	protein lipidation	5	488	1.02%	45	6485	0.69%	YJL062W, YJL091C, YLL031C, YNR007C, YOL003C
GO:0006869	lipid transport	5	488	1.02%	71	6485	1.09%	YCR091W, YLL040C, YNL087W, YNL231C, YNL323W
GO:0006970	response to osmotic stress	5	488	1.02%	68	6485	1.05%	YDR192C, YGR014W, YLR019W, YNL091W, YOR270C
GO:0000902	cell morphogenesis	5	488	1.02%	27	6485	0.42%	YDR085C, YDR141C, YHR158C, YIL129C, YLL021W
GO:0051604	protein maturation	5	488	1.02%	86	6485	1.33%	YHR068W, YLR087C, YML013W, YNL238W, YPR024W
GO:0048308	organelle inheritance	5	488	1.02%	55	6485	0.85%	YDL204W, YDR150W, YDR495C, YMR275C, YOR147W
GO:0000054	ribosomal subunit export from nucleus	4	488	0.82%	52	6485	0.80%	YGR218W, YJL061W, YJR042W, YMR047C
GO:0008643	carbohydrate transport	4	488	0.82%	36	6485	0.56%	YCL040W, YDR345C, YGR097W, YNL318C
GO:0006353	DNA-templated transcription, termination	4	488	0.82%	41	6485	0.63%	YBR049C, YDR301W, YLR277C, YLR430W
GO:0061025	membrane fusion	4	488	0.82%	51	6485	0.79%	YDL058W, YDR189W, YIL004C, YKL212W
GO:0006418	tRNA aminoacylation for protein translation	4	488	0.82%	36	6485	0.56%	YGL105W, YLR060W, YOR335C, YPR033C
GO:0042255	ribosome assembly	3	488	0.61%	64	6485	0.99%	YAL035W, YHR197W, YIR012W
GO:0043144	sno(s)RNA processing	3	488	0.61%	45	6485	0.69%	YCR035C, YLR277C, YLR430W
GO:0009408	response to heat	3	488	0.61%	53	6485	0.82%	YDR192C, YFL033C, YLR019W
GO:0006414	translational elongation	3	488	0.61%	333	6485	5.13%	YGL195W, YMR116C, YOR187W
GO:0006383	transcription by RNA polymerase III	3	488	0.61%	45	6485	0.69%	YDR362C, YGR047C, YKL144C
GO:0006887	exocytosis	3	488	0.61%	47	6485	0.72%	YGL233W, YIL118W, YPR032W
GO:0006413	translational initiation	3	488	0.61%	61	6485	0.94%	YAL035W, YBL090W, YDR283C
GO:0032196	transposition	2	488	0.41%	112	6485	1.73%	YHR154W, YIL143C
GO:0006470	protein dephosphorylation	2	488	0.41%	28	6485	0.43%	YGR123C, YLR019W

GO:0008213	protein alkylation	2	488	0.41%	52	6485	0.80%	YHR119W, YMR223W
GO:0006486	protein glycosylation	2	488	0.41%	64	6485	0.99%	YGL027C, YNL080C
GO:0006766	vitamin metabolic process	1	488	0.20%	52	6485	0.80%	YDL080C
GO:0009311	oligosaccharide metabolic process	1	488	0.20%	43	6485	0.66%	YDR001C

Table S3 Results from the statistical analysis of transcriptomic profiles. Here is shown the genes which were up- or down-regulated in evolved strains compared with the parental S288C.

**Genes which expression changed in the three evolved strains (TTY22, AT22 and TAT12) compared with the parental strain S288C**			
**Systematic Name**	**Expression**	**Standard Name**	**Name Description**
YAL049C	Over expressed	AIM2	Altered Inheritance rate of Mitochondria
YBR025C	Over expressed	OLA1	Obg-Like ATPase
YBR216C	Over expressed	YBP1	Yap1-Binding Protein
YDL061C	Over expressed	RPS29B	Ribosomal Protein of the Small subunit
YDR102C	Over expressed	YDR102C	Conserved among S. cerevisiae strains
YDR142C	Over expressed	PEX7	PEroXin
YDR385W	Over expressed	EFT2	Elongation Factor Two
YDR403W	Over expressed	DIT1	DITyrosine
YER069W	Over expressed	ARG5,6	ARGinine requiring
YFR048W	Over expressed	RMD8	Required for Meiotic nuclear Division
YGL008C	Over expressed	PMA1	Plasma Membrane ATPase
YGR168C	Over expressed	YGR168C	Putative protein of unknown function
YHR174W	Over expressed	ENO2	ENOlase
YJL086C	Over expressed	YJL086C	Unlikely to encode a functional protein, partially overlaps the verified genes YJL085W/EXO70 and YJL087C/TRL1
YLR058C	Over expressed	SHM2	Serine HydroxyMethyltransferase
YLR180W-R	Over expressed	SAM1	S-AdenosylMethionine requiring
YLR467W	Over expressed	YRF1-5	Helicase encoded by the Y' element of subtelomeric regions
YMR066W	Over expressed	SOV1	Synthesis Of Var
YOL060C	Over expressed	MAM3	Protein involved in magnesium homeostasis
YOR247W	Over expressed	SRL1	Suppressor of Rad53 null Lethality
YOR324C	Over expressed	FRT1	Functionally Related to TCP1
YBR042C	Down expressed	CST26	Chromosome STability
YBR110W	Down expressed	ALG1	Asparagine-Linked Glycosylation

YBR112C	Down expressed	CYC8	CYtochrome C
YCR015C	Down expressed	YCR015C	Putative protein of unknown function
YFL036W	Down expressed	RPO41	RNA POlymerase
YGR183C	Down expressed	QCR9	ubiQuinol-cytochrome C oxidoReductase
YHR062C	Down expressed	RPP1	Ribonuclease P Protein
YIL173W	Down expressed	VTH1	Vps Ten Homolog
YKL012W	Down expressed	PRP40	Pre-mRNA Processing
YMR027W	Down expressed	YMR027W	Putative protein of unknown function
YNL120C	Down expressed	YNL120C	Unlikely to encode a functional protein,
YPL075W	Down expressed	GCR1	GlyColysis Regulation
YPR076W	Down expressed	YPR076W	Unlikely to encode a functional protein
**Genes which expression changed in TTY22 and TAT12 evolved strains compared with the parental strain S288C**			
**Systematic Name**	**Expression**	**Standard Name**	**Name Description**
YDL203C	Over expressed	ACK1	Activator of C Kinase 1
YNL220W	Over expressed	ADE12	ADEnine requiring
YMR300C	Over expressed	ADE4	ADEnine requiring
Q0075	Over expressed	AI5_BETA	
YMR157C	Over expressed	AIM36	Altered Inheritance rate of Mitochondria
YPL158C	Over expressed	AIM44	Altered Inheritance rate of Mitochondria
YOR034C	Over expressed	AKR2	AnKyrin Repeat-containing protein
YGL021W	Over expressed	ALK1	
YJR047C	Over expressed	ANB1	ANaeroBically induced
YPR029C	Over expressed	APL4	clathrin Adaptor Protein complex Large chain
YLR242C	Over expressed	ARV1	ARE2 Required for Viability
YMR116C	Over expressed	ASC1	Absence of growth Suppressor of Cyp1
YLR189C	Over expressed	ATG26	AuTophaGy related
YAL020C	Over expressed	ATS1	Alpha Tubulin Suppressor
YKL004W	Over expressed	AUR1	AUreobasidin A Resistance
YJL095W	Over expressed	BCK1	Bypass of C Kinase
YER167W	Over expressed	BCK2	Bypass of C Kinase
YKL061W	Over expressed	BLI1	BLoc-1 Interactor
YNL233W	Over expressed	BNI4	Bud Neck Involved
YDR241W	Over expressed	BUD26	BUD site selection
YGR134W	Over expressed	CAF130	CCR4 Associated Factor
YLR110C	Over expressed	CCW12	Covalently linked Cell Wall protein
YDR134C	Over expressed	CCW22	Covalently bound Cell Wall protein
YBR131W	Over expressed	CCZ1	Calcium Caffeine Zinc sensitivity

YER061C	Over expressed	CEM1	Condensing Enzyme with Mitochondrial function
YER026C	Over expressed	CHO1	CHOline requiring
YNL192W	Over expressed	CHS1	CHitin Synthase
YLR346C	Over expressed	CIS1	CItrinin Sensitive knockout
YLR290C	Over expressed	COQ11	COenzyme Q
YNL336W	Over expressed	COS1	COnserved Sequence
YNR075W	Over expressed	COS10	COnserved Sequence
YDL248W	Over expressed	COS7	COnserved Sequence
YLL018C-A	Over expressed	COX19	Cytochrome c OXidase
YPL200W	Over expressed	CSM4	Chromosome Segregation in Meiosis
YHR175W	Over expressed	CTR2	Copper TRansport
YAL039C	Down expressed	CYC3	CYtochrome C
YLR348C	Over expressed	DIC1	DIcarboxylate Carrier
YAL026C	Over expressed	DRS2	Deficiency of Ribosomal Subunits
YNR067C	Over expressed	DSE4	Daughter Specific Expression
YBR252W	Over expressed	DUT1	DUTp pyrophosphatase
YMR176W	Over expressed	ECM5	ExtraCellular Mutant
YOR133W	Over expressed	EFT1	Elongation Factor Two
YPL086C	Over expressed	ELP3	ELongator Protein
YKL207W	Over expressed	EMC3	ER Membrane protein Complex
YGR254W	Over expressed	ENO1	ENOlase
YDR414C	Over expressed	ERD1	Endoplasmic reticulum Retention Defective
YGL139W	Over expressed	FLC3	FLavin Carrier
YBR179C	Over expressed	FZO1	FuZzy Onions homolog
YDR096W	Over expressed	GIS1	GIg1-2 Suppressor
YHR005C	Over expressed	GPA1	G Protein Alpha subunit
YJR013W	Over expressed	GPI14	GlycosylPhosphatidylInositol anchor biosynthesis
YGR102C	Over expressed	GTF1	Glutaminyl Transamidase subunit F
YDL238C	Over expressed	GUD1	GUanine Deaminase
YJL091C	Over expressed	GWT1	GPI-anchored Wall protein Transfer
YPL001W	Over expressed	HAT1	Histone AcetylTransferase
YLR192C	Over expressed	HCR1	High-Copy suppressor of Rpg1
YMR207C	Over expressed	HFA1	0
YNR055C	Over expressed	HOL1	HistidinOl
YBR272C	Over expressed	HSM3	enHanced Spontaneous Mutability
YOR025W	Over expressed	HST3	Homolog of SIR Two (SIR2)
YHR067W	Over expressed	HTD2	Hydroxyacyl-Thioester Dehydratase
YDL181W	Over expressed	INH1	INHibitor (of F1F0-ATPase)

YHR079C	Over expressed	IRE1	Inositol REquiring
YJR097W	Over expressed	JJJ3	J-protein (Type III)
YLL057C	Over expressed	JLP1	dnaJ-Like Protein
YJL034W	Over expressed	KAR2	KARyogamy
YDL049C	Over expressed	KNH1	Kre9(Nine) Homolog
YOR181W	Over expressed	LAS17	0
YOR171C	Over expressed	LCB4	Long-Chain Base
YOR108W	Over expressed	LEU9	LEUcine biosynthesis
YOR059C	Over expressed	LPL1	LD phospholipase
YGR057C	Over expressed	LST7	Lethal with Sec Thirteen
YBL091C	Over expressed	MAP2	Methionine AminoPeptidase
YHL018W	Over expressed	MCO14	Mitochondrial Class One protein of 14 kDa
YNL328C	Over expressed	MDJ2	Mitochondrial DnaJ homolog
YOL064C	Over expressed	MET22	METhionine requiring
YER091C	Over expressed	MET6	METhionine requiring
YGL089C	Over expressed	MF(ALPHA) 2	Mating Factor ALPHA
YJR077C	Over expressed	MIR1	Mitochondrial Import Receptor
YPR164W	Over expressed	MMS1	Methyl MethaneSulfonate sensitivity
YGL068W	Over expressed	MNP1	Mitochondrial-Nucleoid Protein
YPR112C	Over expressed	MRD1	Multiple RNA-binding domain
YDR205W	Over expressed	MSC2	Meiotic Sister-Chromatid recombination
YNL053W	Over expressed	MSG5	Multicopy Suppressor of GPA1
YGR023W	Over expressed	MTL1	Mid-Two Like
YGL236C	Over expressed	MTO1	Mitochondrial Translation Optimization
YNL063W	Over expressed	MTQ1	Methyltransferase
YBR119W	Over expressed	MUD1	Mutant U1 Die
YPL126W	Over expressed	NAN1	Net1 Associated Nuclear protein
YGL091C	Over expressed	NBP35	Nucleotide Binding Protein
YMR122W-A	Over expressed	NCW1	Novel Cell Wall protein
YLR007W	Over expressed	NSE1	Non-SMC Element
YMR153W	Over expressed	NUP53	NUclear Pore
YOR085W	Over expressed	OST3	OligoSaccharylTransferase
YDR538W	Over expressed	PAD1	Phenylacrylic Acid Decarboxylase
YLL064C	Over expressed	PAU18	seriPAUperin
YNL181W	Over expressed	PBR1	Potentiates Bioactive compound Response
YLR044C	Over expressed	PDC1	Pyruvate DeCarboxylase
YLR064W	Over expressed	PER33	Pore and ER protein, 33 kDa
YFR023W	Over expressed	PES4	Polymerase Epsilon Suppressor

YPL206C	Over expressed	PGC1	Phosphatidyl Glycerol phospholipase C
YPR154W	Over expressed	PIN3	Psi+ INducibility
YDL030W	Over expressed	PRP9	Pre-mRNA Processing
YDL055C	Over expressed	PSA1	0
YOL045W	Over expressed	PSK2	Pas domain-containing Serine/threonine protein Kinase
YBL056W	Over expressed	PTC3	Phosphatase Two C
YKL015W	Over expressed	PUT3	Proline UTilization
YOR347C	Over expressed	PYK2	PYruvate Kinase
YOL010W	Over expressed	RCL1	Rna 3'-terminal phosphate Cyclase Like
YBR073W	Over expressed	RDH54	RaD54 Homolog
YOR346W	Over expressed	REV1	REVersionless
YDR137W	Over expressed	RGP1	Reduced Growth Phenotype
YDR487C	Over expressed	RIB3	RIBoflavin biosynthesis
YLR371W	Over expressed	ROM2	RhO1 Multicopy suppressor
YDR156W	Over expressed	RPA14	RNA Polymerase A
YPR102C	Over expressed	RPL11A	Ribosomal Protein of the Large subunit
YKL006W	Over expressed	RPL14A	Ribosomal Protein of the Large subunit
YNL301C	Over expressed	RPL18B	Ribosomal Protein of the Large subunit
YFL034C-A	Over expressed	RPL22B	Ribosomal Protein of the Large subunit
YLR344W	Over expressed	RPL26A	Ribosomal Protein of the Large subunit
YGR034W	Over expressed	RPL26B	Ribosomal Protein of the Large subunit
YPL249C-A	Over expressed	RPL36B	Ribosomal Protein of the Large subunit
YPR043W	Over expressed	RPL43A	Ribosomal Protein of the Large subunit
YGL147C	Over expressed	RPL9A	Ribosomal Protein of the Large subunit
YDL147W	Over expressed	RPN5	Regulatory Particle Non-ATPase
YOR369C	Over expressed	RPS12	Ribosomal Protein of the Small subunit
YML063W	Over expressed	RPS1B	Ribosomal Protein of the Small subunit
YGL123W	Over expressed	RPS2	Ribosomal Protein of the Small subunit
YER102W	Over expressed	RPS8B	Ribosomal Protein of the Small subunit
YDR394W	Over expressed	RPT3	Regulatory Particle Triple-A protein, or Regulatory Particle Triphosphatase
YLR221C	Over expressed	RSA3	RiboSome Assembly
YLR033W	Over expressed	RSC58	Remodel the Structure of Chromatin
YMR214W	Over expressed	SCJ1	S. Cerevisiae DnaJ
YJL080C	Over expressed	SCP160	S. cerevisiae protein involved in the Control of Ploidy
YGL224C	Over expressed	SDT1	Suppressor of Disruption of TFIIS
YDR164C	Over expressed	SEC1	SECretory
YDL195W	Over expressed	SEC31	SECretory
YGL228W	Over expressed	SHE10	Sensitivity to High Expression

YLR442C	Over expressed	SIR3	Silent Information Regulator
YNL007C	Over expressed	SIS1	SIt4 Suppressor
YDR510W	Over expressed	SMT3	Suppressor of Mif Two
YMR096W	Over expressed	SNZ1	SNooZe
YOL091W	Over expressed	SPO21	SPOrulation
YGL184C	Over expressed	STR3	Sulfur TRansfer
YGL169W	Over expressed	SUA5	Suppressor of Upstream AUG
YLR426W	Over expressed	TDA5	Topoisomerase I Damage Affected
YJL052W	Over expressed	TDH1	Triose-phosphate DeHydrogenase
YBR118W	Over expressed	TEF2	Translation Elongation Factor
YLR178C	Over expressed	TFS1	cdc Twenty-Five Suppressor
YJL138C	Over expressed	TIF2	Translation Initiation Factor
YNR017W	Over expressed	TIM23	Translocase of the Inner Mitochondrial membrane
YGR260W	Over expressed	TNA1	Transporter of Nicotinic Acid
YBR162C	Over expressed	TOS1	Target Of Sbf
YDR165W	Over expressed	TRM82	Transfer RNA Methyltransferase
YDR092W	Over expressed	UBC13	UBiquitin-Conjugating
YLR420W	Over expressed	URA4	URAcil requiring
YHR026W	Over expressed	VMA16	Vacuolar Membrane Atpase
YGR020C	Over expressed	VMA7	Vacuolar Membrane Atpase
YOR043W	Over expressed	WHI2	WHIskey
YLR298C	Over expressed	YHC1	Yeast Homolog of human U1C
YMR241W	Over expressed	YHM2	Yeast suppressor of HM mutant
YBR111C	Over expressed	YSA1	0
YGR016W	Over expressed		
YJR120W	Over expressed		
YLL054C	Over expressed		
YNL234W	Over expressed		
YOR131C	Over expressed		
YOR283W	Over expressed		
ARA18	Over expressed		
YDL187C	Over expressed		
YDR210W-D	Over expressed		
YDR233C-R	Over expressed		
YGL088W	Over expressed		
YGL152C	Over expressed		
YGL188C	Over expressed		
YGR035C	Over expressed		

YGR192C-R	Over expressed		
YGR254W-R	Over expressed		
YGR291C	Over expressed		
YHR095W	Over expressed		
YHR183W-R	Over expressed		
YJL067W	Over expressed		
YJR020W	Over expressed		
YJR128W	Over expressed		
YKL060C-R	Over expressed		
YLR058C-R	Over expressed		
YLR458W	Over expressed		
YLR462W	Over expressed		
YML020W	Over expressed		
YNL143C	Over expressed		
YOR029W	Over expressed		
YLR027C	Down expressed	AAT2	Aspartate AminoTransferase
YJR105W	Down expressed	ADO1	ADenOsine kinase
YPL259C	Down expressed	APM1	clathrin Adaptor Protein complex Medium chain
YIL130W	Down expressed	ASG1	Activator of Stress Genes
YLR155C	Down expressed	ASP3-1	ASParaginase
YLR157C	Down expressed	ASP3-2	ASParaginase
YLR158C	Down expressed	ASP3-3	ASParaginase
YLR160C	Down expressed	ASP3-4	ASParaginase
YLL042C	Down expressed	ATG10	AuTophaGy related
YJL178C	Down expressed	ATG27	AuTophaGy related
YMR301C	Down expressed	ATM1	ABC Transporter, Mitochondrial
YML116W	Down expressed	ATR1	AminoTriazole Resistance
YMR279C	Down expressed	ATR2	AminoTriazole Resistance
YPR122W	Down expressed	AXL1	AXiaL budding
YOL164W	Down expressed	BDS1	Bacterially Derived Sulfatase
YNL275W	Down expressed	BOR1	BORon transporter
YNR027W	Down expressed	BUD17	BUD site selection
YOR299W	Down expressed	BUD7	BUD site selection
YPL215W	Down expressed	CBP3	Cytochrome B mRNA Processing
YOR112W	Down expressed	CEX1	Cytoplasmic EXport protein
YBR023C	Down expressed	CHS3	CHitin Synthase-related
YPR119W	Down expressed	CLB2	CycLin B

YLR003C	Down expressed	CMS1	Complementation of Mcm-10 Suppressor
YML071C	Down expressed	COG8	Conserved Oligomeric Golgi complex
YDR204W	Down expressed	COQ4	COenzyme Q
YBR036C	Down expressed	CSG2	Calcium Sensitive Growth
YBR161W	Down expressed	CSH1	CSG1/SUR1 Homolog
YOL145C	Down expressed	CTR9	Cln Three (CLN3) Requiring
YLR323C	Down expressed	CWC24	Complexed With Cef1p
YLR129W	Down expressed	DIP2	DOM34 Interacting Protein
YEL030W	Down expressed	ECM10	ExtraCellular Mutant
YOR092W	Down expressed	ECM3	ExtraCellular Mutant
YLR299W	Down expressed	ECM38	ExtraCellular Mutant
YKR096W	Down expressed	ESL2	EST/SMG-like
YMR306W	Down expressed	FKS3	FK506 Sensitivity
YAR050W	Down expressed	FLO1	FLOcculation
YER182W	Down expressed	FMP10	Found in Mitochondrial Proteome
YBL042C	Down expressed	FUI1	5-FlUorourIdine resistance
YDR019C	Down expressed	GCV1	GlyCine cleaVage
YLR445W	Down expressed	GMC2	Grand Meiotic recombination Cluster
YOR358W	Down expressed	HAP5	Heme Activator Protein
YEL056W	Down expressed	HAT2	Histone AcetylTransferase
YBL032W	Down expressed	HEK2	HEterogeneous nuclear rnp K-like gene
YOR227W	Down expressed	HER1	Hmg2p ER Remodeling
YOR038C	Down expressed	HIR2	HIstone Regulation
YBR215W	Down expressed	HPC2	Histone Periodic Control
YJL057C	Down expressed	IKS1	0
YDR492W	Down expressed	IZH1	Implicated in Zinc Homeostasis
YFR042W	Down expressed	KEG1	Kre6-binding ER protein responsible for Glucan synthesis
YNL322C	Down expressed	KRE1	Killer toxin REsistant
YBR199W	Down expressed	KTR4	Kre Two Related
YKR063C	Down expressed	LAS1	Lethal in the Absence of SSD1-v
YNL006W	Down expressed	LST8	Lethal with Sec Thirteen
YER106W	Down expressed	MAM1	Monopolar microtubule Attachment during Meiosis I
YPR046W	Down expressed	MCM16	MiniChromosome Maintenance
YBL023C	Down expressed	MCM2	MiniChromosome Maintenance
YJR024C	Down expressed	MDE1	Methylthioribulose-1-phosphate DEhydratase
YKL085W	Down expressed	MDH1	Malate DeHydrogenase
YML062C	Down expressed	MFT1	Mitochondrial Fusion Targeting
YOR211C	Down expressed	MGM1	Mitochondrial Genome Maintenance

YBR262C	Down expressed	MIC12	MItochondrial contact site and Cristae organizing system
YGL209W	Down expressed	MIG2	Multicopy Inhibitor of GAL gene expression
YIL106W	Down expressed	MOB1	Mps One Binder
YOR274W	Down expressed	MOD5	tRNA MODification
YGL080W	Down expressed	MPC1	Mitochondrial Pyruvate Carrier
YGR220C	Down expressed	MRPL9	Mitochondrial Ribosomal Protein, Large subunit
YMR188C	Down expressed	MRPS17	Mitochondrial Ribosomal Protein, Small subunit
YBL095W	Down expressed	MRX3	Mitochondrial oRganization of gene eXpression (MIOREX)
YGL226W	Down expressed	MTC3	Maintenance of Telomere Capping
YNL137C	Down expressed	NAM9	Nuclear Accommodation of Mitochondria
YML031W	Down expressed	NDC1	Nuclear Division Cycle
YHL023C	Down expressed	NPR3	Nitrogen Permease Regulator
YML065W	Down expressed	ORC1	Origin Recognition Complex
YDR488C	Down expressed	PAC11	Perish in the Absence of CIN8
YIL013C	Down expressed	PDR11	Pleiotropic Drug Resistance
YOL147C	Down expressed	PEX11	PEroXin
YNL158W	Down expressed	PGA1	Processing of Gas1p and ALP
YDR281C	Down expressed	PHM6	PHosphate Metabolism
YIL045W	Down expressed	PIG2	Protein Interacting with Gsy2p
YDL093W	Down expressed	PMT5	Protein O-MannosylTransferase
YGR199W	Down expressed	PMT6	Protein O-MannosylTransferase
YDR307W	Down expressed	PMT7	0
YEL055C	Down expressed	POL5	POLymerase
YER099C	Down expressed	PRS2	PhosphoRibosylpyrophosphate Synthetase
YKL077W	Down expressed	PSG1	Pma1 Stabilization in the Golgi
YKR093W	Down expressed	PTR2	Peptide TRansport
YNR062C	Down expressed	PUL3	PULcherrimin
YOR368W	Down expressed	RAD17	RADiation sensitive
YCR066W	Down expressed	RAD18	RADiation sensitive
YER173W	Down expressed	RAD24	RADiation sensitive
YOR048C	Down expressed	RAT1	Ribonucleic Acid Trafficking
YCR036W	Down expressed	RBK1	RiBoKinase
YBR052C	Down expressed	RFS1	Rad55 (Fifty-five) Suppressor
YDR379W	Down expressed	RGA2	Rho GTPase Activating Protein
YOR119C	Down expressed	RIO1	RIght Open reading frame
YLR009W	Down expressed	RLP24	Ribosomal-Like Protein
YPL024W	Down expressed	RMI1	RecQ Mediated genome Instability
YGL250W	Down expressed	RMR1	Reduced Meiotic Recombination

YMR061W	Down expressed	RNA14	poly(A) mRNA metabolism
YGL135W	Down expressed	RPL1B	Ribosomal Protein of the Large subunit
YPR116W	Down expressed	RRG8	Required for Respiratory Growth
YDR143C	Down expressed	SAN1	Sir Antagonist
YGR263C	Down expressed	SAY1	Steryl Acetyl hYdrolase
YER147C	Down expressed	SCC4	Sister Chromatid Cohesion
YMR086W	Down expressed	SEG1	Stability of Eisosomes Guaranteed
YKL130C	Down expressed	SHE2	Swi5p-dependent HO Expression
YNL311C	Down expressed	SKP2	homology with human SKP2
YDR515W	Down expressed	SLF1	SuLFide production
YIL105C	Down expressed	SLM1	Synthetic Lethal with Mss4
YHR066W	Down expressed	SSF1	Suppressor of ste4 (Four)
YDR443C	Down expressed	SSN2	Suppressor of SNf1
YDR169C	Down expressed	STB3	Sin Three Binding protein
YHL007C	Down expressed	STE20	STErile
YIL126W	Down expressed	STH1	SNF Two Homolog
YIL162W	Down expressed	SUC2	SUCrose
YMR149W	Down expressed	SWP1	Suppressor of a WbP1 mutation
YMR227C	Down expressed	TAF7	TATA binding protein-Associated Factor
YEL048C	Down expressed	TCA17	TRAPP Complex Associated protein
YDR362C	Down expressed	TFC6	Transcription Factor C
YOR052C	Down expressed	TMC1	Trivalent Metalloid sensitive, Cuz1-related protein
YOL124C	Down expressed	TRM11	TRna Methyltransferase
YBR082C	Down expressed	UBC4	UBiquitin-Conjugating
YDL210W	Down expressed	UGA4	Utilization of GAba
YKL099C	Down expressed	UTP11	U Three Protein
YPL120W	Down expressed	VPS30	Vacuolar Protein Sorting
YPR173C	Down expressed	VPS4	Vacuolar Protein Sorting
YGL060W	Down expressed	YBP2	Yap1-Binding Protein
YKL095W	Down expressed	YJU2	0
YPR058W	Down expressed	YMC1	Yeast Mitochondrial Carrier
YOR064C	Down expressed	YNG1	Yeast iNG1 homolog
YGR281W	Down expressed	YOR1	Yeast Oligomycin Resistance
YDL235C	Down expressed	YPD1	tYrosine Phosphatase Dependent
YBR219C	Down expressed		
YBR241C	Down expressed		
YDR391C	Down expressed		
YDR444W	Down expressed		

YEL068C	Down expressed		
YER181C	Down expressed		
YGL204C	Down expressed		
YJR056C	Down expressed		
YKR075C	Down expressed		
YMR155W	Down expressed		
YMR196W	Down expressed		
YNL122C	Down expressed		
YOL075C	Down expressed		
YAR061W	Down expressed		
YBL053W	Down expressed		
YBL086C	Down expressed		
YBR144C	Down expressed		
YCR041W	Down expressed		
YCR103C	Down expressed		
YDR098C-R	Down expressed		
YDR149C	Down expressed		
YDR173C-R	Down expressed		
YDR215C	Down expressed		
YDR327W	Down expressed		
YDR401W	Down expressed		
YDR491C	Down expressed		
YDR526C	Down expressed		
YDR537C	Down expressed		
YER187W	Down expressed		
YFL043C	Down expressed		
YGL177W	Down expressed		
YIL054W	Down expressed		
YIL151C	Down expressed		
YJL007C	Down expressed		
YJL169W	Down expressed		
YLL056C	Down expressed		
YLR230W	Down expressed		
YLR279W	Down expressed		
YLR463C	Down expressed		
YML048W-A	Down expressed		
YNL266W	Down expressed		
YOL046C	Down expressed		

YOR170W	Down expressed		
YOR235W	Down expressed		
YPL034W	Down expressed		
YPR092W	Down expressed		
YPR096C	Down expressed		

Genes which expression changed in TAT12 and AT22 evolved strains compared with the parental strain S288C

**Systematic Name**	**Expression**	**Standard Name**	**Name Description**
YPL005W	Over expressed	AEP3	ATPase ExPression
YOR026W	Over expressed	BUB3	Budding Uninhibited by Benzimidazole
YDR182W	Over expressed	CDC1	Cell Division Cycle
YDL220C	Over expressed	CDC13	Cell Division Cycle
YPL049C	Over expressed	DIG1	Down-regulator of Invasive Growth
YDR480W	Over expressed	DIG2	Down-regulator of Invasive Growth
YGR071C	Over expressed	ENV11	late ENdosome and Vacuole interface function
YFL021W	Over expressed	GAT1	0
YLR091W	Over expressed	GEP5	GEnetic interactors of Prohibitins
YER020W	Over expressed	GPA2	G Protein Alpha subunit
YHL032C	Over expressed	GUT1	Glycerol UTilization
YOR032C	Over expressed	HMS1	High-copy Mep Suppressor
YJL106W	Over expressed	IME2	Inducer of MEiosis
YJR138W	Over expressed	IML1	Increased Minichromosome Loss
YBR140C	Over expressed	IRA1	Inhibitory Regulator of the RAS-cAMP pathway
YFL042C	Over expressed	LAM5	Lipid transfer protein Anchored at Membrane contact site
YJL055W	Over expressed	LOG1	LOnely Guy
YPL004C	Over expressed	LSP1	Long chain bases Stimulate Phosphorylation
YGL136C	Over expressed	MRM2	Mitochondrial rRNA Methyl transferase
YOR370C	Over expressed	MRS6	Mitochondrial RNA Splicing
YMR100W	Over expressed	MUB1	MUlti Budding
YNL129W	Over expressed	NRK1	Nicotinamide Riboside Kinase
YIR006C	Over expressed	PAN1	Poly(A)-binding protein-dependent poly(A) riboNuclease
YOR158W	Over expressed	PET123	PETite colonies
YFR034C	Over expressed	PHO4	PHOsphate metabolism
YJL198W	Over expressed	PHO90	PHOsphate metabolism
YNL055C	Over expressed	POR1	PORin
YBR125C	Over expressed	PTC4	Phosphatase Two C
YHR001W-A	Over expressed	QCR10	ubiQuinol-cytochrome C oxidoReductase
YPL143W	Over expressed	RPL33A	Ribosomal Protein of the Large subunit

YPL183C	Over expressed	RTT10	Regulator of Ty1 Transposition
YJR004C	Over expressed	SAG1	Sexual AGglutination
YGL115W	Over expressed	SNF4	Sucrose NonFermenting
YBR289W	Over expressed	SNF5	Sucrose NonFermenting
YKR037C	Over expressed	SPC34	Spindle Pole Component
YNL202W	Over expressed	SPS19	SPorulation-Specific
YML100W	Over expressed	TSL1	Trehalose Synthase Long chain
YJL130C	Over expressed	URA2	URAcil requiring
YIL091C	Over expressed	UTP25	U Three Protein
YML115C	Over expressed	VAN1	VANadate resistance protein
YKR020W	Over expressed	VPS51	Vacuolar Protein Sorting
YDL120W	Over expressed	YFH1	Yeast Frataxin Homolog
YCL074W	Over expressed		
YDR061W	Over expressed		
YER160C	Over expressed		
YGR026W	Over expressed		
YGR161W-A	Over expressed		
YPR158C-D	Over expressed		
YBR124W	Over expressed		
YCL065W	Over expressed		
YDR544C	Over expressed		
YGR139W	Over expressed		
YJL150W	Over expressed		
YJR115W-R	Over expressed		
YKL096W-A-R	Over expressed		
YLR169W	Over expressed		
YLR296W	Over expressed		
YLR342W-R	Over expressed		
YLR365W	Over expressed		
YIL158W	Down expressed	AIM20	Altered Inheritance rate of Mitochondria
YMR092C	Down expressed	AIP1	Actin Interacting Protein
YGR224W	Down expressed	AZR1	Acetic Acid and AZoles Resistance
YML077W	Down expressed	BET5	Blocked Early in Transport
YER016W	Down expressed	BIM1	BInding to Microtubules
YIL142W	Down expressed	CCT2	Chaperonin Containing TCP-1
YJR076C	Down expressed	CDC11	Cell Division Cycle
YHR052W	Down expressed	CIC1	Core Interacting Component

YIL157C	Down expressed	COA1	Cytochrome Oxidase Assembly
YGL223C	Down expressed	COG1	Conserved Oligomeric Golgi complex
YPR158W	Down expressed	CUR1	Curing of [URe3]
YHR019C	Down expressed	DED81	Defines Essential Domain
YIL010W	Down expressed	DOT5	Disruptor Of Telomeric silencing
YKL172W	Down expressed	EBP2	EBNA1-binding protein (homolog)
YAL059W	Down expressed	ECM1	ExtraCellular Mutant
YDR518W	Down expressed	EUG1	ER protein Unnecessary for Growth
YIL131C	Down expressed	FKH1	ForK head Homolog
YOR258W	Down expressed	HNT3	Histidine triad NucleoTide-binding
YJL214W	Down expressed	HXT8	HeXose Transporter
YPR006C	Down expressed	ICL2	IsoCitrate Lyase
YOL108C	Down expressed	INO4	INOsitol requiring
YCR020C-A	Down expressed	MAK31	MAintenance of Killer
YBR227C	Down expressed	MCX1	Mitochondrial ClpX
YJR160C	Down expressed	MPH3	Maltose Permease Homolog
YDL045W-A	Down expressed	MRP10	Mitochondrial Ribosomal Protein
YOL042W	Down expressed	NGL1	0
YML118W	Down expressed	NGL3	0
YLR138W	Down expressed	NHA1	Na+/H+ Antiporter
YDR383C	Down expressed	NKP1	Non-essential Kinetochore Protein
YPL093W	Down expressed	NOG1	NucleOlar G-protein
YBL068W	Down expressed	PRS4	PhosphoRibosylpyrophosphate Synthetase
YHL027W	Down expressed	RIM101	Regulator of IME2
YBL087C	Down expressed	RPL23A	Ribosomal Protein of the Large subunit
YHR027C	Down expressed	RPN1	Regulatory Particle Non-ATPase
YCL031C	Down expressed	RRP7	Ribosomal RNA Processing
YBR269C	Down expressed	SDH8	Succinate DeHydrogenase
YOR315W	Down expressed	SFG1	SuperFicial pseudohyphal Growth
YGR271W	Down expressed	SLH1	SKI2-Like Helicase
YPL002C	Down expressed	SNF8	Sucrose NonFermenting
YEL031W	Down expressed	SPF1	Sensitivity to Pichia Farinosa killer toxin
YOR313C	Down expressed	SPS4	SPorulation Specific trancript
YDR293C	Down expressed	SSD1	Suppressor of SIT4 Deletion
YGR008C	Down expressed	STF2	STabilizing Factor
YGR046W	Down expressed	TAM41	Translocator Assembly and Maintenance
YHR003C	Down expressed	TCD1	tRNA ThreonylCarbamoyladenosine Dehydratase
YBR123C	Down expressed	TFC1	Transcription Factor class C

YHR025W	Down expressed	THR1	THReonine requiring
YER184C	Down expressed	TOG1	Transcriptional regulator of Oleate utilization Genes
YJR066W	Down expressed	TOR1	Target Of Rapamycin
YBR006W	Down expressed	UGA2	Utilization of GAba
YDR520C	Down expressed	URC2	URacil Catabolism
YKL041W	Down expressed	VPS24	Vacuolar Protein Sorting
YCR087C-A	Down expressed		
YGL159W	Down expressed		
YIL067C	Down expressed		
YJR098C	Down expressed		
YKR045C	Down expressed		
YBR064W	Down expressed		
YDR475C	Down expressed		
YGR022C	Down expressed		
YGR053C	Down expressed		
YML084W	Down expressed		
YMR306C-A	Down expressed		
**Genes which expression changed in TTY23 and AT22 evolved strains compared with the parental strain S288C**			
**Systematic Name**	**Expression**	**Standard Name**	**Name Description**
YFL039C	Over expressed	ACT1	ACTin
YLR040C	Over expressed	AFB1	A-Factor Barrier
YER017C	Over expressed	AFG3	ATPase Family Gene
YDR321W	Over expressed	ASP1	ASParaginase
YLR356W	Over expressed	ATG33	AuTophaGy related
YPL048W	Over expressed	CAM1	Calcium And Membrane-binding protein
YEL063C	Over expressed	CAN1	CANavanine resistance
YNR041C	Over expressed	COQ2	COenzyme Q
YGR295C	Over expressed	COS6	COnserved Sequence
YPL172C	Over expressed	COX10	Cytochrome c OXidase
YOL052C-A	Over expressed	DDR2	DNA Damage Responsive
YOR080W	Over expressed	DIA2	Digs Into Agar
YDR069C	Over expressed	DOA4	Degradation Of Alpha
YER140W	Over expressed	EMP65	ER Membrane Protein of 65 kDa
YMR049C	Over expressed	ERB1	Eukaryotic Ribosome Biogenesis
YDR236C	Over expressed	FMN1	FMN biosynthesis
YPL262W	Over expressed	FUM1	FUMarase
YHR183W	Over expressed	GND1	6-phosphoGlucoNateDehydrogenase
YDR437W	Over expressed	GPI19	Glycosyl PhosphatidylInositol anchor biosynthesis

YDR454C	Over expressed	GUK1	GUanylate Kinase
YNL004W	Over expressed	HRB1	Hypothetical RNA-Binding protein
YGL253W	Over expressed	HXK2	HeXoKinase
YDL182W	Over expressed	LYS20	LYSine requiring
YCR019W	Over expressed	MAK32	MAintenance of Killer
YGL125W	Over expressed	MET13	METhionine requiring
YFR011C	Over expressed	MIC19	MItochondrial contact site and Cristae organizing system
YGR076C	Over expressed	MRPL25	Mitochondrial Ribosomal Protein, Large subunit
YDR097C	Over expressed	MSH6	MutS Homolog
YDR128W	Over expressed	MTC5	Maintenance of Telomere Capping
YDR397C	Over expressed	NCB2	Negative Cofactor B
YGR119C	Over expressed	NUP57	NUclear Pore
YLR134W	Over expressed	PDC5	Pyruvate DeCarboxylase
YOL136C	Over expressed	PFK27	6-PhosphoFructo-2-Kinase
YPL096W	Over expressed	PNG1	Peptide N-Glycanase
YOR362C	Over expressed	PRE10	PRoteinase yscE
YGL063W	Over expressed	PUS2	PseudoUridine Synthase
YPL153C	Over expressed	RAD53	RADiation sensitive
YLR107W	Over expressed	REX3	Rna EXonuclease
YJL177W	Over expressed	RPL17B	Ribosomal Protein of the Large subunit
YHR010W	Over expressed	RPL27A	Ribosomal Protein of the Large subunit
YDR500C	Over expressed	RPL37B	Ribosomal Protein of the Large subunit
YHR030C	Over expressed	SLT2	Suppressor of the LyTic phenotype
YDR189W	Over expressed	SLY1	Suppressor of Loss of Ypt1
YDR425W	Over expressed	SNX41	Sorting NeXin
YGR248W	Over expressed	SOL4	Suppressor Of Los1-1
YER148W	Over expressed	SPT15	SuPpressor of Ty insertions
YDL229W	Over expressed	SSB1	Stress-Seventy subfamily B
YOR081C	Over expressed	TGL5	TriacylGlycerol Lipase
YMR146C	Over expressed	TIF34	Translation Initiation Factor
YGL145W	Over expressed	TIP20	SEC20 (Twenty) Interacting Protein
YOR010C	Over expressed	TIR2	TIp1-Related
YMR218C	Over expressed	TRS130	TRapp Subunit
YDR084C	Over expressed	TVP23	Tlg2-Vesicle Protein
YPR036W	Over expressed	VMA13	Vacuolar Membrane Atpase
YDR089W	Over expressed	VTC5	Vacuole Transporter Chaperone
YDR135C	Over expressed	YCF1	Yeast Cadmium Factor
YKL214C	Over expressed	YRA2	Yeast RNA Annealing protein

YCL075W	Over expressed		
YEL077C	Over expressed		
YIL092W	Over expressed		
YIL165C	Over expressed		
YKR078W	Over expressed		
YPL277C	Over expressed		
YDR029W	Over expressed		
YGL008C-R	Over expressed		
YGL262W	Over expressed		
YGR115C	Over expressed		
YHR212C	Over expressed		
YIL025C	Over expressed		
YJL159W-R	Over expressed		
YLR217W	Over expressed		
YMR122W-A-R	Over expressed		
YOL061W-R	Over expressed		
YOR199W	Over expressed		
YPR015C	Over expressed		
YAR015W	Down expressed	ADE1	ADEnine requiring
Q0050	Down expressed	AI1	
Q0065	Down expressed	AI4	
YGL160W	Down expressed	AIM14	Altered Inheritance rate of Mitochondria
YMR169C	Down expressed	ALD3	ALdehyde Dehydrogenase
YER073W	Down expressed	ALD5	ALdehyde Dehydrogenase
YPR199C	Down expressed	ARR1	ARsenicals Resistance
YLR423C	Down expressed	ATG17	AuTophaGy related
YBL089W	Down expressed	AVT5	Amino acid Vacuolar Transport
YHL038C	Down expressed	CBP2	Cytochrome B mRNA Processing
YMR138W	Down expressed	CIN4	Chromosome INstability
YGR255C	Down expressed	COQ6	COenzyme Q
YDR151C	Down expressed	CTH1	Cysteine-Three-Histidine
YDR121W	Down expressed	DPB4	DNA Polymerase B (II) subunit
YDL022W	Down expressed	GPD1	Glycerol-3-Phosphate Dehydrogenase
YGR154C	Down expressed	GTO1	Glutathione Transferase Omega-like
YKR051W	Down expressed	HFL1	Has Fused Lysosomes
YLL026W	Down expressed	HSP104	Heat Shock Protein
YNL318C	Down expressed	HXT14	HeXose Transporter

YMR035W	Down expressed	IMP2	Inner Membrane Protease
YLR021W	Down expressed	IRC25	Increased Recombination Centers
YAL010C	Down expressed	MDM10	Mitochondrial Distribution and Morphology
YKL053C-A	Down expressed	MDM35	Mitochondrial Distribution and Morphology
YOR174W	Down expressed	MED4	MEDiator complex
YOR150W	Down expressed	MRPL23	Mitochondrial Ribosomal Protein, Large subunit
YGR257C	Down expressed	MTM1	Manganese Trafficking factor for Mitochondrial SOD2
YAL029C	Down expressed	MYO4	MYOsin
YDR162C	Down expressed	NBP2	Nap1 Binding Protein
YIL164C	Down expressed	NIT1	NITrilase superfamily
YJR132W	Down expressed	NMD5	Nonsense-Mediated mRNA Decay
YPL196W	Down expressed	OXR1	OXidation Resistance
YGL261C	Down expressed	PAU11	seriPAUperin
YAL032C	Down expressed	PRP45	Pre-mRNA Processing
YER075C	Down expressed	PTP3	Protein Tyrosine Phosphatase
YBR218C	Down expressed	PYC2	PYruvate Carboxylase
YAL036C	Down expressed	RBG1	RiBosome interacting Gtpase
YBR002C	Down expressed	RER2	Retention in the Endoplasmic Reticulum
YHR031C	Down expressed	RRM3	rDNA Recombination Mutation
YBL025W	Down expressed	RRN10	Regulation of RNA polymerase I
YDR180W	Down expressed	SCC2	Sister Chromatid Cohesion
YBR024W	Down expressed	SCO2	Suppressor of Cytochrome Oxidase deficiency
YLR026C	Down expressed	SED5	Suppressor of Erd2 Deletion
YDL212W	Down expressed	SHR3	Super high Histidine Resistant
YAL047C	Down expressed	SPC72	Spindle Pole Component
YDR104C	Down expressed	SPO71	SPOrulation
YLR369W	Down expressed	SSQ1	Stress-Seventy subfamily Q
YDR082W	Down expressed	STN1	Suppressor of cdc ThirteeN
YPL258C	Down expressed	THI21	THIamine metabolism
YLR327C	Down expressed	TMA10	Translation Machinery Associated
YML021C	Down expressed	UNG1	Uracil DNA N-Glycosylase
YLR272C	Down expressed	YCS4	Yeast Condensin Subunit
YGR161C-C	Down expressed		
YBR224W	Down expressed		
YDR193W	Down expressed		
YDR360W	Down expressed		
YEL010W	Down expressed		
YGL072C	Down expressed		

YGL242C	Down expressed		
YHL045W	Down expressed		
YIL059C	Down expressed		
YJL119C	Down expressed		
YJR146W	Down expressed		
YLR416C	Down expressed		
YOL029C	Down expressed		
YOR345C	Down expressed		
ARA4	Down expressed		
ARA6	Down expressed		
**Genes which expression changed in TTY22 evolved strain compared with the parental strain S288C**			
**Systematic Name**	**Expression**	**Standard Name**	**Name Description**
YFL056C	Over expressed	AAD6	Aryl-Alcohol Dehydrogenase
YNR016C	Over expressed	ACC1	Acetyl-CoA Carboxylase
YGL256W	Over expressed	ADH4	Alcohol DeHydrogenase
YGL071W	Over expressed	AFT1	Activator of Ferrous Transport
YDL073W	Over expressed	AHK1	Associated with HKr1
YIR003W	Over expressed	AIM21	Altered Inheritance rate of Mitochondria
YHR199C	Over expressed	AIM46	Altered Inheritance rate of Mitochondria
YIL079C	Over expressed	AIR1	Arginine methyltransferase-Interacting RING finger protein
YMR170C	Over expressed	ALD2	ALdehyde Dehydrogenase
YOR067C	Over expressed	ALG8	Asparagine-Linked Glycosylation
YLR089C	Over expressed	ALT1	ALanine Transaminase
YBR158W	Over expressed	AMN1	Antagonist of Mitotic exit Network
YPR128C	Over expressed	ANT1	Adenine Nucleotide Transporter
YOL062C	Over expressed	APM4	clathrin Adaptor Protein complex Medium chain
YDR441C	Over expressed	APT2	Adenine PhosphoribosylTransferase
YLL052C	Over expressed	AQY2	AQuaporin from Yeast
YLR370C	Over expressed	ARC18	ARp2/3 Complex subunit
YBR249C	Over expressed	ARO4	AROmatic amino acid requiring
YPR060C	Over expressed	ARO7	AROmatic amino acid requiring
YGL202W	Over expressed	ARO8	AROmatic amino acid requiring
YDR106W	Over expressed	ARP10	Actin-Related Protein
YGR097W	Over expressed	ASK10	Activator of SKn7
YGR124W	Over expressed	ASN2	ASparagiNe requiring
YPL100W	Over expressed	ATG21	AuTophaGy related
YLR211C	Over expressed	ATG38	AuTophaGy related

YLR312C	Over expressed	ATG39	AuTophaGy related
YPL250C	Over expressed	ATG41	AuTophaGy related
YJR121W	Over expressed	ATP2	ATP synthase
YDR350C	Over expressed	ATP22	ATPase synthase
YOR079C	Over expressed	ATX2	AnTioXidant
YHR208W	Over expressed	BAT1	Branched-chain Amino acid Transaminase
YPL115C	Over expressed	BEM3	Bud EMergence
YNR056C	Over expressed	BIO5	BIOtin
YDR099W	Over expressed	BMH2	Brain Modulosignalin Homolog
YJL060W	Over expressed	BNA3	Biosynthesis of Nicotinic Acid
YLR078C	Over expressed	BOS1	Bet One Suppressor
YDL141W	Over expressed	BPL1	Biotin:apoProtein Ligase
YGR246C	Over expressed	BRF1	B-Related Factor
YPL069C	Over expressed	BTS1	Bet Two Suppressor
YDR531W	Over expressed	CAB1	Coenzyme A Biosynthesis
YGR277C	Over expressed	CAB4	Coenzyme A Biosynthesis
YML102W	Over expressed	CAC2	Chromatin Assembly Complex
YGR036C	Over expressed	CAX4	CAlmodulin-dependent in cmd one two twenty-siX
YDR197W	Over expressed	CBS2	Cytochrome B Synthesis
YDL143W	Over expressed	CCT4	Chaperonin Containing TCP-1
YCR002C	Over expressed	CDC10	Cell Division Cycle
YHR166C	Over expressed	CDC23	Cell Division Cycle
YFR036W	Over expressed	CDC26	Cell Division Cycle
YGL155W	Over expressed	CDC43	Cell Division Cycle
YDL164C	Over expressed	CDC9	Cell Division Cycle
YLR115W	Over expressed	CFT2	Cleavage Factor Two
YCL064C	Over expressed	CHA1	Catabolism of Hydroxy Amino acids
YER164W	Over expressed	CHD1	Chromatin organization modifier, Helicase, and DNA-binding domains
YBR274W	Over expressed	CHK1	CHeckpoint Kinase
YGR157W	Over expressed	CHO2	CHOline requiring
YPL241C	Over expressed	CIN2	Chromosome INstability
YPR120C	Over expressed	CLB5	CycLin B
YER157W	Over expressed	COG3	Conserved Oligomeric Golgi complex
YGL263W	Over expressed	COS12	COnserved Sequence
YML132W	Over expressed	COS3	COnserved Sequence
YHL048W	Over expressed	COS8	COnserved Sequence
YJL003W	Over expressed	COX16	Cytochrome c OXidase
YNL130C	Over expressed	CPT1	CholinePhosphoTransferase
YHR146W	Over expressed	CRP1	Cruciform DNA-Recognizing Protein

YDR179C	Over expressed	CSN9	Cop9 SigNalosome subunit
YPL018W	Over expressed	CTF19	Chromosome Transmission Fidelity
YML112W	Over expressed	CTK3	Carboxy-Terminal domain Kinase
YLR286C	Over expressed	CTS1	ChiTinaSe
YJL149W	Over expressed	DAS1	Dst1-delta 6-Azauracil Sensitivity
YGR092W	Over expressed	DBF2	DumbBell Former
YFR012W	Over expressed	DCV1	Demands Cdc28 kinase activity for Viability
YKL046C	Over expressed	DCW1	Defective Cell Wall
YNL335W	Over expressed	DDI3	DNA Damage Inducible
YOR163W	Over expressed	DDP1	Diadenosine and Diphosphoinositol Polyphosphate phosphohydrolase
YOR236W	Over expressed	DFR1	DihydroFolate Reductase
YPL266W	Over expressed	DIM1	DIMethylase
YDL178W	Over expressed	DLD2	D-Lactate Dehydrogenase
YEL071W	Over expressed	DLD3	D-Lactate Dehydrogenase
YMR162C	Over expressed	DNF3	Drs2 Neo1 Family
YDR141C	Over expressed	DOP1	homolog of A. nidulans DOPey
YPL107W	Over expressed	DPC25	Delta-Psi dependent mitochondrial import and Cleavage protein of ~25 kDa
YLR143W	Over expressed	DPH6	DiPHthamide biosynthesis
YGL196W	Over expressed	DSD1	0
YNL191W	Over expressed	DUG3	Deficient in Utilization of Glutathione
YDL101C	Over expressed	DUN1	DNA-damage UNinducible
YBR208C	Over expressed	DUR1,2	Degradation of URea
YMR128W	Over expressed	ECM16	ExtraCellular Mutant
YPL095C	Over expressed	EEB1	Ethyl Ester Biosynthesis
YAL003W	Over expressed	EFB1	Elongation Factor Beta
YHL039W	Over expressed	EFM1	Elongation Factor Methyltransferase
YHR193C	Over expressed	EGD2	Enhancer of Gal4 DNA binding
YDR147W	Over expressed	EKI1	Ethanolamine KInase
YPL046C	Over expressed	ELC1	ELongin C
YOR144C	Over expressed	ELG1	Enhanced Level of Genomic instability
YCR034W	Over expressed	ELO2	fatty acid ELOngation
YPL101W	Over expressed	ELP4	ELongator Protein
YCL045C	Over expressed	EMC1	ER Membrane protein Complex
YDR512C	Over expressed	EMI1	Early Meiotic Induction
YDR040C	Over expressed	ENA1	Exitus NAtru (Latin, "exit sodium")
YDR038C	Over expressed	ENA5	Exitus NAtru (Latin, "exit sodium")
YOR246C	Over expressed	ENV9	late ENdosome and Vacuole interface function
YGR060W	Over expressed	ERG25	ERGosterol biosynthesis
YMR134W	Over expressed	ERG29	ERGosterol biosynthesis

YFR041C	Over expressed	ERJ5	Endoplasmic Reticulum located J-protein
YAR002C-A	Over expressed	ERP1	Emp24p/Erv25p Related Protein
YHR110W	Over expressed	ERP5	Emp24p/Erv25p Related Protein
YMR219W	Over expressed	ESC1	Establishes Silent Chromatin
YBR026C	Over expressed	ETR1	2-Enoyl Thioester Reductase
YDR261C	Over expressed	EXG2	EXo-1,3-beta-Glucanase
YDL045C	Over expressed	FAD1	FAD synthetase
YER060W	Over expressed	FCY21	FluoroCYtosine resistance
YBR040W	Over expressed	FIG1	Factor-Induced Gene
YLR454W	Over expressed	FMP27	Found in Mitochondrial Proteome
YFL022C	Over expressed	FRS2	phenylalanyl (F)-tRNA Synthetase
YMR222C	Over expressed	FSH2	Family of Serine Hydrolases
YAL019W	Over expressed	FUN30	Function Unknown Now
YLR068W	Over expressed	FYV7	Function required for Yeast Viability
YCL011C	Over expressed	GBP2	G-strand Binding Protein
YNL062C	Over expressed	GCD10	General Control Derepressed
YDR283C	Over expressed	GCN2	General Control Nonderepressible
YMR189W	Over expressed	GCV2	GlyCine cleaVage
YAL062W	Over expressed	GDH3	Glutamate DeHydrogenase
YJL137C	Over expressed	GLG2	Glycogenin-Like Gene
YPR035W	Over expressed	GLN1	GLutamiNe metabolism
YML004C	Over expressed	GLO1	GLyOxalase
YEL046C	Over expressed	GLY1	GLYcine requiring
YGR256W	Over expressed	GND2	6-phosphoGlucoNateDehydrogenase
YDL021W	Over expressed	GPM2	Glycerate PhosphoMutase
YFL031W	Over expressed	HAC1	Homologous to Atf/Creb1
YER057C	Over expressed	HMF1	Homologous Mmf1p Factor
YBR034C	Over expressed	HMT1	HnRNP MethylTransferase
YMR032W	Over expressed	HOF1	Homolog Of cdc Fifteen
YJL159W	Over expressed	HSP150	Heat Shock Protein
YDR171W	Over expressed	HSP42	Heat Shock Protein
YDR224C	Over expressed	HTB1	Histone h Two B
YJR036C	Over expressed	HUL4	Hect Ubiquitin Ligase
YFL011W	Over expressed	HXT10	HeXose Transporter
YEL034W	Over expressed	HYP2	HYPusine-containing protein
YLR099C	Over expressed	ICT1	Increased Copper Tolerance
YLR174W	Over expressed	IDP2	Isocitrate Dehydrogenase, NADP-specific
YFR017C	Over expressed	IGD1	Inhibitor of Glycogen Debranching
YLR355C	Over expressed	ILV5	IsoLeucine-plus-Valine requiring

YBR107C	Over expressed	IML3	Increased Minichromosome Loss
YGR031W	Over expressed	IMO32	Intermediate cleaved by Mitochondrial Octapeptidyl aminopeptidase
YOR109W	Over expressed	INP53	INositol polyphosphate 5-Phosphatase
YFR013W	Over expressed	IOC3	Iswi One Complex
YBR011C	Over expressed	IPP1	Inorganic PyroPhosphatase
YPL242C	Over expressed	IQG1	IQGAP-related protein
YOR013W	Over expressed	IRC11	Increased Recombination Centers
YPR038W	Over expressed	IRC16	Increased Recombination Centers
YJL142C	Over expressed	IRC9	Increased Recombination Centers
YPR106W	Over expressed	ISR1	Inhibition of Staurosporine Resistance
YOR226C	Over expressed	ISU2	IscU homolog
YOL103W	Over expressed	ITR2	myo-Inositol TRansporter
YPL269W	Over expressed	KAR9	KARyogamy
YGL216W	Over expressed	KIP3	KInesin related Protein
YPR159W	Over expressed	KRE6	Killer toxin REsistant
YPL053C	Over expressed	KTR6	Kre Two Related
YGL079W	Over expressed	KXD1	KxDL homolog
YLR011W	Over expressed	LOT6	LOw Temperature-responsive
YDR503C	Over expressed	LPP1	Lipid Phosphate Phosphatase
YER112W	Over expressed	LSM4	Like SM
YDL131W	Over expressed	LYS21	LYSine
YDR234W	Over expressed	LYS4	LYSine requiring
YGL154C	Over expressed	LYS5	LYSine requiring
YJL013C	Over expressed	MAD3	Mitotic Arrest-Deficient
YBR299W	Over expressed	MAL32	MALtose
YLR244C	Over expressed	MAP1	Methionine AminoPeptidase
YLR163C	Over expressed	MAS1	Mitochondrial ASsembly
YOL119C	Over expressed	MCH4	MonoCarboxylate transporter Homolog
YOR221C	Over expressed	MCT1	Malonyl-CoA:ACP Transferase
YGR012W	Over expressed	MCY1	putative Mitochondrial CYsteine synthase
YJL112W	Over expressed	MDV1	Mitochondrial DiVision
YBR193C	Over expressed	MED8	MEDiator complex
YNL142W	Over expressed	MEP2	0
YGR264C	Over expressed	MES1	MEthionyl-tRNA Synthetase
YKL001C	Over expressed	MET14	METhionine requiring
YIL046W	Over expressed	MET30	METhionine requiring
YKL195W	Over expressed	MIA40	Mitochondrial intermembrane space Import and Assembly
YKR016W	Over expressed	MIC60	MItochondrial contact site and Cristae organizing system
YLR332W	Over expressed	MID2	Mating pheromone-Induced Death

YPR188C	Over expressed	MLC2	Myo1p Light Chain
YJR131W	Over expressed	MNS1	0
YPL082C	Over expressed	MOT1	Modifier of Transcription
YNL249C	Over expressed	MPA43	Multicopy PDC1 Activator
YOR201C	Over expressed	MRM1	Mitochondrial rRNA Methyltransferase
YDL045W-A	Over expressed	MRP10	Mitochondrial Ribosomal Protein
YDL202W	Over expressed	MRPL11	Mitochondrial Ribosomal Protein, Large subunit
YDR462W	Over expressed	MRPL28	Mitochondrial Ribosomal Protein, Large subunit
YPL013C	Over expressed	MRPS16	Mitochondrial Ribosomal Protein, Small subunit
YNL306W	Over expressed	MRPS18	Mitochondrial Ribosomal Protein, Small subunit
YKR077W	Over expressed	MSA2	Mbf and Sbf Associated
YPR047W	Over expressed	MSF1	Mitochondrial aminoacyl-tRNA Synthetase, Phenylalanine (F)
YDL154W	Over expressed	MSH5	MutS Homolog
YGR028W	Over expressed	MSP1	Mitochondrial Sorting of Proteins
YDL107W	Over expressed	MSS2	Mitochondrial Splicing
YDR493W	Over expressed	MZM1	Mitochondrial Zinc Maintenance
YGL211W	Over expressed	NCS6	Needs Cla4 to Survive
YLR194C	Over expressed	NCW2	Novel Cell Wall protein
YDL085W	Over expressed	NDE2	NADH Dehydrogenase, External
YPL226W	Over expressed	NEW1	Nu+
YBR089C-A	Over expressed	NHP6B	Non-Histone Protein
YFR002W	Over expressed	NIC96	Nucleoporin-Interacting Component of 96 kDa
YPR144C	Over expressed	NOC4	NucleOlar Complex associated
YHR072W-A	Over expressed	NOP10	NucleOlar Protein
YNL061W	Over expressed	NOP2	NucleOlar Protein
YPL146C	Over expressed	NOP53	NucleOlar Protein
YDR043C	Over expressed	NRG1	Negative Regulator of Glucose-repressed genes
YDL167C	Over expressed	NRP1	N (asparagine)-Rich Protein
YML103C	Over expressed	NUP188	NUclear Pore
YDL116W	Over expressed	NUP84	NUclear Pore
YGL151W	Over expressed	NUT1	Negative regulation of URS Two
YHL020C	Over expressed	OPI1	OverProducer of Inositol
YPR162C	Over expressed	ORC4	Origin Recognition Complex
YJL002C	Over expressed	OST1	OligoSaccharylTransferase
YLR054C	Over expressed	OSW2	Outer Spore Wall
YDR071C	Over expressed	PAA1	PolyAmine Acetyltransferase
YDR348C	Over expressed	PAL1	Pears And Lemons
YIL145C	Over expressed	PAN6	PANtothenate biosynthesis
YHL046C	Over expressed	PAU13	seriPAUperin

YGR178C	Over expressed	PBP1	Pab1p-Binding Protein
YPL058C	Over expressed	PDR12	Pleiotropic Drug Resistance
YOR153W	Over expressed	PDR5	Pleiotropic Drug Resistance
YGR193C	Over expressed	PDX1	Pyruvate Dehydrogenase complex protein X
YPL154C	Over expressed	PEP4	carboxyPEPtidase Y-deficient
YMR231W	Over expressed	PEP5	carboxyPEPtidase Y-deficient
YMR257C	Over expressed	PET111	PETite colonies
YER153C	Over expressed	PET122	PETite colonies
YHR160C	Over expressed	PEX18	PEroXin
YPL112C	Over expressed	PEX25	PEroXisome related
YOR193W	Over expressed	PEX27	PEroXisome related
YLR324W	Over expressed	PEX30	PEroXisome related
YNL329C	Over expressed	PEX6	PEroXin
YBR074W	Over expressed	PFF1	Protease in FXNA-related Family
YHR185C	Over expressed	PFS1	Prospore Formation at Spindles
YJR153W	Over expressed	PGU1	PolyGalactUronase
YOL001W	Over expressed	PHO80	PHOsphate metabolism
YGR233C	Over expressed	PHO81	PHOsphate metabolism
YGL023C	Over expressed	PIB2	PhosphatidylInositol(3)-phosphate Binding
YPR113W	Over expressed	PIS1	Phosphatidyl Inositol Synthase
YAL023C	Over expressed	PMT2	Protein O-MannosylTransferase
YOR266W	Over expressed	PNT1	PeNTamidine resistance
YCR014C	Over expressed	POL4	POLymerase
YBR257W	Over expressed	POP4	Processing Of Precursor RNAs
YAL033W	Over expressed	POP5	Processing Of Precursor RNAs
YIL114C	Over expressed	POR2	PORin
YJL203W	Over expressed	PRP21	Pre-mRNA Processing
YOR361C	Over expressed	PRT1	PRoTein synthesis
YDL006W	Over expressed	PTC1	Phosphatase type Two C
YGR156W	Over expressed	PTI1	PTa1p Interacting protein
YLR142W	Over expressed	PUT1	Proline UTilization
YKR090W	Over expressed	PXL1	PaXillin-Like protein
YIL121W	Over expressed	QDR2	QuiniDine Resistance
YPL022W	Over expressed	RAD1	RADiation sensitive
YML011C	Over expressed	RAD33	RADiation sensitive
YDL059C	Over expressed	RAD59	RADiation sensitive
YNL216W	Over expressed	RAP1	Repressor/Activator site binding Protein
YOR101W	Over expressed	RAS1	homologous to RAS proto-oncogene
YLR084C	Over expressed	RAX2	0

YDR527W	Over expressed	RBA50	RNA polymerase II (B) Associated protein
YIL139C	Over expressed	REV7	REVersionless
YBL020W	Over expressed	RFT1	Requiring Fifty-Three
YNL294C	Over expressed	RIM21	Regulator of IME2
YGL045W	Over expressed	RIM8	Regulator of IME2
YEL050C	Over expressed	RML2	Ribosomal Mitochondrial Large
YGL044C	Over expressed	RNA15	poly(A) mRNA metabolism
YGR180C	Over expressed	RNR4	RiboNucleotide Reductase
YPL123C	Over expressed	RNY1	RiboNuclease from Yeast
YGR085C	Over expressed	RPL11B	Ribosomal Protein of the Large subunit
YBR084C-A	Over expressed	RPL19A	Ribosomal Protein of the Large subunit
YPL220W	Over expressed	RPL1A	Ribosomal Protein of the Large subunit
YMR242C	Over expressed	RPL20A	Ribosomal Protein of the Large subunit
YOR312C	Over expressed	RPL20B	Ribosomal Protein of the Large subunit
YBR191W	Over expressed	RPL21A	Ribosomal Protein of the Large subunit
YOL127W	Over expressed	RPL25	Ribosomal Protein of the Large subunit
YDR471W	Over expressed	RPL27B	Ribosomal Protein of the Large subunit
YDL191W	Over expressed	RPL35A	Ribosomal Protein of the Large subunit
YDL136W	Over expressed	RPL35B	Ribosomal Protein of the Large subunit
YMR194W	Over expressed	RPL36A	Ribosomal Protein of the Large subunit
YLR325C	Over expressed	RPL38	Ribosomal Protein of the Large subunit
YDR012W	Over expressed	RPL4B	Ribosomal Protein of the Large subunit
YGL076C	Over expressed	RPL7A	Ribosomal Protein of the Large subunit
YPR187W	Over expressed	RPO26	RNA POlymerase
YDL130W	Over expressed	RPP1B	Ribosomal Protein P1 Beta
YMR230W	Over expressed	RPS10B	Ribosomal Protein of the Small subunit
YCR031C	Over expressed	RPS14A	Ribosomal Protein of the Small subunit
YDL083C	Over expressed	RPS16B	Ribosomal Protein of the Small subunit
YDR450W	Over expressed	RPS18A	Ribosomal Protein of the Small subunit
YML026C	Over expressed	RPS18B	Ribosomal Protein of the Small subunit
YNL302C	Over expressed	RPS19B	Ribosomal Protein of the Small subunit
YLR441C	Over expressed	RPS1A	Ribosomal Protein of the Small subunit
YJL190C	Over expressed	RPS22A	Ribosomal Protein of the Small subunit
YPR132W	Over expressed	RPS23B	Ribosomal Protein of the Small subunit
YGL189C	Over expressed	RPS26A	Ribosomal Protein of the Small subunit
YHR021C	Over expressed	RPS27B	Ribosomal Protein of the Small subunit
YLR264W	Over expressed	RPS28B	Ribosomal Protein of the Small subunit
YJR123W	Over expressed	RPS5	Ribosomal Protein of the Small subunit
YBR181C	Over expressed	RPS6B	Ribosomal Protein of the Small subunit

YPL081W	Over expressed	RPS9A	Ribosomal Protein of the Small subunit
YOR117W	Over expressed	RPT5	Regulatory Particle Triple-A protein, or Regulatory Particle Triphosphatase
YHR069C	Over expressed	RRP4	Ribosomal RNA Processing
YBR246W	Over expressed	RRT2	Regulator of rDNA Transcription
YNR037C	Over expressed	RSM19	Ribosomal Small subunit of Mitochondria
YOL138C	Over expressed	RTC1	Restriction of Telomere Capping
YOR077W	Over expressed	RTS2	0
YFR005C	Over expressed	SAD1	SnRNP Assembly Defective
YLR180W	Over expressed	SAM1	S-AdenosylMethionine requiring
YPL274W	Over expressed	SAM3	S-AdenosylMethionine metabolism
YPL218W	Over expressed	SAR1	Secretion-Associated, Ras-related
YOR213C	Over expressed	SAS5	Something About Silencing
YDR351W	Over expressed	SBE2	Suppressor of BEm4
YGL011C	Over expressed	SCL1	Suppressor of Crl3 ts Lethality
YDL139C	Over expressed	SCM3	Suppressor of Chromosome Missegregation
YBR037C	Over expressed	SCO1	Suppressor of Cytochrome Oxidase deficiency
YBL091C-A	Over expressed	SCS22	Suppressor of Choline Sensitivity
YGR245C	Over expressed	SDA1	Severe Depolymerization of Actin
YDR511W	Over expressed	SDH7	Succinate DeHydrogenase
YIL167W	Over expressed	SDL1	0
YBL104C	Over expressed	SEA4	SEh1-Associated
YPR181C	Over expressed	SEC23	SECretory
YDR238C	Over expressed	SEC26	SECretory
YGL137W	Over expressed	SEC27	SECretory
YER081W	Over expressed	SER3	SERine requiring
YPR198W	Over expressed	SGE1	Suppression of Gal11 Expression
YDR393W	Over expressed	SHE9	Sensitivity to High Expression
YOL004W	Over expressed	SIN3	Switch INdependent
YDR409W	Over expressed	SIZ1	SAP and mIZ-finger domain
YOR076C	Over expressed	SKI7	SuperKIller
YBL007C	Over expressed	SLA1	Synthetic Lethal with ABP1
YLR139C	Over expressed	SLS1	Synthetic Lethal with SSM4
YOL034W	Over expressed	SMC5	Structural Maintenance of Chromosomes
YGR074W	Over expressed	SMD1	snRNA Sm binding site protein D1
YPR054W	Over expressed	SMK1	0
YBR182C	Over expressed	SMP1	Second MEF2-like Protein 1
YPR182W	Over expressed	SMX3	0
YDR477W	Over expressed	SNF1	Sucrose NonFermenting
YPL002C	Over expressed	SNF8	Sucrose NonFermenting

YFL059W	Over expressed	SNZ3	SNooZe
YHR163W	Over expressed	SOL3	Suppressor Of Los1-1
YJR010C-A	Over expressed	SPC1	Signal Peptidase Complex
YGL093W	Over expressed	SPC105	Spindle Pole Component
YER018C	Over expressed	SPC25	Spindle Pole Component
YHR172W	Over expressed	SPC97	Spindle Pole Component
YPR069C	Over expressed	SPE3	SPErmidine auxotroph
YPR133C	Over expressed	SPN1	Suppresses Postrecruitment functions gene Number 1
YKR031C	Over expressed	SPO14	SPOrulation
YIL073C	Over expressed	SPO22	SPOrulation
YBR152W	Over expressed	SPP381	Suppressor of PrP38-1
YLR424W	Over expressed	SPP382	Suppressor of PrP38 #2
YDR523C	Over expressed	SPS1	SPorulation Specific
YNL224C	Over expressed	SQS1	SQuelch of Splicing suppression
YDR308C	Over expressed	SRB7	Suppressor of RNA polymerase B
YNL189W	Over expressed	SRP1	Suppressor of Rna Polymerase I
YNL209W	Over expressed	SSB2	Stress-Seventy subfamily B
YMR183C	Over expressed	SSO2	Supressor of Sec One
YJL156C	Over expressed	SSY5	Sulfonylurea Sensitive on YPD
YJR086W	Over expressed	STE18	STErile
YDR103W	Over expressed	STE5	STErile
YMR054W	Over expressed	STV1	Similar To VPH1
YLR251W	Over expressed	SYM1	Stress-inducible Yeast Mpv17
YBR261C	Over expressed	TAE1	Translation Associated Element
YPR048W	Over expressed	TAH18	Top1T722A mutant Hypersensitive
YMR028W	Over expressed	TAP42	Two A phosphatase Associated Protein
YJR009C	Over expressed	TDH2	Triose-phosphate DeHydrogenase
YGR192C	Over expressed	TDH3	Triose-phosphate DeHydrogenase
YIL039W	Over expressed	TED1	Trafficking of Emp24p/Erv25p-dependent cargo Disrupted
YML064C	Over expressed	TEM1	TErmination of M phase
YDR311W	Over expressed	TFB1	Transcription Factor B
YPR056W	Over expressed	TFB4	Transcription Factor B subunit 4
YAL001C	Over expressed	TFC3	Transcription Factor class C
YMR313C	Over expressed	TGL3	TriacylGlycerol Lipase
YKR059W	Over expressed	TIF1	Translation Initiation Factor
YGR181W	Over expressed	TIM13	Translocase of the Inner Mitochondrial membrane
YJL143W	Over expressed	TIM17	Translocase of the Inner Mitochondrial membrane
YPR040W	Over expressed	TIP41	Tap42 Interacting Protein
YER011W	Over expressed	TIR1	TIp1-Related

YDR117C	Over expressed	TMA64	Translation Machinery Associated
YLR262C-A	Over expressed	TMA7	Translation Machinery Associated
YOL102C	Over expressed	TPT1	tRNA 2'-PhosphoTransferase
YKR056W	Over expressed	TRM2	tRNA Methyltransferase
YPL030W	Over expressed	TRM44	TRna Methyltransferase
YHR070W	Over expressed	TRM5	tRNA Methyltransferase
YBR265W	Over expressed	TSC10	Temperature-sensitive Suppressors of Csg2 mutants
YJR136C	Over expressed	TTI2	Two Tel2-Interacting protein
YMR071C	Over expressed	TVP18	Tlg2-Vesicle Protein
YML013W	Over expressed	UBX2	UBiquitin regulatory X
YMR067C	Over expressed	UBX4	UBiquitin regulatory X
YMR271C	Over expressed	URA10	URAcil requiring
YBL039C	Over expressed	URA7	URAcil requiring
YML029W	Over expressed	USA1	U1-Snp1 Associating
YEL040W	Over expressed	UTR2	Unidentified TRanscript
YEL013W	Over expressed	VAC8	VACuole related
YDL077C	Over expressed	VAM6	VAcuolar Morphogenesis
YHL035C	Over expressed	VMR1	Vacuolar Multidrug Resistance
YPL065W	Over expressed	VPS28	Vacuolar Protein Sorting
YDR484W	Over expressed	VPS52	Vacuolar Protein Sorting
YHL028W	Over expressed	WSC4	cell Wall integrity and Stress response Component
YER123W	Over expressed	YCK3	Yeast Casein Kinase
YBL060W	Over expressed	YEL1	Yeast EFA6-Like
YER041W	Over expressed	YEN1	0
YHR048W	Over expressed	YHK8	0
YNL263C	Over expressed	YIF1	YIP1-Interacting Factor
YPR125W	Over expressed	YLH47	Yeast LETM1 Homolog of 47 kD
YPR024W	Over expressed	YME1	Yeast Mitochondrial Escape
YPR028W	Over expressed	YOP1	YIP One Partner
YML027W	Over expressed	YOX1	Yeast homeobOX
YHR105W	Over expressed	YPT35	0
YER190W	Over expressed	YRF1-2	0
YGR296W	Over expressed	YRF1-3	0
YLR466W	Over expressed	YRF1-4	0
YNL339C	Over expressed	YRF1-6	0
YPL283C	Over expressed	YRF1-7	0
YOR396W	Over expressed	YRF1-8	0
YPR107C	Over expressed	YTH1	Yeast THirty kDa Homolog
YBL111C	Over expressed		

YBR016W	Over expressed		
YCL020W	Over expressed		
YDL180W	Over expressed		
YDR239C	Over expressed		
YDR476C	Over expressed		
YGR109W-A	Over expressed		
YGR130C	Over expressed		
YGR266W	Over expressed		
YIL055C	Over expressed		
YIL082W-A	Over expressed		
YIL177C	Over expressed		
YJL068C	Over expressed		
YJL113W	Over expressed		
YJL213W	Over expressed		
YJL225C	Over expressed		
YJR084W	Over expressed		
YKL050C	Over expressed		
YKL063C	Over expressed		
YKL075C	Over expressed		
YLL058W	Over expressed		
YLR177W	Over expressed		
YLR179C	Over expressed		
YLR419W	Over expressed		
YML133C	Over expressed		
YMR084W	Over expressed		
YMR085W	Over expressed		
YMR226C	Over expressed		
YMR310C	Over expressed		
YNL046W	Over expressed		
YNL095C	Over expressed		
YNL296W	Over expressed		
YNR068C	Over expressed		
YPL068C	Over expressed		
YPR027C	Over expressed		
YPR063C	Over expressed		
YPR071W	Over expressed		
YPR097W	Over expressed		
YPR147C	Over expressed		
YPR148C	Over expressed		

YPR174C	Over expressed		
ARA11	Over expressed		
ARA15	Over expressed		
ARA17	Over expressed		
ARA19	Over expressed		
ARA20	Over expressed		
ARA21	Over expressed		
ARA22	Over expressed		
ARA24	Over expressed		
ARA27	Over expressed		
ARA29	Over expressed		
ARA8	Over expressed		
ARA9	Over expressed		
YAL004W	Over expressed		
YAL045C	Over expressed		
YAL064W	Over expressed		
YAR030C	Over expressed		
YAR053W	Over expressed		
YBL044W	Over expressed		
YBL048W	Over expressed		
YBR051W	Over expressed		
YBR118W-R	Over expressed		
YBR157C	Over expressed		
YBR226C	Over expressed		
YCL022C	Over expressed		
YCL023C	Over expressed		
YCR024C-A-R	Over expressed		
YCR085W	Over expressed		
YDL055C-R	Over expressed		
YDL185W-R	Over expressed		
YDL186W	Over expressed		
YDL242W	Over expressed		
YDR008C	Over expressed		
YDR048C	Over expressed		
YDR274C	Over expressed		
YEL045C	Over expressed		
YEL074W	Over expressed		
YEL076C	Over expressed		
YER091C-R	Over expressed		

YER121W	Over expressed		
YGR242W	Over expressed		
YGR272C	Over expressed		
YIL024C	Over expressed		
YIL058W	Over expressed		
YJR012C	Over expressed		
YJR071W	Over expressed		
YKL162C-A	Over expressed		
YKR059W-R	Over expressed		
YLR101C	Over expressed		
YLR123C	Over expressed		
YLR156W	Over expressed		
YLR161W	Over expressed		
YLR282C	Over expressed		
YLR444C	Over expressed		
YLR464W	Over expressed		
YML117W-A	Over expressed		
YMR193C-A	Over expressed		
YNL011C	Over expressed		
YNL109W	Over expressed		
YNL179C	Over expressed		
YNL193W	Over expressed		
YNL244C-R	Over expressed		
YOR263C	Over expressed		
YOR387C	Over expressed		
YPL165C	Over expressed		
YPL197C	Over expressed		
YPL238C	Over expressed		
YPR039W	Over expressed		
YPR059C	Over expressed		
YPR064W	Over expressed		
YPR090W	Over expressed		
YPL267W	Down expressed	ACM1	APC/C[Cdh1] Modulator
YDR448W	Down expressed	ADA2	transcriptional ADAptor
YEL052W	Down expressed	AFG1	ATPase Family Gene
YDR524C	Down expressed	AGE1	Arf Gap with Effector function(s)
YNR074C	Down expressed	AIF1	Apoptosis-Inducing Factor
YJR100C	Down expressed	AIM25	Altered Inheritance of Mitochondria

YOR175C	Down expressed	ALE1	Acyltransferase for Lyso-phosphatidylEthanolamine
YBR070C	Down expressed	ALG14	Asparagine Linked Glycosylation
YBR243C	Down expressed	ALG7	Asparagine-Linked Glycosylation
YOL130W	Down expressed	ALR1	ALuminum Resistance
YBR151W	Down expressed	APD1	Actin Patches Distal
YKL157W	Down expressed	APE2	AminoPEptidase
YDR376W	Down expressed	ARH1	Adrenodoxin Reductase Homolog
YDR380W	Down expressed	ARO10	AROmatic amino acid requiring
YDR184C	Down expressed	ATC1	Aip Three Complex
YCL038C	Down expressed	ATG22	AuTophaGy related
YLR431C	Down expressed	ATG23	AuTophaGy related
YNL223W	Down expressed	ATG4	AuTophaGy related
YBL099W	Down expressed	ATP1	ATP synthase
YDR298C	Down expressed	ATP5	ATP synthase
YKL016C	Down expressed	ATP7	ATP synthase
YKL146W	Down expressed	AVT3	Amino acid Vacuolar Transport
YER119C	Down expressed	AVT6	Amino acid Vacuolar Transport
YNL039W	Down expressed	BDP1	B Double Prime
Q0120	Down expressed	BI4	0
YOR304C-A	Down expressed	BIL1	Bud6-Interacting Ligand
YFR047C	Down expressed	BNA6	Biosynthesis of Nicotinic Acid
YER114C	Down expressed	BOI2	Bem1 (One) Interacting protein
YLR015W	Down expressed	BRE2	BREfeldin A sensitivity
YDR252W	Down expressed	BTT1	BTf Three
YNL305C	Down expressed	BXI1	BaX Inhibitor
YIL083C	Down expressed	CAB2	Coenzyme A Biosynthesis
YDL069C	Down expressed	CBS1	Cytochrome B Synthesis
YDL220C	Down expressed	CDC13	Cell Division Cycle
YAR019C	Down expressed	CDC15	Cell Division Cycle
YGL116W	Down expressed	CDC20	Cell Division Cycle
YOR257W	Down expressed	CDC31	Cell Division Cycle
YOL139C	Down expressed	CDC33	Cell Division Cycle
YDR168W	Down expressed	CDC37	Cell Division Cycle
YLR229C	Down expressed	CDC42	Cell Division Cycle
YDL017W	Down expressed	CDC7	Cell Division Cycle
YDR301W	Down expressed	CFT1	Cleavage Factor Two
YDR267C	Down expressed	CIA1	Cytosolic Iron-sulfur protein Assembly
YOR028C	Down expressed	CIN5	Chromosome INstability
YNL298W	Down expressed	CLA4	CLn Activity dependant

YGR110W	Down expressed	CLD1	CardioLipin-specific Deacylase
YPL256C	Down expressed	CLN2	CycLiN
YOR250C	Down expressed	CLP1	CLeavage/Polyadenylation factor Ia subunit
YLR271W	Down expressed	CMG1	Cytoplasmic and Mitochondrial G-patch protein 1
YOR093C	Down expressed	CMR2	Changed Mutation Rate
YGL005C	Down expressed	COG7	Conserved Oligomeric Golgi complex
YOL008W	Down expressed	COQ10	COenzyme Q
YBR203W	Down expressed	COS111	Ciclopirox Olamine Sensitive
YFL062W	Down expressed	COS4	COnserved Sequence
Q0045	Down expressed	COX1	Cytochrome c OXidase
YLR038C	Down expressed	COX12	Cytochrome c OXidase
Q0275	Down expressed	COX3	Cytochrome c OXidase
YMR256C	Down expressed	COX7	Cytochrome c OXidase
YDR304C	Down expressed	CPR5	Cyclosporin-sensitive Proline Rotamase
YLR216C	Down expressed	CPR6	Cyclosporin-sensitive Proline Rotamase
YJL172W	Down expressed	CPS1	CarboxyPeptidase yscS
YLR429W	Down expressed	CRN1	CoRoNin
YLR380W	Down expressed	CSR1	Chs5 Spa2 Rescue
YPR030W	Down expressed	CSR2	Chs5 Spa2 Rescue
YIL169C	Down expressed	CSS1	Condition Specific Secretion
YIL036W	Down expressed	CST6	Chromosome STability
YDR256C	Down expressed	CTA1	CaTalase A
YMR180C	Down expressed	CTL1	Capping enzyme mRNA Triphosphatase-Like
YHR053C	Down expressed	CUP1-1	Cu, copper, CUPrum
YHR055C	Down expressed	CUP1-2	Cu, copper, CUPrum
YDL209C	Down expressed	CWC2	Complexed With Cef1p
YCR017C	Down expressed	CWH43	Calcofluor White Hypersensitive
YJR151C	Down expressed	DAN4	Delayed ANaerobic
YPR111W	Down expressed	DBF20	DumbBell Forming
YDL031W	Down expressed	DBP10	Dead Box Protein
YOR046C	Down expressed	DBP5	Dead Box Protein
YLR128W	Down expressed	DCN1	Defective in Cullin Neddylation
YLR270W	Down expressed	DCS1	DeCapping Scavenger
YMR173W	Down expressed	DDR48	DNA Damage Responsive
YKL121W	Down expressed	DGR2	2-Deoxy-Glucose Resistant 2
YKL078W	Down expressed	DHR2	DEAH-box RNA helicase
YDL024C	Down expressed	DIA3	Digs Into Agar
YLR437C	Down expressed	DIF1	Damage-regulated Import Facilitator
YDR402C	Down expressed	DIT2	DITyrosine

YMR211W	Down expressed	DML1	Drosophila melanogaster Misato-Like protein
YOR005C	Down expressed	DNL4	DNA Ligase
YDR273W	Down expressed	DON1	DONut
YER088C	Down expressed	DOT6	Disruptor Of Telomeric silencing
YOR264W	Down expressed	DSE3	Daughter Specific Expression
YEL018W	Down expressed	EAF5	Esa1p-Associated Factor
YBR176W	Down expressed	ECM31	ExtraCellular Mutant
YBR078W	Down expressed	ECM33	ExtraCellular Mutant
YKR076W	Down expressed	ECM4	ExtraCellular Mutant
YKR004C	Down expressed	ECM9	ExtraCellular Mutant
YNL230C	Down expressed	ELA1	ELongin A
YKL048C	Down expressed	ELM1	ELongated Morphology
YLR186W	Down expressed	EMG1	Essential for Mitotic Growth
YDR516C	Down expressed	EMI2	Early Meiotic Induction
YDR153C	Down expressed	ENT5	Epsin N-Terminal homology
YGL001C	Down expressed	ERG26	ERGosterol biosynthesis
YER044C	Down expressed	ERG28	ERGosterol biosynthesis
YOR393W	Down expressed	ERR1	Enolase-Related Repeat
YOL017W	Down expressed	ESC8	Establishes Silent Chromatin
YNR054C	Down expressed	ESF2	Eighteen S rRNA Factor 2
YOR317W	Down expressed	FAA1	Fatty Acid Activation
YKL182W	Down expressed	FAS1	Fatty Acid Synthetase
YPR062W	Down expressed	FCY1	FluoroCYtosine resistance
YOR382W	Down expressed	FIT2	Facilitator of Iron Transport
YLR342W	Down expressed	FKS1	FK506 Sensitivity
YDR070C	Down expressed	FMP16	Found in Mitochondrial Proteome
YDL222C	Down expressed	FMP45	Found in Mitochondrial Proteome
YKR049C	Down expressed	FMP46	Found in Mitochondrial Proteome
YLL043W	Down expressed	FPS1	fdp1 Suppressor
YLR060W	Down expressed	FRS1	phenylalanyl (F)-tRNA Synthetase
YAL008W	Down expressed	FUN14	Function Unknown Now
YCL058C	Down expressed	FYV5	Function required for Yeast Viability
YGR196C	Down expressed	FYV8	Function required for Yeast Viability
YLR459W	Down expressed	GAB1	GPI and Actin Bar
YDR009W	Down expressed	GAL3	GALactose metabolism
YHR089C	Down expressed	GAR1	Glycine Arginine Rich
YLR013W	Down expressed	GAT3	0
YIR013C	Down expressed	GAT4	0
YMR311C	Down expressed	GLC8	GLyCogen

YDR272W	Down expressed	GLO2	GLyOxalase
YJL184W	Down expressed	GON7	0
YNL274C	Down expressed	GOR1	GlyOxylate Reductase
YER020W	Down expressed	GPA2	G Protein Alpha subunit
YPR160W	Down expressed	GPH1	Glycogen PHosphorylase
YDR302W	Down expressed	GPI11	GlycosylPhosphatidylInositol anchor biosynthesis
YOR262W	Down expressed	GPN2	Gly-Pro-Asn (N) motif
YER062C	Down expressed	GPP2	Glycerol-3-Phosphate Phosphatase
YLL035W	Down expressed	GRC3	0
YDR513W	Down expressed	GRX2	GlutaRedoXin
YOR185C	Down expressed	GSP2	Genetic Suppressor of Prp20-1
YJL110C	Down expressed	GZF3	Gata Zinc Finger protein
YDR295C	Down expressed	HDA2	Histone DeAcetylase
YNL014W	Down expressed	HEF3	Homolog of EF-3
YKR017C	Down expressed	HEL1	Histone E3 Ligase
YNL031C	Down expressed	HHT2	Histone H Three
YDR420W	Down expressed	HKR1	Hansenula mrakii Killer toxin Resistant
YMR161W	Down expressed	HLJ1	HomoLogous to E. coli dnaJ protein
YLR450W	Down expressed	HMG2	3-Hydroxy-3-MethylGlutaryl-coenzyme a reductase
YOR032C	Down expressed	HMS1	High-copy Mep Suppressor
YGL077C	Down expressed	HNM1	Hyper-resistance to Nitrogen Mustard
YMR251W-A	Down expressed	HOR7	HyperOsmolarity-Responsive
YMR172W	Down expressed	HOT1	High-Osmolarity-induced Transcription
YIL110W	Down expressed	HPM1	Histidine Protein Methyltransferase
YDR138W	Down expressed	HPR1	HyPerRecombination
YLR097C	Down expressed	HRT3	High level expression Reduces Ty3 transposition
YGL073W	Down expressed	HSF1	Heat Shock transcription Factor
YBR072W	Down expressed	HSP26	Heat Shock Protein
YCR021C	Down expressed	HSP30	Heat Shock Protein
YDR258C	Down expressed	HSP78	Heat Shock Protein
YDR225W	Down expressed	HTA1	Histone h Two A
YFR053C	Down expressed	HXK1	HeXoKinase
YEL069C	Down expressed	HXT13	HeXose Transporter
YDR345C	Down expressed	HXT3	HeXose Transporter
YHR092C	Down expressed	HXT4	HeXose Transporter
YDR343C	Down expressed	HXT6	HeXose Transporter
YDR342C	Down expressed	HXT7	HeXose Transporter
YMR195W	Down expressed	ICY1	Interacting with the CYtoskeleton
YIL172C	Down expressed	IMA3	IsoMAltase

YKR019C	Down expressed	IRS4	Increased rDNA Silencing
YLR023C	Down expressed	IZH3	Implicated in Zinc Homeostasis
YPR169W	Down expressed	JIP5	Jumonji domain Interacting Protein
YJR091C	Down expressed	JSN1	Just Say No
YER110C	Down expressed	KAP123	KAryoPherin
YCL055W	Down expressed	KAR4	KARyogamy
YGR238C	Down expressed	KEL2	KELch repeat
YDL108W	Down expressed	KIN28	protein KINase
YLL019C	Down expressed	KNS1	Kinase Next to SPA2
YKR061W	Down expressed	KTR2	Kre Two Related
YDR499W	Down expressed	LCD1	Lethal, Checkpoint-defective, DNA damage sensitive
YDL146W	Down expressed	LDB17	Low Dye Binding
YLL007C	Down expressed	LMO1	eLMO homolog
YJL038C	Down expressed	LOH1	Loss Of Heterozygosity
YHR081W	Down expressed	LRP1	Like RrP6
YFR024C-A	Down expressed	LSB3	Las Seventeen Binding protein
YBR115C	Down expressed	LYS2	LYSine requiring
YOR197W	Down expressed	MCA1	MetaCAspase
YKL221W	Down expressed	MCH2	MonoCarboxylate transporter Homolog
YOL126C	Down expressed	MDH2	Malate DeHydrogenase
YPR083W	Down expressed	MDM36	Mitochondrial Distribution and Morphology
YJL102W	Down expressed	MEF2	Mitochondrial Elongation Factor
YER044C-A	Down expressed	MEI4	MEIosis-specific
YDR296W	Down expressed	MHR1	Mitochondrial Homologous Recombination
YLR035C	Down expressed	MLH2	MutL Homolog
YKL044W	Down expressed	MMO1	Mini Mitochondria ORF
YKL064W	Down expressed	MNR2	MaNganese Resistance
YGR243W	Down expressed	MPC3	Mitochondrial Pyruvate Carrier
YPR166C	Down expressed	MRP2	Mitochondrial Ribosomal Protein
YKL167C	Down expressed	MRP49	Mitochondrial Ribosomal Protein
YNL185C	Down expressed	MRPL19	Mitochondrial Ribosomal Protein, Large subunit
YKR085C	Down expressed	MRPL20	Mitochondrial Ribosomal Protein, Large subunit
YNL177C	Down expressed	MRPL22	Mitochondrial Ribosomal Protein, Large subunit
YMR024W	Down expressed	MRPL3	Mitochondrial Ribosomal Protein, Large subunit
YKL138C	Down expressed	MRPL31	Mitochondrial Ribosomal Protein, Large subunit
YKL170W	Down expressed	MRPL38	Mitochondrial Ribosomal Protein, Large subunit
YDR237W	Down expressed	MRPL7	Mitochondrial Ribosomal Protein, Large subunit
YDR337W	Down expressed	MRPS28	Mitochondrial Ribosomal Protein, Small subunit
YJR003C	Down expressed	MRX12	Mitochondrial oRganization of gene eXpression (MIOREX)

YML128C	Down expressed	MSC1	Meiotic Sister-Chromatid recombination
YHR120W	Down expressed	MSH1	MutS Homolog
YMR037C	Down expressed	MSN2	Multicopy suppressor of SNF1 mutation
YDR335W	Down expressed	MSN5	Multicopy suppressor of SNf1 mutation
YKR080W	Down expressed	MTD1	Methylene Tetrahydrafolate Dehydrogenase
YOR160W	Down expressed	MTR10	Mrna TRansport defective
YKL186C	Down expressed	MTR2	Mrna TRansport
YPR149W	Down expressed	NCE102	NonClassical Export
YPL006W	Down expressed	NCR1	Niemann-pick type C Related
YMR145C	Down expressed	NDE1	NADH Dehydrogenase, External
YOL104C	Down expressed	NDJ1	NonDisJunction
YLR265C	Down expressed	NEJ1	Nonhomologous End-Joining defective
YOR156C	Down expressed	NFI1	Neck Filament Interacting
YMR309C	Down expressed	NIP1	Nuclear ImPort
YLR195C	Down expressed	NMT1	N-Myristoyl Transferase
YJR112W	Down expressed	NNF1	Necessary for Nuclear Function
YNL200C	Down expressed	NNR1	Nicotinamide Nucleotide Repair
YKL151C	Down expressed	NNR2	Nicotinamide Nucleotide Repair
YNR053C	Down expressed	NOG2	NucleOlar G-protein
YJL010C	Down expressed	NOP9	NucleOlar Protein
YEL062W	Down expressed	NPR2	Nitrogen Permease Regulator
YOR071C	Down expressed	NRT1	Nicotinamide Riboside Transporter
YNL091W	Down expressed	NST1	Negatively affects Salt Tolerance
YPR031W	Down expressed	NTO1	NuA Three Orf
YDR179W-A	Down expressed	NVJ3	Nucleus-Vacuole Junction
YML060W	Down expressed	OGG1	8-OxoGuanine Glycosylase/lyase
YOL032W	Down expressed	OPI10	OverProducer of Inositol
YOR130C	Down expressed	ORT1	ORnithine Transporter
YHL013C	Down expressed	OTU2	member of the Ovarian TUmor family
YPL171C	Down expressed	OYE3	Old Yellow Enzyme
YMR325W	Down expressed	PAU19	seriPAUperin
YAL068C	Down expressed	PAU8	seriPAUperin
YBR233W	Down expressed	PBP2	Pbp1p Binding Protein
YIL071C	Down expressed	PCI8	Proteasome-COP9 signalosome (CSN)-eIF3
YPR002W	Down expressed	PDH1	prpD Homolog
YCL043C	Down expressed	PDI1	Protein Disulfide Isomerase
YMR076C	Down expressed	PDS5	Precocious Dissociation of Sisters
YOR158W	Down expressed	PET123	PETite colonies
YOR017W	Down expressed	PET127	PETite colonies

YGR239C	Down expressed	PEX21	PEroXin
YMR105C	Down expressed	PGM2	PhosphoGlucoMutase
YDR481C	Down expressed	PHO8	PHOsphate metabolism
YJL117W	Down expressed	PHO86	PHOsphate metabolism
YNL267W	Down expressed	PIK1	Phosphatidyl Inositol Kinase
YOL100W	Down expressed	PKH2	Pkb-activating Kinase Homolog
YKR046C	Down expressed	PLN1	PeriLipiN
YOR281C	Down expressed	PLP2	Phosducin-Like Protein
YDR276C	Down expressed	PMP3	Plasma Membrane Proteolipid
YGL037C	Down expressed	PNC1	Pyrazinamidase and NiCotinamidase
YNL055C	Down expressed	POR1	PORin
YDL134C	Down expressed	PPH21	Protein PHosphatase
YOL141W	Down expressed	PPM2	Protein Phosphatase Methyltransferase
YML016C	Down expressed	PPZ1	Protein Phosphatase Z
YER012W	Down expressed	PRE1	PRoteinase yscE
YIL095W	Down expressed	PRK1	p53 Regulatory Kinase
YJL108C	Down expressed	PRM10	Pheromone-Regulated Membrane protein
YIL037C	Down expressed	PRM2	Pheromone-Regulated Membrane protein
YDL043C	Down expressed	PRP11	Pre-mRNA Processing
YKR086W	Down expressed	PRP16	Pre-mRNA Processing
YNR011C	Down expressed	PRP2	Pre-mRNA Processing
YKL116C	Down expressed	PRR1	Pheromone Response Regulator
YKL181W	Down expressed	PRS1	PhosphoRibosylpyrophosphate Synthetase
YDR013W	Down expressed	PSF1	Partner of Sld Five
YMR137C	Down expressed	PSO2	PSOralen derivative sensitive
YBR125C	Down expressed	PTC4	Phosphatase Two C
YOR243C	Down expressed	PUS7	PseudoUridine Synthase
YOR348C	Down expressed	PUT4	Proline UTilization
YHR001W-A	Down expressed	QCR10	ubiQuinol-cytochrome C oxidoReductase
YJR035W	Down expressed	RAD26	RADiation sensitive
YDR030C	Down expressed	RAD28	RADiation sensitive
YGR173W	Down expressed	RBG2	RiBosome interacting Gtpase
YOR127W	Down expressed	RGA1	Rho GTPase Activating Protein
YBR260C	Down expressed	RGD1	Related GAP Domain
YER067W	Down expressed	RGI1	Respiratory Growth Induced
YKR055W	Down expressed	RHO4	Ras HOmolog
YNL180C	Down expressed	RHO5	Ras HOmolog
YOL143C	Down expressed	RIB4	RIBoflavin biosynthesis
YGR250C	Down expressed	RIE1	Restoration of Impaired growths of ERMES-lacking cells

YFL033C	Down expressed	RIM15	Regulator of IME2
YEL024W	Down expressed	RIP1	Rieske Iron-sulfur Protein
YMR283C	Down expressed	RIT1	Ribosylation of Initiator tRNA
YLL034C	Down expressed	RIX7	RIbosome eXport
YHR177W	Down expressed	ROF1	Regulator Of Fluffy
YFR022W	Down expressed	ROG3	Revertant Of Glycogen synthase kinase mutation
YJR063W	Down expressed	RPA12	RNA Polymerase A
YLR029C	Down expressed	RPL15A	Ribosomal Protein of the Large subunit
YGL030W	Down expressed	RPL30	Ribosomal Protein of the Large subunit
YDR064W	Down expressed	RPS13	Ribosomal Protein of the Small subunit
YPL152W	Down expressed	RRD2	Resistant to Rapamycin Deletion
YOR305W	Down expressed	RRG7	Required for Respiratory Growth
YPL012W	Down expressed	RRP12	Ribosomal RNA Processing
YKL082C	Down expressed	RRP14	Ribosomal RNA Processing
YJR101W	Down expressed	RSM26	Ribosomal Small subunit of Mitochondria
YGR152C	Down expressed	RSR1	RaS-Related
YHR079C-A	Down expressed	SAE3	Sporulation in the Absence of spo Eleven
YMR272C	Down expressed	SCS7	Suppressor of Ca2+ Sensitivity
YBL011W	Down expressed	SCT1	Suppressor of Choline-Transport mutants
YOL098C	Down expressed	SDD3	Suppressor of Degenerative Death
YKL148C	Down expressed	SDH1	Succinate DeHydrogenase
YDR178W	Down expressed	SDH4	Succinate DeHydrogenase
YIL113W	Down expressed	SDP1	Stress-inducible Dual specificity Phosphatase
YDR498C	Down expressed	SEC20	SECretory
YDR166C	Down expressed	SEC5	SECretory
YDR077W	Down expressed	SED1	Suppression of Exponential Defect
YGR208W	Down expressed	SER2	SERine requiring
YDR023W	Down expressed	SES1	SEryl-tRNA Synthetase
YJL168C	Down expressed	SET2	SET domain-containing
YBL102W	Down expressed	SFT2	Suppressor of sed Five Ts
YIL099W	Down expressed	SGA1	Sporulation-specific GlycoAmylase
YIR001C	Down expressed	SGN1	Slower Growth on Non-fermentable carbon sources
YLR079W	Down expressed	SIC1	Substrate/Subunit Inhibitor of Cyclin-dependent protein kinase
YMR175W	Down expressed	SIP18	Salt Induced Protein
YMR216C	Down expressed	SKY1	SRPK1-like Kinase in Yeast
YGL113W	Down expressed	SLD3	Synthetically Lethal with Dpb11-1
YDR489W	Down expressed	SLD5	Synthetic Lethality with Dpb11-1
YOR060C	Down expressed	SLD7	Synthetic Lethality with Dpb11-24
YBR266C	Down expressed	SLM6	Synthetic Lethal with Mss4

YDR088C	Down expressed	SLU7	Synergistic Lethal with U5 snRNA
YLR135W	Down expressed	SLX4	Synthetic Lethal of unknown (X) function
YGR081C	Down expressed	SLX9	0
YER029C	Down expressed	SMB1	SmB/B' homolog
YNR015W	Down expressed	SMM1	Suppressor of Mitochondrial Mutation
YOR290C	Down expressed	SNF2	Sucrose NonFermenting
YGR013W	Down expressed	SNU71	Small NUclear ribonucleoprotein associated
YJR104C	Down expressed	SOD1	SuperOxide Dismutase
YHR008C	Down expressed	SOD2	SuperOxide Dismutase
YLL011W	Down expressed	SOF1	Suppressor Of Fibrillarin
YMR016C	Down expressed	SOK2	Suppressor Of Kinase
YJR159W	Down expressed	SOR1	0
YKL184W	Down expressed	SPE1	SPErmidine auxotroph
YDR504C	Down expressed	SPG3	Stationary Phase Gene
YER150W	Down expressed	SPI1	Stationary Phase Induced
YDR522C	Down expressed	SPS2	SPorulation Specific
YJL127C	Down expressed	SPT10	SuPpressor of Ty
YIR012W	Down expressed	SQT1	Suppressor of QSR1 Truncations
YCR018C	Down expressed	SRD1	0
YPR032W	Down expressed	SRO7	Suppressor of rho3
YAL005C	Down expressed	SSA1	Stress-Seventy subfamily A
YER103W	Down expressed	SSA4	Stress-Seventy subfamily A
YBR283C	Down expressed	SSH1	Sec Sixty-one Homolog
YCR073C	Down expressed	SSK22	Suppressor of Sensor Kinase
YDR160W	Down expressed	SSY1	Sulfonylurea Sensitive on YPD
YDL130W-A	Down expressed	STF1	STabilizing Factor
YCL008C	Down expressed	STP22	STerile Pseudoreversion
YDL048C	Down expressed	STP4	protein with similarity to Stp1p
YIR011C	Down expressed	STS1	Sec Twenty-three Suppressor 1
YML052W	Down expressed	SUR7	SUppressor of Rvs167 mutation
YDR320C	Down expressed	SWA2	Synthetic lethal With Arf1
YJL035C	Down expressed	TAD2	tRNA-specific Adenosine Deaminase
YBR198C	Down expressed	TAF5	TATA binding protein-Associated Factor
YJR046W	Down expressed	TAH11	Topo-A Hypersensitive
YLR010C	Down expressed	TEN1	TElomeric pathways with STn1
YJR019C	Down expressed	TES1	ThioESterase
YJR135W-A	Down expressed	TIM8	Translocase of the Inner Mitochondrial membrane
YBR067C	Down expressed	TIP1	Temperature shock-Inducible Protein
YDL110C	Down expressed	TMA17	Translation Machinery Associated

YJR085C	Down expressed	TMH11	TMem14 Homolog of 11 kDa
YLR118C	Down expressed	TML25	acyl-protein Thioesterase with Multiple Localizations, protein of 25 kDa
YKL058W	Down expressed	TOA2	0
YMR203W	Down expressed	TOM40	Translocase of the Outer Mitochondrial membrane
YNL300W	Down expressed	TOS6	0
YGR096W	Down expressed	TPC1	Thiamine Pyrophosphate Carrier
YAL016W	Down expressed	TPD3	tRNA Processing Deficient
YNL299W	Down expressed	TRF5	Topoisomerase one-Related Function
YMR233W	Down expressed	TRI1	0
YJL129C	Down expressed	TRK1	TRansport of potassium (K)
YJL087C	Down expressed	TRL1	tRNA Ligase
YKL034W	Down expressed	TUL1	Transmembrane Ubiquitin Ligase
YOR251C	Down expressed	TUM1	ThioUridine Modification
YHR111W	Down expressed	UBA4	UBiquitin-Activating
YBR165W	Down expressed	UBS1	UBiquitin-conjugating enzyme Suppressor
YOR191W	Down expressed	ULS1	Ubiquitin Ligase for SUMO conjugates
YIL008W	Down expressed	URM1	Ubiquitin Related Modifier
YGR090W	Down expressed	UTP22	U Three Protein
YEL005C	Down expressed	VAB2	VAc8p Binding
YOR106W	Down expressed	VAM3	VAcuolar Morphogenesis
YCL069W	Down expressed	VBA3	Vacuolar Basic Amino acid transporter
YER128W	Down expressed	VFA1	Vps Four-Associated
YIL056W	Down expressed	VHR1	VHt1 Regulator
YDR495C	Down expressed	VPS3	Vacuolar Protein Sorting
YLR240W	Down expressed	VPS34	Vacuolar Protein Sorting
YJR044C	Down expressed	VPS55	Vacuolar Protein Sorting
YDR372C	Down expressed	VPS74	Vacuolar Protein Sorting
YLR090W	Down expressed	XDJ1	0
YJR133W	Down expressed	XPT1	Xanthine Phosphoribosyl Transferase
YNL064C	Down expressed	YDJ1	Yeast dnaJ
YLL012W	Down expressed	YEH1	Yeast steryl Ester Hydrolase
YMR040W	Down expressed	YET2	Yeast Endoplasmic reticulum Transmembrane protein
YNL160W	Down expressed	YGP1	Yeast GlycoProtein
YMR284W	Down expressed	YKU70	Yeast KU protein
YIR039C	Down expressed	YPS6	YaPSin
YJL056C	Down expressed	ZAP1	Zinc-responsive Activator Protein
YGL255W	Down expressed	ZRT1	Zinc-Regulated Transporter
YLR130C	Down expressed	ZRT2	Zinc-Regulated Transporter
YBL005W-A	Down expressed		

YBR137W	Down expressed		
YBR242W	Down expressed		
YBR287W	Down expressed		
YCL042W	Down expressed		
YDL057W	Down expressed		
YDL124W	Down expressed		
YDL199C	Down expressed		
YDL206W	Down expressed		
YDR034C-D	Down expressed		
YDR098C-A	Down expressed		
YDR109C	Down expressed		
YDR210W-A	Down expressed		
YDR248C	Down expressed		
YDR261C-C	Down expressed		
YDR261W-A	Down expressed		
YDR262W	Down expressed		
YDR316W-A	Down expressed		
YDR338C	Down expressed		
YDR341C	Down expressed		
YDR365W-A	Down expressed		
YDR415C	Down expressed		
YDR433W	Down expressed		
YER137C-A	Down expressed		
YER138C	Down expressed		
YER159C-A	Down expressed		
YGL036W	Down expressed		
YGL114W	Down expressed		
YGR026W	Down expressed		
YGR054W	Down expressed		
YGR067C	Down expressed		
YHR033W	Down expressed		
YHR112C	Down expressed		
YIL001W	Down expressed		
YIL089W	Down expressed		
YJL163C	Down expressed		
YJL206C	Down expressed		
YKL070W	Down expressed		
YKR018C	Down expressed		
YKR070W	Down expressed		

YLR042C	Down expressed		
YLR269C	Down expressed		
YLR278C	Down expressed		
YLR345W	Down expressed		
YML018C	Down expressed		
YML082W	Down expressed		
YML131W	Down expressed		
YMR051C	Down expressed		
YMR295C	Down expressed		
YMR315W	Down expressed		
YNL134C	Down expressed		
YOL079W	Down expressed		
YOR192C-A	Down expressed		
YPL088W	Down expressed		
YKL134C	Down expressed		
ARA1	Down expressed		
ARA2	Down expressed		
ARA23	Down expressed		
ARA32	Down expressed		
YAL066W	Down expressed		
YAL069W	Down expressed		
YBL073W	Down expressed		
YBL109W	Down expressed		
YBR124W	Down expressed		
YCL009C-R	Down expressed		
YCR064C	Down expressed		
YDL016C	Down expressed		
YDL023C	Down expressed		
YDL050C	Down expressed		
YDR010C	Down expressed		
YDR034C-A	Down expressed		
YDR154C-R	Down expressed		
YDR199W	Down expressed		
YDR230W	Down expressed		
YDR249C	Down expressed		
YDR276C-R	Down expressed		
YDR286C	Down expressed		
YDR345C-R	Down expressed		
YDR366C	Down expressed		

YDR431W	Down expressed		
YDR445C	Down expressed		
YDR455C	Down expressed		
YDR521W	Down expressed		
YER188W	Down expressed		
YFL067W	Down expressed		
YGL069C	Down expressed		
YGL074C	Down expressed		
YGL176C	Down expressed		
YGR164W	Down expressed		
YGR182C	Down expressed		
YGR190C	Down expressed		
YGR226C	Down expressed		
YGR228W	Down expressed		
YGR269W	Down expressed		
YGR273C	Down expressed		
YHR021W-A	Down expressed		
YIL080W	Down expressed		
YJL028W	Down expressed		
YJL220W	Down expressed		
YJR023C	Down expressed		
YJR038C	Down expressed		
YJR162C	Down expressed		
YKL039W	Down expressed		
YKL096W-A-R	Down expressed		
YKL102C	Down expressed		
YKL111C	Down expressed		
YKL131W	Down expressed		
YKL182W-R	Down expressed		
YKL225W	Down expressed		
YKR047W	Down expressed		
YLL030C	Down expressed		
YLL065W	Down expressed		
YLR149C	Down expressed		
YLR162W	Down expressed		
YLR198C	Down expressed		
YLR390W-A-R	Down expressed		

YLR402W	Down expressed		
YLR465C	Down expressed		
YML058C-A	Down expressed		
YML100W-A	Down expressed		
YMR103C	Down expressed		
YMR107W	Down expressed		
YMR158C-B	Down expressed		
YMR173W-A	Down expressed		
YMR206W	Down expressed		
YNL203C	Down expressed		
YNL228W	Down expressed		
YNL285W	Down expressed		
YOL024W	Down expressed		
YOL037C	Down expressed		
YOL106W	Down expressed		
YOR015W	Down expressed		
YOR053W	Down expressed		
YOR082C	Down expressed		
YOR097C	Down expressed		
YOR331C	Down expressed		
YPL054W	Down expressed		
YPL056C	Down expressed		
YPL062W	Down expressed		
YPL102C	Down expressed		
YPL182C	Down expressed		
YPR002C-A	Down expressed		
YPR014C	Down expressed		
YPR117W	Down expressed		
YPR136C	Down expressed		
YPR142C	Down expressed		
**Genes which expression changed in AT22 evolved strain compared with the parental strain S288C**			
**Systematic Name**	**Expression**	**Standard Name**	**Name Description**
YJR155W	Over expressed	AAD10	Aryl-Alcohol Dehydrogenase
YCR088W	Over expressed	ABP1	Actin Binding Protein
YOR239W	Over expressed	ABP140	Actin Binding Protein
YLR144C	Over expressed	ACF2	Assembly Complementing Factor
YPL267W	Over expressed	ACM1	APC/C[Cdh1] Modulator
YLR153C	Over expressed	ACS2	Acetyl CoA Synthetase

YCR010C	Over expressed	ADY2	Accumulation of DYads
YNR044W	Over expressed	AGA1	a-AGglutinin
YCR082W	Over expressed	AHC2	Ada Histone acetyltransferase complex Component
YNR074C	Over expressed	AIF1	Apoptosis-Inducing Factor
YHL021C	Over expressed	AIM17	Altered Inheritance rate of Mitochondria
YBR243C	Over expressed	ALG7	Asparagine-Linked Glycosylation
YNL270C	Over expressed	ALP1	
YEL036C	Over expressed	ANP1	ANP and osmotic sensitive
YHR126C	Over expressed	ANS1	
YHR113W	Over expressed	APE4	
YIL040W	Over expressed	APQ12	APical growth revealed by Quantitative morphological analysis
YFL054C	Over expressed	AQY3	AQuaporin from Yeast
YER036C	Over expressed	ARB1	ATP-binding cassette protein involved in Ribosome Biogenesis
YDL192W	Over expressed	ARF1	ADP-Ribosylation Factor
YGL157W	Over expressed	ARI1	Aldehyde Reductase Intermediate, subclass of SDR
YNL020C	Over expressed	ARK1	Actin Regulating Kinase
YDL088C	Over expressed	ASM4	Anti-Suppressor in Multicopy
YLR160C	Over expressed	ASP3-4	ASParaginase
YGL180W	Over expressed	ATG1	AuTophaGy related
YPR049C	Over expressed	ATG11	AuTophaGy related
YJL178C	Over expressed	ATG27	AuTophaGy related
YPL166W	Over expressed	ATG29	AuTophaGy related
YPR026W	Over expressed	ATH1	Acid TreHalase
YDR384C	Over expressed	ATO3	Ammonia (Ammonium) Transport Outward
YLR295C	Over expressed	ATP14	ATP synthase
Q0085	Over expressed	ATP6	ATP synthase
YIL124W	Over expressed	AYR1	1-AcyldihYdroxyacetone-phosphate Reductase
YOR134W	Over expressed	BAG7	
YLR399C	Over expressed	BDF1	BromoDomain Factor
YOL164W	Over expressed	BDS1	Bacterially Derived Sulfatase
YOR198C	Over expressed	BFR1	BreFeldin A Resistance
YNL271C	Over expressed	BNI1	Bud Neck Involved
YLR015W	Over expressed	BRE2	BREfeldin A sensitivity
YGR188C	Over expressed	BUB1	Budding Uninhibited by Benzimidazole
YGL174W	Over expressed	BUD13	BUD site selection
YEL029C	Over expressed	BUD16	BUD site selection
YKL092C	Over expressed	BUD2	BUD site selection
YDL151C	Over expressed	BUD30	BUD site selection
YCR063W	Over expressed	BUD31	BUD site selection
YOR299W	Over expressed	BUD7	BUD site selection
YHR114W	Over expressed	BZZ1	
YOR276W	Over expressed	CAF20	Cap Associated Factor
YLR175W	Over expressed	CBF5	Centromere Binding Factor
YNL161W	Over expressed	CBK1	Cell wall Biosynthesis Kinase
YPR025C	Over expressed	CCL1	

YDR188W	Over expressed	CCT6	Chaperonin Containing TCP-1
YLR390W-A	Over expressed	CCW14	Covalently linked Cell Wall protein
YOR074C	Over expressed	CDC21	Cell Division Cycle
YOL139C	Over expressed	CDC33	Cell Division Cycle
YPL160W	Over expressed	CDC60	Cell Division Cycle
YPL008W	Over expressed	CHL1	CHromosome Loss
YLR133W	Over expressed	CKI1	Choline KInase
YGR109C	Over expressed	CLB6	CycLin B
YPL256C	Over expressed	CLN2	CycLiN
Q0045	Over expressed	COX1	Cytochrome c OXidase
YLL009C	Over expressed	COX17	Cytochrome c OXidase
YGR062C	Over expressed	COX18	Cytochrome c OXidase
YMR256C	Over expressed	COX7	Cytochrome c OXidase
YOR303W	Over expressed	CPA1	Carbamyl Phosphate synthetase A
YHR209W	Over expressed	CRG1	Cantharidin Resistance Gene
YGR189C	Over expressed	CRH1	Congo Red Hypersensitive
YLR087C	Over expressed	CSF1	Cold Sensitive for Fermentation
YIL132C	Over expressed	CSM2	Chromosome Segregation in Meiosis
YFR020W	Over expressed	CSS2	Condition Specific Secretion
YLR411W	Over expressed	CTR3	Copper TRansport
YDR482C	Over expressed	CWC21	Complexed With Cef1p
YGR278W	Over expressed	CWC22	Complexed With Cef1p
YGL128C	Over expressed	CWC23	Complexed With Cef1p
YLR323C	Over expressed	CWC24	Complexed With Cef1p
YKR034W	Over expressed	DAL80	Degradation of Allantoin
YDR020C	Over expressed	DAS2	Dst1-delta 6-Azauracil Sensitivity
YDR052C	Over expressed	DBF4	DumbBell Former
YNR038W	Over expressed	DBP6	Dead Box Protein
YKL149C	Over expressed	DBR1	DeBRanching
YCL016C	Over expressed	DCC1	Defective in sister Chromatid Cohesion
YLR128W	Over expressed	DCN1	Defective in Cullin Neddylation
YPR082C	Over expressed	DIB1	S. pombe DIm1+ in Budding yeast
YLR437C	Over expressed	DIF1	Damage-regulated Import Facilitator
YPL265W	Over expressed	DIP5	DIcarboxylic amino acid Permease
YHR164C	Over expressed	DNA2	DNA synthesis defective
YOR005C	Over expressed	DNL4	DNA Ligase
YLL001W	Over expressed	DNM1	DyNaMin-related
YDR440W	Over expressed	DOT1	Disruptor Of Telomeric silencing
YER088C	Over expressed	DOT6	Disruptor Of Telomeric silencing
YBR278W	Over expressed	DPB3	DNA Polymerase B (II) subunit
YKR071C	Over expressed	DRE2	Derepressed for Ribosomal protein S14 Expression
YIR010W	Over expressed	DSN1	Dosage Suppressor of NNF1
YDR359C	Over expressed	EAF1	Esa1p-Associated Factor
YNL136W	Over expressed	EAF7	Esa1-Associated Factor
YDR125C	Over expressed	ECM18	ExtraCellular Mutant

YLR228C	Over expressed	ECM22	ExtraCellular Mutant
YKR004C	Over expressed	ECM9	ExtraCellular Mutant
YMR212C	Over expressed	EFR3	PHO Eighty Five Requiring
YDR036C	Over expressed	EHD3	
YLR050C	Over expressed	EMA19	Efficient Mitochondria targeting-Associated protein
YLR083C	Over expressed	EMP70	
YIL005W	Over expressed	EPS1	ER-retained Pma1 Suppressing
YMR220W	Over expressed	ERG8	ERGosterol biosynthesis
YML067C	Over expressed	ERV41	ER Vesicle
YLR318W	Over expressed	EST2	Ever Shorter Telomeres
YOR051C	Over expressed	ETT1	Enhancer of Translation Termination 1
YNL127W	Over expressed	FAR11	Factor ARrest
YKL187C	Over expressed	FAT3	FATty acid transporter 3
YER056C	Over expressed	FCY2	FluoroCYtosine resistance
YER060W-A	Over expressed	FCY22	FluoroCYtosine resistance
YMR058W	Over expressed	FET3	FErrous Transport
YFL041W	Over expressed	FET5	FErrous Transport
YCR089W	Over expressed	FIG2	Factor-Induced Gene
YKR102W	Over expressed	FLO10	FLOcculation
YHR211W	Over expressed	FLO5	FLOcculation
YPL222W	Over expressed	FMP40	Found in Mitochondrial Proteome
YML074C	Over expressed	FPR3	Fk 506-sensitive Proline Rotamase
YLL029W	Over expressed	FRA1	Fe Repressor of Activation
YLR214W	Over expressed	FRE1	Ferric REductase
YBR207W	Over expressed	FTH1	FTR1 Homolog
YDR024W	Over expressed	FYV1	Function required for Yeast Viability
YGR196C	Over expressed	FYV8	Function required for Yeast Viability
YER027C	Over expressed	GAL83	GALactose metabolism
YGR252W	Over expressed	GCN5	General Control Nonderepressible
YPR184W	Over expressed	GDB1	Glycogen DeBranching
YER136W	Over expressed	GDI1	GDP Dissociation Inhibitor
YKR106W	Over expressed	GEX2	Glutathione EXchanger
YKL104C	Over expressed	GFA1	Glutamine:Fructose-6-phosphate Amidotransferase
YDL198C	Over expressed	GGC1	GDP/GTP Carrier
YDR309C	Over expressed	GIC2	GTPase Interactive Component
YDR152W	Over expressed	GIR2	Genetically Interacts with Ribosomal genes
YDL207W	Over expressed	GLE1	GLFG (glycine-leucine-phenylalanine-glycine) LEthal
YOR040W	Over expressed	GLO4	GLyOxalase
YDR302W	Over expressed	GPI11	GlycosylPhosphatidylInositol anchor biosynthesis
YDR331W	Over expressed	GPI8	GlycosylPhosphatidylInositol anchor biosynthesis
YKR067W	Over expressed	GPT2	Glycerol-3-Phosphate acylTransferase
YDR098C	Over expressed	GRX3	GlutaRedoXin
YLR293C	Over expressed	GSP1	Genetic Suppressor of Prp20-1
YLR258W	Over expressed	GSY2	Glycogen SYnthase
YDR221W	Over expressed	GTB1	Glucosidase Two Beta-subunit

YML121W	Over expressed	GTR1	GTp binding protein Resemblance
YGL181W	Over expressed	GTS1	Glycine Threonine Serine repeat protein
YIR038C	Over expressed	GTT1	GlutaThione Transferase
YPL189W	Over expressed	GUP2	Glycerol UPtake
YDL234C	Over expressed	GYP7	Gtpase-activating protein for Ypt7 Protein
YPR005C	Over expressed	HAL1	HALotolerance
YCR065W	Over expressed	HCM1	High-Copy suppressor of Calmodulin
YBL032W	Over expressed	HEK2	HEterogeneous nuclear rnp K-like gene
YPL254W	Over expressed	HFI1	Histone H2A Functional Interactor
YPL127C	Over expressed	HHO1	Histone H One
YEL059W	Over expressed	HHY1	Hypersensitivity to HYgromycin B
YDR174W	Over expressed	HMO1	High MObility group (HMG) family
YGL077C	Over expressed	HNM1	Hyper-resistance to Nitrogen Mustard
YDR158W	Over expressed	HOM2	HOMoserine requiring
YOL155C	Over expressed	HPF1	Haze Protective Factor
YOL013C	Over expressed	HRD1	HMG-coA Reductase Degradation
YOR267C	Over expressed	HRK1	Hygromycin Resistance Kinase
YJR122W	Over expressed	IBA57	Iron-sulfur cluster assembly factor for Biotin synthase and Aconitase-like mitochondrial proteins
YPL117C	Over expressed	IDI1	Isopentenyl Diphosphate Isomerase
YHR132W-A	Over expressed	IGO2	Initiation of G zerO
YJL057C	Over expressed	IKS1	
YJR118C	Over expressed	ILM1	Increased Loss of Mitochondrial DNA
YMR108W	Over expressed	ILV2	IsoLeucine-plus-Valine requiring
YJL216C	Over expressed	IMA5	IsoMAltase
YIL154C	Over expressed	IMP2'	Independent of Mitochondrial Particle
YNL106C	Over expressed	INP52	INositol polyphosphate 5-Phosphatase
YJR141W	Over expressed	IPA1	Important for cleavage and PolyAdenylation
YMR073C	Over expressed	IRC21	Increased Recombination Centers
YDR332W	Over expressed	IRC3	Increased Recombination Centers
YFR038W	Over expressed	IRC5	Increased Recombination Centers
YFR043C	Over expressed	IRC6	Increased Recombination Centers
YFR055W	Over expressed	IRC7	Increased Recombination Centers
YPL040C	Over expressed	ISM1	Isoleucyl tRNA Synthetase of Mitochondria
YBR086C	Over expressed	IST2	Increased Sodium Tolerance
YDR229W	Over expressed	IVY1	Interacting with Vps33p and Ypt7p
YDR492W	Over expressed	IZH1	Implicated in Zinc Homeostasis
YLR023C	Over expressed	IZH3	Implicated in Zinc Homeostasis
YNL227C	Over expressed	JJJ1	J-protein (Type III)
YMR132C	Over expressed	JLP2	dnaJ-Like Protein
YCL024W	Over expressed	KCC4	
YKL161C	Over expressed	KDX1	Kinase Dead X-talker
YFR042W	Over expressed	KEG1	Kre6-binding ER protein responsible for Glucan synthesis
YGL203C	Over expressed	KEX1	Killer EXpression defective
YDR532C	Over expressed	KRE28	
YJL174W	Over expressed	KRE9	Killer toxin REsistant

YDR037W	Over expressed	KRS1	Lysyl (K) tRNA Synthetase
YBR199W	Over expressed	KTR4	Kre Two Related
YMR296C	Over expressed	LCB1	Long-Chain Base
YLR260W	Over expressed	LCB5	Long-Chain Base
YDR499W	Over expressed	LCD1	Lethal, Checkpoint-defective, DNA damage sensitive
YER127W	Over expressed	LCP5	Lethal with Conditional Pap1
YLR451W	Over expressed	LEU3	LEUcine biosynthesis
YHR002W	Over expressed	LEU5	LEUcine biosynthesis
YLL007C	Over expressed	LMO1	eLMO homolog
YHR192W	Over expressed	LNP1	LuNaPark family member
YPR139C	Over expressed	LOA1	Lysophosphatidic acid: Oleoyl-CoA Acyltransferase
YKL205W	Over expressed	LOS1	Loss Of Suppression
YOR142W	Over expressed	LSC1	Ligase of Succinyl-CoA
YDR378C	Over expressed	LSM6	Like SM
YKL176C	Over expressed	LST4	Lethal with Sec Thirteen
YNL260C	Over expressed	LTO1	required for biogenesis of the Large ribosomal subunit and initiation of Translation in Oxygen
YPR051W	Over expressed	MAK3	MAintenance of Killer
YDL056W	Over expressed	MBP1	MluI-box Binding Protein
YDL003W	Over expressed	MCD1	Mitotic Chromosome Determinant
YDL054C	Over expressed	MCH1	MonoCarboxylate transporter Homolog
YEL032W	Over expressed	MCM3	MiniChromosome Maintenance
YPR083W	Over expressed	MDM36	Mitochondrial Distribution and Morphology
YLR303W	Over expressed	MET17	METhionine requiring
YPL038W	Over expressed	MET31	METhionine requiring
YJR144W	Over expressed	MGM101	Mitochondrial Genome Maintenance
YKL089W	Over expressed	MIF2	MItotic Fidelity of chromosome transmission
YGL035C	Over expressed	MIG1	Multicopy Inhibitor of GAL gene expression
YGL209W	Over expressed	MIG2	Multicopy Inhibitor of GAL gene expression
YEL007W	Over expressed	MIT1	Muc1 expressed Independent of TEC1
YDR031W	Over expressed	MIX14	Mitochondrial Intermembrane space CX(n)C motif protein
YMR002W	Over expressed	MIX17	Mitochondrial Intermembrane space CX(n)C motif protein
YJR039W	Over expressed	MLO127	Mitochondrially LOcalized protein of 127 kDa
YBR098W	Over expressed	MMS4	Methyl MethaneSulfonate sensitivity
YPL224C	Over expressed	MMT2	Mitochondrial Metal Transporter
YER068W	Over expressed	MOT2	Modulator Of Transcription
YHR162W	Over expressed	MPC2	Mitochondrial Pyruvate Carrier
YGL178W	Over expressed	MPT5	Multicopy suppressor of Pop Two
YGL143C	Over expressed	MRF1	Mitochondrial peptide chain Release Factor
YGR084C	Over expressed	MRP13	Mitochondrial Ribosomal Protein
YNL177C	Over expressed	MRPL22	Mitochondrial Ribosomal Protein, Large subunit
YMR024W	Over expressed	MRPL3	Mitochondrial Ribosomal Protein, Large subunit
YDR322W	Over expressed	MRPL35	Mitochondrial Ribosomal Protein, Large subunit
YGR220C	Over expressed	MRPL9	Mitochondrial Ribosomal Protein, Large subunit
YPL168W	Over expressed	MRX4	Mitochondrial oRganization of gene eXpression (MIOREX)
YOR066W	Over expressed	MSA1	Mbf and Sbf Associated

YOL090W	Over expressed	MSH2	MutS Homolog
YPR134W	Over expressed	MSS18	Mitochondrial Splicing System
YHL036W	Over expressed	MUP3	Methionine UPtake
YPL190C	Over expressed	NAB3	Nuclear polyAdenylated RNA-Binding
YNL240C	Over expressed	NAR1	Nuclear Architecture Related
YGR147C	Over expressed	NAT2	N-terminal AcetylTransferase
YNL036W	Over expressed	NCE103	NonClassical Export
YNL119W	Over expressed	NCS2	Needs Cla4 to Survive
YLR254C	Over expressed	NDL1	NuDeL homolog
YDR456W	Over expressed	NHX1	Na+/H+ eXchanger
YLR315W	Over expressed	NKP2	Non-essential Kinetochore Protein
YOR206W	Over expressed	NOC2	NucleOlar Complex associated
YOL144W	Over expressed	NOP8	NucleOlar Protein
YEL062W	Over expressed	NPR2	Nitrogen Permease Regulator
YIR035C	Over expressed	NRE1	Novel REductase
YDL193W	Over expressed	NUS1	Nuclear Undecaprenyl pyrophosphate Synthase
YHL029C	Over expressed	OCA5	Oxidant-induced Cell-cycle Arrest
YDR067C	Over expressed	OCA6	Oxidant-induced Cell-cycle Arrest
YDR316W	Over expressed	OMS1	OXA1 Multicopy Suppressor
YJL212C	Over expressed	OPT1	OligoPeptide Transporter
YLL004W	Over expressed	ORC3	Origin Recognition Complex
YJR051W	Over expressed	OSM1	OSMotic sensitivity
YFL044C	Over expressed	OTU1	Ovarian TUmor
YOR269W	Over expressed	PAC1	Perish in the Absence of Cin8p
YMR174C	Over expressed	PAI3	Proteinase A Inhibitor
YEL049W	Over expressed	PAU2	seriPAUperin family
YBR233W	Over expressed	PBP2	Pbp1p Binding Protein
YDL053C	Over expressed	PBP4	Pbp1p Binding Protein
YDL127W	Over expressed	PCL2	Pho85 CycLin
YGR101W	Over expressed	PCP1	Processing of Cytochrome c Peroxidase
YGR087C	Over expressed	PDC6	Pyruvate DeCarboxylase
YGL248W	Over expressed	PDE1	PhosphoDiEsterase
YMR087W	Over expressed	PDL32	Protein of Dual Localization of 32 kDa
YIL013C	Over expressed	PDR11	Pleiotropic Drug Resistance
YDR406W	Over expressed	PDR15	Pleiotropic Drug Resistance
YMR076C	Over expressed	PDS5	Precocious Dissociation of Sisters
YER058W	Over expressed	PET117	PETite colonies
YPL159C	Over expressed	PET20	PETite colonies
YDR329C	Over expressed	PEX3	PEroXin
YMR018W	Over expressed	PEX9	PEroXin
YNL317W	Over expressed	PFS2	Polyadenylation Factor Subunit
YCR012W	Over expressed	PGK1	3-PhosphoGlycerate Kinase
YOL084W	Over expressed	PHM7	PHosphate Metabolism
YDL236W	Over expressed	PHO13	PHOsphate metabolism
YDL106C	Over expressed	PHO2	PHOsphate metabolism

YDR481C	Over expressed	PHO8	PHOsphate metabolism
YPL031C	Over expressed	PHO85	PHOsphate metabolism
YNR013C	Over expressed	PHO91	PHOsphate metabolism
YLR273C	Over expressed	PIG1	Protein Interacting with Gsy2p
YKL163W	Over expressed	PIR3	Protein containing Internal Repeats
YDR466W	Over expressed	PKH3	Pkb-activating Kinase Homolog
YPL268W	Over expressed	PLC1	PhosphoLipase C
YER003C	Over expressed	PMI40	PhosphoMannose Isomerase
YOR321W	Over expressed	PMT3	Protein O-MannosylTransferase
YGR199W	Over expressed	PMT6	Protein O-MannosylTransferase
YLL023C	Over expressed	POM33	POre Membrane, 33 kDa
YNL282W	Over expressed	POP3	Processing Of Precursor RNAs
YDL188C	Over expressed	PPH22	Protein PHosphatase
YPL148C	Over expressed	PPT2	Phosphopantetheine:Protein Transferase
YML092C	Over expressed	PRE8	PRoteinase yscE
YIL095W	Over expressed	PRK1	p53 Regulatory Kinase
YPL156C	Over expressed	PRM4	Pheromone-Regulated Membrane protein
YGR006W	Over expressed	PRP18	Pre-mRNA Processing
YDR473C	Over expressed	PRP3	Pre-mRNA Processing
YHR165C	Over expressed	PRP8	Pre-mRNA Processing
YKL181W	Over expressed	PRS1	PhosphoRibosylpyrophosphate Synthetase
YHL011C	Over expressed	PRS3	PhosphoRibosylpyrophosphate Synthetase
YKR013W	Over expressed	PRY2	Pathogen Related in Yeast
YGR170W	Over expressed	PSD2	PhosphatidylSerine Decarboxylase
YDR013W	Over expressed	PSF1	Partner of Sld Five
YDR505C	Over expressed	PSP1	Polymerase SuPpressor
YOR208W	Over expressed	PTP2	Protein Tyrosine Phosphatase
YKR093W	Over expressed	PTR2	Peptide TRansport
YGL014W	Over expressed	PUF4	PUmilio-homology domain Family
YLR204W	Over expressed	QRI5	Quasi-Renownless Information
YDL104C	Over expressed	QRI7	
YML095C	Over expressed	RAD10	RADiation sensitive
YDR030C	Over expressed	RAD28	RADiation sensitive
YDR314C	Over expressed	RAD34	Homologous to RAD4
YDR004W	Over expressed	RAD57	RADiation sensitive
YGL058W	Over expressed	RAD6	RADiation sensitive
YJR033C	Over expressed	RAV1	Regulator of (H+)-ATPase in Vacuolar membrane
YOR220W	Over expressed	RCN2	Regulator of CalciNeurin
YOR286W	Over expressed	RDL2	RhoDanese-Like protein
YOR380W	Over expressed	RDR1	Repressor of Drug Resistance
YMR133W	Over expressed	REC114	RECombination
YPL010W	Over expressed	RET3	RETrieval from ER
YOR127W	Over expressed	RGA1	Rho GTPase Activating Protein
YER067W	Over expressed	RGI1	Respiratory Growth Induced
YKR055W	Over expressed	RHO4	Ras HOmolog

YBR275C	Over expressed	RIF1	RAP1-Interacting Factor
YMR063W	Over expressed	RIM9	Regulator of IME2
YPL089C	Over expressed	RLM1	Resistance to Lethality of MKK1P386 overexpression
YGL250W	Over expressed	RMR1	Reduced Meiotic Recombination
YCL028W	Over expressed	RNQ1	Rich in asparagine (N) and glutamine (Q)
YFR022W	Over expressed	ROG3	Revertant Of Glycogen synthase kinase mutation
YOR341W	Over expressed	RPA190	RNA Polymerase A
YJL148W	Over expressed	RPA34	RNA Polymerase A
YDR404C	Over expressed	RPB7	RNA Polymerase B
YNL113W	Over expressed	RPC19	RNA Polymerase C
YKR025W	Over expressed	RPC37	RNA Polymerase C
YGL135W	Over expressed	RPL1B	Ribosomal Protein of the Large subunit
YGL031C	Over expressed	RPL24A	Ribosomal Protein of the Large subunit
YLR448W	Over expressed	RPL6B	Ribosomal Protein of the Large subunit
YPR108W	Over expressed	RPN7	Regulatory Particle Non-ATPase
YLR333C	Over expressed	RPS25B	Ribosomal Protein of the Small subunit
YER131W	Over expressed	RPS26B	Ribosomal Protein of the Small subunit
YKL156W	Over expressed	RPS27A	Ribosomal Protein of the Small subunit
YKL145W	Over expressed	RPT1	Regulatory Particle Triple-A protein, or Regulatory Particle Triphosphatase
YOR259C	Over expressed	RPT4	Regulatory Particle Triple-A protein, or Regulatory Particle Triphosphatase
YGL048C	Over expressed	RPT6	Regulatory Particle Triple-A protein, or Regulatory Particle Triphosphatase
YPL152W	Over expressed	RRD2	Resistant to Rapamycin Deletion
YOL142W	Over expressed	RRP40	Ribosomal RNA Processing
YDR280W	Over expressed	RRP45	Ribosomal RNA Processing
YIL093C	Over expressed	RSM25	Ribosomal Small subunit of Mitochondria
YDR494W	Over expressed	RSM28	Ribosomal Small subunit of Mitochondria
YHR087W	Over expressed	RTC3	Restriction of Telomere Capping
YPL183W-A	Over expressed	RTC6	Restriction of Telomere Capping
YDL025C	Over expressed	RTK1	Ribosome biogenesis and TRNA synthetase-associated Kinase
YDR066C	Over expressed	RTR2	Regulator of TRanscription
YLL002W	Over expressed	RTT109	Regulator of Ty1 Transposition
YHR079C-A	Over expressed	SAE3	Sporulation in the Absence of spo Eleven
YER043C	Over expressed	SAH1	S-Adenosyl-l-Homocysteine hydrolase
YDR143C	Over expressed	SAN1	Sir Antagonist
YDL153C	Over expressed	SAS10	Something About Silencing
YER019C-A	Over expressed	SBH2	Sec61 beta homolog 2
YMR272C	Over expressed	SCS7	Suppressor of Ca2+ Sensitivity
YGL083W	Over expressed	SCY1	
YMR074C	Over expressed	SDD2	Suppressor of Degenerative Death
YNL287W	Over expressed	SEC21	SECretory
YMR013C	Over expressed	SEC59	SECretory
YLR430W	Over expressed	SEN1	Splicing ENdonuclease
YJL168C	Over expressed	SET2	SET domain-containing
YIL099W	Over expressed	SGA1	Sporulation-specific GlycoAmylase
YPL047W	Over expressed	SGF11	SaGa associated Factor 11kDa

YGL066W	Over expressed	SGF73	SaGa associated Factor, 73 kDa
YOR057W	Over expressed	SGT1	Suppressor of G2 (Two) allele of skp1
YBR130C	Over expressed	SHE3	Swi5p-dependent HO Expression
YER118C	Over expressed	SHO1	Synthetic, High Osmolarity-sensitive
YKR072C	Over expressed	SIS2	SIt4 Suppressor
YHR149C	Over expressed	SKG6	Suppressor of lethality of Kex2 Gas1 double null mutant
YGR143W	Over expressed	SKN1	Suppressor of Kre Null
YHR206W	Over expressed	SKN7	Suppressor of Kre Null
YDR515W	Over expressed	SLF1	SuLFide production
YDR088C	Over expressed	SLU7	Synergistic Lethal with U5 snRNA
YLR135W	Over expressed	SLX4	Synthetic Lethal of unknown (X) function
YPL027W	Over expressed	SMA1	Spore Membrane Assembly
YML066C	Over expressed	SMA2	Spore Membrane Assembly
YOR159C	Over expressed	SME1	Sm protein E
YLR034C	Over expressed	SMF3	
YDR186C	Over expressed	SND1	Srp-iNDependent targeting
YNR023W	Over expressed	SNF12	Sucrose NonFermenting
YCR033W	Over expressed	SNT1	SaNT domains
YIL166C	Over expressed	SOA1	SulfOnAte transport
YOR353C	Over expressed	SOG2	
YMR016C	Over expressed	SOK2	Suppressor Of Kinase
YJL192C	Over expressed	SOP4	Suppressor Of Pma1-7
YFL002C	Over expressed	SPB4	Suppressor of PAB1
YMR017W	Over expressed	SPO20	SPOrulation
YPL175W	Over expressed	SPT14	SuPpressor of Ty
YKL020C	Over expressed	SPT23	SuPpressor of Ty
YDR392W	Over expressed	SPT3	SuPpressor of Ty's
YML034W	Over expressed	SRC1	Spliced mRNA and Cell cycle regulated gene
YCR018C	Over expressed	SRD1	
YKR091W	Over expressed	SRL3	Suppressor of rad53 Lethality
YPL210C	Over expressed	SRP72	Signal Recognition Particle
YMR101C	Over expressed	SRT1	Suppressor of Rer-Two
YIL030C	Over expressed	SSM4	Suppressor of mrna Stability Mutant
YHR184W	Over expressed	SSP1	
YHL007C	Over expressed	STE20	STErile
YJR117W	Over expressed	STE24	STErile
YOR212W	Over expressed	STE4	STErile
YJR130C	Over expressed	STR2	Sulfur TRansfer
YPR086W	Over expressed	SUA7	Suppressor of Upstream AUG
YPR151C	Over expressed	SUE1	
YNL244C	Over expressed	SUI1	SUppressor of Initiator codon
YPL057C	Over expressed	SUR1	SUppressor of Rvs161 and rvs167 mutations
YML052W	Over expressed	SUR7	SUppressor of Rvs167 mutation
YPR009W	Over expressed	SUT2	Sterol UpTake
YPL029W	Over expressed	SUV3	SUppressor of Var1

YPL032C	Over expressed	SVL3	Styryl dye Vacuolar Localization
YHR181W	Over expressed	SVP26	Sed5 Vesicle Protein
YDR126W	Over expressed	SWF1	Spore Wall Formation
YPL016W	Over expressed	SWI1	SWItching deficient
YFL049W	Over expressed	SWP82	SWi/snf-associated Protein
YAL014C	Over expressed	SYN8	SYNtaxin
YLR316C	Over expressed	TAD3	tRNA-specific Adenosine Deaminase
YPL129W	Over expressed	TAF14	TATA binding protein-Associated Factor
YPL128C	Over expressed	TBF1	TTAGGG repeat-Binding Factor
YBR150C	Over expressed	TBS1	ThiaBendazole Sensitive
YOR086C	Over expressed	TCB1	Three Calcium and lipid Binding domains (TriCalBins)
YER071C	Over expressed	TDA2	Topoisomerase I Damage Affected
YJR116W	Over expressed	TDA4	Topoisomerase I Damage Affected
YBR223C	Over expressed	TDP1	Tyrosyl-DNA Phosphodiesterase
YKL081W	Over expressed	TEF4	Translation Elongation Factor
YJR019C	Over expressed	TES1	ThioESterase
YGR047C	Over expressed	TFC4	Transcription Factor class C
YOR110W	Over expressed	TFC7	
YLR237W	Over expressed	THI7	THIamine metabolism
YOR192C	Over expressed	THI72	THI7 homolog 2
YLR004C	Over expressed	THI73	THIamine regulon
YOL072W	Over expressed	THP1	Tho2/Hpr1 Phenotype
YIL078W	Over expressed	THS1	THreonyl tRNA Synthetase
YPR163C	Over expressed	TIF3	Translation Initiation Factor
YLR136C	Over expressed	TIS11	similar to the mammalian TPA Induced Sequence gene family
YDR468C	Over expressed	TLG1	T-snare affecting a Late Golgi compartment
YOR091W	Over expressed	TMA46	Translation Machinery Associated
YDR457W	Over expressed	TOM1	Temperature dependent Organization in Mitotic nucleus or Trigger Of Mitosis
YNL121C	Over expressed	TOM70	Translocase of the Outer Mitochondrial membrane
YER049W	Over expressed	TPA1	Termination and PolyAdenylation
YGR138C	Over expressed	TPO2	Transporter of POlyamines
YPL176C	Over expressed	TRE1	Transferrin REceptor like
YDR120C	Over expressed	TRM1	tRNA Methyltransferase
YML014W	Over expressed	TRM9	TRna Methyltransferase
YLR043C	Over expressed	TRX1	ThioRedoXin
YML028W	Over expressed	TSA1	Thiol-Specific Antioxidant
YDL015C	Over expressed	TSC13	Temperature-sensitive Suppressors of Csg2 mutants
YKL034W	Over expressed	TUL1	Transmembrane Ubiquitin Ligase
YLR425W	Over expressed	TUS1	TOR Unique function Suppressor
YOR295W	Over expressed	UAF30	Upstream Activation Factor subunit
YDR059C	Over expressed	UBC5	UBiquitin-Conjugating
YDL091C	Over expressed	UBX3	UBiquitin regulatory X
YDL190C	Over expressed	UFD2	Ubiquitin Fusion Degradation
YOR075W	Over expressed	UFE1	Unknown Function Essential
YDL210W	Over expressed	UGA4	Utilization of GAba

YPL003W	Over expressed	ULA1	Ubiquitin-Like protein Activation
YOR191W	Over expressed	ULS1	Ubiquitin Ligase for SUMO conjugates
YPR152C	Over expressed	URN1	U2-U5-U6 snRNP, RES complex, and NTC interacting protein
YDR324C	Over expressed	UTP4	U Three Protein
YER082C	Over expressed	UTP7	U Three Protein
YOR068C	Over expressed	VAM10	VAcuolar Morphogenesis
YCL069W	Over expressed	VBA3	Vacuolar Basic Amino acid transporter
YDR119W	Over expressed	VBA4	Vacuolar Basic Amino acid transporter
YDL128W	Over expressed	VCX1	VaCuolar H+/Ca2+ eXchanger
YER128W	Over expressed	VFA1	Vps Four-Associated
YOR054C	Over expressed	VHS3	Viable in a Hal3 Sit4 background
YIL017C	Over expressed	VID28	Vacuolar Import and Degradation
YPL253C	Over expressed	VIK1	Vegetative Interaction with Kar3p
YIR014W	Over expressed	VLD1	Vacuole Localized Dsc protein
YPL234C	Over expressed	VMA11	Vacuolar Membrane Atpase
YKL080W	Over expressed	VMA5	Vacuolar Membrane Atpase
YEL051W	Over expressed	VMA8	Vacuolar Membrane Atpase
YDR495C	Over expressed	VPS3	Vacuolar Protein Sorting
YPL120W	Over expressed	VPS30	Vacuolar Protein Sorting
YLR396C	Over expressed	VPS33	Vacuolar Protein Sorting
YOL129W	Over expressed	VPS68	Vacuolar Protein Sorting
YDL224C	Over expressed	WHI4	WHIskey
YOL105C	Over expressed	WSC3	cell Wall integrity and Stress response Component
YHR134W	Over expressed	WSS1	Weak Suppressor of Smt3
YIL101C	Over expressed	XBP1	XhoI site-Binding Protein
YLR090W	Over expressed	XDJ1	
YHR161C	Over expressed	YAP1801	Yeast Assembly Polypeptide
YGR241C	Over expressed	YAP1802	Yeast Assembly Polypeptide
YAR035W	Over expressed	YAT1	
YLL055W	Over expressed	YCT1	Yeast Cysteine Transporter
YMR040W	Over expressed	YET2	Yeast Endoplasmic reticulum Transmembrane protein
YOR291W	Over expressed	YPK9	Yeast PARK9
YGR198W	Over expressed	YPP1	alpha-sYnuclein Protective Protein
YLR120C	Over expressed	YPS1	YaPSin
YFL038C	Over expressed	YPT1	Yeast Protein Two
YOR162C	Over expressed	YRR1	Yeast Reveromycin-A Resistant
YLR277C	Over expressed	YSH1	Yeast Seventy-three Homolog
YGR270W	Over expressed	YTA7	Yeast Tat-binding Analog
YMR273C	Over expressed	ZDS1	Zillion Different Screens
YKL175W	Over expressed	ZRT3	Zinc-Regulated Transporter
ARA14	Over expressed		
YAL064W-B	Over expressed		
YBR197C	Over expressed		
YBR219C	Over expressed		
YBR220C	Over expressed		

YBR284W	Over expressed		
YCR101C	Over expressed		
YDL012C	Over expressed		
YDL124W	Over expressed		
YDR018C	Over expressed		
YDR034C-D	Over expressed		
YDR034W-B	Over expressed		
YDR131C	Over expressed		
YDR415C	Over expressed		
YDR433W	Over expressed		
YEL043W	Over expressed		
YER010C	Over expressed		
YER034W	Over expressed		
YER079W	Over expressed		
YER134C	Over expressed		
YER156C	Over expressed		
YER181C	Over expressed		
YFL051C	Over expressed		
YFR016C	Over expressed		
YFR018C	Over expressed		
YGL036W	Over expressed		
YGL082W	Over expressed		
YGL108C	Over expressed		
YGL204C	Over expressed		
YGR064W	Over expressed		
YGR122W	Over expressed		
YGR125W	Over expressed		
YGR151C	Over expressed		
YHL044W	Over expressed		
YHR022C	Over expressed		
YHR033W	Over expressed		
YHR173C	Over expressed		
YIL108W	Over expressed		
YKL023W	Over expressed		
YLR311C	Over expressed		
YML119W	Over expressed		
YNR048W	Over expressed		
YNR066C	Over expressed		
YOL162W	Over expressed		
YOR238W	Over expressed		
YOR296W	Over expressed		
YPL150W	Over expressed		
YPL229W	Over expressed		
YPL264C	Over expressed		
YPR003C	Over expressed		

YPR158C-C	Over expressed		
EMPTY	Over expressed		
Q0143	Over expressed		
YAL012W-R	Over expressed		
YAR044W	Over expressed		
YBL109W	Over expressed		
YBR116C	Over expressed		
YBR292C	Over expressed		
YCL006C	Over expressed		
YCR001W	Over expressed		
YCR022C	Over expressed		
YCR099C	Over expressed		
YCR100C	Over expressed		
YDL026W	Over expressed		
YDL032W	Over expressed		
YDL172C	Over expressed		
YDL221W	Over expressed		
YDR042C	Over expressed		
YDR054C-R	Over expressed		
YDR124W	Over expressed		
YDR187C	Over expressed		
YDR215C	Over expressed		
YDR230W	Over expressed		
YDR344C	Over expressed		
YDR401W	Over expressed		
YDR413C	Over expressed		
YDR455C	Over expressed		
YDR491C	Over expressed		
YEL014C	Over expressed		
YEL023C	Over expressed		
YEL036C-R	Over expressed		
YEL073C	Over expressed		
YER084W	Over expressed		
YER130C	Over expressed		
YER187W-A	Over expressed		
YFL015C	Over expressed		
YGL052W	Over expressed		
YGL074C	Over expressed		
YGL117W	Over expressed		
YGL132W	Over expressed		
YGL177W	Over expressed		
YGL182C	Over expressed		
YGL193C	Over expressed		
YGL199C	Over expressed		
YGR018C	Over expressed		

YGR079W	Over expressed		
YGR107W	Over expressed		
YGR114C	Over expressed		
YGR137W	Over expressed		
YGR190C	Over expressed		
YGR259C	Over expressed		
YHL005C	Over expressed		
YHR214W-A	Over expressed		
YHR217C	Over expressed		
YIL032C	Over expressed		
YIL130W-R	Over expressed		
YIL152W	Over expressed		
YJR037W	Over expressed		
YJR038C	Over expressed		
YJR114W	Over expressed		
YJR115W	Over expressed		
YJR157W	Over expressed		
YKL202W	Over expressed		
YKL225W	Over expressed		
YKR012C	Over expressed		
YKR032W	Over expressed		
YKR035C	Over expressed		
YKR047W	Over expressed		
YKR073C	Over expressed		
YLL020C	Over expressed		
YLL030C	Over expressed		
YLR162W	Over expressed		
YLR202C	Over expressed		
YLR230W	Over expressed		
YLR302C	Over expressed		
YLR317W	Over expressed		
YLR366W	Over expressed		
YLR390W-A-R	Over expressed		
YLR437C-R	Over expressed		
YML010C-B	Over expressed		
YML089C	Over expressed		
YMR153C-A	Over expressed		
YMR172C-A	Over expressed		
YMR206W	Over expressed		
YMR304C-A	Over expressed		
YMR317W	Over expressed		
YMR320W	Over expressed		
YNL034W	Over expressed		
YNL190W-R	Over expressed		
YNL228W	Over expressed		

YNL338W-R	Over expressed		
YNR042W	Over expressed		
YOL035C	Over expressed		
YOL099C	Over expressed		
YOL123W-R	Over expressed		
YOR024W	Over expressed		
YOR072W	Over expressed		
YOR105W	Over expressed		
YOR146W	Over expressed		
YOR186W	Over expressed		
YOR218C	Over expressed		
YOR235W	Over expressed		
YOR268C	Over expressed		
YPL035C	Over expressed		
YPL062W	Over expressed		
YPL114W	Over expressed		
YPL136W	Over expressed		
YPL182C	Over expressed		
YPL261C	Over expressed		
YPL278C	Over expressed		
YPR050C	Over expressed		
YPR126C	Over expressed		
YPR150W	Over expressed		
YBL015W	Down expressed	ACH1	Acetyl CoA Hydrolase
YLL063C	Down expressed	AYT1	AcetYlTransferase
YNL059C	Down expressed	ARP5	Actin-Related Protein
YGL071W	Down expressed	AFT1	Activator of Ferrous Transport
YKL192C	Down expressed	ACP1	Acyl Carrier Protein
YCR048W	Down expressed	ARE1	Acyl-coenzyme A: cholesterol acyl transferase-Related Enzyme
YOR023C	Down expressed	AHC1	Ada Histone acetyltransferase complex Component
YMR300C	Down expressed	ADE4	ADEnine requiring
YDR111C	Down expressed	ALT2	ALanine Transaminase
YBR145W	Down expressed	ADH5	Alcohol DeHydrogenase
YDR148C	Down expressed	KGD2	alpha-KetoGlutarate Dehydrogenase
YER093C-A	Down expressed	AIM11	Altered Inheritance rate of Mitochondria
YIR003W	Down expressed	AIM21	Altered Inheritance rate of Mitochondria
YFL050C	Down expressed	ALR2	ALuminum Resistance
YNL159C	Down expressed	ASI2	Amino acid Sensor-Independent
YOR378W	Down expressed	AMF1	AMmonium Facilitator
YDL008W	Down expressed	APC11	Anaphase Promoting Complex
YNL172W	Down expressed	APC1	Anaphase Promoting Complex subunit
YBR158W	Down expressed	AMN1	Antagonist of Mitotic exit Network
YCL050C	Down expressed	APA1	AP4A phosphorylase
YMR041C	Down expressed	ARA2	ARAbinose
YDL175C	Down expressed	AIR2	Arginine methyltransferase-Interacting RING finger protein

YIL062C	Down expressed	ARC15	ARp2/3 Complex subunit
YPR200C	Down expressed	ARR2	ARsenicals Resistance
YJL180C	Down expressed	ATP12	ATP synthase
YER101C	Down expressed	AST2	ATPase STabilizing
YCR068W	Down expressed	ATG15	AuTophaGy related
YDR022C	Down expressed	ATG31	AuTophaGy related
YDL149W	Down expressed	ATG9	AuTophaGy related
YIL140W	Down expressed	AXL2	AXiaL 2 bud site selection
YLR078C	Down expressed	BOS1	Bet One Suppressor
YJL058C	Down expressed	BIT61	Binding partner of Tor2p
YPR171W	Down expressed	BSP1	Binding protein of Synaptojanin Polyphosphoinositide phosphatase domain
YPL217C	Down expressed	BMS1	BMh Sensitive
YJL020C	Down expressed	BBC1	Bni1 synthetic lethal and Bee1 (las17) Complex member
YGL220W	Down expressed	BOL2	BolA-like protein
YCR047C	Down expressed	BUD23	BUD site selection
YCR038C	Down expressed	BUD5	BUD site selection
YJL095W	Down expressed	BCK1	Bypass of C Kinase
YER167W	Down expressed	BCK2	Bypass of C Kinase
YNR069C	Down expressed	BSC5	Bypass of Stop Codon
YLR226W	Down expressed	BUR2	Bypass UAS Requirement
YAL058W	Down expressed	CNE1	CalNExin and calreticulin homolog
YJR109C	Down expressed	CPA2	Carbamyl Phosphate synthetase A
YMR231W	Down expressed	PEP5	carboxyPEPtidase Y-deficient
YDL142C	Down expressed	CRD1	CaRDiolipin synthase
YPL111W	Down expressed	CAR1	Catabolism of ARginine
YGR134W	Down expressed	CAF130	CCR4 Associated Factor
YLR178C	Down expressed	TFS1	cdc Twenty-Five Suppressor
YLR215C	Down expressed	CDC123	Cell Division Cycle
YBL084C	Down expressed	CDC27	Cell Division Cycle
YBR160W	Down expressed	CDC28	Cell Division Cycle
YLR314C	Down expressed	CDC3	Cell Division Cycle
YDR364C	Down expressed	CDC40	Cell Division Cycle
YGL190C	Down expressed	CDC55	Cell Division Cycle
YBL016W	Down expressed	FUS3	cell FUSion
YDL156W	Down expressed	CMR1	Changed Mutation Rate
YJL014W	Down expressed	CCT3	Chaperonin Containing TCP-1
YJL008C	Down expressed	CCT8	Chaperonin Containing TCP-1
YNL192W	Down expressed	CHS1	CHitin Synthase
YDR387C	Down expressed	CIN10	Chromosome INstability
YGR218W	Down expressed	CRM1	Chromosome Region Maintenance
YMR048W	Down expressed	CSM3	Chromosome Segregation in Meiosis
YJR005W	Down expressed	APL1	clathrin Adaptor Protein complex Large chain
YLR170C	Down expressed	APS1	clathrin Associated Protein complex Small subunit
YKL088W	Down expressed	CAB3	Coenzyme A Biosynthesis
YML110C	Down expressed	COQ5	COenzyme Q

YNL286W	Down expressed	CUS2	Cold sensitive U2 snRNA Suppressor
YDR163W	Down expressed	CWC15	Complexed With Cef1p
YNL336W	Down expressed	COS1	COnserved Sequence
YGL263W	Down expressed	COS12	COnserved Sequence
YFR046C	Down expressed	CNN1	Co-purified with NNf1p
YGL110C	Down expressed	CUE3	Coupling of Ubiquitin conjugation to ER degradation
YLR117C	Down expressed	CLF1	Crooked neck-Like Factor
YPL181W	Down expressed	CTI6	Cyc8-Tup1 Interacting protein
YNR028W	Down expressed	CPR8	Cyclosporin-sensitive Proline Rotamase
YAL012W	Down expressed	CYS3	CYStathionine gamma-lyase
YML054C	Down expressed	CYB2	CYtochrome B
YGL191W	Down expressed	COX13	Cytochrome c OXidase
YLL018C-A	Down expressed	COX19	Cytochrome c OXidase
YLR395C	Down expressed	COX8	Cytochrome c OXidase
YDL117W	Down expressed	CYK3	CYtoKinesis
YML086C	Down expressed	ALO1	D-Arabinono-1,4-Lactone Oxidase
YER124C	Down expressed	DSE1	Daughter Specific Expression
YLR276C	Down expressed	DBP9	Dead Box Protein
YBR281C	Down expressed	DUG2	Deficient in Utilization of Glutathione
YIR028W	Down expressed	DAL4	Degradation of Allantoin
YNR040W	Down expressed	DPI29	Delta-Psi dependent mitochondrial Import protein of 29 kDa
YOR311C	Down expressed	DGK1	DiacylGlycerol Kinase
YML080W	Down expressed	DUS1	DihydroUridine Synthase
YLR405W	Down expressed	DUS4	DihydroUridine Synthase
YKL191W	Down expressed	DPH2	DiPHthamide
YLR172C	Down expressed	DPH5	DiPHthamide
YDL174C	Down expressed	DLD1	D-Lactate Dehydrogenase
YIR004W	Down expressed	DJP1	DnaJ Protein
YLL057C	Down expressed	JLP1	dnaJ-Like Protein
YJL065C	Down expressed	DLS1	Dpb3-Like Subunit of ISW2/yCHRAC complex
YOL087C	Down expressed	DUF1	DUB-associated Factor 1
YKR083C	Down expressed	DAD2	Duo1 And Dam1 interacting
YAL003W	Down expressed	EFB1	Elongation Factor Beta
YBR271W	Down expressed	EFM2	Elongation Factor Methyltransferase
YIL064W	Down expressed	EFM4	Elongation Factor Methyltransferase
YMR312W	Down expressed	ELP6	ELongator Protein
YDL018C	Down expressed	ERP3	Emp24p/Erv25p Related Protein
YOL043C	Down expressed	NTG2	eNdonuclease Three-like Glycosylase
YMR171C	Down expressed	EAR1	Endosomal Adaptor of Rsp5p
YFL024C	Down expressed	EPL1	Enhancer of Polycomb Like
YMR323W	Down expressed	ERR3	Enolase-Related Repeat
YGL231C	Down expressed	EMC4	ER Membrane protein Complex
YIL027C	Down expressed	EMC5	ER Membrane protein Complex
YML012W	Down expressed	ERV25	ER Vesicle
YHR007C	Down expressed	ERG11	ERGosterol biosynthesis

YMR208W	Down expressed	ERG12	ERGosterol biosynthesis
YGR015C	Down expressed	EAT1	Ethanol AcetylTransferase
YHL010C	Down expressed	ETP1	Ethanol Tolerance Protein
YNR034W-A	Down expressed	EGO4	Exit from rapamycin-induced GrOwth arrest
YDR261C	Down expressed	EXG2	EXo-1,3-beta-Glucanase
YJL085W	Down expressed	EXO70	EXOcyst
YBR033W	Down expressed	EDS1	Expression Dependent on Slt2
YBL001C	Down expressed	ECM15	ExtraCellular Mutant
YMR128W	Down expressed	ECM16	ExtraCellular Mutant
YBL101C	Down expressed	ECM21	ExtraCellular Mutant
YJL201W	Down expressed	ECM25	ExtraCellular Mutant
YDL166C	Down expressed	FAP7	Factor Activating Pos9
YJL157C	Down expressed	FAR1	Factor ARrest
YMR052W	Down expressed	FAR3	Factor ARrest
YBR101C	Down expressed	FES1	Factor Exchange for Ssa1p
YBR040W	Down expressed	FIG1	Factor-Induced Gene
YDR339C	Down expressed	FCF1	Faf1p Copurifying Factor
YHR049W	Down expressed	FSH1	Family of Serine Hydrolases
YER015W	Down expressed	FAA2	Fatty Acid Activation
YDR519W	Down expressed	FPR2	FKBP Proline Rotamase
YNL023C	Down expressed	FAP1	FKBP12-Associated Protein
YER109C	Down expressed	FLO8	FLOcculation
YNL068C	Down expressed	FKH2	ForK head Homolog
YGR052W	Down expressed	FMP48	Found in Mitochondrial Proteome
YEL047C	Down expressed	FRD1	Fumarate ReDuctase
YNL133C	Down expressed	FYV6	Function required for Yeast Viability
YAL028W	Down expressed	FRT2	Functionally Related to TCP1
YBR179C	Down expressed	FZO1	FuZzy Onions homolog
YHR005C	Down expressed	GPA1	G Protein Alpha subunit
YLR081W	Down expressed	GAL2	GALactose metabolism
YER163C	Down expressed	GCG1	Gamma-glutamyl Cyclotransferase acting on Glutathione
YOR260W	Down expressed	GCD1	General Control Derepressed
YJL125C	Down expressed	GCD14	General Control Nonderepressible
YDR283C	Down expressed	GCN2	General Control Nonderepressible
YEL009C	Down expressed	GCN4	General Control Nonderepressible
YNL255C	Down expressed	GIS2	GIg Suppressor
YER107C	Down expressed	GLE2	GLFG LEthal
YCL040W	Down expressed	GLK1	GLucoKinase
YHR074W	Down expressed	QNS1	glutamine (Q) dependent Nad+ Synthetase
YPL059W	Down expressed	GRX5	GlutaRedoXin
YBR014C	Down expressed	GRX7	GlutaRedoXin
YJL101C	Down expressed	GSH1	glutathione (GSH)
YMR251W	Down expressed	GTO3	Glutathione Transferase Omega-like
YIL053W	Down expressed	GPP1	Glycerol-3-Phosphate Phosphatase
YLR343W	Down expressed	GAS2	Glycophospholipid-Anchored Surface protein

YGL142C	Down expressed	GPI10	GlycosylPhosphatidylInositol anchor biosynthesis
YJR013W	Down expressed	GPI14	GlycosylPhosphatidylInositol anchor biosynthesis
YJL091C	Down expressed	GWT1	GPI-anchored Wall protein Transfer
YLR289W	Down expressed	GUF1	Gtpase of Unknown Function
YMR217W	Down expressed	GUA1	GUanine Auxotroph
YER083C	Down expressed	GET2	Guided Entry of Tail-anchored proteins
YJL165C	Down expressed	HAL5	HALotolerance
YPR068C	Down expressed	HOS1	Hda One Similar
YGL040C	Down expressed	HEM2	HEMe biosynthesis
YCL025C	Down expressed	AGP1	high-Affinity Glutamine Permease
YFL055W	Down expressed	AGP3	high-Affinity Glutamine Permease
YBR248C	Down expressed	HIS7	HIStidine requiring
YBL003C	Down expressed	HTA2	Histone h Two A
YBL002W	Down expressed	HTB2	Histone h Two B
YBL008W	Down expressed	HIR1	HIstone Regulation
YBR034C	Down expressed	HMT1	HnRNP MethylTransferase
YOL021C	Down expressed	DIS3	homolog of S. pombe dis3 (chromosome DISjunction)
YGL056C	Down expressed	SDS23	homolog of S. pombe SDS23
YDR291W	Down expressed	HRQ1	Homologous to RecQ protein
YDL227C	Down expressed	HO	HOmothallic switching endonuclease
YJL146W	Down expressed	IDS2	IME2-Dependent Signaling
YBR107C	Down expressed	IML3	Increased Minichromosome Loss
YKL117W	Down expressed	SBA1	increased Sensitivity to Benzoquinone Ansamycins
YOL023W	Down expressed	IFM1	Initiation Factor of Mitochondria
YNL215W	Down expressed	IES2	Ino Eighty Subunit
YOL065C	Down expressed	INP54	INositol polyphosphate 5-Phosphatase
YDR315C	Down expressed	IPK1	Inositol Polyphosphate Kinase
YHR079C	Down expressed	IRE1	Inositol REquiring
YER078C	Down expressed	ICP55	Intermediate Cleaving Peptidase
YKL032C	Down expressed	IXR1	Intrastrand cross (X)-link Recognition
YBL076C	Down expressed	ILS1	IsoLeucine-tRNA Synthetase
YMR044W	Down expressed	IOC4	Iswi One Complex
YJR119C	Down expressed	JHD2	JmjC domain-containing Histone Demethylase
YJL094C	Down expressed	KHA1	K/H ion Antiporter
YPR159W	Down expressed	KRE6	Killer toxin REsistant
YGL216W	Down expressed	KIP3	KInesin related Protein
YMR298W	Down expressed	LIP1	Lag1p/Lac1p Interacting Protein
YGR136W	Down expressed	LSB1	Las Seventeen Binding protein
YOR246C	Down expressed	ENV9	late ENdosome and Vacuole interface function
YNL323W	Down expressed	LEM3	Ligand-Effect Modulator
YJL124C	Down expressed	LSM1	Like SM
YNL147W	Down expressed	LSM7	Like SM
YMR147W	Down expressed	LDO45	Lipid Droplet Organization protein of 45 Kda
YDR503C	Down expressed	LPP1	Lipid Phosphate Phosphatase
YLR072W	Down expressed	LAM6	Lipid transfer protein Anchored at Membrane contact site

YOR196C	Down expressed	LIP5	LIPoic acid
YCL005W	Down expressed	LDB16	Low Dye Binding
YJR002W	Down expressed	MPP10	M Phase Phosphoproteins
YKL021C	Down expressed	MAK11	MAintenance of Killer
YBR142W	Down expressed	MAK5	MAintenance of Killer
YHR204W	Down expressed	MNL1	MaNnosidase-Like protein
YIL014W	Down expressed	MNT3	MaNnosylTransferase
YCR040W	Down expressed	MATALPHA 1	MATing type protein ALPHA
YOL135C	Down expressed	MED7	MEDiator complex
YCL061C	Down expressed	MRC1	Mediator of the Replication Checkpoint
YFL034W	Down expressed	MIL1	Medium adaptin-Interacting Ligand
YPL121C	Down expressed	MEI5	MEIosis
YDR205W	Down expressed	MSC2	Meiotic Sister-Chromatid recombination
YOR354C	Down expressed	MSC6	Meiotic Sister-Chromatid recombination
YJR008W	Down expressed	MHO1	Memo HOmolog
YPR019W	Down expressed	MCM4	MiniChromosome Maintenance
YIL070C	Down expressed	MAM33	Mitochondrial Acidic Matrix protein
YNL073W	Down expressed	MSK1	Mitochondrial aminoacyl-tRNA Synthetase, lysine (K)
YGR171C	Down expressed	MSM1	Mitochondrial aminoacyl-tRNA Synthetase, Methionine
YHR024C	Down expressed	MAS2	Mitochondrial ASsembly
YGR235C	Down expressed	MIC26	MItochondrial contact site and Cristae organizing system
YML104C	Down expressed	MDM1	Mitochondrial Distribution and Morphology
YHR194W	Down expressed	MDM31	Mitochondrial Distribution and Morphology
YOL027C	Down expressed	MDM38	Mitochondrial Distribution and Morphology
YFL016C	Down expressed	MDJ1	Mitochondrial DnaJ
YLR069C	Down expressed	MEF1	Mitochondrial Elongation Factor
YKL195W	Down expressed	MIA40	Mitochondrial intermembrane space Import and Assembly
YMR166C	Down expressed	MME1	Mitochondrial Magnesium Exporter
YNL211C	Down expressed	MRX7	Mitochondrial oRganization of gene eXpression (MIOREX)
YDR347W	Down expressed	MRP1	Mitochondrial Ribosomal Protein
YBL090W	Down expressed	MRP21	Mitochondrial Ribosomal Protein
YNL284C	Down expressed	MRPL10	Mitochondrial Ribosomal Protein, Large subunit
YJL096W	Down expressed	MRPL49	Mitochondrial Ribosomal Protein, Large subunit
YJL063C	Down expressed	MRPL8	Mitochondrial Ribosomal Protein, Large subunit
YBR146W	Down expressed	MRPS9	Mitochondrial Ribosomal Protein, Small subunit
YMR023C	Down expressed	MSS1	Mitochondrial Splicing System
YGL236C	Down expressed	MTO1	Mitochondrial Translation Optimization
YGL068W	Down expressed	MNP1	Mitochondrial-Nucleoid Protein
YGL124C	Down expressed	MON1	MONensin sensitivity
YNL118C	Down expressed	DCP2	mRNA DeCaPping
YPL060W	Down expressed	MFM1	Mrs2 Function Modulating factor
YBR057C	Down expressed	MUM2	MUddled Meiosis
YBR185C	Down expressed	MBA1	Multi-copy Bypass of AFG3
YGR014W	Down expressed	MSB2	Multicopy Suppression of a Budding defect

YAR033W	Down expressed	MST28	Multicopy suppressor of Sec Twenty one
YMR004W	Down expressed	MVP1	Multi-copy suppressor of vps1
YPL184C	Down expressed	MRN1	Multicopy supressor of rsc nhp6
YBR119W	Down expressed	MUD1	Mutant U1 Die
YOL103W	Down expressed	ITR2	myo-Inositol TRansporter
YIL149C	Down expressed	MLP2	Myosin-Like Protein
YGL221C	Down expressed	NIF3	Ngg1p-Interacting Factor
YDL046W	Down expressed	NPC2	Niemann Pick type C homolog
YCL017C	Down expressed	NFS1	NiFS-like
YDL002C	Down expressed	NHP10	Non-Histone Protein
YDR150W	Down expressed	NUM1	NUclear Migration
YJR042W	Down expressed	NUP85	NUclear Pore
YJR072C	Down expressed	NPA3	Nucleolar Preribosomal Associated
YNL175C	Down expressed	NOP13	NucleOlar Protein
YOR103C	Down expressed	OST2	OligoSaccharylTransferase
YOR085W	Down expressed	OST3	OligoSaccharylTransferase
YPR075C	Down expressed	OPY2	Overproduction-induced Pheromone-resistant Yeast
YDL019C	Down expressed	OSH2	OxySterol binding protein Homolog
YHR073W	Down expressed	OSH3	OxySterol binding protein Homolog
YHR063C	Down expressed	PAN5	PANtothenate biosynthesis
YGR078C	Down expressed	PAC10	Perish in the Absence of Cin8p
YKL197C	Down expressed	PEX1	PEroXin
YHR160C	Down expressed	PEX18	PEroXin
YPL147W	Down expressed	PXA1	PeroXisomal ABC-transporter
YGL153W	Down expressed	PEX14	PEroXisome related
YOL044W	Down expressed	PEX15	PEroXisome related
YHR150W	Down expressed	PEX28	PEroXisome related
YGR004W	Down expressed	PEX31	PEroXisome related
YNL279W	Down expressed	PRM1	Pheromone-Regulated Membrane protein
YHR071W	Down expressed	PCL5	Pho85 CycLin
YDR183W	Down expressed	PLP1	Phosducin-Like Protein
YBL056W	Down expressed	PTC3	Phosphatase Two C
YOR360C	Down expressed	PDE2	PhosphoDiEsterase
YKL128C	Down expressed	PMU1	PhosphoMUtase
YLR376C	Down expressed	PSY3	Platinum SensitivitY
YGL013C	Down expressed	PDR1	Pleiotropic Drug Resistance
YNL264C	Down expressed	PDR17	Pleiotropic Drug Resistance
YBL005W	Down expressed	PDR3	Pleiotropic Drug Resistance
YGL094C	Down expressed	PAN2	Poly(A)-binding protein-dependent poly(A) riboNuclease
YJR006W	Down expressed	POL31	POLymerase
YMR129W	Down expressed	POM152	POre Membrane
YLR016C	Down expressed	PML1	Pre-mRNA Leakage
YPR178W	Down expressed	PRP4	Pre-mRNA Processing
YJL104W	Down expressed	PAM16	Presequence translocase-Associated Motor
YLR008C	Down expressed	PAM18	Presequence translocase-Associated Motor

YAL043C	Down expressed	PTA1	Pre-Trna Accumulation
YML125C	Down expressed	PGA3	Processing of Gas1p and ALP
YOR122C	Down expressed	PFY1	ProFilin of Yeast
YGR132C	Down expressed	PHB1	ProHiBitin
YIL042C	Down expressed	PKP1	Protein Kinase of PDH
YDR435C	Down expressed	PPM1	Protein Phosphatase Methyltransferase
YOR361C	Down expressed	PRT1	PRoTein synthesis
YJL001W	Down expressed	PRE3	PRoteinase yscE
YBR188C	Down expressed	NTC20	Prp19p (NineTeen)-associated Complex
YDL036C	Down expressed	PUS9	PseudoUridine Synthase
YOR104W	Down expressed	PIN2	Psi+ INducibility
YBR295W	Down expressed	PCA1	P-type Cation-transporting ATPase
YLR209C	Down expressed	PNP1	purine nucleoside phosphorylase
YKL198C	Down expressed	PTK1	Putative serine/Threonine protein Kinase
YPR186C	Down expressed	PZF1	Putative Zinc Finger
YBR035C	Down expressed	PDX3	PyriDoXine auxotrophy
YGL062W	Down expressed	PYC1	PYruvate Carboxylase
YBR221C	Down expressed	PDB1	Pyruvate Dehydrogenase Beta subunit
YGR193C	Down expressed	PDX1	Pyruvate Dehydrogenase complex protein X
YBR043C	Down expressed	QDR3	QuiniDine Resistance
YBR073W	Down expressed	RDH54	RaD54 Homolog
YBR114W	Down expressed	RAD16	RADiation sensitive
YDR076W	Down expressed	RAD55	RADiation sensitive
YMR235C	Down expressed	RNA1	rapid cessation of net RNA accumulation
YKL019W	Down expressed	RAM2	RAS protein and A-factor Maturation
YJL204C	Down expressed	RCY1	ReCYcling
YNL330C	Down expressed	RPD3	Reduced Potassium Dependency
YDR388W	Down expressed	RVS167	Reduced Viability on Starvation
YBL093C	Down expressed	ROX3	Regulation by OXygen
YJL217W	Down expressed	REE1	REgulation of Enolase
YMR034C	Down expressed	RCH1	Regulator of Calcium Homeostasis
YLR073C	Down expressed	RFU1	Regulator of Free Ubiquitin chains
YMR139W	Down expressed	RIM11	Regulator of IME2
YPR115W	Down expressed	RGC1	Regulator of the Glycerol Channel
YJL047C	Down expressed	RTT101	Regulator of Ty1 Transposition
YLR176C	Down expressed	RFX1	Regulatory Factor X
YOR261C	Down expressed	RPN8	Regulatory Particle Non-ATPase
YBR192W	Down expressed	RIM2	Replication In Mitochondria
YBR050C	Down expressed	REG2	REsistance to Glucose repression
YJL026W	Down expressed	RNR2	RiboNucleotide Reductase
YGR180C	Down expressed	RNR4	RiboNucleotide Reductase
YBR030W	Down expressed	RKM3	Ribosomal lysine (K) Methyltransferase
YMR121C	Down expressed	RPL15B	Ribosomal Protein of the Large subunit
YBL027W	Down expressed	RPL19B	Ribosomal Protein of the Large subunit
YKR094C	Down expressed	RPL40B	Ribosomal Protein of the Large subunit

YBR031W	Down expressed	RPL4A	Ribosomal Protein of the Large subunit
YGL147C	Down expressed	RPL9A	Ribosomal Protein of the Large subunit
YNL067W	Down expressed	RPL9B	Ribosomal Protein of the Large subunit
YLR048W	Down expressed	RPS0B	Ribosomal Protein of the Small subunit
YCR031C	Down expressed	RPS14A	Ribosomal Protein of the Small subunit
YOL040C	Down expressed	RPS15	Ribosomal Protein of the Small subunit
YML024W	Down expressed	RPS17A	Ribosomal Protein of the Small subunit
YML063W	Down expressed	RPS1B	Ribosomal Protein of the Small subunit
YJL136C	Down expressed	RPS21B	Ribosomal Protein of the Small subunit
YGR118W	Down expressed	RPS23A	Ribosomal Protein of the Small subunit
YGR027C	Down expressed	RPS25A	Ribosomal Protein of the Small subunit
YOR167C	Down expressed	RPS28A	Ribosomal Protein of the Small subunit
YPL090C	Down expressed	RPS6A	Ribosomal Protein of the Small subunit
YER102W	Down expressed	RPS8B	Ribosomal Protein of the Small subunit
YCR035C	Down expressed	RRP43	Ribosomal RNA Processing
YNR037C	Down expressed	RSM19	Ribosomal Small subunit of Mitochondria
YGR215W	Down expressed	RSM27	Ribosomal Small subunit of Mitochondria
YJR113C	Down expressed	RSM7	Ribosomal Small subunit of Mitochondria
YNL163C	Down expressed	RIA1	RIbosome Assembly
YOL005C	Down expressed	RPB11	RNA Polymerase B
YJL140W	Down expressed	RPB4	RNA Polymerase B
YOR224C	Down expressed	RPB8	RNA Polymerase B
YDL150W	Down expressed	RPC53	RNA Polymerase C
YBR049C	Down expressed	REB1	RNA polymerase I Enhancer Binding protein
YGR276C	Down expressed	RNH70	RNase H
YDR091C	Down expressed	RLI1	RNase L Inhibitor
YJL080C	Down expressed	SCP160	S. cerevisiae protein involved in the Control of Ploidy
YBL050W	Down expressed	SEC17	SECretory
YBR080C	Down expressed	SEC18	SECretory
YIL076W	Down expressed	SEC28	SECretory
YIL068C	Down expressed	SEC6	SECretory
YPL094C	Down expressed	SEC62	SECretory
YOR254C	Down expressed	SEC63	SECretory
YGR009C	Down expressed	SEC9	SECretory
YKR043C	Down expressed	SHB17	SedoHeptulose 1,7-Bisphosphatase
YGL228W	Down expressed	SHE10	Sensitivity to High Expression
YJL151C	Down expressed	SNA3	Sensitivity to NA+
YHL046C	Down expressed	PAU13	seriPAUperin
YHR119W	Down expressed	SET1	SET domain-containing
YDL225W	Down expressed	SHS1	Seventh Homolog of Septin
YLR066W	Down expressed	SPC3	Signal Peptidase Complex
YLR442C	Down expressed	SIR3	Silent Information Regulator
YNL066W	Down expressed	SUN4	Sim1 Uth1 Nca3
YHR178W	Down expressed	STB5	Sin Three Binding protein
YKL072W	Down expressed	STB6	Sin Three Binding protein

YJL098W	Down expressed	SAP185	Sit4 Associated Protein
YGL229C	Down expressed	SAP4	Sit4 Associated Protein
YLR321C	Down expressed	SFH1	Snf Five Homolog
YJL089W	Down expressed	SIP4	SNF1-Interacting Protein
YMR096W	Down expressed	SNZ1	SNooZe
YMR095C	Down expressed	SNO1	SNZ proximal Open reading frame
YJL036W	Down expressed	SNX4	Sorting NeXin
YPR069C	Down expressed	SPE3	SPErmidine auxotroph
YLL021W	Down expressed	SPA2	Spindle Pole Antigen
YKL042W	Down expressed	SPC42	Spindle Pole Component
YHR152W	Down expressed	SPO12	SPOrulation
YOL091W	Down expressed	SPO21	SPOrulation
YBR250W	Down expressed	SPO23	SPOrulation
YDR218C	Down expressed	SPR28	SPorulation Regulated
YGR059W	Down expressed	SPR3	SPorulation Regulated
YHR139C	Down expressed	SPS100	SPorulation Specific
YBR106W	Down expressed	SND3	SRP-iNDependent targeting
YDR410C	Down expressed	STE14	STErile
YJR086W	Down expressed	STE18	STErile
YOR027W	Down expressed	STI1	STress Inducible
YLR086W	Down expressed	SMC4	Structural Maintenance of Chromosomes
YLL041C	Down expressed	SDH2	Succinate DeHydrogenase
YDR511W	Down expressed	SDH7	Succinate DeHydrogenase
YDR536W	Down expressed	STL1	Sugar Transporter-Like protein
YPR133C	Down expressed	SPN1	Suppresses Postrecruitment functions gene Number 1
YPR198W	Down expressed	SGE1	Suppression of Gal11 Expression
YBR143C	Down expressed	SUP45	SUPpressor
YDL084W	Down expressed	SUB2	SUppressor of Brr1-1
YLL016W	Down expressed	SDC25	Suppressor of cdc25
YGL224C	Down expressed	SDT1	Suppressor of Disruption of TFIIS
YBL058W	Down expressed	SHP1	Suppressor of High-copy PP1
YJR007W	Down expressed	SUI2	SUppressor of Initiator codon
YBR253W	Down expressed	SRB6	Suppressor of RNA polymerase B
YKL006C-A	Down expressed	SFT1	Suppressor of sed Five Ts
YLR006C	Down expressed	SSK1	Suppressor of Sensor Kinase
YNL139C	Down expressed	THO2	suppressor of the Transcriptional defect of Hpr1 by Overexpression
YER161C	Down expressed	SPT2	SuPpressor of Ty's
YBR081C	Down expressed	SPT7	SuPpressor of Ty's
YMR183C	Down expressed	SSO2	Supressor of Sec One
YDR146C	Down expressed	SWI5	SWItching deficient
YPR101W	Down expressed	SNT309	Synthetic lethal to prp NineTeen mutation
YBL007C	Down expressed	SLA1	Synthetic Lethal with ABP1
YGR129W	Down expressed	SYF2	SYnthetic lethal with cdc40 (Forty)
YBR077C	Down expressed	SLM4	Synthetic Lethal with Mss4
YCR024C	Down expressed	SLM5	Synthetic Lethal with Mss4

YNL187W	Down expressed	SWT21	Synthetic With Tgs1
YBR156C	Down expressed	SLI15	Synthetically Lethal with Ipl1
YKL166C	Down expressed	TPK3	Takashi's Protein Kinase
YCR042C	Down expressed	TAF2	TATA binding protein-Associated Factor
YMR005W	Down expressed	TAF4	TATA binding protein-Associated Factor
YBL088C	Down expressed	TEL1	TELomere maintenance
YBR265W	Down expressed	TSC10	Temperature-sensitive Suppressors of Csg2 mutants
YNL128W	Down expressed	TEP1	TEnsin-like Phosphatase
YMR277W	Down expressed	FCP1	tfiiF-associating component of Ctd Phosphatase
YPL214C	Down expressed	THI6	THIamine biosynthesis
YPR121W	Down expressed	THI22	THIamine metabolism
YCR053W	Down expressed	THR4	THReonine requiring
YIL011W	Down expressed	TIR3	TIp1-Related
YLR234W	Down expressed	TOP3	TOPoisomerase
YCR060W	Down expressed	TAH1	Tpr-containing protein Associated with Hsp90
YGR005C	Down expressed	TFG2	Transcription Factor G
YBR061C	Down expressed	TRM7	Transfer RNA Methyltransferase
YJL138C	Down expressed	TIF2	Translation Initiation Factor
YPR016C	Down expressed	TIF6	Translation Initiation Factor
YER007C-A	Down expressed	TMA20	Translation Machinery Associated
YJL143W	Down expressed	TIM17	Translocase of the Inner Mitochondrial membrane
YJL054W	Down expressed	TIM54	Translocase of the Inner Mitochondrial membrane
YHR117W	Down expressed	TOM71	Translocase of the Outer Mitochondrial membrane
YKL174C	Down expressed	TPO5	Transporter of POlyamines
YMR313C	Down expressed	TGL3	TriacylGlycerol Lipase
YBR044C	Down expressed	TCM62	TriCarboxylic acid cycle Mutant
YGR024C	Down expressed	THG1	tRNAHis Guanylyltransferase
YKR079C	Down expressed	TRZ1	tRNase Z
YIL138C	Down expressed	TPM2	TroPoMyosin
YDR007W	Down expressed	TRP1	TRyPtophan
YDR354W	Down expressed	TRP4	TRyPtophan
YOL020W	Down expressed	TAT2	Tryptophan Amino acid Transporter
YOL018C	Down expressed	TLG2	T-snare affecting a Late Golgi compartment
YML085C	Down expressed	TUB1	TUBulin
YMR028W	Down expressed	TAP42	Two A phosphatase Associated Protein
YGR185C	Down expressed	TYS1	TYrosyl-tRNA Synthetase
YJL069C	Down expressed	UTP18	U Three Protein
YHR196W	Down expressed	UTP9	U Three Protein
YBR273C	Down expressed	UBX7	UBiquitin regulatory X
YDR177W	Down expressed	UBC1	UBiquitin-Conjugating
YBL067C	Down expressed	UBP13	UBiquitin-specific Protease
YBR058C	Down expressed	UBP14	UBiquitin-specific Protease
YOR124C	Down expressed	UBP2	UBiquitin-specific Protease
YFR010W	Down expressed	UBP6	UBiquitin-specific Protease
YIL156W	Down expressed	UBP7	UBiquitin-specific Protease

YAR027W	Down expressed	UIP3	Ulp1 Interacting Protein
YPL230W	Down expressed	USV1	Up in StarVation
YDL170W	Down expressed	UGA3	Utilization of GAba
YNL212W	Down expressed	VID27	Vacuolar Import and Degradation
YGR020C	Down expressed	VMA7	Vacuolar Membrane Atpase
YHR012W	Down expressed	VPS29	Vacuolar Protein Sorting
YJL154C	Down expressed	VPS35	Vacuolar Protein Sorting
YER072W	Down expressed	VTC1	Vacuolar Transporter Chaperone
YJL222W	Down expressed	VTH2	Vps Ten Homolog
YMR197C	Down expressed	VTI1	Vps10 (Ten) Interacting
YOR229W	Down expressed	WTM2	WD repeat containing Transcriptional Modulator
YDR349C	Down expressed	YPS7	YaPSin
YIR018W	Down expressed	YAP5	Yeast AP-1
YLL048C	Down expressed	YBT1	Yeast Bile Transporter
YLR249W	Down expressed	YEF3	Yeast Elongation Factor
YDL072C	Down expressed	YET3	Yeast Endoplasmic reticulum Transmembrane protein
YDR451C	Down expressed	YHP1	Yeast Homeo-Protein
YJL059W	Down expressed	YHC3	Yeast Homolog of human Cln3
YDR057W	Down expressed	YOS9	Yeast OS-9 homolog
YOR172W	Down expressed	YRM1	Yeast Reveromycin resistance Modulator
YKR053C	Down expressed	YSR3	Yeast Sphingolipid Resistance
YMR241W	Down expressed	YHM2	Yeast suppressor of HM mutant
YJR099W	Down expressed	YUH1	Yeast Ubiquitin Hydrolase
YJL141C	Down expressed	YAK1	Yet Another Kinase
Q0060	Down expressed	AI3	
YGL021W	Down expressed	ALK1	
YOR355W	Down expressed	GDS1	
YKL189W	Down expressed	HYM1	
YDL055C	Down expressed	PSA1	
YDL103C	Down expressed	QRI1	
YNL022C	Down expressed	RCM1	
YBL061C	Down expressed	SKT5	
YLR275W	Down expressed	SMD2	
YIL061C	Down expressed	SNP1	
YGL131C	Down expressed	SNT2	
YHR039C-A	Down expressed	VMA10	
YER041W	Down expressed	YEN1	
YHR048W	Down expressed	YHK8	
YGR296W	Down expressed	YRF1-3	
YLR466W	Down expressed	YRF1-4	
YBR111C	Down expressed	YSA1	
YBR148W	Down expressed	YSW1	
Q0255	Down expressed		
YAR023C	Down expressed		
YBL028C	Down expressed		

YBL036C	Down expressed		
YBR138C	Down expressed		
YCL020W	Down expressed		
YCL049C	Down expressed		
YCR043C	Down expressed		
YCR102C	Down expressed		
YDL180W	Down expressed		
YDR514C	Down expressed		
YFL066C	Down expressed		
YGL230C	Down expressed		
YGR016W	Down expressed		
YGR017W	Down expressed		
YGR237C	Down expressed		
YHL009W-A	Down expressed		
YHR020W	Down expressed		
YIL060W	Down expressed		
YJL068C	Down expressed		
YJL213W	Down expressed		
YJL218W	Down expressed		
YJR015W	Down expressed		
YJR029W	Down expressed		
YKL033W-A	Down expressed		
YKL050C	Down expressed		
YKL071W	Down expressed		
YLL066C	Down expressed		
YLL067C	Down expressed		
YLR126C	Down expressed		
YLR157C-A	Down expressed		
YLR173W	Down expressed		
YLR179C	Down expressed		
YLR287C	Down expressed		
YML079W	Down expressed		
YMR114C	Down expressed		
YNL195C	Down expressed		
YNL247W	Down expressed		
YNR029C	Down expressed		
YNR071C	Down expressed		
YOR019W	Down expressed		
YPL108W	Down expressed		
YPL216W	Down expressed		
YPR071W	Down expressed		
ARA17	Down expressed		
ARA25	Down expressed		
ARA5	Down expressed		
ARA8	Down expressed		

Q0182	Down expressed		
Q0297	Down expressed		
YAL004W	Down expressed		
YAL043C-A	Down expressed		
YAL064C-A	Down expressed		
YAR030C	Down expressed		
YAR053W	Down expressed		
YAR064W	Down expressed		
YAR068W	Down expressed		
YAR069C	Down expressed		
YBL012C	Down expressed		
YBL070C	Down expressed		
YBL101W-B	Down expressed		
YBR063C	Down expressed		
YBR150C-R	Down expressed		
YBR232C	Down expressed		
YCL023C	Down expressed		
YCL041C	Down expressed		
YDR112W	Down expressed		
YDR271C	Down expressed		
YDR442W	Down expressed		
YEL008W	Down expressed		
YER053C-A-R	Down expressed		
YER085C	Down expressed		
YER091C-A	Down expressed		
YFL032W	Down expressed		
YGR045C	Down expressed		
YHR078W	Down expressed		
YIL141W	Down expressed		
YIL174W	Down expressed		
YJL018W	Down expressed		
YJL022W	Down expressed		
YJL067W	Down expressed		
YJL105W	Down expressed		
YJL199C	Down expressed		
YKL107W	Down expressed		
YKL115C	Down expressed		
YLL044W	Down expressed		
YLR156W	Down expressed		
YLR374C	Down expressed		
YLR464W	Down expressed		
YML013C-A	Down expressed		
YML083C	Down expressed		
YMR324C	Down expressed		
YNL057W	Down expressed		

YNL184C	Down expressed		
YNR005C	Down expressed		
YOR111W	Down expressed		
YOR389W	Down expressed		
YPL251W	Down expressed		
YPR016C-R	Down expressed		
YPR053C	Down expressed		
YPR059C	Down expressed		
**Genes which expression changed in TAT12 evolved strain compared with the parental strain S288C**			
**Systematic Name**	**Expression**	**Standard Name**	**Name Description**
YKL206C	Over expressed	ADD66	Alpha1-proteinase inhibitor-Degradation Deficient
YAR015W	Over expressed	ADE1	ADEnine requiring
YGL234W	Over expressed	ADE5,7	ADEnine requiring
YOL086C	Over expressed	ADH1	Alcohol DeHydrogenase
YDR214W	Over expressed	AHA1	Activator of Heat shock protein 90 ATPase
Q0065	Over expressed	AI4	
YDL175C	Over expressed	AIR2	Arginine methyltransferase-Interacting RING finger protein
YMR169C	Over expressed	ALD3	ALdehyde Dehydrogenase
YNL219C	Over expressed	ALG9	Asparagine-Linked Glycosylation
YBL009W	Over expressed	ALK2	
YOR378W	Over expressed	AMF1	AMmonium Facilitator
YDR530C	Over expressed	APA2	AP4A phosphorylase
YKL114C	Over expressed	APN1	APurinic/apyrimidinic eNdonuclease
YGL105W	Over expressed	ARC1	Aminoacyl-tRNA synthetase Cofactor
YNR035C	Over expressed	ARC35	ARp2/3 Complex subunit
YBR164C	Over expressed	ARL1	ADP-Ribosylation factor-Like
YHL040C	Over expressed	ARN1	AFT1 ReguloN
YJR065C	Over expressed	ARP3	Actin-Related Protein
YJL081C	Over expressed	ARP4	Actin-Related Protein
YNL059C	Over expressed	ARP5	Actin-Related Protein
YMR033W	Over expressed	ARP9	Actin-Related Protein
YOR058C	Over expressed	ASE1	Anaphase Spindle Elongation
YKL185W	Over expressed	ASH1	Asymmetric Synthesis of HO
YNL159C	Over expressed	ASI2	Amino acid Sensor-Independent
YNL008C	Over expressed	ASI3	Amino acid Sensor-Independent
YFR021W	Over expressed	ATG18	AuTophaGy related
YNL242W	Over expressed	ATG2	AuTophaGy related
YDL113C	Over expressed	ATG20	AuTophaGy related

YNR007C	Over expressed	ATG3	AuTophaGy related
YDR022C	Over expressed	ATG31	AuTophaGy related
YJL185C	Over expressed	ATG36	AuTophaGy related
YOL077W-A	Over expressed	ATP19	ATP synthase
YNR020C	Over expressed	ATP23	
YPL078C	Over expressed	ATP4	ATP synthase
YJR001W	Over expressed	AVT1	Amino acid Vacuolar Transport
YER119C	Over expressed	AVT6	Amino acid Vacuolar Transport
YHR040W	Over expressed	BCD1	Box C/D snoRNA accumulation
YMR237W	Over expressed	BCH1	Bud7 and Chs6 Homolog
YKR027W	Over expressed	BCH2	Bud7 and Chs6 Homolog
YFL007W	Over expressed	BLM10	BLeoMycin resistance
YJR025C	Over expressed	BNA1	Biosynthesis of Nicotinic Acid
YGL220W	Over expressed	BOL2	BolA-like protein
YHR036W	Over expressed	BRL1	BRr6 Like protein
YGL247W	Over expressed	BRR6	Bad Response to Refrigeration
YDR275W	Over expressed	BSC2	Bypass of Stop Codon
YOL137W	Over expressed	BSC6	Bypass of Stop Codon
YGR142W	Over expressed	BTN2	BaTteN disease
YDR252W	Over expressed	BTT1	BTf Three
YLR074C	Over expressed	BUD20	BUD site selection
YLR062C	Over expressed	BUD28	BUD site selection
YJR092W	Over expressed	BUD4	BUD site selection
YLR319C	Over expressed	BUD6	BUD site selection
YML111W	Over expressed	BUL2	Binds Ubiquitin Ligase
YIL083C	Over expressed	CAB2	Coenzyme A Biosynthesis
YKL088W	Over expressed	CAB3	Coenzyme A Biosynthesis
YKR036C	Over expressed	CAF4	CCR4 Associated Factor
YFL029C	Over expressed	CAK1	Cdk-Activating Kinase
YPL111W	Over expressed	CAR1	Catabolism of ARginine
YJR060W	Over expressed	CBF1	Centromere Binding Factor
YKL011C	Over expressed	CCE1	Cruciform Cutting Endonuclease
YKR066C	Over expressed	CCP1	Cytochrome c Peroxidase
YKL022C	Over expressed	CDC16	Cell Division Cycle
YLR314C	Over expressed	CDC3	Cell Division Cycle
YLR103C	Over expressed	CDC45	Cell Division Cycle
YGL190C	Over expressed	CDC55	Cell Division Cycle
YLR418C	Over expressed	CDC73	Cell Division Cycle
YMR213W	Over expressed	CEF1	CErevisiae homolog of cdc Five

YLR098C	Over expressed	CHA4	Catabolism of Hydroxy Amino acids
YDR267C	Over expressed	CIA1	Cytosolic Iron-sulfur protein Assembly
YDR387C	Over expressed	CIN10	Chromosome INstability
YMR138W	Over expressed	CIN4	Chromosome INstability
YJL158C	Over expressed	CIS3	CIk1 Suppressing
YOR061W	Over expressed	CKA2	Casein Kinase Alpha' subunit
YGL019W	Over expressed	CKB1	Casein Kinase Beta subunit
YDL155W	Over expressed	CLB3	CycLin B
YMR012W	Over expressed	CLU1	CLUstered mitochondria
YKL137W	Over expressed	CMC1	Cx9C Mitochondrial protein necessary for full assembly of Cytochrome c oxidase
YBR109C	Over expressed	CMD1	CalMoDulin
YLR271W	Over expressed	CMG1	Cytoplasmic and Mitochondrial G-patch protein 1
YPR013C	Over expressed	CMR3	Changed Mutation Rate
YFR046C	Over expressed	CNN1	Co-purified with NNf1p
Q0105	Over expressed	COB	CytochrOme B
YGL119W	Over expressed	COQ8	COenzyme Q
YLR038C	Over expressed	COX12	Cytochrome c OXidase
YLR395C	Over expressed	COX8	Cytochrome c OXidase
YLR216C	Over expressed	CPR6	Cyclosporin-sensitive Proline Rotamase
YJR032W	Over expressed	CPR7	Cyclosporin-sensitive Proline Rotamase
YGL238W	Over expressed	CSE1	Chromosome SEgregation
YNL232W	Over expressed	CSL4	Cep1 Synthetic Lethal
YIL169C	Over expressed	CSS1	Condition Specific Secretion
YCR054C	Over expressed	CTR86	Copper TRansport protein
YKL090W	Over expressed	CUE2	Coupling of Ubiquitin conjugation to ER degradation
YMR240C	Over expressed	CUS1	Cold sensitive U2 snRNA Suppressor
YDR163W	Over expressed	CWC15	Complexed With Cef1p
YNL245C	Over expressed	CWC25	Complexed With Cef1p
YKL096W	Over expressed	CWP1	Cell Wall Protein
YML054C	Over expressed	CYB2	CYtochrome B
YKL087C	Over expressed	CYT2	CYTochrome-c1-heme-lyase
YDR016C	Over expressed	DAD1	Duo1 And Dam1 interacting
YPL119C	Over expressed	DBP1	Dead Box Protein
YKR024C	Over expressed	DBP7	Dead Box Protein
YLR276C	Over expressed	DBP9	Dead Box Protein
YOR180C	Over expressed	DCI1	delta(3,5)-delta(2,4)-Dienoyl-CoA Isomerase
YNL118C	Over expressed	DCP2	mRNA DeCaPping
YOR311C	Over expressed	DGK1	DiacylGlycerol Kinase
YOL021C	Over expressed	DIS3	homolog of S. pombe dis3 (chromosome DISjunction)

YDL174C	Over expressed	DLD1	D-Lactate Dehydrogenase
YGL240W	Over expressed	DOC1	Destruction Of Cyclin B
YLR172C	Over expressed	DPH5	DiPHthamide
YMR287C	Over expressed	DSS1	Deletion of SUV3 Supressor
YGL043W	Over expressed	DST1	DNA Strand Transfer
YLR401C	Over expressed	DUS3	DihydroUridine Synthase
YDR424C	Over expressed	DYN2	DYNein
YPR023C	Over expressed	EAF3	Esa1p-Associated Factor
YGR015C	Over expressed	EAT1	Ethanol AcetylTransferase
YDR206W	Over expressed	EBS1	Est1-like Bcy1 Suppressor
YDR446W	Over expressed	ECM11	ExtraCellular Mutant
YBL001C	Over expressed	ECM15	ExtraCellular Mutant
YNL024C	Over expressed	EFM6	Elongation Factor Methyltransferase
YIR007W	Over expressed	EGH1	Cryptococcus neoformans EGCrP2 Homolog
YNR034W-A	Over expressed	EGO4	Exit from rapamycin-induced GrOwth arrest
YKL048C	Over expressed	ELM1	ELongated Morphology
YML126C	Over expressed	ERG13	ERGosterol biosynthesis
YJL167W	Over expressed	ERG20	ERGosterol biosynthesis
YBR210W	Over expressed	ERV15	ER Vesicle Protein
YLR300W	Over expressed	EXG1	EXo-1,3-beta-Glucanase
YJL157C	Over expressed	FAR1	Factor ARrest
YMR052W	Over expressed	FAR3	Factor ARrest
YKL182W	Over expressed	FAS1	Fatty Acid Synthetase
YMR277W	Over expressed	FCP1	tfiiF-associating component of Ctd Phosphatase
YPR062W	Over expressed	FCY1	FluoroCYtosine resistance
YBR101C	Over expressed	FES1	Factor Exchange for Ssa1p
YOR382W	Over expressed	FIT2	Facilitator of Iron Transport
YOR383C	Over expressed	FIT3	Facilitator of Iron Transport
YLR342W	Over expressed	FKS1	FK506 Sensitivity
YJL161W	Over expressed	FMP33	Found in Mitochondrial Proteome
YKR009C	Over expressed	FOX2	Fatty acid OXidation
YKL220C	Over expressed	FRE2	Ferric REductase
YLR047C	Over expressed	FRE8	0
YLR291C	Over expressed	GCD7	General Control Derepressed
YOR375C	Over expressed	GDH1	Glutamate DeHydrogenase
YNL153C	Over expressed	GIM3	Gene Involved in Microtubule biogenesis
YER054C	Over expressed	GIP2	Glc7-Interacting Protein
YCL040W	Over expressed	GLK1	GLucoKinase
YOR168W	Over expressed	GLN4	GLutamiNe metabolism

YJL184W	Over expressed	GON7	0
YGR216C	Over expressed	GPI1	GlycosylPhosphatidylInositol anchoring biosynthesis
YLL031C	Over expressed	GPI13	GlycosylPhosphatidylInositol anchor biosynthesis
YKL152C	Over expressed	GPM1	Glycerate PhosphoMutase
YER062C	Over expressed	GPP2	Glycerol-3-Phosphate Phosphatase
YPL059W	Over expressed	GRX5	GlutaRedoXin
YLR364W	Over expressed	GRX8	GlutaRedoXin
YMR251W	Over expressed	GTO3	Glutathione Transferase Omega-like
YGL245W	Over expressed	GUS1	GlUtamyl-tRNA Synthetase
YFL027C	Over expressed	GYP8	Gtpase-activating protein for Ypt Proteins
YBL021C	Over expressed	HAP3	Heme Activator Protein
YMR290C	Over expressed	HAS1	Helicase Associated with Set1
YNL281W	Over expressed	HCH1	High-Copy Hsp90 suppressor
YOR176W	Over expressed	HEM15	HEMe biosynthesis
YNL030W	Over expressed	HHF2	Histone H Four
YNL031C	Over expressed	HHT2	Histone H Three
YJR140C	Over expressed	HIR3	HIstone Regulation
YDR420W	Over expressed	HKR1	Hansenula mrakii Killer toxin Resistant
YML075C	Over expressed	HMG1	3-Hydroxy-3-MethylGlutaryl-coenzyme a reductase
YCL066W	Over expressed	HMLALPHA 1	Hidden Mat Left ALPHA
YCL067C	Over expressed	HMLALPHA 2	Hidden Mat Left ALPHA
YJR139C	Over expressed	HOM6	HOMoserine requiring
YMR251W-A	Over expressed	HOR7	HyperOsmolarity-Responsive
YKL084W	Over expressed	HOT13	Helper Of Tim
YLR097C	Over expressed	HRT3	High level expression Reduces Ty3 transposition
YMR186W	Over expressed	HSC82	0
YGL073W	Over expressed	HSF1	Heat Shock transcription Factor
YKL101W	Over expressed	HSL1	Histone Synthetic Lethal
YLL026W	Over expressed	HSP104	Heat Shock Protein
YBR072W	Over expressed	HSP26	Heat Shock Protein
YCR021C	Over expressed	HSP30	Heat Shock Protein
YDR258C	Over expressed	HSP78	Heat Shock Protein
YPL015C	Over expressed	HST2	Homolog of SIR Two (SIR2)
YDR225W	Over expressed	HTA1	Histone h Two A
YPL067C	Over expressed	HTC1	Histidine Triad with Channel
YNR032C-A	Over expressed	HUB1	Homologous to UBiquitin
YGL141W	Over expressed	HUL5	Hect Ubiquitin Ligase

YKL189W	Over expressed	HYM1	0
YNL037C	Over expressed	IDH1	Isocitrate DeHydrogenase
YOR136W	Over expressed	IDH2	Isocitrate DeHydrogenase
YNL009W	Over expressed	IDP3	Isocitrate Dehydrogenase, NADP-dependent
YBR159W	Over expressed	IFA38	0
YNL157W	Over expressed	IGO1	Initiation of G zerO
YJR016C	Over expressed	ILV3	IsoLeucine-plus-Valine requiring
YJR094C	Over expressed	IME1	Inducer of MEiosis
YHR046C	Over expressed	INM1	INositol Monophosphatase
YMR044W	Over expressed	IOC4	Iswi One Complex
YDR315C	Over expressed	IPK1	Inositol Polyphosphate Kinase
YOR044W	Over expressed	IRC23	Increased Recombination Centers
YJL051W	Over expressed	IRC8	Increased Recombination Centers
YKL032C	Over expressed	IXR1	Intrastrand cross (X)-link Recognition
YKL217W	Over expressed	JEN1	0
YMR294W	Over expressed	JNM1	Just Nuclear Migration
YGL241W	Over expressed	KAP114	KAryoPherin
YPR141C	Over expressed	KAR3	KARyogamy
YCL055W	Over expressed	KAR4	KARyogamy
YJL094C	Over expressed	KHA1	K/H ion Antiporter
YHR102W	Over expressed	KIC1	Kinase that Interacts with Cdc31p
YLR096W	Over expressed	KIN2	KINase
YAR018C	Over expressed	KIN3	protein KINase
YOR233W	Over expressed	KIN4	KINase
YOR099W	Over expressed	KTR1	Kre Two Related
YJL134W	Over expressed	LCB3	Long-Chain Base
YCL005W	Over expressed	LDB16	Low Dye Binding
YLL049W	Over expressed	LDB18	Low Dye Binding
YMR147W	Over expressed	LDO45	Lipid Droplet Organization protein of 45 Kda
YJR107W	Over expressed	LIH1	LIpase Homolog
YGR136W	Over expressed	LSB1	Las Seventeen Binding protein
YGL099W	Over expressed	LSG1	Large-Subunit Gtpase
YPR073C	Over expressed	LTP1	Low molecular weight protein Tyrosine Phosphatase
YNR050C	Over expressed	LYS9	LYSine requiring
YGR288W	Over expressed	MAL13	MALtose fermentation
YHR024C	Over expressed	MAS2	Mitochondrial ASsembly
YOR298C-A	Over expressed	MBF1	Multiprotein Bridging Factor
YKL150W	Over expressed	MCR1	Mitochondrial NADH-Cytochrome b5 Reductase
YLR188W	Over expressed	MDL1	MultiDrug resistance-Like

YML104C	Over expressed	MDM1	Mitochondrial Distribution and Morphology
YKL053C-A	Over expressed	MDM35	Mitochondrial Distribution and Morphology
YOL111C	Over expressed	MDY2	Mating-Deficient Yeast
YLR069C	Over expressed	MEF1	Mitochondrial Elongation Factor
YPL023C	Over expressed	MET12	METhionine requiring
YDL200C	Over expressed	MGT1	O-6-MethylGuanine-DNA methylTransferase
YOL026C	Over expressed	MIM1	Mitochondrial IMport
YHR015W	Over expressed	MIP6	Mex67-Interacting Protein
YPL140C	Over expressed	MKK2	Mitogen-activated Kinase Kinase
YOR350C	Over expressed	MNE1	0
YER001W	Over expressed	MNN1	MaNNosyltransferase
YKL064W	Over expressed	MNR2	MaNganese Resistance
YNL297C	Over expressed	MON2	MONensin sensitivity
YOR288C	Over expressed	MPD1	Multicopy suppressor of PDI1 deletion
YDL247W	Over expressed	MPH2	Maltose Permease Homolog
YJL019W	Over expressed	MPS3	MonoPolar Spindle
YDR033W	Over expressed	MRH1	Membrane protein Related to Hsp30p
YBL090W	Over expressed	MRP21	Mitochondrial Ribosomal Protein
YKR085C	Over expressed	MRPL20	Mitochondrial Ribosomal Protein, Large subunit
YJL096W	Over expressed	MRPL49	Mitochondrial Ribosomal Protein, Large subunit
YJL063C	Over expressed	MRPL8	Mitochondrial Ribosomal Protein, Large subunit
YGR165W	Over expressed	MRPS35	Mitochondrial Ribosomal Protein, Small subunit
YNL293W	Over expressed	MSB3	Multicopy Suppressor of Bud Emergence
YNR049C	Over expressed	MSO1	Multicopy suppressor of Sec One
YKL194C	Over expressed	MST1	Mitochondrial aminoacyl-tRNA Synthetase, Threonine
YMR228W	Over expressed	MTF1	Mitochondrial Transcription Factor
YKL186C	Over expressed	MTR2	Mrna TRansport
YAL029C	Over expressed	MYO4	MYOsin
YOR253W	Over expressed	NAT5	N-terminal AcetylTransferase
YDR162C	Over expressed	NBP2	Nap1 Binding Protein
YHR124W	Over expressed	NDT80	Non-DiTyrosine
YHR035W	Over expressed	NEL1	Non-ERES Localized Sec23 homolog 1
YJL076W	Over expressed	NET1	Nucleolar silencing Establishing factor and Telophase regulator
YBR212W	Over expressed	NGR1	Negative Growth Regulatory protein
YPL174C	Over expressed	NIP100	Nuclear ImPort
YNL123W	Over expressed	NMA111	Nuclear Mediator of Apoptosis
YJR132W	Over expressed	NMD5	Nonsense-Mediated mRNA Decay
YKL151C	Over expressed	NNR2	Nicotinamide Nucleotide Repair
YOR209C	Over expressed	NPT1	Nicotinate PhosphoribosylTransferase

YNL156C	Over expressed	NSG2	0
YDR026C	Over expressed	NSI1	NTS1 SIlencing protein 1
YOL043C	Over expressed	NTG2	eNdonuclease Three-like Glycosylase
YKR082W	Over expressed	NUP133	NUclear Pore
YIL115C	Over expressed	NUP159	NUclear Pore
YHR195W	Over expressed	NVJ1	Nucleus-Vacuole Junction
YKL055C	Over expressed	OAR1	3-Oxoacyl-[Acyl-carrier-protein] Reductase
Q0130	Over expressed	OLI1	OLIgomycin resistance
YKR003W	Over expressed	OSH6	OxySterol binding protein Homolog
YOR103C	Over expressed	OST2	OligoSaccharylTransferase
YGL226C-A	Over expressed	OST5	OligoSaccharylTransferase
YML019W	Over expressed	OST6	OligoSaccharylTransferase
YCR104W	Over expressed	PAU3	seriPAUperin family
YLR199C	Over expressed	PBA1	Proteasome Biogenesis-Associated
YNL015W	Over expressed	PBI2	Proteinase B Inhibitor
YNL289W	Over expressed	PCL1	Pho85 CycLin
YHR071W	Over expressed	PCL5	Pho85 CycLin
YDR081C	Over expressed	PDC2	Pyruvate DeCarboxylase
YGL013C	Over expressed	PDR1	Pleiotropic Drug Resistance
YBL017C	Over expressed	PEP1	carboxyPEPtidase Y-deficient
YLR067C	Over expressed	PET309	PETite colonies
YGL153W	Over expressed	PEX14	PEroXisome related
YOL044W	Over expressed	PEX15	PEroXisome related
YGR004W	Over expressed	PEX31	PEroXisome related
YJL179W	Over expressed	PFD1	PreFolDin
YNL149C	Over expressed	PGA2	Processing of Gas1p and ALP
YBR196C	Over expressed	PGI1	PhosphoGlucoIsomerase
YMR105C	Over expressed	PGM2	PhosphoGlucoMutase
YJL117W	Over expressed	PHO86	PHOsphate metabolism
YDR374C	Over expressed	PHO92	PHOsphate metabolism
YDR313C	Over expressed	PIB1	PhosphatidylInositol(3)-phosphate Binding
YML061C	Over expressed	PIF1	Petite Integration Frequency
YOR104W	Over expressed	PIN2	Psi+ INducibility
YNL082W	Over expressed	PMS1	PostMeiotic Segregation
YJR043C	Over expressed	POL32	POLymerase
YNL217W	Over expressed	PPN2	0
YMR314W	Over expressed	PRE5	PRoteinase yscE
YKL045W	Over expressed	PRI2	DNA PRImase
YNL279W	Over expressed	PRM1	Pheromone-Regulated Membrane protein

YPR178W	Over expressed	PRP4	Pre-mRNA Processing
YMR308C	Over expressed	PSE1	Protein Secretion Enhancer
YDR032C	Over expressed	PST2	Protoplasts-SecreTed
YOL114C	Over expressed	PTH4	peptidyl tRNA hydrolase 4
YNL016W	Over expressed	PUB1	PolyUridylate Binding
YNR063W	Over expressed	PUL4	PULcherrimin
YGR253C	Over expressed	PUP2	PUtative Proteasome subunit
YNL010W	Over expressed	PYP1	PolYol Phosphatase
YHR074W	Over expressed	QNS1	glutamine (Q) dependent Nad+ Synthetase
YJR035W	Over expressed	RAD26	RADiation sensitive
YNL250W	Over expressed	RAD50	RADiation sensitive
YDR014W	Over expressed	RAD61	RADiation sensitive
YJR052W	Over expressed	RAD7	RADiation sensitive
YOR265W	Over expressed	RBL2	Rescues Beta-tubulin Lethality
YDL189W	Over expressed	RBS1	RNA-Binding Suppressor of PAS kinase
YML030W	Over expressed	RCF1	Respiratory superComplex Factor
YCR106W	Over expressed	RDS1	Regulator of Drug Sensitivity
YPR094W	Over expressed	RDS3	Regulator of Drug Sensitivity
YLR387C	Over expressed	REH1	REI1 Homolog
YBR002C	Over expressed	RER2	Retention in the Endoplasmic Reticulum
YJL173C	Over expressed	RFA3	Replication Factor A
YOR217W	Over expressed	RFC1	Replication Factor C
YNL290W	Over expressed	RFC3	Replication Factor C
YLR073C	Over expressed	RFU1	Regulator of Free Ubiquitin chains
YLR176C	Over expressed	RFX1	Regulatory Factor X
YPL066W	Over expressed	RGL1	Rho1 GEF Localizing
YOR107W	Over expressed	RGS2	Regulator of heterotrimeric G protein Signaling
YNL090W	Over expressed	RHO2	Ras HOmolog
YMR139W	Over expressed	RIM11	Regulator of IME2
YDR257C	Over expressed	RKM4	Ribosomal lysine (K) Methyltransferase
YKL132C	Over expressed	RMA1	Reduced Mating A
YMR234W	Over expressed	RNH1	RNase H
YGR276C	Over expressed	RNH70	RNase H
YJL026W	Over expressed	RNR2	RiboNucleotide Reductase
YGL171W	Over expressed	ROK1	Rescuer Of Kem1
YPR010C	Over expressed	RPA135	RNA Polymerase A
YOL005C	Over expressed	RPB11	RNA Polymerase B
YKL144C	Over expressed	RPC25	RNA Polymerase C
YJL121C	Over expressed	RPE1	Ribulose 5-Phosphate Epimerase

YLR061W	Over expressed	RPL22A	Ribosomal Protein of the Large subunit
YGR148C	Over expressed	RPL24B	Ribosomal Protein of the Large subunit
YGL103W	Over expressed	RPL28	Ribosomal Protein of the Large subunit
YIL018W	Over expressed	RPL2B	Ribosomal Protein of the Large subunit
YBL092W	Over expressed	RPL32	Ribosomal Protein of the Large subunit
YER056C-A	Over expressed	RPL34A	Ribosomal Protein of the Large subunit
YHR141C	Over expressed	RPL42B	Ribosomal Protein of the Large subunit
YML091C	Over expressed	RPM2	RNase P Mitochondrial
YLR421C	Over expressed	RPN13	Regulatory Particle Non-ATPase
YDL140C	Over expressed	RPO21	RNA POlymerase
YOL121C	Over expressed	RPS19A	Ribosomal Protein of the Small subunit
YER074W	Over expressed	RPS24A	Ribosomal Protein of the Small subunit
YIL069C	Over expressed	RPS24B	Ribosomal Protein of the Small subunit
YNL178W	Over expressed	RPS3	Ribosomal Protein of the Small subunit
YNL096C	Over expressed	RPS7B	Ribosomal Protein of the Small subunit
YOL117W	Over expressed	RRI2	0
YPR137W	Over expressed	RRP9	Ribosomal RNA Processing
YDR303C	Over expressed	RSC3	Remodel the Structure of Chromatin
YGL129C	Over expressed	RSM23	Ribosomal Small subunit of Mitochondria
YMR266W	Over expressed	RSN1	Rescue of Sro7 at high Nacl
YNL254C	Over expressed	RTC4	Restriction of Telomere Capping
YDR233C	Over expressed	RTN1	ReTiculoN-like
YNL206C	Over expressed	RTT106	Regulator of Ty1 Transposition
YDR139C	Over expressed	RUB1	Related to UBiquitin
YBR095C	Over expressed	RXT2	0
YDR502C	Over expressed	SAM2	S-AdenosylMethionine requiring
YGL229C	Over expressed	SAP4	Sit4 Associated Protein
YMR127C	Over expressed	SAS2	Something About Silencing
YDR180W	Over expressed	SCC2	Sister Chromatid Cohesion
YGR049W	Over expressed	SCM4	Suppressor of Cdc4 Mutation
YER120W	Over expressed	SCS2	Suppressor of Choline Sensitivity
YGL056C	Over expressed	SDS23	homolog of S. pombe SDS23
YLR208W	Over expressed	SEC13	SECretory
YIL109C	Over expressed	SEC24	SECretory
YFL005W	Over expressed	SEC4	SECretory
YDR077W	Over expressed	SED1	Suppression of Exponential Defect
YLR026C	Over expressed	SED5	Suppressor of Erd2 Deletion
YGL100W	Over expressed	SEH1	SEc13 Homolog
YLR404W	Over expressed	SEI1	SEIpin

YDL168W	Over expressed	SFA1	Sensitive to FormAldehyde
YLR321C	Over expressed	SFH1	Snf Five Homolog
YJL145W	Over expressed	SFH5	Sec Fourteen Homolog
YOR140W	Over expressed	SFL1	Suppressor gene for FLocculation
YKL006C-A	Over expressed	SFT1	Suppressor of sed Five Ts
YJR134C	Over expressed	SGM1	Slow growth on Galactose and Mannose
YIL104C	Over expressed	SHQ1	Small nucleolar RNAs of the box H/ACA family Quantitative accumulation
YNL236W	Over expressed	SIN4	Switch INdependent
YJL089W	Over expressed	SIP4	SNF1-Interacting Protein
YKR100C	Over expressed	SKG1	Suppressor of lethality of Kex2 Gas1 double null mutant
YDL033C	Over expressed	SLM3	Synthetic Lethal with Mss4
YJL074C	Over expressed	SMC3	Stability of MiniChromosomes
YLR147C	Over expressed	SMD3	0
YOL122C	Over expressed	SMF1	Suppressor of Mitochondria import Function
YBR172C	Over expressed	SMY2	Suppressor of MYo2-66
YOR327C	Over expressed	SNC2	Suppressor of the Null allele of CAP
YDL194W	Over expressed	SNF3	Sucrose NonFermenting
YMR322C	Over expressed	SNO4	SNZ proximal Open reading frame
YGL131C	Over expressed	SNT2	0
YDL098C	Over expressed	SNU23	Small NUclear ribonucleoprotein associated
YDR240C	Over expressed	SNU56	Small NUclear ribonucleoprotein associated
YOR357C	Over expressed	SNX3	Sorting NeXin
YNR034W	Over expressed	SOL1	Suppressor Of Los1-1
YLR066W	Over expressed	SPC3	Signal Peptidase Complex
YLR313C	Over expressed	SPH1	SPa2 Homolog
YER150W	Over expressed	SPI1	Stationary Phase Induced
YML010W	Over expressed	SPT5	SuPpressor of Ty's
YBR081C	Over expressed	SPT7	SuPpressor of Ty's
YLR082C	Over expressed	SRL2	Suppressor of Rad53 null Lethality
YDR292C	Over expressed	SRP101	Signal Recognition Particle
YKL154W	Over expressed	SRP102	Signal Recognition Particle
YLL024C	Over expressed	SSA2	Stress-Seventy subfamily A
YER103W	Over expressed	SSA4	Stress-Seventy subfamily A
YPL106C	Over expressed	SSE1	Stress Seventy subfamily E
YLR006C	Over expressed	SSK1	Suppressor of Sensor Kinase
YLR005W	Over expressed	SSL1	Suppressor of Stem-Loop mutation
YLR250W	Over expressed	SSP120	Saccharomyces Secretory Protein
YLR452C	Over expressed	SST2	SuperSensiTive
YPL092W	Over expressed	SSU1	Sensitive to SUlfite

YHR084W	Over expressed	STE12	STErile
YFL026W	Over expressed	STE2	STErile
YLR375W	Over expressed	STP3	protein with similarity to Stp1p
YBR231C	Over expressed	SWC5	SWr Complex
YLR385C	Over expressed	SWC7	SWr Complex
YJL176C	Over expressed	SWI3	SWItching deficient
YNL187W	Over expressed	SWT21	Synthetic With Tgs1
YDR395W	Over expressed	SXM1	Suppressor of mRNA eXport Mutant
YIL047C	Over expressed	SYG1	Suppressor of Yeast Gpa1
YML015C	Over expressed	TAF11	TATA binding protein-Associated Factor
YBR069C	Over expressed	TAT1	Tyrosine and tryptophan Amino acid Transporter
YPR157W	Over expressed	TDA6	Topoisomerase I Damage Affected
YPL122C	Over expressed	TFB2	Transcription Factor B subunit 2
YOL055C	Over expressed	THI20	THIamine metabolism
YPL214C	Over expressed	THI6	THIamine biosynthesis
YPR045C	Over expressed	THP3	THO-related Protein
YGR033C	Over expressed	TIM21	Translocase of the Inner Mitochondrial membrane
YIL011W	Over expressed	TIR3	TIp1-Related
YOL018C	Over expressed	TLG2	T-snare affecting a Late Golgi compartment
YDR105C	Over expressed	TMS1	0
YGL179C	Over expressed	TOS3	Target Of Sbf
YNL300W	Over expressed	TOS6	0
YMR156C	Over expressed	TPP1	Three Prime Phosphatase
YMR261C	Over expressed	TPS3	Trehalose Phosphate Synthase
YOL093W	Over expressed	TRM10	Transfer RNA Methyltransferase
YML005W	Over expressed	TRM12	TRna Methyltransferase
YKL211C	Over expressed	TRP3	TRyPtophan
YHR106W	Over expressed	TRR2	ThioRedoxin Reductase
YML085C	Over expressed	TUB1	TUBulin
YDR100W	Over expressed	TVP15	Tlg2-Vesicle Protein
YGR080W	Over expressed	TWF1	TWinFilin
YJL048C	Over expressed	UBX6	UBiquitin regulatory X
YDL170W	Over expressed	UGA3	Utilization of GAba
YML021C	Over expressed	UNG1	Uracil DNA N-Glycosylase
YEL021W	Over expressed	URA3	URAcil requiring
YNL229C	Over expressed	URE2	UREidosuccinate transport
YHR196W	Over expressed	UTP9	U Three Protein
YDR247W	Over expressed	VHS1	Viable in a Hal3 Sit4 background
YDL185W	Over expressed	VMA1	Vacuolar Membrane Atpase

YBR127C	Over expressed	VMA2	Vacuolar Membrane Atpase
YLR261C	Over expressed	VPS63	Vacuolar Protein Sorting
YFL004W	Over expressed	VTC2	Vacuolar Transporter Chaperone
YOL097C	Over expressed	WRS1	W (tryptophan) RS (tRNA synthetase)
YOR230W	Over expressed	WTM1	WD repeat containing Transcriptional Modulator
YGR194C	Over expressed	XKS1	XyluloKinaSe
YIR018W	Over expressed	YAP5	Yeast AP-1
YDR259C	Over expressed	YAP6	Yeast homolog of AP-1
YDR319C	Over expressed	YFT2	0
YDR451C	Over expressed	YHP1	Yeast Homeo-Protein
YNL044W	Over expressed	YIP3	Ypt-Interacting Protein
YGL161C	Over expressed	YIP5	Ypt-Interacting Protein
YDR057W	Over expressed	YOS9	Yeast OS-9 homolog
YKL126W	Over expressed	YPK1	Yeast Protein Kinase
YNL093W	Over expressed	YPT53	Yeast Protein Two
YHR016C	Over expressed	YSC84	0
YPL074W	Over expressed	YTA6	Yeast Tat-binding Analog
YJL139C	Over expressed	YUR1	Yeast Unknown Reading frame
YJL056C	Over expressed	ZAP1	Zinc-responsive Activator Protein
YDR285W	Over expressed	ZIP1	molecular ZIPper
YFL052W	Over expressed	ZNF1	ZiNc Finger protein
YBL005W-A	Over expressed		
YBR012W-B	Over expressed		
YCL002C	Over expressed		
YCL019W	Over expressed		
YDL241W	Over expressed		
YDR098C-B	Over expressed		
YDR170W-A	Over expressed		
YDR261C-C	Over expressed		
YDR261W-B	Over expressed		
YDR262W	Over expressed		
YDR341C	Over expressed		
YDR365W-A	Over expressed		
YDR365W-B	Over expressed		
YER159C-A	Over expressed		
YFL002W-B	Over expressed		
YGR038C-A	Over expressed		
YGR210C	Over expressed		
YHL009W-A	Over expressed		

YHR214C-B	Over expressed		
YIL089W	Over expressed		
YIL168W	Over expressed		
YJL218W	Over expressed		
YJR029W	Over expressed		
YJR096W	Over expressed		
YKL033W-A	Over expressed		
YKL071W	Over expressed		
YKL162C	Over expressed		
YKR005C	Over expressed		
YLL032C	Over expressed		
YLR035C-A	Over expressed		
YLR157C-A	Over expressed		
YLR227W-B	Over expressed		
YLR278C	Over expressed		
YLR326W	Over expressed		
YML039W	Over expressed		
YML045W	Over expressed		
YML053C	Over expressed		
YML096W	Over expressed		
YNL054W-B	Over expressed		
YNL208W	Over expressed		
YNL284C-B	Over expressed		
YNR029C	Over expressed		
YNR071C	Over expressed		
YOL103W-A	Over expressed		
YOL103W-B	Over expressed		
YOL163W	Over expressed		
YOR142W-B	Over expressed		
YOR338W	Over expressed		
ARA5	Over expressed		
Q0297	Over expressed		
YAL038W-R	Over expressed		
YAL043C-A	Over expressed		
YAL064C-A	Over expressed		
YBL096C	Over expressed		
YBR011C-R	Over expressed		
YBR063C	Over expressed		
YBR134W	Over expressed		

YBR174C	Over expressed		
YBR206W	Over expressed		
YBR300C	Over expressed		
YCR064C	Over expressed		
YDL023C	Over expressed		
YDL196W	Over expressed		
YDR015C	Over expressed		
YDR114C	Over expressed		
YDR193W	Over expressed		
YDR210W-C	Over expressed		
YDR271C	Over expressed		
YDR355C	Over expressed		
YDR445C	Over expressed		
YDR525W	Over expressed		
YER053C-A-R	Over expressed		
YER066W	Over expressed		
YER085C	Over expressed		
YER119C-A	Over expressed		
YER158C	Over expressed		
YFL006W	Over expressed		
YFL019C	Over expressed		
YFL032W	Over expressed		
YFR024C	Over expressed		
YGL034C	Over expressed		
YGL055W-R	Over expressed		
YGL149W	Over expressed		
YGL235W	Over expressed		
YGR240C-R	Over expressed		
YGR273C	Over expressed		
YIL163C	Over expressed		
YIL175W	Over expressed		
YJL027C	Over expressed		
YJL118W	Over expressed		
YJL119C	Over expressed		
YJL130C-R	Over expressed		
YJL181W	Over expressed		
YJL182C	Over expressed		
YJL202C	Over expressed		
YJL205C-A	Over expressed		

YJR018W	Over expressed		
YJR104C-R	Over expressed		
YJR146W	Over expressed		
YKL053W	Over expressed		
YKL097C	Over expressed		
YKL115C	Over expressed		
YLL039C-R	Over expressed		
YLL059C	Over expressed		
YLR049C	Over expressed		
YLR053C	Over expressed		
YLR124W	Over expressed		
YLR140W	Over expressed		
YLR184W	Over expressed		
YLR407W	Over expressed		
YML095C-A	Over expressed		
YML100W-A	Over expressed		
YMR103C	Over expressed		
YMR119W-A	Over expressed		
YMR130W	Over expressed		
YMR173W-A	Over expressed		
YMR265C	Over expressed		
YNL013C	Over expressed		
YNL050C	Over expressed		
YNL165W	Over expressed		
YNL205C	Over expressed		
YNL269W	Over expressed		
YNL285W	Over expressed		
YNL295W	Over expressed		
YNR005C	Over expressed		
YOL131W	Over expressed		
YOR008C-A	Over expressed		
YOR041C	Over expressed		
YOR225W	Over expressed		
YOR240W	Over expressed		
YOR343C	Over expressed		
YOR343C-A	Over expressed		
YPL073C	Over expressed		
YPL102C	Over expressed		
YPR016C-R	Over expressed		

YPR099C	Over expressed		
YJR155W	Down expressed	AAD10	Aryl-Alcohol Dehydrogenase
YFL057C	Down expressed	AAD16	Aryl Alcohol Dehydrogenase
YDL243C	Down expressed	AAD4	Aryl-Alcohol Dehydrogenase
YHR047C	Down expressed	AAP1	Arginine/alanine AminoPeptidase
YBL074C	Down expressed	AAR2	A1-Alpha2 Repression
YKL106W	Down expressed	AAT1	Aspartate AminoTransferase
YJR108W	Down expressed	ABM1	Aberrant Microtubules
YCR088W	Down expressed	ABP1	Actin Binding Protein
YGR037C	Down expressed	ACB1	Acyl-CoA-Binding
YLR153C	Down expressed	ACS2	Acetyl CoA Synthetase
YCR105W	Down expressed	ADH7	Alcohol DeHydrogenase
YCR011C	Down expressed	ADP1	ATP-Dependent Permease
YMR282C	Down expressed	AEP2	ATPase ExPression
YCR082W	Down expressed	AHC2	Ada Histone acetyltransferase complex Component
YIL087C	Down expressed	AIM19	Altered Inheritance rate of Mitochondria
YBR194W	Down expressed	AIM4	Altered Inheritance rate of Mitochondria
YBR059C	Down expressed	AKL1	Ark family Kinase-Like protein
YNR030W	Down expressed	ALG12	Asparagine-Linked Glycosylation
YNL270C	Down expressed	ALP1	0
YKR021W	Down expressed	ALY1	Arrestin-Like Yeast protein
YGR225W	Down expressed	AMA1	Activator of Meiotic APC/C
YBR211C	Down expressed	AME1	Associated with Microtubules and Essential
YKL047W	Down expressed	ANR2	Avl Nine Related family 2
YMR010W	Down expressed	ANY1	Antagonizes Neo1 Yeast phospholipid flippase
YDR118W	Down expressed	APC4	Anaphase Promoting Complex
YOL062C	Down expressed	APM4	clathrin Adaptor Protein complex Medium chain
YLL052C	Down expressed	AQY2	AQuaporin from Yeast
YKL013C	Down expressed	ARC19	ARp2/3 Complex subunit
YDL192W	Down expressed	ARF1	ADP-Ribosylation Factor
YDR173C	Down expressed	ARG82	ARGinine requiring
YDR106W	Down expressed	ARP10	Actin-Related Protein
YDL029W	Down expressed	ARP2	Actin-Related Protein
YPR085C	Down expressed	ASA1	AStra Associated protein
YKL052C	Down expressed	ASK1	Associated with Spindles and Kinetochores
YPR145W	Down expressed	ASN1	ASparagiNe requiring
YGR177C	Down expressed	ATF2	AcetylTransFerase
YGL180W	Down expressed	ATG1	AuTophaGy related

YPR185W	Down expressed	ATG13	AuTophaGy related
YOL083W	Down expressed	ATG34	AuTophaGy related
YNL315C	Down expressed	ATP11	ATP synthase
YDL004W	Down expressed	ATP16	ATP synthase
YNL259C	Down expressed	ATX1	AnTioXidant
YOR134W	Down expressed	BAG7	0
YPL255W	Down expressed	BBP1	Bfr1 Binding Protein
YDL070W	Down expressed	BDF2	BromoDomain Factor
YLR412W	Down expressed	BER1	Benomyl REsistant 1
YOR198C	Down expressed	BFR1	BreFeldin A Resistance
YBR141C	Down expressed	BMT2	Base MethylTransferase of 25S RNA
YJR078W	Down expressed	BNA2	Biosynthesis of Nicotinic Acid
YLR231C	Down expressed	BNA5	Biosynthesis of Nicotinic Acid
YIL159W	Down expressed	BNR1	BNi1 Related
YCR032W	Down expressed	BPH1	Beige Protein Homolog
YBL097W	Down expressed	BRN1	homolog of BaRreN
YPL069C	Down expressed	BTS1	Bet Two Suppressor
YKL092C	Down expressed	BUD2	BUD site selection
YKL005C	Down expressed	BYE1	BYpass of ESS1
YGR277C	Down expressed	CAB4	Coenzyme A Biosynthesis
YDR196C	Down expressed	CAB5	0
YNL278W	Down expressed	CAF120	CCR4 Associated Factor
YMR280C	Down expressed	CAT8	CATabolite repression
YJL209W	Down expressed	CBP1	Cytochrome B mRNA Processing
YBR120C	Down expressed	CBP6	Cytochrome B Protein synthesis
YLR220W	Down expressed	CCC1	Cross-Complements Ca(2+) phenotype of csg1
YFR028C	Down expressed	CDC14	Cell Division Cycle
YLR310C	Down expressed	CDC25	Cell Division Cycle
YMR001C	Down expressed	CDC5	Cell Division Cycle
YJR057W	Down expressed	CDC8	Cell Division Cycle
YGL003C	Down expressed	CDH1	CDC20 Homolog
YMR168C	Down expressed	CEP3	CEntromere Protein
YIL003W	Down expressed	CFD1	Cytosolic Fe-S cluster Deficient
YML036W	Down expressed	CGI121	homolog of human CGI-121
YLR330W	Down expressed	CHS5	CHitin Synthase-related
YER030W	Down expressed	CHZ1	Chaperone for Htz1/H2A-H2B dimer
YCR005C	Down expressed	CIT2	CITrate synthase
YPR001W	Down expressed	CIT3	CITrate synthase
YAL040C	Down expressed	CLN3	CycLiN

YBL059C-A	Down expressed	CMC2	Cx9C Motif-Containing protein
YDR357C	Down expressed	CNL1	CNo-Like
YBR302C	Down expressed	COS2	COnserved Sequence
YOR316C	Down expressed	COT1	CObalt Toxicity
YDL067C	Down expressed	COX9	Cytochrome c OXidase
YOR031W	Down expressed	CRS5	Copper-Resistant Suppressor
YNL027W	Down expressed	CRZ1	Calcineurin-Responsive Zinc finger
YIL132C	Down expressed	CSM2	Chromosome Segregation in Meiosis
YMR078C	Down expressed	CTF18	Chromosome Transmission Fidelity
YKL139W	Down expressed	CTK1	Carboxy-Terminal domain Kinase
YGR088W	Down expressed	CTT1	CaTalase T
YMR264W	Down expressed	CUE1	Coupling of Ubiquitin conjugation to ER degradation
YML101C	Down expressed	CUE4	Coupling of Ubiquitin conjugation to ER degradation
YGR003W	Down expressed	CUL3	CULlin
YDR430C	Down expressed	CYM1	CYtosolic Metalloprotease
YGR113W	Down expressed	DAM1	Duo1 And Mps1 interacting
YDR020C	Down expressed	DAS2	Dst1-delta 6-Azauracil Sensitivity
YNL112W	Down expressed	DBP2	Dead Box Protein
YKL149C	Down expressed	DBR1	DeBRanching
YFL061W	Down expressed	DDI2	DNA Damage Inducible
YBR201W	Down expressed	DER1	Degradation in the Endoplasmic Reticulum
YMR238W	Down expressed	DFG5	Defective for Filamentous Growth
YPR082C	Down expressed	DIB1	S. pombe DIm1+ in Budding yeast
YKR035W-A	Down expressed	DID2	Doa4-Independent Degradation
YKL002W	Down expressed	DID4	Doa4-Independent Degradation
YDL178W	Down expressed	DLD2	D-Lactate Dehydrogenase
YLL001W	Down expressed	DNM1	DyNaMin-related
YKL213C	Down expressed	DOA1	Degradation Of Alpha
YDR284C	Down expressed	DPP1	Diacylglycerol Pyrophosphate Phosphatase
YDR370C	Down expressed	DXO1	Decapping eXOnuclease
YJR082C	Down expressed	EAF6	Esa1p-Associated Factor
YJR106W	Down expressed	ECM27	ExtraCellular Mutant
YER176W	Down expressed	ECM32	ExtraCellular Mutant
YMR212C	Down expressed	EFR3	PHO Eighty Five Requiring
YDR036C	Down expressed	EHD3	0
YPL046C	Down expressed	ELC1	ELongin C
YKL160W	Down expressed	ELF1	ELongation Factor
YLR050C	Down expressed	EMA19	Efficient Mitochondria targeting-Associated protein
YCL045C	Down expressed	EMC1	ER Membrane protein Complex

YLL014W	Down expressed	EMC6	ER Membrane protein Complex
YFL048C	Down expressed	EMP47	0
YDR040C	Down expressed	ENA1	Exitus NAtru (Latin, "exit sodium")
YLL038C	Down expressed	ENT4	Epsin N-Terminal homology
YPL281C	Down expressed	ERR2	Enolase-Related Repeat
YGL054C	Down expressed	ERV14	ER Vesicle
YLR318W	Down expressed	EST2	Ever Shorter Telomeres
YDL045C	Down expressed	FAD1	FAD synthetase
YLR238W	Down expressed	FAR10	Factor ARrest
YBR041W	Down expressed	FAT1	FATty acid transporter
YER056C	Down expressed	FCY2	FluoroCYtosine resistance
YDR539W	Down expressed	FDC1	Ferulic acid DeCarboxylase
YCR028C	Down expressed	FEN2	FENpropimorph resistance
YPR104C	Down expressed	FHL1	Fork Head-Like
YKR102W	Down expressed	FLO10	FLOcculation
YHR176W	Down expressed	FMO1	Flavin containing MonoOxygenase
YFL046W	Down expressed	FMP32	Found in Mitochondrial Proteome
YER004W	Down expressed	FMP52	Found in Mitochondrial Proteome
YDR110W	Down expressed	FOB1	FOrk Blocking less
YLL051C	Down expressed	FRE6	Ferric REductase
YFL022C	Down expressed	FRS2	phenylalanyl (F)-tRNA Synthetase
YCR076C	Down expressed	FUB1	FUnction of Boundary
YCL027W	Down expressed	FUS1	cell FUSion
YBR020W	Down expressed	GAL1	GALactose metabolism
YML051W	Down expressed	GAL80	GALactose metabolism
YMR307W	Down expressed	GAS1	Glycophospholipid-Anchored Surface protein
YMR215W	Down expressed	GAS3	Glycophospholipid-Anchored Surface protein
YGR083C	Down expressed	GCD2	General Control Derepressed
YFR009W	Down expressed	GCN20	General Control Nonderepressible
YDL226C	Down expressed	GCS1	Growth Cold Sensitive
YOR120W	Down expressed	GCY1	Galactose-inducible Crystallin-like Yeast protein
YPL110C	Down expressed	GDE1	GlycerophosphoDiEsterase
YOR205C	Down expressed	GEP3	GEnetic interactors of Prohibitins
YHR100C	Down expressed	GEP4	GEnetic interactors of Prohibitins
YKL104C	Down expressed	GFA1	Glutamine:Fructose-6-phosphate Amidotransferase
YDR507C	Down expressed	GIN4	Growth Inhibitory
YBR045C	Down expressed	GIP1	Glc7-Interacting Protein
YDR152W	Down expressed	GIR2	Genetically Interacts with Ribosomal genes
YPR109W	Down expressed	GLD1	Golgi/endosome Localized Dsc protein

YEL046C	Down expressed	GLY1	GLYcine requiring
YDR506C	Down expressed	GMC1	Grand Meiotic recombination Cluster
YBR244W	Down expressed	GPX2	Glutathione PeroXidase
YPL223C	Down expressed	GRE1	Genes de Respuesta a Estres (spanish for stress responsive genes)
YDR517W	Down expressed	GRH1	GRasp65 (Golgi reassembly stacking protein of 65kD) Homolog
YJR090C	Down expressed	GRR1	Glucose Repression-Resistant
YPR081C	Down expressed	GRS2	Glycyl-tRNA Synthase
YDR098C	Down expressed	GRX3	GlutaRedoXin
YML048W	Down expressed	GSF2	Glucose Signaling Factor
YDR221W	Down expressed	GTB1	Glucosidase Two Beta-subunit
YML121W	Down expressed	GTR1	GTp binding protein Resemblance
YIR038C	Down expressed	GTT1	GlutaThione Transferase
YGL237C	Down expressed	HAP2	Heme Activator Protein
YKR084C	Down expressed	HBS1	Hsp70 subfamily B Suppressor
YJL033W	Down expressed	HCA4	Helicase CA
YPR179C	Down expressed	HDA3	Histone DeAcetylase
YER014W	Down expressed	HEM14	HEMe biosynthesis
YPL254W	Down expressed	HFI1	Histone H2A Functional Interactor
YLL022C	Down expressed	HIF1	Hat1 Interacting Factor
YDR174W	Down expressed	HMO1	High MObility group (HMG) family
YGL033W	Down expressed	HOP2	HOmologous Pairing
YDR399W	Down expressed	HPT1	Hypoxanthine guanine PhosphoribosylTransferase
YPL204W	Down expressed	HRR25	HO and Radiation Repair
YBR133C	Down expressed	HSL7	Histone Synthetic Lethal
YOR020C	Down expressed	HSP10	Heat Shock Protein
YLR259C	Down expressed	HSP60	Heat Shock Protein
YPR033C	Down expressed	HTS1	Histidine-Trna Synthetase
YOR284W	Down expressed	HUA2	0
YPL117C	Down expressed	IDI1	Isopentenyl Diphosphate Isomerase
YJL216C	Down expressed	IMA5	IsoMAltase
YAR073W	Down expressed	IMD1	IMP Dehydrogenase
YJL082W	Down expressed	IML2	Increased Minichromosome Loss
YGR031W	Down expressed	IMO32	Intermediate cleaved by Mitochondrial Octapeptidyl aminopeptidase
YNL075W	Down expressed	IMP4	Interacting with Mpp10p
YGL150C	Down expressed	INO80	INOsitol requiring
YHR085W	Down expressed	IPI1	Involved in Processing ITS2
YFR055W	Down expressed	IRC7	Increased Recombination Centers
YOR155C	Down expressed	ISN1	IMP-Specific 5'-Nucleotidase
YPR106W	Down expressed	ISR1	Inhibition of Staurosporine Resistance

YGL018C	Down expressed	JAC1	J-type Accessory Chaperone
YJL162C	Down expressed	JJJ2	J-protein (Type III)
YMR132C	Down expressed	JLP2	dnaJ-Like Protein
YGL016W	Down expressed	KAP122	KAryoPherin
YJR054W	Down expressed	KCH1	Potassium (K) regulator of CcH1
YKL161C	Down expressed	KDX1	Kinase Dead X-talker
YGL203C	Down expressed	KEX1	Killer EXpression defective
YDR532C	Down expressed	KRE28	0
YOR336W	Down expressed	KRE5	Killer toxin REsistant
YNL308C	Down expressed	KRI1	KRR1-Interacting protein
YGR040W	Down expressed	KSS1	Kinase Suppressor of Sst2 mutations
YNL029C	Down expressed	KTR5	Kre Two Related
YMR296C	Down expressed	LCB1	Long-Chain Base
YLR260W	Down expressed	LCB5	Long-Chain Base
YER127W	Down expressed	LCP5	Lethal with Conditional Pap1
YOL047C	Down expressed	LDS2	Lipid Droplets in Sporulation
YOR123C	Down expressed	LEO1	LEft Open reading frame
YHR002W	Down expressed	LEU5	LEUcine biosynthesis
YDL051W	Down expressed	LHP1	La-Homologous Protein
YHR156C	Down expressed	LIN1	LINk
YKL205W	Down expressed	LOS1	Loss Of Suppression
YDR439W	Down expressed	LRS4	Loss of RDNA Silencing
YKL176C	Down expressed	LST4	Lethal with Sec Thirteen
YNL260C	Down expressed	LTO1	required for biogenesis of the Large ribosomal subunit and initiation of Translation in Oxygen
YKL143W	Down expressed	LTV1	Low Temperature Viability
YLR352W	Down expressed	LUG1	Lets [URE3]/ure2 Grow
YER142C	Down expressed	MAG1	3-MethylAdenine DNA Glycosylase
YLR427W	Down expressed	MAG2	0
YEL053C	Down expressed	MAK10	MAintenance of Killer
YDR060W	Down expressed	MAK21	MAintenance of Killer
YPR051W	Down expressed	MAK3	MAintenance of Killer
YGR292W	Down expressed	MAL12	MALtose fermentation
YBR297W	Down expressed	MAL33	MALtose
YCR039C	Down expressed	MATALPHA 2	MATing type protein ALPHA
YDL003W	Down expressed	MCD1	Mitotic Chromosome Determinant
YDR318W	Down expressed	MCM21	MiniChromosome Maintenance
YGL201C	Down expressed	MCM6	MiniChromosome Maintenance
YLR253W	Down expressed	MCP2	Mdm10 Complementing Protein

YOL076W	Down expressed	MDM20	Mitochondrial Distribution and Morphology
YBR136W	Down expressed	MEC1	Mitosis Entry Checkpoint
YHR058C	Down expressed	MED6	MEDiator complex
YNL210W	Down expressed	MER1	MEiotic Recombination
YIR017C	Down expressed	MET28	METhionine
YFR011C	Down expressed	MIC19	MItochondrial contact site and Cristae organizing system
YGL035C	Down expressed	MIG1	Multicopy Inhibitor of GAL gene expression
YOR330C	Down expressed	MIP1	MItochondrial DNA Polymerase
YDR144C	Down expressed	MKC7	Multicopy suppressor of Kex2 Cold sensitivity
YNL085W	Down expressed	MKT1	Maintenance of K2 Killer Toxin
YGL106W	Down expressed	MLC1	Myosin Light Chain
YPR188C	Down expressed	MLC2	Myo1p Light Chain
YNL074C	Down expressed	MLF3	Multicopy suppressor of LeFlunomide sensitivity
YKR095W	Down expressed	MLP1	Myosin-Like Protein
YLR320W	Down expressed	MMS22	Methyl MethaneSulfonate sensitivity
YMR177W	Down expressed	MMT1	Mitochondrial Metal Transporter
YLR057W	Down expressed	MNL2	MaNnosidase-Like protein
YPL050C	Down expressed	MNN9	MaNNosyltransferase
YNR059W	Down expressed	MNT4	MaNnosylTransferase
YJR074W	Down expressed	MOG1	Multicopy suppressor Of ts Gsp1
YER068W	Down expressed	MOT2	Modulator Of Transcription
YGL178W	Down expressed	MPT5	Multicopy suppressor of Pop Two
YKL003C	Down expressed	MRP17	Mitochondrial Ribosomal Protein
YHL004W	Down expressed	MRP4	Mitochondrial Ribosomal Protein
YLR312W-A	Down expressed	MRPL15	Mitochondrial Ribosomal Protein, Large subunit
YBR195C	Down expressed	MSI1	Multicopy Suppressor of IRA1
YHR091C	Down expressed	MSR1	Mitochondrial tRNA Synthetase aRginine
YPR134W	Down expressed	MSS18	Mitochondrial Splicing System
YPL097W	Down expressed	MSY1	Mitochondrial aminoacyl-tRNA Synthetase, tyrosine (Y)
YGR042W	Down expressed	MTE1	Mph1-associated TElomere maintenance protein
YDR277C	Down expressed	MTH1	MSN Three Homolog
YGR158C	Down expressed	MTR3	MRNA TRansport
YKL074C	Down expressed	MUD2	Mutant U1 Die
YPL070W	Down expressed	MUK1	coMpUtationally-linked to Kap95
YHR023W	Down expressed	MYO1	MYOsin
YNL036W	Down expressed	NCE103	NonClassical Export
YGL211W	Down expressed	NCS6	Needs Cla4 to Survive
YDL085W	Down expressed	NDE2	NADH Dehydrogenase, External
YML120C	Down expressed	NDI1	NADH Dehydrogenase Internal

YKL040C	Down expressed	NFU1	NifU-like protein
YBR089C-A	Down expressed	NHP6B	Non-Histone Protein
YHR170W	Down expressed	NMD3	Nonsense-Mediated mRNA Decay
YGR089W	Down expressed	NNF2	0
YDL148C	Down expressed	NOP14	NucleOlar Protein
YER002W	Down expressed	NOP16	NucleOlar Protein
YPL043W	Down expressed	NOP4	NucleOlar Protein
YOL144W	Down expressed	NOP8	NucleOlar Protein
YPR072W	Down expressed	NOT5	Negative On TATA
YCR026C	Down expressed	NPP1	ecto-Nucleotide Pyrophosphatase/Phosphodiesterase
YJR062C	Down expressed	NTA1	N-Terminal Amidase
YJL208C	Down expressed	NUC1	NUClease
YER105C	Down expressed	NUP157	NUclear Pore
YDL089W	Down expressed	NUR1	NUclear Rim1
YPR091C	Down expressed	NVJ2	Nucleus-Vacuole Junction
YNL056W	Down expressed	OCA2	Oxidant-induced Cell cycle Arrest
YPL134C	Down expressed	ODC1	OxoDicarboxylate Carrier
YBR230C	Down expressed	OM14	Outer Membrane Protein of 14 kDa
YIL136W	Down expressed	OM45	Outer Membrane
YDR316W	Down expressed	OMS1	OXA1 Multicopy Suppressor
YLL004W	Down expressed	ORC3	Origin Recognition Complex
YGR038W	Down expressed	ORM1	0
YJR051W	Down expressed	OSM1	OSMotic sensitivity
YDR071C	Down expressed	PAA1	PolyAmine Acetyltransferase
YMR165C	Down expressed	PAH1	Phosphatidic Acid phosphoHydrolase
YMR174C	Down expressed	PAI3	Proteinase A Inhibitor
YDR251W	Down expressed	PAM1	Pp2A Multicopy suppressor
YDL173W	Down expressed	PAR32	Phosphorylated After Rapamycin
YNR076W	Down expressed	PAU6	seriPAUperin family
YDL053C	Down expressed	PBP4	Pbp1p Binding Protein
YDL127W	Down expressed	PCL2	Pho85 CycLin
YER059W	Down expressed	PCL6	Pho85 CycLin
YEL058W	Down expressed	PCM1	PhosphoaCetylglucosamine Mutase
YBR222C	Down expressed	PCS60	Peroxisomal CoA-dependent Synthetase
YLR266C	Down expressed	PDR8	Pleiotropic Drug Resistance
YDR113C	Down expressed	PDS1	Precocious Dissociation of Sisters
YCR044C	Down expressed	PER1	protein Processing in the ER
YDR079W	Down expressed	PET100	PETite colonies
YBL080C	Down expressed	PET112	PETite colonies

YDL065C	Down expressed	PEX19	PEroXisome related
YNL326C	Down expressed	PFA3	Protein Fatty Acyltransferase
YOL136C	Down expressed	PFK27	6-PhosphoFructo-2-Kinase
YKL127W	Down expressed	PGM1	PhosphoGlucoMutase
YGL023C	Down expressed	PIB2	PhosphatidylInositol(3)-phosphate Binding
YLR273C	Down expressed	PIG1	Protein Interacting with Gsy2p
YDR501W	Down expressed	PLM2	PLasmid Maintenance
YER132C	Down expressed	PMD1	Paralog of MDS3
YBL035C	Down expressed	POL12	POLymerase
YNL262W	Down expressed	POL2	POLymerase
YAL033W	Down expressed	POP5	Processing Of Precursor RNAs
YGR030C	Down expressed	POP6	Processing Of Precursor RNAs
YPL188W	Down expressed	POS5	PerOxide Sensitive
YLR014C	Down expressed	PPR1	Pyrimidine Pathway Regulation
YOR362C	Down expressed	PRE10	PRoteinase yscE
YPL192C	Down expressed	PRM3	Pheromone-Regulated Membrane protein
YML047C	Down expressed	PRM6	Pheromone-Regulated Membrane protein
YDR243C	Down expressed	PRP28	Pre-mRNA Processing
YDR473C	Down expressed	PRP3	Pre-mRNA Processing
YGR091W	Down expressed	PRP31	Pre-mRNA Processing
YGR075C	Down expressed	PRP38	Pre-mRNA Processing
YGL120C	Down expressed	PRP43	Pre-mRNA Processing
YBR055C	Down expressed	PRP6	Pre-mRNA Processing
YOL061W	Down expressed	PRS5	PhosphoRibosylpyrophosphate Synthetase
YOL054W	Down expressed	PSH1	Pob3/Spt16 Histone associated
YOR090C	Down expressed	PTC5	Phosphatase Two C
YOR208W	Down expressed	PTP2	Protein Tyrosine Phosphatase
YPL212C	Down expressed	PUS1	PseudoUridine Synthase
YLR142W	Down expressed	PUT1	Proline UTilization
YCR057C	Down expressed	PWP2	Periodic tryptophan (W) Protein
YGR280C	Down expressed	PXR1	PinX1-Related gene
YIL120W	Down expressed	QDR1	QuiniDine Resistance
YEL037C	Down expressed	RAD23	RADiation sensitive
YER171W	Down expressed	RAD3	RADiation sensitive
YNL098C	Down expressed	RAS2	homologous to RAS proto-oncogene
YOR301W	Down expressed	RAX1	Revert to Axial
YJR030C	Down expressed	RBH2	Ran Binding domain Homolog
YMR274C	Down expressed	RCE1	Ras and a-factor Converting Enzyme
YDR003W	Down expressed	RCR2	Resistance to Congo Red

YLR106C	Down expressed	REA1	Ribosome Export/Assembly
YLR263W	Down expressed	RED1	REDuctional division
YIL057C	Down expressed	RGI2	Respiratory growth induced
YDL138W	Down expressed	RGT2	Restores Glucose Transport
YHR197W	Down expressed	RIX1	RIbosome eXport
YDL001W	Down expressed	RMD1	Required for Meiotic nuclear Division
YGR044C	Down expressed	RME1	Regulator of MEiosis
YLR145W	Down expressed	RMP1	RNase MRP Protein
YDR465C	Down expressed	RMT2	aRginine MeThyltransferase
YGL144C	Down expressed	ROG1	Revertant Of Glycogen synthase kinase mutation
YOR210W	Down expressed	RPB10	RNA Polymerase B
YBR154C	Down expressed	RPB5	RNA Polymerase B
YHR143W-A	Down expressed	RPC10	RNA Polymerase C
YJL011C	Down expressed	RPC17	RNA Polymerase C
YKR025W	Down expressed	RPC37	RNA Polymerase C
YPR190C	Down expressed	RPC82	RNA Polymerase C
YKR081C	Down expressed	RPF2	Ribosome Production Factor
YMR142C	Down expressed	RPL13B	Ribosomal Protein of the Large subunit
YDR471W	Down expressed	RPL27B	Ribosomal Protein of the Large subunit
YPL131W	Down expressed	RPL5	Ribosomal Protein of the Large subunit
YHR200W	Down expressed	RPN10	Regulatory Particle Non-ATPase
YIL075C	Down expressed	RPN2	Regulatory Particle Non-ATPase
YDR427W	Down expressed	RPN9	Regulatory Particle Non-ATPase
YDL130W	Down expressed	RPP1B	Ribosomal Protein P1 Beta
YDR382W	Down expressed	RPP2B	Ribosomal Protein P2 Beta
YIR015W	Down expressed	RPR2	RNase P Ribonucleoprotein
YKR057W	Down expressed	RPS21A	Ribosomal Protein of the Small subunit
YPL009C	Down expressed	RQC2	Ribosome Quality control Complex
YDR087C	Down expressed	RRP1	Ribosomal RNA Processing
YDR280W	Down expressed	RRP45	Ribosomal RNA Processing
YOR294W	Down expressed	RRS1	Regulator of Ribosome Synthesis
YOL048C	Down expressed	RRT8	Regulator of rDNA Transcription
YPL193W	Down expressed	RSA1	RiboSome Assembly
YCR052W	Down expressed	RSC6	Remodel the Structure of Chromatin
YMR030W	Down expressed	RSF1	ReSpiration Factor
YDR041W	Down expressed	RSM10	Ribosomal Small subunit of Mitochondria
YKL155C	Down expressed	RSM22	Ribosomal Small subunit of Mitochondria
YER125W	Down expressed	RSP5	Reverses Spt- Phenotype
YHR087W	Down expressed	RTC3	Restriction of Telomere Capping

YOR118W	Down expressed	RTC5	Restriction of Telomere Capping
YGL244W	Down expressed	RTF1	Restores TBP Function
YGL252C	Down expressed	RTG2	ReTroGrade regulation
YMR185W	Down expressed	RTP1	Required for the nuclear Transport of RNA Pol II
YOR014W	Down expressed	RTS1	Rox Three Suppressor
YER104W	Down expressed	RTT105	Regulator of Ty1 Transposition
YDR389W	Down expressed	SAC7	Suppressor of ACtin
YGL175C	Down expressed	SAE2	Sporulation in the Absence of spo Eleven
YBR280C	Down expressed	SAF1	SCF Associated Factor
YER129W	Down expressed	SAK1	Snf1 Activating Kinase
YBL052C	Down expressed	SAS3	Something About Silencing
YER019C-A	Down expressed	SBH2	Sec61 beta homolog 2
YPR129W	Down expressed	SCD6	Suppressor of Clathrin Deficiency
YHR205W	Down expressed	SCH9	0
YIR022W	Down expressed	SEC11	SECretory
YLR440C	Down expressed	SEC39	SECretory
YPR055W	Down expressed	SEC8	SECretory
YDR363W-A	Down expressed	SEM1	Suppressor of Exocyst Mutations
YOR184W	Down expressed	SER1	SERine requiring
YOR165W	Down expressed	SEY1	Synthetic Enhancement of YOP1
YLL003W	Down expressed	SFI1	Suppressor of Fermentation Induced loss of stress resistance
YLR403W	Down expressed	SFP1	Split Finger Protein
YPL047W	Down expressed	SGF11	SaGa associated Factor 11kDa
YCL010C	Down expressed	SGF29	SaGa associated Factor
YER096W	Down expressed	SHC1	Sporulation-specific Homolog of CSD4
YOR035C	Down expressed	SHE4	Swi5p-dependent HO Expression
YLR164W	Down expressed	SHH4	SDH4 Homolog
YER118C	Down expressed	SHO1	Synthetic, High Osmolarity-sensitive
YOL110W	Down expressed	SHR5	Suppressor of Hyperactive Ras
YDR227W	Down expressed	SIR4	Silent Information Regulator
YKR072C	Down expressed	SIS2	SIt4 Suppressor
YEL065W	Down expressed	SIT1	Siderophore Iron Transport
YDR328C	Down expressed	SKP1	Suppressor of Kinetochore Protein mutant
YLR139C	Down expressed	SLS1	Synthetic Lethal with SSM4
YFL008W	Down expressed	SMC1	Stability of MiniChromosomes
YOR149C	Down expressed	SMP3	Stable Maintenance of pSRI
YDR525W-A	Down expressed	SNA2	Sensitivity to NA+
YDL123W	Down expressed	SNA4	Sensitivity to NA+
YEL026W	Down expressed	SNU13	Small NUclear ribonucleoprotein associated

YDR425W	Down expressed	SNX41	Sorting NeXin
YFL002C	Down expressed	SPB4	Suppressor of PAB1
YLR146C	Down expressed	SPE4	SPErmidine auxotroph
YHR136C	Down expressed	SPL2	Suppressor of PLc1 deletion
YDR464W	Down expressed	SPP41	Suppressor of PrP4
YER115C	Down expressed	SPR6	SPorulation Regulated
YGL207W	Down expressed	SPT16	SuPpressor of Ty
YER022W	Down expressed	SRB4	Suppressor of RNA polymerase B
YCR081W	Down expressed	SRB8	Suppressor of RNA polymerase B
YML034W	Down expressed	SRC1	Spliced mRNA and Cell cycle regulated gene
YDL092W	Down expressed	SRP14	Signal Recognition Particle
YKL122C	Down expressed	SRP21	Signal Recognition Particle
YKR092C	Down expressed	SRP40	Serine Rich Protein
YPR088C	Down expressed	SRP54	Signal Recognition Particle 54-kD subunit
YKL218C	Down expressed	SRY1	Serine Racemase of Yeast
YBR169C	Down expressed	SSE2	Stress Seventy subfamily E
YNL025C	Down expressed	SSN8	Suppressor of SNf1
YPL232W	Down expressed	SSO1	Supressor of Sec One
YHR184W	Down expressed	SSP1	0
YOR219C	Down expressed	STE13	STErile
YOR212W	Down expressed	STE4	STErile
YPR086W	Down expressed	SUA7	Suppressor of Upstream AUG
YPR151C	Down expressed	SUE1	0
YDR310C	Down expressed	SUM1	SUppresor of Mar1-1
YDR297W	Down expressed	SUR2	SUppressor of Rvs161 and rvs167 mutations
YHR181W	Down expressed	SVP26	Sed5 Vesicle Protein
YAL011W	Down expressed	SWC3	SWr Complex
YGR002C	Down expressed	SWC4	SWr Complex
YAR042W	Down expressed	SWH1	0
YDR260C	Down expressed	SWM1	Spore Wall Maturation
YNL081C	Down expressed	SWS2	Sick Without Securin
YAL014C	Down expressed	SYN8	SYNtaxin
YGL112C	Down expressed	TAF6	TATA binding protein-Associated Factor
YPR048W	Down expressed	TAH18	Top1T722A mutant Hypersensitive
YIL129C	Down expressed	TAO3	Transcriptional Activator of OCH1
YOR086C	Down expressed	TCB1	Three Calcium and lipid Binding domains (TriCalBins)
YHR159W	Down expressed	TDA11	Topoisomerase I Damage Affected
YER071C	Down expressed	TDA2	Topoisomerase I Damage Affected
YIL039W	Down expressed	TED1	Trafficking of Emp24p/Erv25p-dependent cargo Disrupted

YGR099W	Down expressed	TEL2	TELomere maintenance
YNL253W	Down expressed	TEX1	TrEX component
YDR058C	Down expressed	TGL2	TriacylGlycerol Lipase
YBR240C	Down expressed	THI2	THIamine metabolism
YIL078W	Down expressed	THS1	THreonyl tRNA Synthetase
YHR005C-A	Down expressed	TIM10	Translocase of the Inner Membrane
YGR181W	Down expressed	TIM13	Translocase of the Inner Mitochondrial membrane
YEL020W-A	Down expressed	TIM9	Translocase of the Inner Mitochondrial membrane
YLR136C	Down expressed	TIS11	similar to the mammalian TPA Induced Sequence gene family
YIL137C	Down expressed	TMA108	Translation Machinery Associated
YJR014W	Down expressed	TMA22	Translation Machinery Associated
YER175C	Down expressed	TMT1	Trans-aconitate MethylTransferase
YBL054W	Down expressed	TOD6	Twin Of Dot6p
YLL028W	Down expressed	TPO1	Transporter of POlyamines
YOR256C	Down expressed	TRE2	Transferrrin REceptor like
YDL201W	Down expressed	TRM8	Transfer RNA Methyltransferase
YML014W	Down expressed	TRM9	TRna Methyltransferase
YER090W	Down expressed	TRP2	TRyPtophan
YDR353W	Down expressed	TRR1	ThioRedoxin Reductase
YDR407C	Down expressed	TRS120	TRapp Subunit
YGR209C	Down expressed	TRX2	ThioRedoXin
YCR084C	Down expressed	TUP1	dTMP-UPtake
YBR166C	Down expressed	TYR1	TYRosine
YPR066W	Down expressed	UBA3	UBiquitin-like protein-Activating enzyme
YDR059C	Down expressed	UBC5	UBiquitin-Conjugating
YEL012W	Down expressed	UBC8	UBiquitin-Conjugating
YDL122W	Down expressed	UBP1	UBiquitin-specific Protease
YER151C	Down expressed	UBP3	UBiquitin-specific Protease
YGR048W	Down expressed	UFD1	Ubiquitin Fusion Degradation protein
YKL010C	Down expressed	UFD4	Ubiquitin Fusion Degradation protein
YPL186C	Down expressed	UIP4	Ulp1 Interacting Protein
YDR207C	Down expressed	UME6	Unscheduled Meiotic gene Expression
YMR271C	Down expressed	URA10	URAcil requiring
YGL098W	Down expressed	USE1	Unconventional SNARE in the ER
YDL058W	Down expressed	USO1	yUSOu - transport in Japanese
YJL109C	Down expressed	UTP10	U Three Protein
YBL004W	Down expressed	UTP20	U Three Protein
YDR324C	Down expressed	UTP4	U Three Protein
YDR398W	Down expressed	UTP5	U Three Protein

YDR449C	Down expressed	UTP6	U Three Protein
YJR049C	Down expressed	UTR1	Unidentified TRanscript
YNL054W	Down expressed	VAC7	VACuolar segregation
YER064C	Down expressed	VHR2	VHt1 Regulator
YOR054C	Down expressed	VHS3	Viable in a Hal3 Sit4 background
YIL017C	Down expressed	VID28	Vacuolar Import and Degradation
YGR105W	Down expressed	VMA21	Vacuolar Membrane Atpase
YHR060W	Down expressed	VMA22	Vacuolar Membrane Atpase
YNL321W	Down expressed	VNX1	Vacuolar Na+/H+ eXchanger
YGR106C	Down expressed	VOA1	V0 Assembly protein
YLR396C	Down expressed	VPS33	Vacuolar Protein Sorting
YGL095C	Down expressed	VPS45	Vacuolar Protein Sorting
YDR200C	Down expressed	VPS64	Vacuolar Protein Sorting
YPR087W	Down expressed	VPS69	Vacuolar Protein Sorting
YLR181C	Down expressed	VTA1	VpsTwenty Associated
YDL224C	Down expressed	WHI4	WHIskey
YML007W	Down expressed	YAP1	Yeast AP-1
YER123W	Down expressed	YCK3	Yeast Casein Kinase
YIL006W	Down expressed	YIA6	0
YLR200W	Down expressed	YKE2	Yeast ortholog of mouse KE2
YHL014C	Down expressed	YLF2	0
YKL067W	Down expressed	YNK1	Yeast Nucleoside diphosphate Kinase
YML027W	Down expressed	YOX1	Yeast homeobOX
YDR352W	Down expressed	YPQ2	Yeast PQ-loop protein
YKR014C	Down expressed	YPT52	Yeast Protein Two
YHR017W	Down expressed	YSC83	0
YDR326C	Down expressed	YSP2	Yeast Suicide Protein
YOR272W	Down expressed	YTM1	0
YIR026C	Down expressed	YVH1	Yeast vaccinia virus VH1 Homolog
YMR273C	Down expressed	ZDS1	Zillion Different Screens
YNL310C	Down expressed	ZIM17	ZInc finger Motif protein of 17 kDa
YKL175W	Down expressed	ZRT3	Zinc-Regulated Transporter
YAR029W	Down expressed		
YBL010C	Down expressed		
YBL100C	Down expressed		
YBR013C	Down expressed		
YBR085C-A	Down expressed		
YBR284W	Down expressed		
YCR051W	Down expressed		

YCR061W	Down expressed		
YDL009C	Down expressed		
YDL109C	Down expressed		
YDL211C	Down expressed		
YDR239C	Down expressed		
YDR306C	Down expressed		
YER034W	Down expressed		
YER152C	Down expressed		
YFL068W	Down expressed		
YFR018C	Down expressed		
YGL185C	Down expressed		
YGR201C	Down expressed		
YHR131C	Down expressed		
YHR202W	Down expressed		
YIL108W	Down expressed		
YIL161W	Down expressed		
YIR042C	Down expressed		
YIR043C	Down expressed		
YKR078W	Down expressed		
YLR297W	Down expressed		
YLR446W	Down expressed		
YML108W	Down expressed		
YML119W	Down expressed		
YMR046C	Down expressed		
YMR253C	Down expressed		
YNL046W	Down expressed		
YNL108C	Down expressed		
YNL115C	Down expressed		
YNR014W	Down expressed		
YNR048W	Down expressed		
YNR061C	Down expressed		
YNR065C	Down expressed		
YOR192C-B	Down expressed		
YOR296W	Down expressed		
YPR097W	Down expressed		
YPR148C	Down expressed		
YPR174C	Down expressed		
ARA11	Down expressed		
ARA24	Down expressed		

ARA29	Down expressed		
ARA3	Down expressed		
ARA30	Down expressed		
ARA9	Down expressed		
YAR066W	Down expressed		
YBL071C	Down expressed		
YBL081W	Down expressed		
YBL094C	Down expressed		
YBR012C	Down expressed		
YBR190W	Down expressed		
YBR292C	Down expressed		
YCL007C	Down expressed		
YCR013C	Down expressed		
YCR022C	Down expressed		
YDL118W	Down expressed		
YDL158C	Down expressed		
YDL162C	Down expressed		
YDL163W	Down expressed		
YDL237W	Down expressed		
YDR054C-R	Down expressed		
YDR396W	Down expressed		
YDR413C	Down expressed		
YEL014C	Down expressed		
YEL035C	Down expressed		
YER066C-A	Down expressed		
YER121W	Down expressed		
YER137C	Down expressed		
YFL015C	Down expressed		
YGL132W	Down expressed		
YGL182C	Down expressed		
YGL199C	Down expressed		
YGL217C	Down expressed		
YGR069W	Down expressed		
YGR153W	Down expressed		
YGR160W	Down expressed		
YGR259C	Down expressed		
YHR139C-A	Down expressed		
YIL171W	Down expressed		
YJL120W	Down expressed		

YJL152W	Down expressed		
YJR079W	Down expressed		
YJR157W	Down expressed		
YKL083W	Down expressed		
YKR015C	Down expressed		
YKR033C	Down expressed		
YLR031W	Down expressed		
YLR159W	Down expressed		
YLR161W	Down expressed		
YLR171W	Down expressed		
YLR267W	Down expressed		
YLR302C	Down expressed		
YLR331C	Down expressed		
YLR428C	Down expressed		
YMR057C	Down expressed		
YMR122C	Down expressed		
YMR194C-A	Down expressed		
YMR290W-A	Down expressed		
YNL028W	Down expressed		
YNL033W	Down expressed		
YNL114C	Down expressed		
YNL179C	Down expressed		
YNL319W	Down expressed		
YOL013W-A	Down expressed		
YOL099C	Down expressed		
YOL150C	Down expressed		
YOR072W	Down expressed		
YOR146W	Down expressed		
YOR200W	Down expressed		
YOR277C	Down expressed		
YPL044C	Down expressed		
YPR084W	Down expressed		
YPR123C	Down expressed		
YPR177C	Down expressed		

Table S4 Results from the analysis of mutated TFs and their association with DEGs using YeTFaSC. "% in user set" refers the percentage of genes which transcription is potently associated to the TF out of the set provided by the user. "% in scerevisae" is the percentage of genes associated to this TF out of the total genes in the genome.

**AT22**				
**Up regulated genes**				
**Transcription Factor**	**% in user set**	**% in scerevisiae**	**p-value**	**Target ORF/Genes**
Bas1	47.16%	12.43%	0.000923	MMS4 YBR124W SHE3 TBS1 KTR4 FTH1 YBP1 TDP1 ALG7 RIF1 YBR284W YCL065W DCC1 ADY2 PGK1 SRD1 MAK32 HCM1 AHC2 FIG2 GYP7 UGA4 NUS1 VCX1 PCL2 YDL124W YFH1 QRI7 UBX3 MBP1 MCH1 PBP4 YDR018C DAS2 RAD28 YDR042C UBC5 TVP23 SLU7 VTC5 GRX3 VBA4 TRM1 ECM18 SWF1 MTC5 YDR131C PEX7 HMO1 SND1 YDR187C GTB1 IVY1 GPI11 RAD34 OMS1 ASP1 MRPL35 GPI8 IRC3 ATO3 DIT1 GPI1 9 PHO8 CWC21 VPS3 KRE28 YEL073C NPR2 VMA8 PAU2 ANP1 YEL023C YEL014C MIT1 PMI40 YER010C GPA2 GAL83 YER034W ARB1 SAH1 TPA1 FCY22 YER079W DOT6 SHO1 VFA1 COM2 RPS26B SPT15 YER156C YER181C AQY3 OTU1 LAM5 GAT1 MIC19 YFR018C ROG3 IRC5 IRC6 IRC7 YGL262W HXK2 PDE1 ATG1 MPT5 ARI1 MRM2 RPL1B CWC23 YGL117W YGL108C SCY1 SGF73 PUS2 RPT6 YGL036W MIG1 RPL24A PM A1 YGR026W COX18 ENV11 MRPL25 YGR079W PDC6 CLB6 YGR114C YGR125W TPO2 YGR139W PSD2 CRH1 FYV8 MRPL9 SOL4 YGR259C C WC22 YHL044W MUP3 GUT1 OCA5 PRS3 QCR10 RPL27A YHR022C SLT2 YHR033W SAE3 RTC3 ANS1 YAP1801 ENO2 GND1 SSP1 CRG1 FLO5 YHR214W- A YIL152W AYR1 YIL108W XBP1 SGA1 YIL092W VID28 PDR11 DSN1 NRE1 OPT1 PHO90 ATG27 RPL17B KRE9 SET2 RPA34 URA2 IME2 SAG1 TES1 MLO127 OSM1 YJR114W STE24 IBA57 STR2 AAD10 YRA2 PRS1 PIR3 KDX1 RPS27A DBR1 RPT1 GFA1 YKL023W PRY2 RPC37 DAL80 RH O4 YKR078W SRL3 PTR2 YCT1 FRA1 POM33 LMO1 RTT109 DNM1 THI73 IZH3 AFB1 EMA19 SHM2 XDJ1 REX3 SLX4 TIS11 ACF2 ASP3- 4 YLR162W YLR202C FRE1 YLR217W THI7 NDL1 GSY2 LCB5 PIG1 YSH1 ATP14 MET17 CWC24 RPS25B ATG33 CCW14 CTR3 DIF1 RPL6B YML 119W VAN1 TSL1 PRE8 ERV41 SMA2 SUR7 TSA1 MIX17 SOK2 SPO20 YET2 ERB1 FET3 IRC21 SRT1 ILV2 JLP2 TIF34 PAI3 YMR206W EFR3 ERG 8 COX7 SCS7 YMR317W ALP1 LTO1 SUI1 NAR1 JJJ1 SPS19 EAF7 NRK1 TOM70 NCS2 POR1 NCE103 ARK1 DBP6 COQ2 AGA1 AIF1 BDS1 YOL1 62W HPF1 NOP8 CDC33 PHM7 MAM3 DDR2 DNL4 TIR2 BUB3 GLO4 VHS3 MSA1 YOR072W CDC21 TMA46 BAG7 YRR1 YOR186W THI72 BFR 1 YOR199W NOC2 PTP2 STE4 YOR218C RCN2 YOR238W ABP140 SRL1 RPT4 YOR268C PAC1 CAF20 RDL2 BUD7 CPA1 FRT1 PRE10 RDR1 YPL 278C YPL277C DIP5 YPL264C FUM1 VIK1 YPL229W FMP40 SRP72 NAB3 GUP2 RTT10 MRX4 CDC60 PET20 PRM4 RPL33A TAF14 TBF1 HHO1 IDI1 RLM1 SVL3 SMA1 SWI1 LSP1 HAL1 SUT2 YPR015C ATH1 ATG11 MDM36 SUA7 MSS18 SUE1 GDB1 COX1 ATP6 YAT1 HEK2 OLA1 IST2 YB R219C YDR034C-D YER160C IMP2' YPR158C-D YPR158C-C YGR161W-A
Oaf3	4.17%	16.34%	0.008124	YBR116C ADY2 GGC1 PCL2 SND1 GIC2 ATO3 YGR079W YGR139W RTC3 AYR1 SET2 YJR114W YKR012C PRY2 YKR032W PTR2 RTT109 ASP3- 4 GTR1 SCS7 BNI1 YNR066C YOR072W CDC21 FRT1 YPL261C PHO85 SWI1 LSP1 YAT1 OSH1 IKS1
Put3	13.65%	13.27%	0.016759	FTH1 ADY2 PGK1 YCR022C ABP1 GYP7 GGC1 RPS29B TSC13 MCD1 ECM18 GIR2 GIC2 LSM6 RPL37B YEL059W PMI40 SBH2 FCY2 YER079W RPS26B MIC19 YFR018C CSS2 RMR1 KEX1 YGL182C GTS1 ARI1 YGL074C RPL24A MRP13 PDC6 YGR107W CLB6 YGR122W YGR137W CRH1 Y HL044W MUP3 AIM17 RPL27A SKG6 ENO2 SSP1 AYR1 EPS1 RPA34 SAG1 YJR115W TDA4 STE24 LST4 ZRT3 PIR3 RPS27A TEF4 YKR012C PRY 2 RHO4 PTR2 POM33 COX17 AFB1 TRX1 TIS11 ACS2 FRE1 NDL1 YLR302C MET17 YLR311C TSA1 SPO20 SDD2 YMR304C- A YMR320W ALP1 SUI1 POR1 NCE103 YNR042W AGA1 HPF1 NOP8 WSC3 TIR2 HMS1 MSA1 RCN2 IRC13 ABP140 SRL1 CPA1 FRT1 RDR1 FU M1 CLN2 YPL182C PRM4 HHO1 CAM1 SUT2 YPR015C SUE1 SYN8 HEK2 YBR219C

Pdc2	0.51%	16.00%	0.13601	YDR042C THI73 PDC5 THI7
Dot6	2.65%	13.21%	0.160732	YDL012C EHD3 HMO1 GIC2 YGR139W STR2 VPS51 BRE2 YNR066C HPF1 PFK27 THP1 ETT1 CDC21 UFE1 STE4 FRT1 PRM4 DIG1 SWI1 SUT2
Cat8	2.78%	12.43%	0.237914	YCL065W ADY2 PGK1 ATO3 RGI1 CWC23 AIM17 SAG1 IBA57 PTR2 AFB1 YLR302C MET17 TSL1 AGA1 PET123 FUM1 PRM4 YPL062W VMA13 YAT1 OSH1
**Down regulated genes**				
**Transcription Factor**	**% in user set**	**% in scerevisiae**	**p-value**	**Target ORF/Genes**
Bas1	44.03%	10.93%	0.115892	FES1 YSA1 RAD16 SUP45 ADH5 MRPS9 YSW1 NTC20 PYC2 HIS7 SPO23 TSC10 SDH8 EFM2 UBX7 MRC1 APA1 YCL049C GLK1 RRP7 AGP1 RPS14A MATALPHA1 YCR043C BUD23 ARE1 ATG15 YCR102C HO SHR3 YDL180W AIR2 DLD1 UGA3 FAP7 CMR1 RPC53 ATG9 YET3 PSA1 GPD1 APC11 ATG31 RLI1 SPO71 DPB4 SWI5 KGD2 CTH1 CWC15 UBC1 SPR28 EXG2 MRP1 YPS7 TRP4 CDC40 YHP1 JIP4 EUG1 FPR2 STL1 BIM1 YEN1 VTC1 ALD5 ICP55 YER085C YER091C-A AST2 RPS8B DSE1 GCG1 AGP3 ALR2 MDJ1 UBP6 CNN1 COS12 MTO1 EMC4 YGL230C SAP4 SDT1 COX13 RPL9A PAN2 AFT1 SDS23 ALK1 PDR1 PEX31 TFG2 STF2 MSB2 VMA7 RPS25A YGR045C TAM41 FMP48 MCO32 SPR3 PAC10 RPS23A PHB1 GTO1 RNR4 QCR9 AZR1 MIC26 YGR237C COQ6 RNH70 RIM101 ETP1 DED81 YHR020W RRM3 VMA10 YHK8 PCL5 YHR078W TOM71 SPS100 PEX28 PEX18 VTH1 AIM20 SEC28 MAM33 YIL060W PKP1 DOT5 YAP5 DAL4 VTH2 REE1 YJL213W HAL5 FAR1 SNA3 YAK1 GSH1 SAP185 RNR2 CCT8 APL1 SUI2 MHO1 CDC11 CPA2 JHD2 PTK1 PEX1 HYM1 TPO5 SBA1 YKL107W STB6 YKL071W YKL050C YKL033W-A SFT1 HFL1 YSR3 TRZ1 RPL40B YLL067C JLP1 SDH2 HSP104 SSK1 PAM18 SED5 MEF1 LAM6 RFU1 GAL2 CLF1 NHA1 YLR156W APS1 DPH5 TFS1 PNP1 YCS4 DBP9 YLR287C SFH1 TMA10 GAS2 COX8 DUS4 ATG17 PGA3 NGL3 TUB1 RPS1B CYB2 RPS17A UNG1 TAF4 MSS1 YMR027W RCH1 IMP2 ARA2 FAR3 AIP1 SNO1 SNZ1 YMR114C RPL15B CIN4 RIM11 LDO45 ALD3 EAR1 VTI1 RNA1 GTO3 FCP1 LIP1 ADE4 ERR3 HXT14 CUS2 PRM1 YNL247W YNL195C CHS1 SWT21 NOP13 APC1 FYV6 TEP1 RPL9B SUN4 ARP5 CPR8 YNR029C EGO4 BSC5 YNR071C MED7 SPO21 NTG2 RPS15 MDM38 TAT2 TLG2 YOR019W AHC1 STI1 YOR111W YRM1 LIP5 WTM2 SEC63 HNT3 GCD1 RPN8 DGK1 SPS4 SFG1 PDE2 PRT1 AMF1 YOR389W YPL251W USV1 THI6 OXR1 CTI6 PXA1 CAR1 RPS6A GCR1 MFM1 GRX5 SNF8 ICL2 YPR071W RGC1 KRE6 ARR2 AI3 CNE1 PTA1 RBG1 PRP45 MYO4 FRT2 ADE1 YAR023C UIP3 YAR053W YAR068W ROX3 SHP1 YBL028C RPL19B FUS3 ACH1 HTA2 HTB2 ECM15 UGA2 SCO2 PDX3 FIG1 CST26 QDR3 REB1 REG2 YBR063C SIP4 SCP160 YJL068C MRPL8 YHC3 SNX4 SDC25 YGR161C-C YLR157C-A YBL101W-B YCL020W YJR029W
Dot6	2.68%	12.58%	0.153239	TSC10 AGP1 CYK3 FCF1 PYC1 CAF130 QNS1 EFM4 STE18 YLR416C MSS1 RCH1 EAR1 LIP1 PDR17 WTM2 ARR1 ADE1 YAR068W FUS3
Cat8	2.82%	11.86%	0.222077	MATALPHA1 DLD1 SWI5 STL1 EPL1 SAP4 MNP1 TIM17 TPK3 MEF1 YLR173W GAS2 CYB2 DCP2 MSC6 TIF6 FUS3 ACH1 REG2 SIP4 MRPL8
Oaf3	2.95%	10.89%	0.36151	CYC8 SPO23 TSC10 YCL023C HO CYK3 OSH2 MRP1 YGR022C YHK8 SET4 PAM16 RSM7 MSS1 RCH1 TGL3 MRPL10 THO2 MSC6 YPR076W AI4 MRPL8
Put3	10.87%	9.95%	0.660995	ADH5 AMN1 PDB1 TSC10 UBX7 MRC1 MATALPHA1 BUD23 HO NPC2 SWI5 CTH1 IPK1 NKP1 YHP1 JIP4 STL1 FRD1 YEL010W ALD5 GET2 DSE1 SDT1 YGL072C AFT1 SDS23 TFG2 SEC9 MSB2 VMA7 RPS23A RNR4 TYS1 MTM1 THR1 YHK8 FSH1 UBP7 PKP1 TIR3 DJP1 YJR098C TPO5 EBP2 YKL107W IXR1 SFT1 HSP104 YLR126C TFS1 GAS2 YLR374C YLR416C PGA3 TUB1 ARA2 IOC4 FAR3 ALD3 GTO3 ERR3 PRM1 PDR17 LSM7 YNL057W RSM19 WTM2 ENV9 SFG1 AMF1 USV1 KRE6 CYS3 UIP3 YAR068W YBL028C ACH1 HTA2 RPL4A EXO70 SNX4
Pdc2	0.13%	4.00%	0.751998	THI6
**TAT12**				

Up regulated genes				
**Transcription Factor**	**% in user set**	**% in scerevisiae**	**p-value**	**Target upregulated ORF/Genes**
Hfi1	25.61%	15.21%	0.000021	RXT2 FES1 CMD1 TEF2 MUD1 IFA38 TOS1 ARL1 ERV15 HSM3 PAU3 SFA1 RPO21 RPS29B YDL023C YDR015C PST2 MRH1 SED1 CCW22 RPA14 NBP2 CWC15 HTA1 YAP6 BSC2 ZIP1 DIT1 API2 YDR544C MNN1 GPA2 CHO1 RPL34A GPP2 MET6 RPS8B AVT6 YER119C-A SCS2 SPI1 LAM5 RPL22B STE2 GAT1 VTC2 RPL9A FLC3 MRM2 RPS2 NBP35 MF(ALPHA)2 YGL088W EAT1 VMA7 RPL26B YGR035C SCM4 TWF1 LSB1 RPL24B PEX35 GPI1 ENO1 TNA1 GPA1 MAS2 PCL5 QNS1 RPL42B ENO2 UTP9 CSS1 YIL168W CAB2 RPS24B SYG1 RPL2B TIR3 ERG20 CIS3 FAR1 PHO86 ILV3 YJR018W ANB1 SGM1 IML1 HOM6 TRP3 EMC3 ASH1 FAS1 GPM1 MCR1 CWP1 PUT3 CCE1 RPL14A AUR1 SKG1 COX19 SSK1 COX12 MLO50 RPL22A BUD28 PER33 PET309 RFU1 BUD20 SRL2 HRT3 CCW12 MDL1 CPR6 SSP120 YHC1 EXG1 SPH1 YLR326W RPL26A DIC1 GRX8 SST2 YRF1-5 ERG13 TUB1 HMG1 RPS1B IOC4 FAR3 SNZ1 ASC1 YMR173W-A HSC82 SCJ1 MTF1 HOR7 RSN1 COS1 RPL18B TOS6 PPN2 CHS1 PBR1 RPS3 NSG2 RPS7B POR1 MSG5 YIP3 TIM23 ATP23 MSO1 HOL1 DSE4 RPS19A RRI2 ADH1 MET22 THI20 PEX15 MIM1 TLG2 YOR041C WHI2 YOR225W WTM1 MPD1 MBF1 SNC2 MRS6 GDH1 FIT2 FIT3 RPL36B THI6 PGC1 AIM44 RPL33A SSE1 SSU1 ATP4 YTA6 HTC1 RGL1 GRX5 DIG1 HST2 LSP1 HAT1 RPL43A LTP1 RPL11A RPL32 MAP2 TAT1 NET1 YCL019W YDR170W-A YDR210C-C YDR365W-B YDR365W-A YER159C-A YGR038C-A YLR035C-A YLR157C-A YOL103W-B YOL103W-A YOR142W-B YOR343W-A YBL005W-A YBR012W-B YDR210C-D YDR261W-B YFL002W-B YGR161W-A YHR214C-B YJR029W
**Skn7**	**20.95%**	**15.60%**	**0.000033**	HMLALPHA1 YCL065W HSP30 YDL241W VMA1 RPS29B PSA1 KNH1 PRP9 PST2 SED1 RPA14 RTN1 HSP78 YAP6 YDR341C SAM2 SMT3 API2 URA3 MNN1 RPL34A GPP2 MET6 RPS8B SSA4 SPI1 YER158C STE2 GAT1 PES4 LSB3 GUS1 ADE5,7 SAP4 SHE10 OST5 STR3 HUL5 RPS2 ARC1 RPL28 MF(ALPHA)2 SDS23 PMA1 EAT1 YGR016W MTL1 TIM21 RPL26B SCM4 RPL24B YGR210C ENO1 QCR10 BCD1 RPL42B SHQ1 RPS24B SYG1 TIR3 YAP5 CIS3 FAR1 SFH5 LCB3 URA2 IME2 SAG1 POL32 MIR1 IME1 YJR096W MST1 FAS1 GPM1 YKL097C CWP1 CYT2 HOT13 YKL071W MDM35 PRI2 IXR1 CCP1 HSP104 SSA2 COX19 PDC1 SHM2 HRT3 CCW12 YLR296W EXG1 SPH1 SFH1 FKS1 URA4 SST2 YLR462W YRF1-5 ERG13 BUL2 TSL1 RPM2 TUB1 RPS1B YML053C IOC4 ASC1 NCW1 CIN4 RIM11 LDO45 AIM36 YMR173W-A HSC82 GTO3 HOR7 TPS3 YMR265C DSS1 COS1 TOS6 PCL1 CWC25 YNL234W ADE12 CHS1 RPS3 POR1 HHT2 HHF2 PBI2 SIS1 TIM23 LYS9 SMF1 MDY2 WRS1 ADH1 ATP19 MAM3 YOR029W HMS1 AKR2 RGS2 IDH2 SFL1 GLN4 LCB4 WTM1 ABP140 SRL1 MBF1 PYK2 FIT3 RPL33A CAR1 SSE1 SSU1 HTC1 RGL1 MET12 RPA135 CMR3 LTP1 TDA6 ADE1 RPL32 OLA1 HSP26 SCP160 ZAP1 TDH1 UBX6 KAR2 YLR035C-A YOR343W-A YPR158C-D
Pho4	20.95%	15.01%	0.000297	CMD1 IRA1 IFA38 ARL1 YCL065W RDS1 CDC13 INH1 UGA3 YFH1 RPS29B KNH1 MRH1 CCW22 SEC1 TRM82 HTA1 BTT1 YDR271C YFT2 YHP1 PAD1 URA3 MNN1 CHO1 GPP2 YER085C RPS8B SSA4 SCS2 SPI1 BCK2 GAT1 VTC2 PHO4 SAP4 SDT1 FLC3 SNT2 RPL28 SEH1 LSG1 DST1 PEX31 YGR016W VMA7 YGR026W RPL26B RPL24B XKS1 PUP2 ENO1 YGR273C GPA1 VMA16 BRL1 INM1 PCL5 IRE1 TRR2 ENO2 CTR2 NVJ1 UTP9 CSS1 YIL168W TIR3 RFA3 ERG20 CIS3 FAR1 RPE1 YJL119C YJL118W PHO86 IME2 MPS3 SAG1 ILV3 CBF1 IME1 YJR128W ADD66 FAS1 SRP102 NNR2 CWP1 HOT13 OAR1 MDM35 RPL14A YLL054C YLL032C COX19 COX12 PDC1 FRE8 SHM2 RPL22A BUD28 SPC3 PET309 TFS1 CPR6 RSA3 GCD7 CDC3 RPL26A CIS1 YLR407W RPN13 SST2 BUL2 RPS1B SNZ1 PGM2 YMR119W-A DPI35 RIM11 TPP1 ALD3 YMR173W-A HOR7 RSN1 PRE5 COS1 TOS6 PCL1 YNL234W BNI4 ADE12 PPN2 YNL208W RPS3 RPS7B RHO2 POR1 YNL050C YIP3 IDH1 HHT2 HHF2 PUB1 PBI2 ARC35 DSE4 YOL131W SMF1 RPS19A MDY2 WRS1 MIM1 WHI2 NPT1 WTM1 MBF1 PYK2 RPS12 GDH1 FIT2 FIT3 RPL36B RPL33A YTA6 HTC1 LSP1 FCY1 MYO4 RPL32 HAP3 HSP26 YJL086C NET1 ZAP1 TDH1 IRC8 YCL074W YML100W-A MIP6 YFL002W-B

Cup2	13.74%	15.86%	0.000433	GLK1 HSP30 SFA1 ATG20 RPS29B PSA1 KNH1 PST2 MRH1 NBP2 SEC1 HTA1 IPK1 YHP1 SPI1 YER158C BLM10 YGL188C RPL9A SDS23 ALK1 PMA1 TIM21 YGR139W ENO1 QCR10 BCD1 ENO2 UTP9 TIR3 RFA3 FAR1 BNA1 RAD7 BUD4 IME1 JEN1 MST1 ASH1 NNR2 CMC1 YPK1 HSL1 CWP1 HOT13 RPL14A MRPL20 HSP104 COX19 RSC58 PDC1 MLO50 KIN2 CCW12 YLR140W TFS1 EXG1 SPH1 BUD6 SFH1 GRX8 STP3 REH1 COX8 DUS3 SST2 TSL1 SPT5 ARP9 MUB1 YMR103C PGM2 ECM5 HSC82 HOR7 FCP1 HAS1 TOS6 PCL1 YNL208W NMA111 DCP2 YPT53 POR1 PUB1 PBI2 IDP3 PUL4 DSE4 YOL131W ADH1 ATP19 HMS1 YOR041C WHI2 RFC1 SRL1 FRT1 YOR338W REV1 MRS6 AMF1 NIP100 AIM44 TFB2 HTC1 RGL1 GRX5 HST2 RPL43A AIM2 ATS1 ALK2 NET1 TDH1 IRC8 KAR2 YDR365W-B
Reb1	18.28%	14.98%	0.000837	RXT2 CCZ1 TOS1 ARL1 FZO1 DLD1 CLB3 RPO21 RPS29B TVP15 RPA14 AHA1 RTN1 RKM4 RSC3 PIB1 YDR341C SAM2 API2 URA3 RPL34A GPP2 ARG5,6 MET6 AVT6 YER119C-A SPI1 BLM10 SEC4 ATG18 GUS1 KAP114 DOC1 CSE1 OST5 SDT1 YGL152C FLC3 MRM2 COQ8 ARC1 MF(ALPHA)2 YGL088W MNP1 SDS23 SCM4 CAF130 ENO1 TNA1 VMA16 BCD1 KIC1 NDT80 UTP9 SEC24 UTP25 RPL2B GON7 CIS3 RPE1 IME2 YJL027C ILV3 CPR7 POL32 ANB1 ARP3 MIR1 JJJ3 YJR128W YJR146W FRE2 FAS1 YKL097C YKR005C SSA2 SHM2 RPL22A SRL2 CCW12 DPH5 RPL26A SWC7 ERG13 YML053C TAF11 MUB1 YMR103C PGM2 YMR119W-A NCW1 NUP53 ALD3 YMR173W-A HSC82 CEF1 SCJ1 HOR7 HAS1 COS1 RPL18B URE2 IGO1 DCP2 RHO2 YNL050C HHF2 ASI3 SIS1 ATP23 SOL1 LYS9 NTG2 DIS3 RPB11 HMS1 IRC23 NPT1 WTM1 SRL1 FRT1 PYK2 PGC1 NAN1 CAR1 ATP4 CMR3 APL4 YPR099C MMS1 OLA1 SPT7 SIP4 YJL086C NET1 ZAP1 IRC8 UBX6 YDR170W-A YDR210C-C YDR365W-A YER159C-A YGR038C-A YHL009W-A YLR035C-A YLR157C-A YOL103W-B YOL103W-A MIP6 YOR343W-A YBL005W-A YBR012W-B YDR210C-D YFL002W-B YGR161W-A YHR214C-B YJR029W
Rtg3	12.22%	15.72%	0.001211	FES1 ARL1 HMLALPHA2 YCL065W HSP30 YDL241W YDL187C YFH1 YDL023C PST2 SED1 TVP15 RUB1 EFT2 SMT3 MNN1 RPL34A ARG5,6 YER158C RPL22B SUA5 RPL28 VMA7 SCM4 ENV11 LSB1 BTN2 RPL24B PUP2 ARN1 PCL5 YJL182C ERG20 SFH5 ILV3 BNA1 YJR146W FRE2 ADD66 RPC25 YKL071W CCE1 FOX2 COX12 MLO50 RPL22A BUD28 SRL2 YLR140W SMD3 CPR6 DBP9 CIS1 URA4 RPN13 RPS1B PGM2 HSC82 MTF1 YHM2 HOR7 ADE4 PRE5 CHS1 RPS3 IGO1 PGA2 RPS7B RHO2 POR1 IDH1 HHT2 HHF2 PYP1 SIS1 TIM23 LYS9 COS10 RPS19A RRI2 MDY2 ATP19 HMS1 RGS2 IDH2 YOR225W WTM1 RBL2 MBF1 SNC2 SNX3 FIT2 FIT3 RPL36B CAR1 FCY1 LTP1 MYO4 ADE1 KIN3 RPL32 HSP26 NET1 YCL074W YDR210C-D
Ifh1	7.92%	16.08%	0.004480	TEF2 HMLALPHA1 RPS29B EFT2 RPL34A RPS24A MET6 RPS8B SSA4 RPL22B STE2 MTO1 YGL188C RPL9A MRM2 RPS2 RPL28 MF(ALPHA)2 PMA1 TIM21 RPL26B RPL24B GUT1 RPL42B ENO2 RPS24B RPL2B SAG1 BNA1 YJR146W JEN1 MST1 ASH1 HOT13 SFT1 RPL14A FRE8 RPL22A MEF1 BUD20 YLR184W YLR326W RPL26A REH1 YLR407W SIR3 YRF1-5 RPS1B SNZ1 ASC1 RPL18B PPN2 RPS3 RPS7B RPS19A MIM1 RPS12 MRS6 GDH1 RPL36B AIM44 RPL33A DBP1 RPL43A RPL11A Q0297 RPL32 HSP26

Snf2	32.95%	13.56%	0.005287	RXT2 FES1 TEF2 VMA2 CCZ1 TOS1 FZO1 PGI1 YBR300C HMLALPHA1 PAU3 YDL241W VMA1 AIR2 SFA1 YFH1 YDL023C MRH1 SED1 TMS1 CCW22 RPA14 CDC1 MSC2 EBS1 AHA1 RTN1 SRP101 YDR341C EFT2 RPT3 HKR1 DIG2 API2 MNN1 GPP2 ARG5,6 MET6 RPS8B YER119C-A SCS2 RPL22B STE2 GAT1 VTC2 PHO4 GUS1 CSE1 MTO1 ADE5,7 SDT1 STR3 ROK1 SUA5 RPL9A FLC3 RPS2 COQ8 ARC1 RPL28 LSG1 SDS23 CKB1 PDR1 PMA1 PEX31 VMA7 YGR026W TIM21 LSB1 RPL24B XKS1 YGR210C GPI1 ENO1 TNA1 RNH70 GUT1 QCR10 GPA1 MAS2 INM1 PCL5 QNS1 STE12 NDT80 RPL42B ENO2 UTP9 CSS1 SEC24 UTP25 CAB2 SYG1 RPL2B TIR3 PHO90 ERG20 CIS3 FAR1 YJL150W TIF2 LCB3 RPE1 YJL119C PHO86 MRPL49 RNR2 AVT1 ILV3 CPR7 ANB1 MIR1 NMD5 YJR146W ADD66 FAS1 GPM1 MCR1 RPC25 YPK1 HSL1 CUE2 CYT2 YKL071W MNR2 YKL033W-A PUT3 RPL14A AUR1 OSH6 DBP7 LDB18 HSP104 SSA2 COX19 RSC58 PDC1 SHM2 SRL2 GEP5 CDC45 CCW12 SMD3 DPH5 TFS1 HCR1 SEC13 VPS63 YLR278C COQ11 YLR296W YHC1 EXG1 SPH1 BUD6 FKS1 RPL26A DIC1 GRX8 CDC73 URA4 RPN13 SST2 YLR462W YRF1-5 ERG13 VAN1 TSL1 RPM2 HMG1 RPS1B UNG1 OST6 FAR3 SOV1 MUB1 ASC1 ECM5 HSC82 CEF1 RNH1 HOR7 RSN1 DSS1 ADE4 COS1 RPL18B TOS6 RFC3 YNL285W PRM1 ADE12 PPN2 NSG2 RPS7B PMS1 ARP5 YNL050C IDH1 PYP1 IDP3 ATP23 MSO1 LYS9 HOL1 DSE4 YNR071C BSC6 YOL131W SMF1 RPS19A RRI2 PTH4 WRS1 TRM10 ADH1 PEX15 NTG2 MIM1 DIS3 TLG2 RCL1 HST3 BUB3 HMS1 YOR041C LPL1 CKA2 OST3 NPT1 WTM1 SRL1 YOR283W REV1 PYK2 MNE1 SNX3 RPS12 GDH1 RPL36B PGC1 CSM4 MKK2 SSE1 YPL102C YTA6 DIG1 LSP1 RPA135 CMR3 MRD1 ADE1 KIN3 RPL32 MAP2 PTC3 RER2 TAT1 SCP160 TDH1 KAR2 YCL074W YDR170W-A YDR210C-C YDR365W-B YDR365W-A YER159C-A YGR038C-A YLR035C-A YLR157C-A YOL103W-B YOL103W-A YOR142W-B YOR343W-A YBL005W-A YBR012W-B YDR210C-D YDR261W-B YFL002W-B YGR161W-A YHR214C-B YJR029W
Gzf3	8.50%	15.53%	0.008018	HMLALPHA2 HMLALPHA1 YCL065W RDS1 YDL241W GUD1 MGT1 DLD1 PSA1 SED1 GIS1 HTA1 IPK1 YHP1 YDR544C MNN1 GPP2 SSA4 SPI1 GAT1 SHE10 ALK1 BTN2 MCO14 BCD1 PCL5 SFH5 IME2 BCK1 ILV3 POL32 IME1 ASH1 CWP1 HOT13 YLL032C GPI13 SSA2 COX19 PDC1 MLO50 SHM2 MEF1 HRT3 CCW12 URA4 YRF1-5 YML053C SNZ1 ECM5 BCH1 CUS1 HOR7 PRE5 PCL1 PRM1 CWC25 YNL143C YNL050C YIP3 HHT2 HHF2 LYS9 DSE4 YNR071C RGS2 SRL1 SSE1 SSU1 YPR099C HSP26 NET1 YML100W-A
**Sum1**	10.83%	14.98%	0.009143	FES1 IFA38 FZO1 PGI1 YCL065W YDL023C SED1 TMS1 HTA1 YFT2 DIT1 YER085C AVT6 PES4 ROK1 FLC3 RPS2 YGR026W CAF130 LSB1 TNA1 YGR273C TRR2 NDT80 ENO2 CIS3 ILV3 BNA1 ANB1 CBF1 MIR1 IME1 LIH1 HOM6 EMC3 ADD66 GPM1 NNR2 MCR1 CWP1 RPL14A DBP7 HSP104 COX19 PDC1 MLO50 PER33 SRL2 CPR6 DBP9 FKS1 STP3 URA4 YLR462W YRF1-5 ERG13 SNZ1 YMR119W-A ALD3 COS1 MDJ2 HCH1 PRM1 YNL205C SPS19 POR1 SIS1 SOL1 ARC35 LYS9 HOL1 PUL4 YNR071C YOL163W SPO21 ASE1 NPT1 WTM1 MBF1 DGK1 REV1 PYK2 THI6 PGC1 HTC1 LSP1 PIN3 YBR063C KHA1 NET1 KAR2 YFL002W-B YJR029W

Msn4	37.60%	13.03%	0.028303	FES1 YSA1 TEF2 MUD1 YBR134W IRA1 TOS1 PGI1 HMLALPHA1 YCL065W GLK1 HSP30 CTR86 YCR064C COS7 GUD1 CDC13 SEC31 SNF3 INH1 DLD1 UGA3 RPO21 PSA1 KNH1 DAD1 ATG31 PST2 MRH1 YDR061W SED1 UBC13 GIS1 YDR102C CCW22 RPA14 TRM82 SCC2 AHA1 RTN1 VHS1 HSP78 YAP6 BSC2 ZIP1 PIB1 YDR341C PHO92 EFT2 RPT3 DIT1 YHP1 RIB3 SAM2 SMT3 API2 YDR544C URA3 MNN1 CEM1 GPP2 MET6 RPS8B SSA4 AVT6 SPI1 YER158C LAM5 RPL22B YFL032W CAK1 STE2 ATG18 PES4 PHO4 DOC1 MTO1 SAP4 CDC55 STR3 ROK1 RPL28 NBP35 MNP1 SDS23 PDR1 PMA1 MTL1 YGR035C LST7 ENV11 GTF1 BTN2 MRPS35 XKS1 YGR210C GPI1 PUP2 ENO1 TNA1 ARN1 YSC84 MAS2 NEL1 INM1 PCL5 IRE1 STE12 RPL42B ENO2 CTR2 YIL175W UTP25 CAB2 SYG1 RPL2B YAP5 SWI3 FMP33 CIS3 YJL150W YJL119C YJL118W PHO86 YJL027C RNR2 BNA1 ANB1 IME1 YJR096W JJJ3 LIH1 YJR120W YJR128W IML1 JEN1 MTR2 ASH1 FAS1 GPM1 NNR2 MCR1 RPC25 HSL1 YKL097C CWP1 HOT13 YKL071W YKL053W IXR1 PUT3 AUR1 YKR005C FOX2 SPC34 CCP1 MRPL20 PAU18 JLP1 LDB18 HSP104 SSA2 PDC1 MLO50 YLR053C SHM2 PER33 SRL2 KIN2 CDC45 CCW12 TFS1 PBA1 CPR6 RSA3 SSP120 DBP9 YLR278C EXG1 SPH1 YLR326W FKS1 CIS1 REH1 DUS3 YLR462W YRF1-5 VAN1 TSL1 RPM2 HMG1 CYB2 RCF1 TAF11 FAR3 SNZ1 YMR103C PGM2 ASC1 YMR119W-A NCW1 RIM11 NUP53 AIM36 ALD3 YMR173W-A HSC82 SCJ1 MTF1 CUS1 YHM2 GTO3 HOR7 TPS3 HAS1 PRE5 SNO4 COS1 TOS6 PCL1 YNL285W HCH1 PRM1 RAD50 YNL234W BNI4 PPN2 YNL208W SPS19 RPS3 PGA2 YNL143C DCP2 YPT53 MTQ1 YIP3 IDH1 HHT2 PBI2 IDP3 ASI3 SIS1 TIM23 ATP23 SOL1 EGO4 ARC35 COS10 YOL163W YOL131W RPS19A RRI2 MDY2 ADH1 MAM3 THI20 MIM1 DIS3 RCL1 YOR008C-A HMS1 LPL1 KTR1 OST2 PIN2 RGS2 IDH2 PET123 GLN4 HEM15 DCI1 WTM1 SRL1 MPD1 MBF1 DGK1 FRT1 SNC2 YOR343C PYK2 RPS12 GDH1 FIT2 RPL36B CSM4 RTT10 NAN1 DBP1 CAR1 SSE1 HTC1 MET12 LSP1 RPA135 CMR3 APL4 THP3 RDS3 RPL11A MRD1 RRP9 PIN3 MRP21 OLA1 HSP26 RDH54 KHA1 SIP4 ARP4 NET1 SMC3 MRPL8 TDH1 UBX6 KAR2 YCL074W YDR098C-B YER160C YML045W YML100W-A GIP2 YDR210C-D YJR029W
Uga3	5.59%	15.05%	0.041045	FES1 YBR124W ARL1 SMY2 YBR174C ERV15 YCL065W YDL241W UGA3 HTA1 DYN2 YDR544C MNN1 CDC55 LSB1 BTN2 RPL24B PUP2 FRE2 ADD66 VPS51 SED5 HRT3 DPH5 HCR1 URA4 RPN13 YRF1-5 RPS1B PRE5 PCL1 CSL4 IGO1 RPS7B RHO2 HHT2 HHF2 SIS1 MDY2 NPT1 MBF1 SNC2 PYK2 RPL36B TFB2 HSP26 NET1 YDR210C-D
Rph1	11.29%	13.59%	0.082542	FES1 IFA38 YCL065W YCL002C RDS1 COS7 YDL241W PST2 RPA14 YAP6 SRP101 PIB1 IPK1 PHO92 ECM11 DIG2 GPP2 AVT6 GUS1 CSE1 ADE5,7 PEX14 ARC1 BTN2 XKS1 QCR10 YSC84 MAS2 CSS1 YIL089W RNR2 ILV3 MST1 NNR2 RMA1 CYT2 YKL071W YKL033W-A OSH6 SED5 FRE8 MLO50 SRL2 YLR140W TFS1 ARV1 COQ11 SPH1 YLR326W CIS1 TSL1 TUB1 ARP9 SNZ1 AIM36 HSC82 RFC3 BSC4 CHS1 PGA2 DCP2 YPT53 IDH1 PBI2 SIS1 TIM23 SOL1 YNR071C YOL163W YOL131W RPS19A RRI2 WRS1 MAM3 BUB3 WHI2 NPT1 RFC1 FRT1 AMF1 FIT3 PGC1 CSM4 NIP100 AIM44 TFB2 ELP3 RGL1 GRX5 HST2 RPA135 AIM2 HSP26 SIP4 YJL067W YML100W-A YDR210C-D
Tog1	3.73%	14.68%	0.095742	GLK1 HSP30 YDL023C GIS1 SPI1 YER158C YGR139W QCR10 FOX2 HSP104 MLO50 YLR140W TFS1 HCR1 STP3 COX8 SST2 TSL1 YMR103C PGM2 FCP1 PPN2 CHS1 POR1 BUB3 WHI2 OST2 FRT1 MRS6 LSP1 NET1 UBX6
Mig2	4.89%	13.91%	0.134379	FES1 YSA1 ARL1 YDL241W SNF3 HTA1 VHS1 GPP2 SSA4 ZNF1 MF(ALPHA)2 BTN2 GUT1 NUP159 YJL218W SAG1 JEN1 ADD66 CYT2 DBP7 HRT3 RPS1B TRM12 YMR103C PRE5 TOS6 NSG2 HHT2 HHF2 YNR071C RCL1 RGS2 MBF1 YOR343C SNX3 FIT2 FIT3 ATP4 LSP1 RRP9 NET1 GIP2
Dal81	5.70%	13.00%	0.248616	FES1 YDL241W GUD1 UGA3 TVP15 YDR102C HSP78 SMT3 MNN1 GAT1 TIM21 LSB1 BTN2 MAS2 RFA3 SFH5 BCK1 AVT1 FRE2 ADD66 CMC1 YKL097C CCE1 YLL032C MLO50 BUD28 KIN2 RPS1B DPI35 RNH1 PCL1 YNL143C NRK1 RPS7B HHT2 SIS1 DIS3 MBF1 PYK2 GDH1 PGC1 TFB2 CAR1 HST2 TAT1 HSP26 SIP4 NET1 YDR098C-B
Yrm1	0.12%	9.09%	0.388996	PAN1
War1	0.93%	11.27%	0.488571	SEC4 YGL188C TIM21 DPI35 ALD3 PYP1 YNR029C WHI2
**Down regulated genes**				

Transcription Factor	% in user set	% in scerevisiae	p-value	Target downregulated ORF/Genes
Reb1	18.22%	15.65%	0.000741	PCS60 COS2 CIT2 ADP1 BPH1 GCS1 WHI4 PAR32 NOP14 RGT2 BDF2 MRP10 ARP2 RCR2 EHD3 RSM10 MAK21 SAN1 MKC7 VPS64 UME6 DPP1 YDR306C SUM1 SKP1 CNL1 TFC6 YDR391C YDR396W RPN9 YDR444W RMT2 URC2 SNA2 GLY1 SNU13 UBC8 NOP16 BIM1 SBH2 YER066C-A MOT2 TRP2 NUP157 MAM1 TOG1 RPO41 FRS2 YFR018C IRC7 RTF1 HAP2 YGL182C ATG1 YGL159W PRP43 MLC1 ACB1 ORM1 VOA1 YGR259C PXR1 NPR3 MRP4 DED81 THR1 VMA22 NMD3 SUC2 ESL1 CSM2 STH1 YIL108W MOB1 SLM1 RPN2 YIR042C YIR043C YJL152W YJL007C TMA22 RBH2 OSM1 TOR1 CDC11 GRR1 YJR098C ADO1 ABM1 SRY1 KDX1 LTV1 PGM1 SRP21 GFA1 ASK1 BYE1 YKR015C DID2 SIS2 ESL2 EMC6 DIP2 YLR171W PIG1 YLR297W MMS22 LUG1 NDI1 YML119W YAP1 CDC5 RPL13B PAI3 RTP1 MRPS17 CAT8 LCB1 KRE1 KRI1 POL2 ATX1 YNL115C IMP4 YNR061C CTR9 NOP8 YOL046C RAT1 GCY1 LEO1 SMP3 BFR1 GEP3 YTM1 YOR277C BUD7 KRE5 GRE1 PUS1 HRR25 RSA1 RPL5 YPL044C NOP4 SEC8 UBA3 YPR076W CLB2 ECM1 SWH1 CMC2 SAS3 HEK2 YBR012C GAL1 CSG2 FAT1 UBC4 IKS1 HCA4 YBR219C YMR046C YOR192C-B
War1	1.78%	22.54%	0.006057	AHC2 VPS64 POL5 RAD23 MRPL9 SPL2 MET28 ENT4 YLR297W RCE1 YOR072W YTM1 PUS1 YPR123C AAR2 SAS
Yrm1	0.44%	36.36%	0.007573	YCR061W AZR1 YOR1 TPO
Mig2	5.33%	15.89%	0.035874	BMT2 TYR1 GPX2 MAL33 ARF1 SRP14 ENA1 PAA1 PET100 GRX3 APC4 MTH1 UTP6 IZH1 SIT1 UBC8 YER121W YCK3 RPO41 MIG1 RIX1 SUC2 COA1 RPR2 YNK1 ARC19 AQY2 ENT4 TPO1 YMR027W PAH1 CAT8 KRI1 NAM9 ATG34 PSH1 CBP3 NOP4 ICL2 CUR1 ECM1 POP5 SYN8 TOD6 FUI1 GAL1 CHS3 HCA
Rph1	11.44%	14.43%	0.057083	TYR1 DER1 YBR241C GPX2 MAL33 YCR061W ADH7 GCS1 SNA4 NDE2 BDF2 GCV1 ARP10 APC4 GIR2 UME6 PHM6 DPP1 SEM1 RPN9 UTP6 RPL27B PRP3 SNA2 SIT1 ECM10 UBC8 SHC1 MAG1 DDI2 RMR1 COG1 ATG1 TAF6 HOP2 KSS1 MCO32 CTT1 ATF2 MRP4 SPL2 RPN10 CCT2 AIM19 GTT1 YIR042C BNA2 AAD10 DBR1 YNK1 YLR031W TIS11 SPE4 ASP3-1 ASP3-2 ASP3-3 YLR159W ASP3-4 YLR161W SHH4 BOP2 YLR297W ECM38 YLR302C MAG2 YLR446W GTR1 CUE4 PAH1 PAI3 GAS3 TAF7 URA10 GAS1 PFA3 YNL120C YNL115C KTR5 CRZ1 ALG12 YOL150C PEX11 ATG34 MDM20 RRT8 LDS2 HIR2 BAG7 BFR1 TRE2 SPS4 MIP1 APM1 GRE1 PRM3 UIP4 ODC1 GCR1 SUE1 CYC3 SWH1 UGA2 CSG

Msn4	37.00%	13.43%	0.060096	NHP6B CYC8 CBP6 HSL7 TYR1 SSE2 YBR190W DER1 PCS60 OM14 THI2 YBR241C GPX2 MIC12 SAF1 YBR284W MAL33 FUS1 CIT2 ADP1 YCR013C YCR022C FEN2 YCR051W PWP2 YCR061W FUB1 TUP1 AIM6 YPD1 UGA4 ARF1 PAR32 NOP14 RGT2 PCL2 UBP1 SRP14 NUR1 NDE2 COX9 YDL009C MCD1 GCV1 ENA1 RSM10 TGL2 UBC5 MAK21 MKC7 GIR2 CAB5 COQ4 UME6 YDR215C GTB1 SWM1 MTH1 PHM6 SSD1 YDR306C OMS1 UTP4 YSP2 YDR327W TRR1 SEM1 RGA2 RPP2B NKP1 YDR391C YDR396W HPT1 RPN9 CYM1 SSN2 UTP6 PAC11 YDR491C IZH1 PLM2 GMC1 SLF1 EUG1 URC2 SNA2 GLY1 RAD23 UTR5 UBC8 FMP52 HEM14 FCY2 YER066C-A MOT2 SHC1 YER121W RSP5 LCP5 YER137C MAG1 FMP10 TOG1 LAM5 RPO41 FRS2 YFL015C MIC19 YFR018C CDC14 KEG1 RMR1 MTC3 ATG1 YGL159W PRP43 VPS45 ERV14 STF2 IMO32 ACB1 KSS1 UFD1 CTT1 NNF2 MTR3 YGR160W ATF2 TIM13 TRX2 MRPL9 SLH1 PXR1 MAL12 RIM101 STE20 MRP4 CIC1 MED6 SSF1 IPI1 RTC3 MSR1 SPL2 LIN1 NMD3 RIX1 VTH1 SUC2 AIM20 TMA108 OM45 TAO3 YIL108W MOB1 AIM19 RGI2 YIL054W PIG2 PDR11 DOT5 RPR2 MET28 GTT1 YIR043C IMA5 HXT8 YJL152W UTP10 UTR1 KCH1 TOR1 BNA2 YJR079W ADO1 ECM27 EBP2 RSM22 DBR1 LTV1 PGM1 GFA1 UTP11 BUD2 MDH1 YNK1 ASK1 PRP40 DID2 YKR075C RPF2 PTR2 FLO10 YLL056C FRE6 TPO1 RLP24 AAT2 EMA19 MNL2 DIP2 SLS1 PUT1 YLR159W YLR161W SHH4 MCP2 HSP60 BOP2 YLR279W YLR297W ECM38 YLR302C MRPL15 GMC2 YLR463C NDI1 YML119W ATR1 CUE4 PRM6 YOX1 YAP1 YMR027W CTF18 YMR122C YMR155W PAH1 PAI3 MMT1 MRPS17 YMR194C-A YMR196W TAF7 URA10 RCE1 CAT8 LCB1 FKS3 YMR306C-A GAS1 ATP11 KRI1 BOR1 ATX1 YNL179C NAM9 YNL122C DBP2 SWS2 IMP4 NCE103 LST8 YNR014W ALG12 YNR065C BDS1 YOL150C PEX11 PFK27 TRM11 SHR5 MDM20 YOL075C RRT8 HSP10 CRS5 TMC1 YNG1 TCB1 ECM3 RIO1 GCY1 BAG7 HER1 IRC13 YTM1 RRS1 RAX1 SFG1 KRE5 RAD17 BBP1 SSO1 GRE1 PUS1 UIP4 RPL5 IDI1 GDE1 MSY1 NOG1 BTS1 NOP4 RMI1 RQC2 CIT3 MCM16 GRS2 FHL1 SCD6 ASN1 SUE1 VPS4 YPR174C HDA3 ATG13 CLN3 CYC3 SWC3 FLO1 YAR061W YAR066W YBL100C YBL086C CMC2 TOD6 FUI1 HEK2 UTP20 UGA2 YBR012C YBR013C GAL1 GIP1 RFS1 UBC4 YBR085C-A IML2 HCA4 YBR219C YIL171W YMR046
Rtg3	10.33%	13.92%	0.129674	TYR1 GPX2 MAL33 RRP7 FUS1 CIT2 MAK31 YCR061W ADH7 TRM8 COX9 LHP1 RMD1 ENA1 RSM10 PAA1 PET100 GRX3 CNL1 PRP3 SIT1 NOP16 CHZ1 SAK1 EMP47 IRC7 YGL159W MLC1 USE1 SWC4 TRX2 SLH1 CAB4 VMA22 SPL2 LIN1 OM45 PIG2 HXT8 MOG1 ELF1 DBR1 YNK1 YKR075C PTR2 FLO10 ORC3 DNM1 RLP24 AAT2 TIS11 PUT1 YKE2 BNA5 PDR8 BOP2 ECM38 YLR302C EST2 BER1 YML108W RPL13B TAF7 ZIM17 BOR1 RAS2 NCE103 YOL150C CTR9 NOP8 PFK27 TMC1 TCB1 SER1 BFR1 RPB10 HER1 MOD5 YOR277C COT1 ODC1 IDI1 NOG1 GCR1 YPL034W CIT3 CLB2 POP5 SYN8 YAR061W FUI1 UBC4 AAP
Snf2	30.78%	13.27%	0.132229	DER1 HPC2 MCX1 OM14 GPX2 MAL33 COS2 EMC1 RRP7 CIT2 YCR022C FEN2 PER1 YCR051W RSC6 PWP2 TUP1 YCR087C-A ABP1 TRM8 PAR32 BDF2 PEX19 LHP1 ARP2 RCR2 GCV1 DAS2 RSM10 RRP1 APC4 SAN1 YDR149C GIR2 HMO1 GTB1 RRP45 PHM6 SSD1 SUR2 YDR306C UTP4 YSP2 TRR1 DXO1 RPP2B UTP5 HPT1 SNX41 RPN9 YDR444W RMT2 RPL27B GIN4 SNA2 SIT1 PCM1 GLY1 RAD23 SPF1 SNU13 YEL014C UBC8 FCY2 VHR2 TRP2 SHC1 PRS2 NUP157 SHO1 LCP5 SAK1 PMD1 YER137C UBP3 YER152C RAD3 EMP47 RPO41 FRS2 SPB4 GCN20 MIC19 YFR018C CDC14 KEG1 IRC7 NCS6 SPT16 YGL204C RPL1B PRP43 TAF6 MLC1 VPS45 KAP122 CDH1 ACB1 ORM1 KSS1 RME1 TAM41 PRP31 VOA1 MTR3 YGR160W ATF2 QCR9 PMT6 YGR259C CAB4 PXR1 RIM101 MRP4 TCD1 DED81 MYO1 THR1 RPN1 CIC1 VMA22 RPP1 GEP4 YHR131C SPL2 NMD3 RIX1 RPN10 SCH9 VTH1 SUC2 YIL161W BNR1 AIM20 CCT2 ASG1 TAO3 YIL108W MOB1 AIM19 THS1 RPN2 YIL067C TED1 PDR11 MET28 YVH1 YIR043C NUC1 RBH2 CDC11 YJR079W LST4 ZRT3 ELF1 LTV1 PGM1 AAT1 GFA1 BUD2 PSG1 MUD2 ANR2 UFD4 BYE1 RPC37 YKR045C RPS21A SIS2 YKR075C RPF2 SRP40 PTR2 ESL2 FRE6 TPO1 REA1 DIP2 ASP3-1 ASP3-2 ASP3-3 YLR159W ASP3-4 SHH4 VTA1 BNA5 HSP60 LCB5 RED1 BOP2 YLR302C SEC39 NDI1 ATR1 COG8 MFT1 GSF2 PRM6 SRC1 TRM9 CDC5 RSF1 RNA14 CTF18 YMR122C RPL13B MRPS17 GAS3 CUE1 URA10 YMR279C AEP2 YMR306C-A GAS1 KRE1 YNL266W LTO1 YNL115C YNL108C SWS2 NCE103 BDS1 PFK27 TRM11 INO4 PRS5 RRT8 LDS2 NGL1 YOL013W-A VHS3 YNG1 PTC5 RIO1 YOR200W MGM1 HER1 TRE2 MOD5 YOR277C RRS1 YOR296W MIP1 ERR2 APM1 BBP1 POS5 UIP4 ODC1 RPL5 MSY1 NOG1 MUK1 YPL044C NOP4 ICL2 HTS1 UBA3 YPR076W ECM1 YAR029W YBL100C YBL094C RPL23A YBL081W PRS4 YBL053W FUI1 HEK2 MCM2 UGA2 YBR012C AKL1 HCA4 AAP1 YMR046C YOR192C-

Pho4	17.67%	13.26%	0.214563	ALG1 CYC8 YBR144C TYR1 SSE2 MSI1 AME1 HPC2 GPX2 SDH8 COS2 FUS1 CIT2 ADP1 BPH1 RAD18 WHI4 TRM8 ARP2 YDL009C ATP16 RSM10 FOB1 STB3 ARG82 HMO1 YDR215C PHM6 SUR2 YDR327W TFC6 YDR396W YDR491C SNA2 SIT1 GLY1 TIM9 UBC8 NOP16 MAM1 RSP5 SAK1 YER137C YGL217C ROG1 TAF6 MPC1 ERV14 CUL3 ACB1 TAM41 CTT1 NNF2 VOA1 CAB4 RIM101 NPR3 YLF2 MRP4 TCD1 RPN1 CIC1 MED6 VMA22 RPP1 SSF1 IPI1 RTC3 MSR1 YHR131C SPL2 RPC10 LIN1 NMD3 FMO1 SVP26 SSP1 RIX1 RPN10 VTH1 SUC2 TMA108 OM45 TAO3 PDR11 RPR2 YIR043C MOG1 BNA2 YJR098C ADO1 ABM1 LST4 LTV1 CTK1 UTP11 MDH1 PSG1 ARC19 RPS21A RPF2 HBS1 FLO10 AQY2 FRE6 ENT4 TPO1 TIS11 ACS2 YLR159W YLR161W CCC1 MCP2 PDR8 BOP2 BER1 GTR1 CUE4 RPL13B PAH1 MRPS17 GAS3 GAS1 KRE1 SKP2 RAS2 MKT1 KTR5 YNL028W CRZ1 YNR061C PFK27 APM4 CRS5 TMC1 YNG1 YOR072W PTC5 SEY1 BFR1 HNT3 KRE5 PRE10 SSO1 RPL5 IDI1 GDE1 CLB2 AXL1 ASN1 CLN3 CYC3 SYN8 SWC3 FLO1 YAR061W RFS1 UBC4 YIL171
Gzf3	7.11%	13.62%	0.224686	TYR1 SSE2 GPX2 FUS1 CIT2 MATALPHA2 YCR041W RSC6 ADH7 UGA4 PCL2 GCV1 ENA1 RSM10 PHM6 NOP16 BIM1 MOT2 MAG1 IRC7 RMR1 YGL185C SWC4 PMT6 MAL12 RTC3 MSR1 SPL2 LIN1 SUC2 AIM20 LTV1 YKR075C SRP40 TIS11 PUT1 CCC1 YLR297W YLR463C ATR1 MFT1 YOX1 TRM9 PAH1 GAS3 YMR253C ZIM17 ALP1 ATX1 YNR014W PUL3 BDS1 NOP8 STE4 SFG1 SSO1 SEC8 ISR1 SCD6 SUE1 SWH1 YAR066W FUI1 YIL171
Tog1	3.33%	13.76%	0.262152	TYR1 SSE2 MSI1 TRM8 UME6 PHM6 DXO1 YCK3 YFL068W CDC14 TAF6 STF2 STE20 SPL2 AIM19 KCH1 DIP2 YLR297W NDI1 YOX1 CDC5 TAF7 MER1 MDM20 RRT8 LDS2 HIR2 YTM1 UGA2 YBR219
Dal81	5.44%	13.00%	0.372178	MSI1 GPX2 AIM6 UGA4 UBP1 GRX3 UME6 MTH1 PHM6 SIT1 GLY1 UTR5 VHR2 FRS2 ATG1 RPL1B YSC83 RPN10 KCH1 DBR1 PSG1 SRP40 PTR2 MLP1 YLR031W MNL2 NHA1 SLS1 PUT1 YLR159W YLR161W YLR171W FAR10 PDR8 BOP2 ECM38 YNL319W BOR1 MKT1 YOL013W-A TMC1 SFG1 RAD17 GCR1 NOT5 ASN1 FUI1 UGA2 UBC
Cup2	10.67%	12.90%	0.374536	SSE2 OM14 THI2 YBR241C GPX2 MIC12 CIT2 NOP14 RPP1B PCL2 YDL109C MCD1 GCV1 RSM10 TGL2 UME6 YDR239C MTH1 PHM6 DXO1 NKP1 YDR391C HPT1 JIP4 GMC1 GIN4 SNA2 SIT1 GLY1 RAD23 FCY2 VHR2 TMT1 YFL068W MIC19 RMR1 YBP2 STF2 CTT1 QCR9 TRX2 RTC3 MSR1 GEP4 SPL2 FMO1 AIM20 COA1 AIM19 RGI2 PIG2 SEC11 KCH1 LOS1 LTV1 MDH1 ANR2 EMA19 DIP2 TIS11 RED1 YLR297W MRPL15 MAG2 NDI1 YML084W CDC5 YMR122C RTP1 MRPS17 EFR3 GAS3 TAF7 SKP2 MLF3 INO4 MDM20 RRT8 LDS2 CRS5 HIR2 PTC5 BFR1 YTM1 SFG1 GDE1 NOG1 YPL044C SEC8 CLB2 CLN3 YAR029W YAR066W CMC2 UGA2 GAL
Uga3	4.56%	12.85%	0.406607	TYR1 GPX2 RRP7 MATALPHA2 YCR041W YCR061W ADH7 UGA4 TRM8 PAA1 GRX3 HMO1 PHM6 SUM1 NNF2 CAB4 MSR1 LIN1 RPN10 STH1 ELF1 YPT52 FLO10 YLR463C RPL13B ZIM17 BOR1 YNL115C YNL108C TRM11 TMC1 RPB10 YOR277C IDI1 GCR1 YPL034W ASN1 SWH1 FUI1 UGA2 UBC
Sum1	8.56%	12.40%	0.536783	KTR4 OM14 COS2 YCR013C ADH7 AIM6 YDL211C BDF2 RMD1 SIR4 YDR239C PHM6 SUM1 OMS1 NOP16 NUP157 MAM1 MAG1 YGL199C RPL1B YGR259C PXR1 MSR1 SPL2 SVP26 SSP1 RPN10 AIM19 YVH1 YIR043C YJL152W MDE1 BNA2 YJR079W EAF6 EBP2 LTV1 YKR015C YKR033C DID2 ORC3 AAT2 TIS11 BNA5 PIG1 YLR428C YLR463C ORC1 PRM6 GAS3 KRE1 YNL319W KRI1 MLF3 OCA2 MNT4 YNR061C PAU6 PFK27 ATG34 LDS2 YOL046C TMC1 YOR072W SPS4 SFG1 HFI1 GCR1 YPL044C NOP4 ASN1 SWH1 HEK2 CSG2 FAT1 GIP1 CSH
Skn7	14.78%	11.53%	0.879306	BMT2 SSE2 GPX2 FUS1 CIT2 ADP1 YCR013C YCR022C YCR041W RSC6 ABP1 TRM8 PCL2 MCD1 SUR2 TRR1 CNL1 SEM1 RGA2 RPP2B HPT1 PAC11 GRH1 SNA2 YDR526C SIT1 GLY1 BIM1 SBH2 FCY2 YCK3 YER152C DDI2 FRS2 RMR1 MTC3 MPT5 RPL1B RME1 MCO32 CTT1 VOA1 TRX2 PXR1 YOR1 STE20 LEU5 DED81 THR1 RPN1 MSR1 RPC10 SSP1 SUC2 THS1 RGI2 PDR11 BNA2 YJR079W ADO1 ZRT3 KDX1 LTV1 PGM1 YNK1 DID2 YKR045C YKR075C SRP40 PTR2 MLP1 ESL2 TPO1 DNM1 AAT2 SPE4 YLR159W YLR161W YKE2 CCC1 MCP2 HSP60 YLR297W YLR302C BER1 SEC39 GTR1 ATR1 CUE4 YML084W YAP1 SEG1 SWP1 PAH1 PAI3 YMR196W FKS3 GAS1 KRE1 ATX1 YNL122C RAS2 LST8 YNR014W NOP8 PFK27 RRT8 PTC5 ECM3 BFR1 PTP2 RPB10 HER1 MOD5 SFG1 BBP1 RPL5 BTS1 RMI1 CIT3 YPR097W YPR148C SUE1 VPS4 RPC82 CYC3 FLO1 IMD1 TOD6 HEK2 CHS3 YBR085C-A YIL171

Hfi1	17.89%	11.13%	0.969311	SSE2 AIM4 KTR4 DER1 PCS60 OM14 GPX2 SDH8 MAL33 COS2 EMC1 CIT2 ADP1 MATALPHA2 YCR041W YCR061W AHC2 YCR087C-A ABP1 DLD2 RPP1B BDF2 PEX19 USO1 LHP1 ATP16 GCV1 DAS2 RSM10 GTB1 PHM6 SUR2 UTP4 RPP2B YDR396W HPT1 RPL27B YEL068C GLY1 SNU13 FMP52 FCY2 TRP2 LCP5 MAG1 YER152C RAD24 YER181C LAM5 FRS2 YFR018C IRC7 RPL1B PRP43 MLC1 ERV14 ACB1 KSS1 RME1 UFD1 PRP31 YGR160W ATF2 TIM13 QCR9 YGR259C MRP4 RPP1 SPL2 RIX1 VID28 YIR042C YIR043C RBH2 UTR1 OSM1 KCH1 BNA2 YJR079W RSM22 BUD2 VPS24 NFU1 UFD4 DID4 YPT52 RPS21A MLP1 ESL2 SFI1 DNM1 EMA19 MNL2 DIP2 ASP3-1 ASP3-2 ASP3-3 YLR159W ASP3-4 VTA1 HSP60 LCB5 RED1 PDR8 BOP2 MMS22 JIP3 YLR446W ATR1 COG8 NDC1 YMR027W RPL13B EFR3 GAS3 CUE1 YMR290W-A ATM1 GAS1 YNL115C DBP2 YNL108C NCE103 YOL150C PFK27 RRT8 NGL1 YOL013W-A HSP10 YNG1 BFR1 HER1 TRE2 HNT3 RAX1 KRE5 ERR2 SSO1 POS5 ODC1 RPL5 IDI1 NOG1 GCR1 BTS1 YPL034W HTS1 YPR076W POP5 SWC3 YBL100C RPL23A YBL081W PRS4 HEK2 UTP20 UGA2 YBR064W AAP1 YMR046C YOR192C-
Ifh1	4.22%	8.98%	0.98986	YBR190W BPH1 ARF1 RPP1B PHM6 RPP2B UTP6 RPL27B PLM2 MAK10 YER137C RPL1B VOA1 MRP4 SPL2 CSM2 ATG27 BNA2 KDX1 RPS21A MLP1 CWC24 YLR446W ATR1 RPL13B YNL179C DBP2 RAS2 MLF3 SER1 IRC13 SPS4 SFG1 RPL5 FHL1 FLO1 RPL23A YBR085C-
**TTY23**				
**Up regulated genes**				
**Transcription Factor**	**% in user set**	**% in scerevisiae**	**p-value**	**Target upregulated ORF/Genes**
Sfp1	55.95%	8.58%	1.6627E-06	
Yrr1	32.34%	8.51%	0.00188803	
Pho23	2.78%	10.22%	0.05879699	
Leu3	8.53%	8.04%	0.15812479	
Gal3	1.59%	9.09%	0.16722665	
Aro80	6.55%	7.64%	0.27613842	
Cyc8	0.20%	6.25%	0.31366624	
Cat8	2.38%	6.78%	0.4840607	
Wtm1	1.98%	5.29%	0.79039005	
**DOWN**				
**Transcription Factor**	**% in user set**	**% in scerevisiae**	**p-value**	
Yrr1	33.21%	9.19%	0.00030865	
Sfp1	51.32%	8.27%	0.00529358	
Leu3	9.25%	9.16%	0.05047669	
Wtm1	3.58%	10.05%	0.06760715	
Gal3	1.70%	10.23%	0.11608478	
Pho23	2.45%	9.49%	0.1381444	
Cat8	2.83%	8.47%	0.2407558	
Aro80	5.85%	7.18%	0.53510908	
